# The demise of the giant ape *Gigantopithecus blacki*

**DOI:** 10.1038/s41586-023-06900-0

**Published:** 2024-01-10

**Authors:** Yingqi Zhang, Kira E. Westaway, Simon Haberle, Juliën K. Lubeek, Marian Bailey, Russell Ciochon, Mike W. Morley, Patrick Roberts, Jian-xin Zhao, Mathieu Duval, Anthony Dosseto, Yue Pan, Sue Rule, Wei Liao, Grant A. Gully, Mary Lucas, Jinyou Mo, Liyun Yang, Yanjun Cai, Wei Wang, Renaud Joannes-Boyau

**Affiliations:** 1 Key Laboratory of Vertebrate Evolution and Human Origins, Institute of Vertebrate Paleontology and Paleoanthropology, Chinese Academy of Sciences, Beijing, China; 2 School of Natural Sciences, Faculty of Science and Engineering, Macquarie University, Sydney, New South Wales Australia; 3 School of Culture, History and Languages, ANU College of Asia and the Pacific, Australian National University, Canberra, Australian Capital Territory Australia; 4 GARG, Southern Cross University, Lismore, New South Wales Australia; 5 Department of Anthropology and Museum of Natural History, University of Iowa, Iowa City, IA USA; 6 College of Humanities, Arts and Social Sciences, Flinders University, Adelaide, South Australia Australia; 7 isoTROPIC Research Group, Max Planck Institute for Geoanthropology, Jena, Germany; 8 Department of Archaeology, Max Planck Institute for Geoanthropology, Jena, Germany; 9 School of Social Sciences, University of Queensland, Brisbane, Queensland Australia; 10 School of Earth and Environmental Sciences, University of Queensland, Brisbane, Queensland Australia; 11 National Research Centre on Human Evolution CENIEH, Burgos, Spain; 12 Australian Research Centre for Human Evolution (ARCHE), Griffith University, Brisbane, Queensland Australia; 13 Wollongong Isotope Geochronology Laboratory, School of Earth, Atmospheric and Life Sciences, University of Wollongong, Wollongong, New South Wales Australia; 14 Institute of Cultural Heritage, Shandong University, Qingdao, China; 15 College of Science and Engineering, Flinders University, Adelaide, South Australia Australia; 16 Natural History Museum of Guangxi, Nanning, China; 17 Chongzuo Zhuang Ethnological Musuem, Chongzuo, China; 18 Institute of Global Environmental Change, Xi’an Jiaotong University, Xi’an, China; 19 Palaeo-Research Institute, University of Johannesburg, Johannesburg, South Africa

**Keywords:** Palaeontology, Anthropology, Palaeoclimate

## Abstract

The largest ever primate and one of the largest of the southeast Asian megafauna, *Gigantopithecus blacki*^1^, persisted in China from about 2.0 million years until the late middle Pleistocene when it became extinct^2–4^. Its demise is enigmatic considering that it was one of the few Asian great apes to go extinct in the last 2.6 million years, whereas others, including orangutan, survived until the present^5^. The cause of the disappearance of *G. blacki* remains unresolved but could shed light on primate resilience and the fate of megafauna in this region^6^. Here we applied three multidisciplinary analyses—timing, past environments and behaviour—to 22 caves in southern China. We used 157 radiometric ages from six dating techniques to establish a timeline for the demise of *G. blacki*. We show that from 2.3 million years ago the environment was a mosaic of forests and grasses, providing ideal conditions for thriving *G. blacki* populations. However, just before and during the extinction window between 295,000 and 215,000  years ago there was enhanced environmental variability from increased seasonality, which caused changes in plant communities and an increase in open forest environments. Although its close relative *Pongo weidenreichi* managed to adapt its dietary preferences and behaviour to this variability, *G. blacki* showed signs of chronic stress and dwindling populations. Ultimately its struggle to adapt led to the extinction of the greatest primate to ever inhabit the Earth.

## Abstract (web summary)

A multiproxy record of *Gigantopithecus blacki* provides insights into the ecological context of this species, which became extinct around 250,000 years ago, when increased seasonality led to a change in forest cover.

## Main

**Fig. 1:**
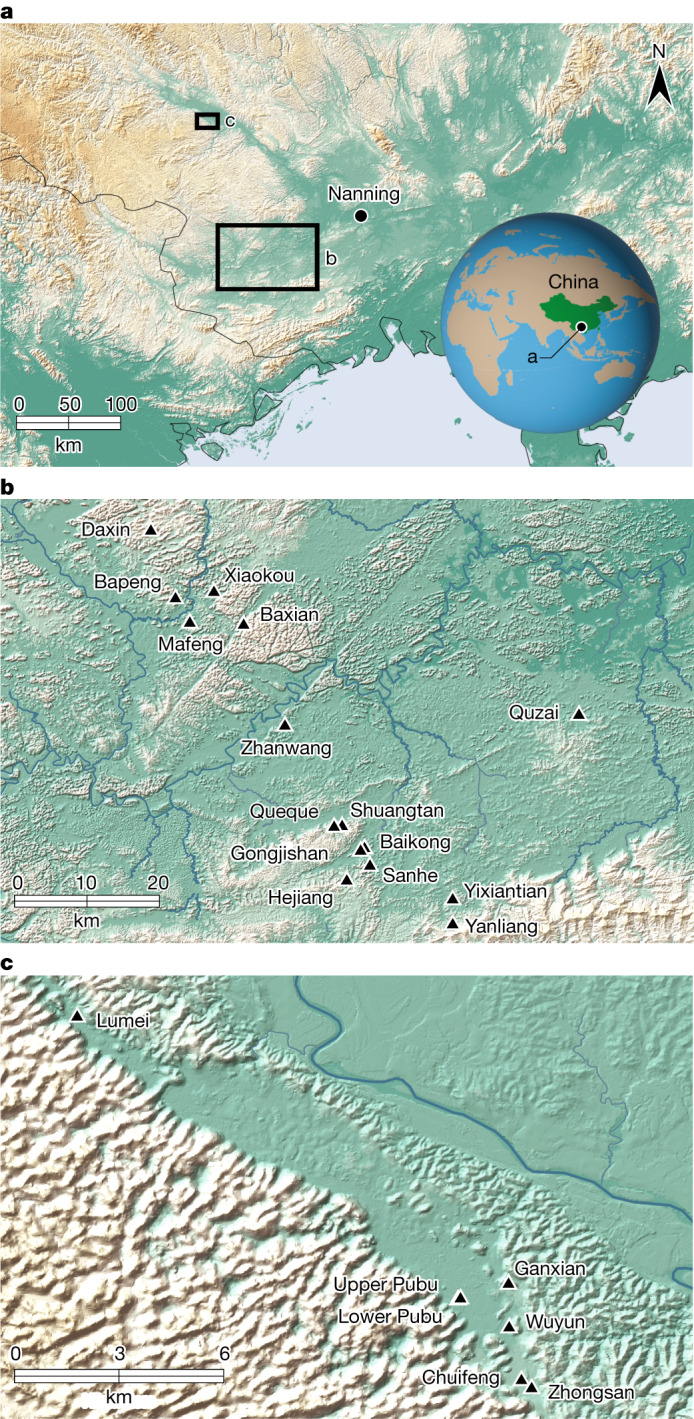
The location of the study sites in this research. **a**–**c**, The location of Southern China, Guangxi ZAR province and the city of Nanning (**a**), with the location of the Chongzuo study area marked by a large box (**b**) and the Bubing Basin study area marked by a smaller box (**c**). **b**, The location of the 16 cave sites analysed in the Chongzuo study area. **c**, The location of the six caves analysed in the Bubing Basin study area including both *G. blacki*-bearing and non-*G. blacki*-bearing caves from both regions.

**Extended Data Fig. 1:**
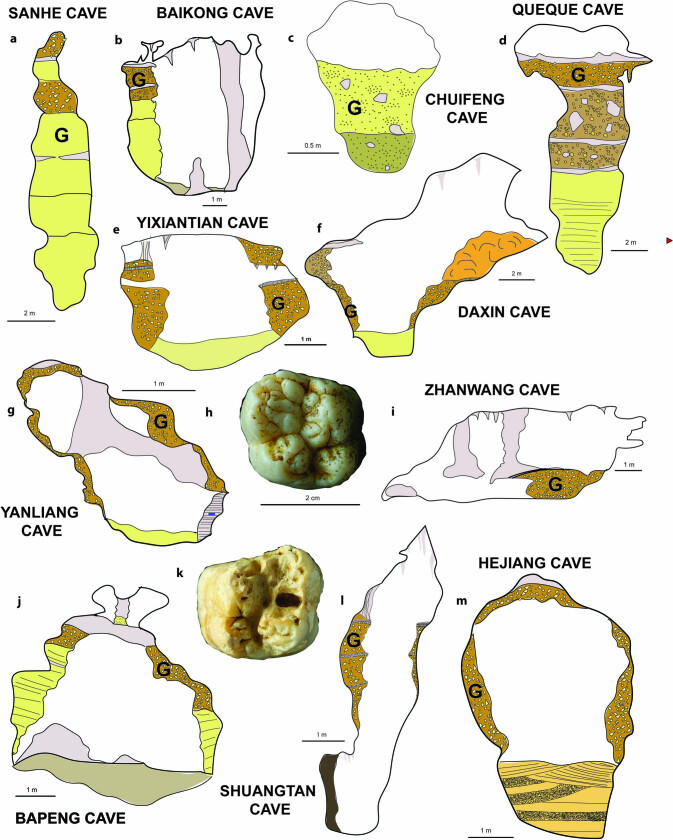
The *G. blacki* caves in Chongzuo and Bubing Basin. **a**, Sanhe; **b**, Baikong; **c**, Chuifeng; **d**, Queque; **e**, Yixiantian; **f**, Daxin; **g**, Yanliang; **h**, a typical *G. blacki* teeth from older evidence from Sanhe cave; **i**, Zhanwang; **j**, Bapeng; **k**, a typical *G. blacki* teeth from younger evidence from Hejiang cave; **l**, Shuangtan; and **m, ** Hejiang. All profiles display the main stratigraphic relationships and the units containing *G. blacki* evidence. See the individual cave plans, profiles and sampling locations plus the keys for the symbols in Fig. S1a–v.

Our current understanding of *Gigantopithecus blacki* derives from Early to Middle Pleistocene cave deposits in southern China between the Yangtze River and the South China Sea (Fig. 1 and Supplementary Information section 1). This pongine^7^ is considered a key member of the Early to Middle Pleistocene *Gigantopithecus*–*Sinomastodon* and *Stegodon–Ailuropoda* faunal zones of (sub)tropical oriental Asia, from about 2.0 million years ago (Ma) to 330 thousand years ago (ka)^2,3,8,9^. It is known for its unusually large molars, atypical enamel thickness, estimated body height of about 3 m and mass of 200–300 kg, making it the largest primate ever to have existed on Earth^4^. Despite 85 years of searching, the *G. blacki* fossil record is restricted to four mandibles and almost 2,000 isolated teeth with no postcranial evidence^4^. Its initial discovery in an apothecary shop in Hong Kong as a ‘Dragon tooth’^1^ initiated a search for the first in situ finds^10^ (Extended Data Fig. 1f) and culminated in the discovery of several cave sites in two main areas, Chongzuo and Bubing Basin, in the Guangxi ZAR province^4^. These sites contain crucial evidence for its survival and eventual demise.

Very few of these *G. blacki* sites have been dated using more than one radiometric technique; thus the timing of extinction remains uncertain^11^. The current timeline for its presence is 2.2 Ma (Baikong cave^12^) to 420–330 ka (Hejiang Cave^9^). During this time, *G. blacki* underwent morphological changes including an increase in tooth size^13^ and dental complexity^9^, seemingly indicating a dietary change in response to ecological pressure^13^. Reconstructions of *G. blacki* diet based on the dental anatomy indicate a specialized herbivore with adaptations for the consumption of abrasive food^14,15^, heavy mastication of fibrous food^16,17^ and a fruit-rich diet^6,18^. The diverse forest ecosystem at the time of Baikong had the capacity to support the biomass of several primate communities^4^ over a wide area from Guangxi, Guizhou, Hainan and Hubei Provinces^19^. However, by the time of Hejiang, *G. blacki* had a dramatic range reduction to just Guangxi^9,13^. The reasons for this dramatic reduction and eventual extinction remain hotly disputed^4^ because of a lack of a regional approach, a focus on single sites and methods and an absence of behavioural^4^ and environmental evidence^20^.

**Extended Data Fig. 2:**
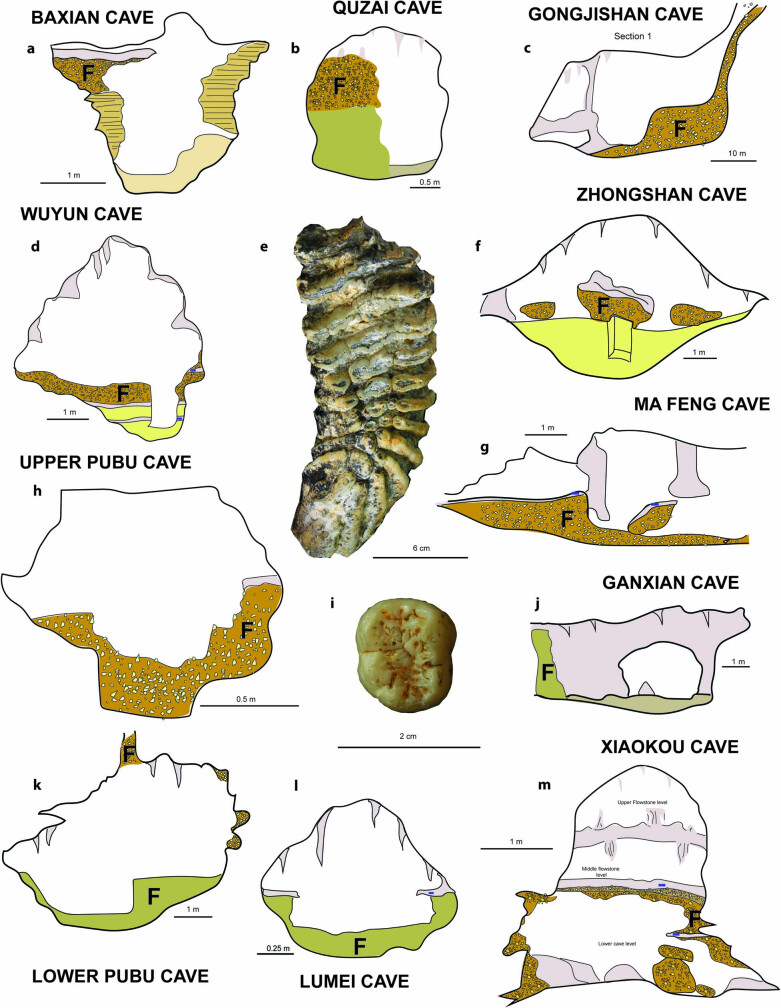
The non-*G.blacki*-bearing caves in Chongzuo and Bubing Basin. **a**, Baxian; **b**, Quzai; **c**, Gongjishan; **d**, Wuyun; **e**, typical fauna from Baxian Cave – *Elephas maximus*, left M3 (CFLSY201104-2-1); **f,** Zhongshan; **g**, Ma Feng; **h**, Upper Pubu; **i**, typical fauna from Baxian Cave – *Pongo weidenreichi*, left M2, (CFLSY201011-23-871); **j,** Ganxian; **k**, Lower Pubu; **l**, Lumei; **m**, Xiao Kou. All profiles display the main stratigraphic relationships and the units containing fossil evidence but no evidence of *G. blacki*. See the individual cave plans, profiles and sampling locations plus the keys for the symbols in Fig. S1a–v.

To identify the potential causes of *G. blacki* extinction, we applied a regional approach to 22 caves in Chongzuo and Bubing Basin that contained either *G. blacki*-bearing (11) or non-*G. blacki*-bearing (11) cave deposits (Extended Data Figs. 1 and 2 and Supplementary Information sections 2 and 3). Using a combination of previous excavations (1999–2016) and newly discovered caves (2017–2020) we identified and sampled fossil breccias for dating, palaeoclimate proxies and behavioural analyses. We applied six independent dating techniques to the sediments (post-infrared-infrared stimulated luminescence (pIR-IRSL), optically stimulated luminescence (OSL), electron spin resonance (ESR) on quartz and U-series on speleothem) and fossils (U-series on teeth, coupled US-ESR) to determine a Bayesian modelled age range for each site (Supplementary Information sections 4–8), which were then further modelled to provide a regional extinction window (EW). We applied pollen, charcoal, palaeontological, stable isotope and microstratigraphical analyses to the sediments and fossils to reconstruct the past environments (Supplementary Information sections 3 and 10–12). Finally, we applied trace element, stable isotope and dental microwear textural analysis (DMTA) to the *G. blacki* and closest relative *Pongo weidenreichi* teeth to determine any changes in the diet and behaviour of *G. blacki* before and within the EW that may have related to its demise (Supplementary Information sections 12–14).

**Fig. 2:**
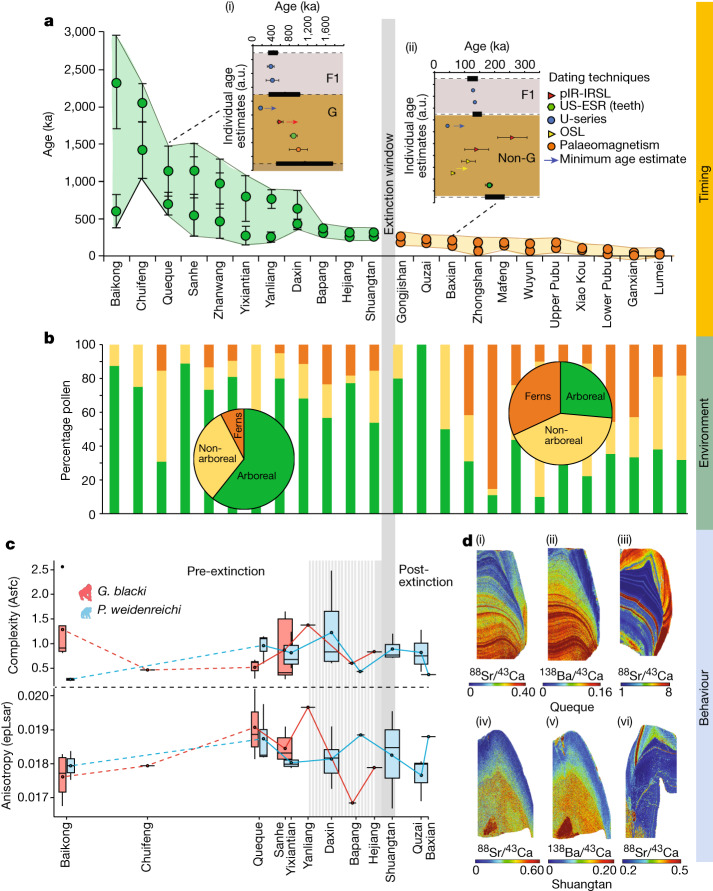
Example datasets to support the extinction events. **a**–**d**, Data relate to timing (**a**), environment (**b**) and behaviour (**c**,**d**) presented by sites. **a**, Modelled age ranges of each cave (*n* = 22 caves) using the minimum and maximum age of the fossil-bearing unit (*n* = 157 samples). The caves (*x* axis) versus age (*y* axis), with *G. blacki* (green circles) and non-*G. blacki* (orange circles) breccia. The data points represent mean ages with s.d. at 2*σ* uncertainties. The insets are modelled breccia from Queque (i) and Baxian (ii). G, *G. blacki*-bearing breccia; F1, overlying flowstone; and Non-G, absence of *G. blacki*. Data points are mean ages with s.d. at 2*σ* uncertainties. The black horizontal rectangles (with dashed lines) represent the boundary according to the modelling (Supplementary Information section 2 and Supplementary Fig. S1a–v). The modelled EW is the vertical grey line. **b**, Percentage pollen from the sites in **a** representing arboreal (green), non-arboreal (yellow) and ferns (orange). The pie charts provide an average of pollen changes for pre- (left) and post-extinction (right). **c**, DMTA boxplot series according to age of 12 caves (*x* axis) versus molar microwear complexity (Asfc, top, *y* axis) and anisotropy (epLsar, bottom, *y* axis) of *G. blacki* (red, *n* = 16) and *P. weidenreichi* (blue, *n* = 22). The boxplots size ranges represent mean complexity and anisotropy values per site. Data are presented as mean values ± interquartile range and whiskers at 95% CI (Supplementary Table S28). **d**, Trace elemental mapping of *G. blacki* and *P. weidenreichi*. Sr/Ca (i) and Ba/Ca (ii) of a right M3 *G. blacki* tooth (CSQSN-44) and Sr/Ca map from a right M2 *P. weidenreichi* tooth (CSQ0811-4) (iii) all from Queque Cave. Below, Sr/Ca (iv) and Ba/Ca (v) from a P4 tooth of *G. blacki* (ST_02_109) compared to Sr/Ca (vi) from a left M3 tooth of *P. weidenreichi* (CLMST0911-118) all from Shuangtan Cave. a.u., arbitrary units. Source Data

**Fig. 3:**
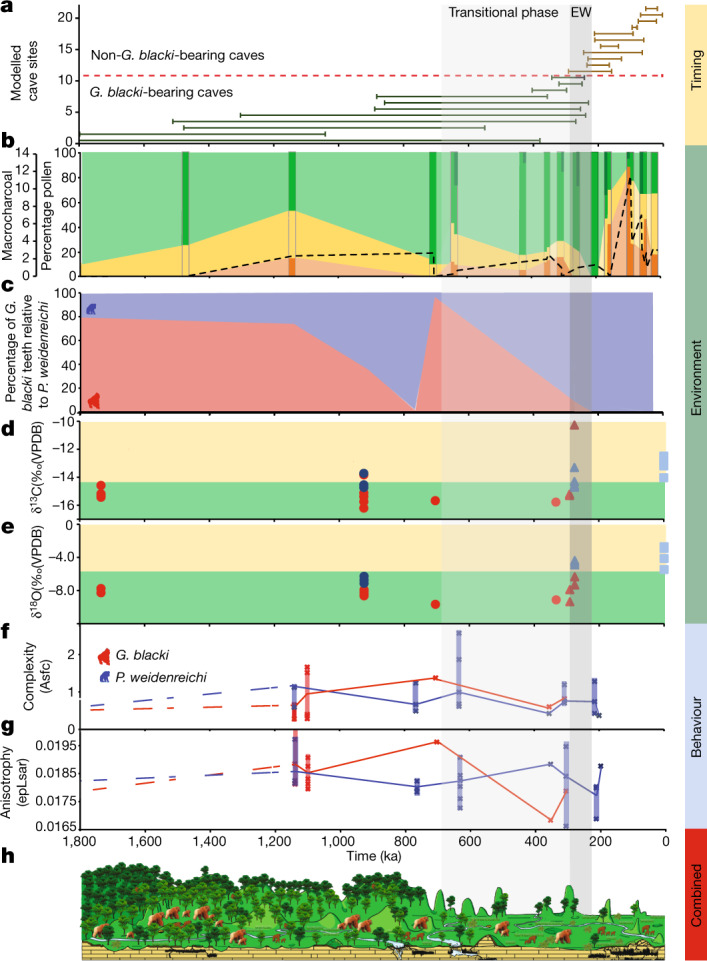
A summary of all datasets plotted against time. **a**, Timeline for extinction based on the modelled age ranges for all 22 caves. The numbers on the *y* axis relate to the caves in Fig. 2a. Note the reduced timeline (1,800 ka). The EW (255 ± 40 ka) is a vertical grey box (EW) with a solid lighter grey box (transitional phase) for the start of increased environmental variability. **b**, The percentage pollen plotted on a timeline grouped into arboreal (green), non-arboreal (yellow) and ferns (orange). The darker strips represent sites that contain pollen data, whereas the lighter sections in between represent an estimation of pollen changes. The microcharcoal (black dashed line) correlates with the increase in ferns and decline in arboreal cover. The dark green arboreal sections represent forest disturbance/high turnover taxa such as *Trema*, *Celtis* and *Sapindaceae* are present during the transitional phase and EW. **c**, The percentage of *G. blacki* teeth (red) relative to *P. weidenreichi* teeth (blue) at representative caves as a rough proxy for the relative abundance of *G. blacki* in comparison to *P. weidenreichi* in each site. The relative number of *G. blacki* teeth declines just before the transitional phase representing a change in faunal composition and during the transitional phase representing the extirpation of *G. blacki*. **d**,**e**, Isotopic changes for fossil *P. weidenreichi* (blue circles and triangles) and *G blacki* (red circles and triangles) teeth plotted on a timeline; modern *P. weidenreichi* are blue squares. δ^13^C (‰) (**d**) and δ^18^O(‰) (**e**). **f**,**g**, DMTA boxplot time-series for microwear complexity (**f**) and anisotropy (**g**) of *G. blacki* (red) and *P. weidenreichi* (blue); see Fig. 2c for definitions. **h**, A landscape and environment timeslice demonstrating the change in vegetation and primate species from the pre-EW, through the EW to the post-EW. Source Data

**Extended Data Fig. 3:**
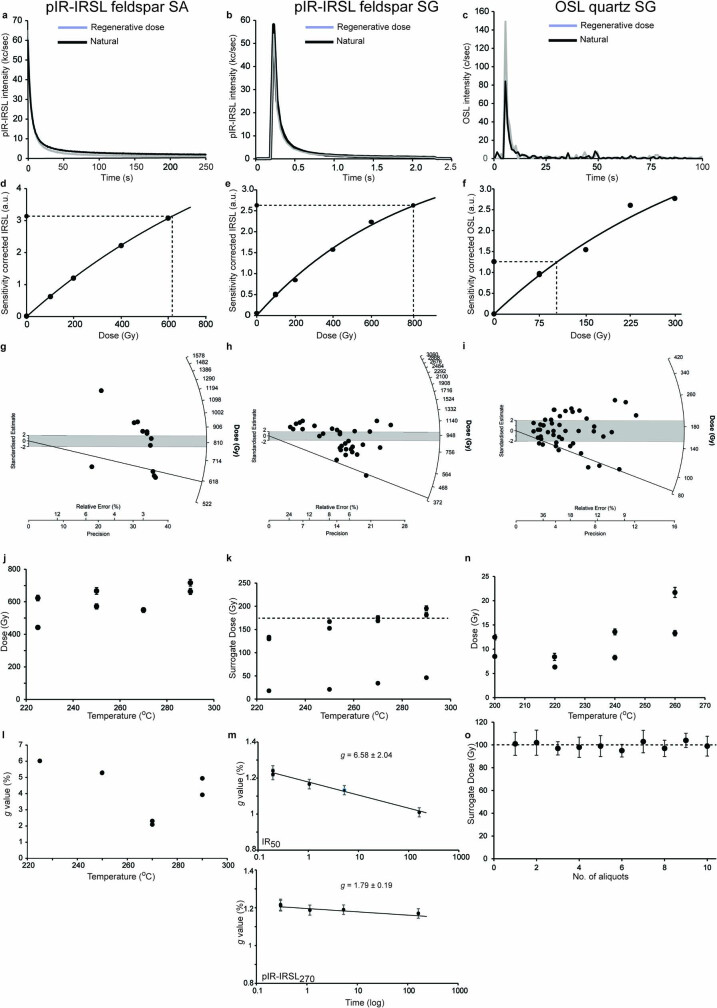
The luminescence characteristics of the quartz and feldspar sampled from caves in the Guangxi region of southern China. The resulting data from the application of three luminescence dating techniques; pIR-IRSL SA feldspars, pIR-IRSL SG feldspars and OSL SG quartz to typical samples from three different caves have been displayed. **a**, **d**, **g**, pIR-IRSL data for a single-aliquot from sample HEJ1 from Hejiang cave (a *G. blacki* bearing cave): **a**, a shine down curve including both the natural and regenerative dose; **d**, the dose response of the sample aliquot with a D_e_ of 631 ± 18 Gy, a recycling ratio of 1.04 ± 0.03 and a D0 of 1136 ± 139 Gy; **g**, the corresponding radial plot for this sample with an uncorrected MAM of 615 ± 11 Gy (n = 12 aliquots). **b**, **e**, **h**, pIR-IRSL single-grain data for sample GONG-16 from Gongjishan cave (a non-*G. blacki* bearing cave): **b**, single-grain shine down including both the natural and regenerative dose; **e**, the dose response of the sample aliquot with a D_e_ of 801 ± 51 Gy, a recycling ratio of 1.06 ± 0.05 and a D0 of 787 ± 124 Gy; **h**, the corresponding radial plot for this sample with an uncorrected MAM of 388 ± 23 Gy (n = 32 grains). **c**, **f**, **i**, OSL quartz single-grain data for sample CLPB-5 from Lower Pubu cave (a non-Giganto bearing cave): **c**, a single-grain shine down curve including both the natural and regenerative dose; **f, ** the dose response of the sample aliquot with a D_e_ of 108 ± 14 Gy, a recycling ratio of 1.03 ± 0.17 and a D0 of 378 ± 188 Gy; **i**, the corresponding radial plot for this sample with a MAM of 64 ± 7 Gy (n = 45 grains). **j**-**m**, Preheat, dose recovery, bleaching and fading tests for the pIR signal at the four preheat and pIR-IRSL stimulation temperature combinations (outlined in the supp) using sample CSH1 (n = 8). **j**, A preheat plateau test with the 270 °C pIR-IRSL stimulation and preheat temperature of 300 °C producing the least scatter between aliquots (n = 12). **k**, A dose recovery test with a surrogate dose of 175 Gy, with the 270/300 °C combination recovering the dose within error margins, also shown are the residual values (in Gy) after a 4 hours bleach using a solar simulator (n = 8). **l**, The difference in *g* values as a measure of fading at the 4 different combinations. **m**, An example of the difference in fading between the IR_50_ measurement and pIR_270_ measurement (n = 8). **n**, **o**, procedural tests for the OSL signal conducted on small aliquots: **n**, preheat plateau test using 2 aliquots per temperature point (n = 8), 220 °C was the chosen temperature; **o**, a dose recovery test using a surrogate dose of 100 Gy (n = 10) and the chosen preheat of 220 °C on 10 small aliquots – all aliquots returned the surrogate dose within standard errors. All data points from j-o are mean values with s.d. at 1σ uncertainties.

**Extended Data Fig. 4:**
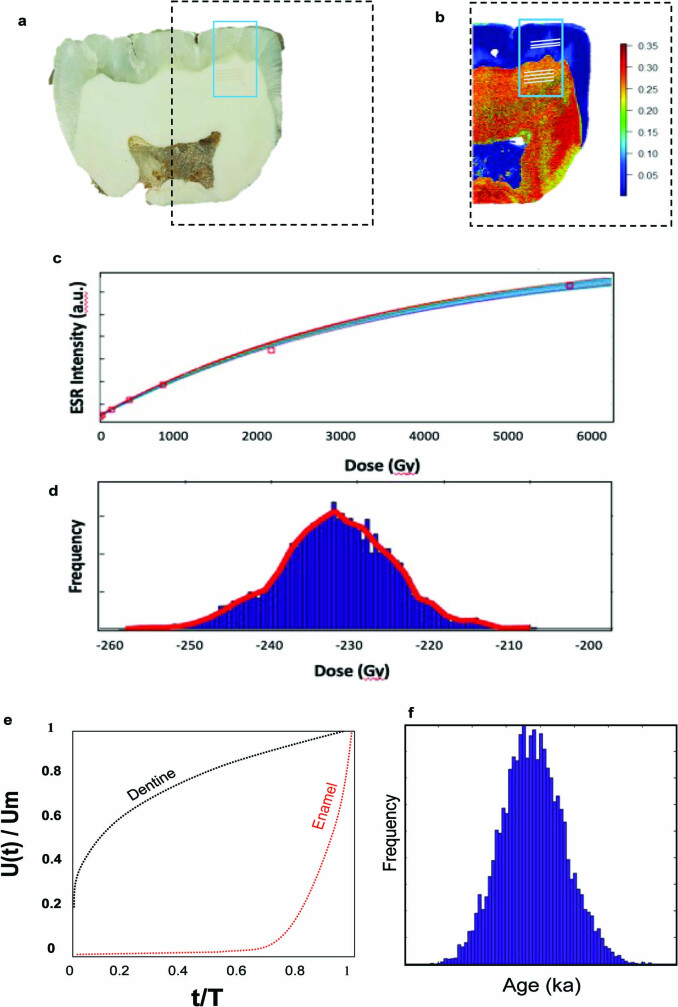
Coupled US-ESR dating protocol. **a**, Section of *Gigantopithecus blacki* tooth (Baikong 1096), with dotted black rectangle describes the area analysed for uranium distribution, while the blue rectangle highlights the uranium series rasters position. Uranium rasters are placed parallel to each other with a 200 micron spacing to avoid contaminations. Surface prior to analyses are cleaned with a preablation run at high-speed (200microns/s) and lower energy (~10% of ablation energy). **b**, LA-ICP-MS elemental mapping of the uranium distribution and diffusion pattern into the dental tissues of Baikong 1096; the pattern observed is typical of a diagenetic diffusion signal with heterogenous distribution. **c**, Dose Response Curve (DRC) modelling using Monte Carlo algorithm (Baxian RTK201306-77). **d**, Dose equivalent distribution frequency of Baxian RTK201306-77 according to the DRC simulation, using the McDoseE 2.0 software from ref. ^69^. **e**, Model of uranium diffusion over time for both dentine and enamel of Zhanwang O1Z1 sample. **f**, Coupled US-ESR Age distribution frequency using^70^ programme of Zhanwang O1Z1 sample.

**Extended Data Fig. 5:**
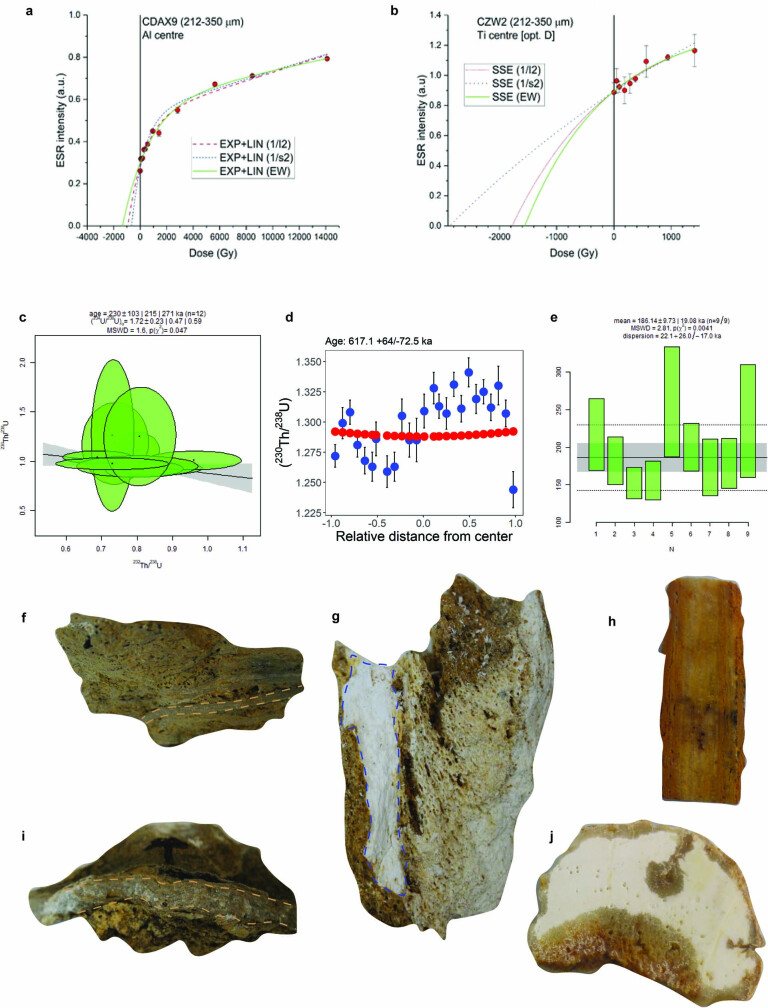
ESR quartz and U-series on carbonates and bone. **a**, ESR dose response curve for CDAX9 (212-350 µm) obtained from the measurement of the Al centre. Experimental data points represent mean ESR intensities and associated 1 standard deviation (vertical error bars), derived from the repeated measurements (n = 3; see Table S18). **b**, ESR dose response curve for CZW2 (212-350 µm) obtained from the measurement of the Ti centre. Experimental data points represent mean ESR intensities and associated 1 standard deviation (vertical error bars) derived from the repeated measurements (n = 3; see Table S19). **c**, U-series series isochron plot for sample WUY-F1 providing an age estimate of 230 ± 103 ka. **d**, Diffusion absorption density (DAD) modelling^197^ of the U-series ages for a bone from Yanliang Cave (image in **g**) providing an age estimate of 617 + 64/-73 ka, n = 10, with each point representing the mean age with s.d. at 2 σ uncertainties. **e**, A weighted mean plot of U-series ages for sample CMF-F3 providing an age estimate of 186 ± 10 ka. **f**, A flowstone sample from Shuangtan Cave (SHTC-F1C) with the purest layer depicted by a brown dashed line providing an age estimate of 330 ± 33 ka. **g**, A bone encased in breccia deposits sampled from Yanliang Cave (data in **b**). **h**, A flowstone sample from Ma Feng cave (CMF-F3) providing an age estimate of 186 ± 9 ka. **i**, A flowstone sample from Baxian cave (BAX-F2a) with the purest layer depicted by a brown dashed line providing an age estimate of 128 ± 3 ka. **j**, The bone sampled from Yanliang (in g) after being cleaned and cut prior to sampling.

**Extended Data Fig. 6:**
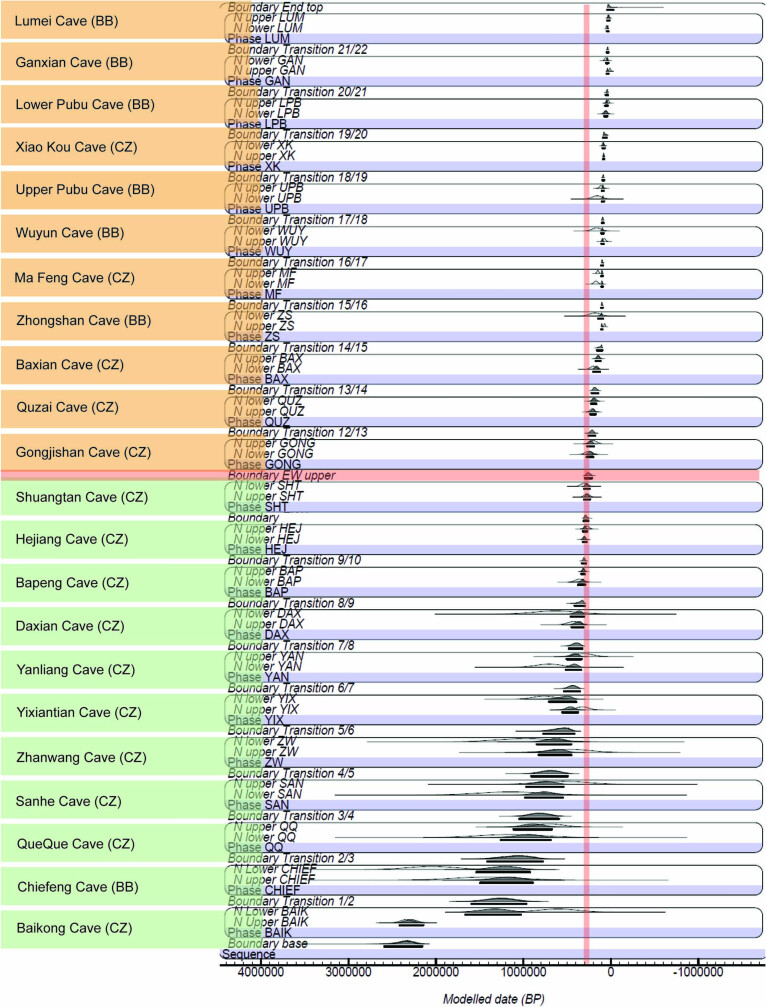
Modelling of all 22 sites to establish the timing of the Extinction Window. The master Bayesian plot composed of the modelled age ranges for all 22 caves. The names of the caves have been included on the left – colour coded to signify the *G. blacki*-bearing caves (green) and the non-*G. blacki*-bearing caves (brown). Following each cave name its location is signified in brackets with either CZ – Chongzuo area or BB – Bubing Basin. See Fig. 1 for the locations of these areas. The master model has been presented at 2 σ error margin with the age range of each cave representing the upper and lower boundary of the fossil-bearing layer. The extinction window of *G. blacki* lies in the boundary between Shuangtan and Gongjisgan Caves (red horizontal line) and has been modelled between 295–215,000 yrs ago (255 + − 40 ka) (red vertical line).

According to the 157 radiometric age estimates, the fossil evidence in the 22 caves ranges from 2,300 to 49 ka (Figs. 2a and 3a, Extended Data Figs. 3–6 and Supplementary Information sections 4–8 for all dating tables and discussion of limitations and uncertainties). This study expands the timeline for the presence of *G. blacki* from 2.3 Ma to 255 ka, provides a precise timing for the window of extinction at 295–215 ka (2*σ*) (Supplementary Information section 9) and establishes focus points for the palaeoenvironmental and behavioural analysis (pre-EW (2,300–700 ka), transitional phase (700–295 ka), EW (295–215 ka) and post-EW (215 ka to the present)).

**Extended Data Fig. 7:**
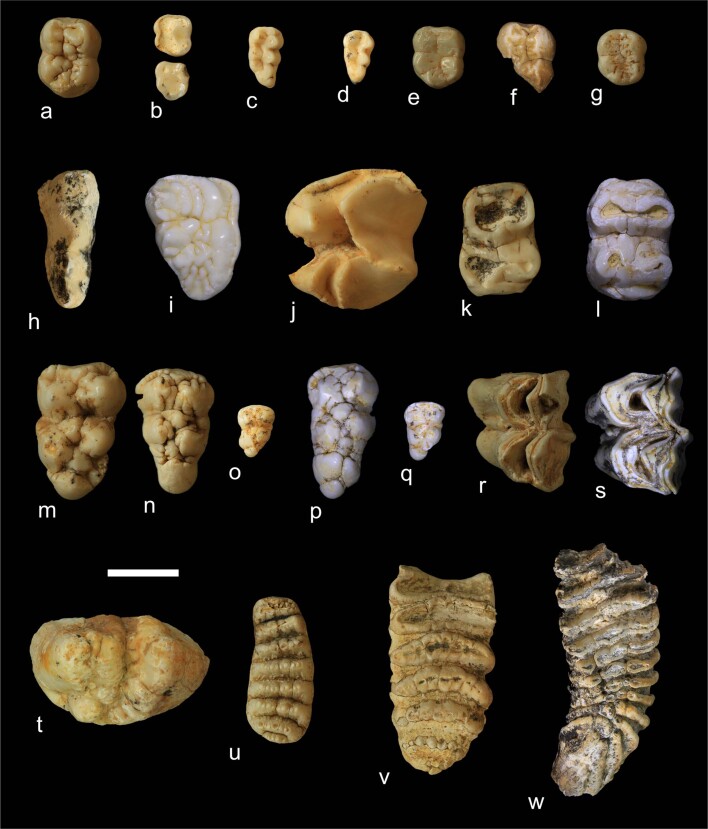
Typical fossils of the selected Chongzuo Pleistocene faunas. **Baikong**: **a**, *Gigantopithecus blacki*, left M2, CLBBD201011-31-MT33; **b**, Mystery ape, left M2-3, CLBBD201011-32-867, 932; **c**, Procynocephalus cf. wimani, right M3, CLBBD201011-32-976; **d**, Macaca sp., right M3, CLBBD201011-32-977; **e**, *Pongo weidenreichi*, left M2, CLBBD201011-32-875; *H. Pachycrocuta brevirostris licenti*, left P4, CLBBD201011-24-2026; **j**, Hesperotherium cf. sinense, left M2, CLBBD201011-7-523; **k**, Tapirus cf. sanyuanensis, left M1, CLBBD201011-27-2442; **m**, Hippopotamodon ultimus, left M3, CLBBD201011−1-31; **n**, Sus peii, right M3, CLBBD201011-1-62; **o**, Sus xiaozhu, left M3, CLBBD201011-27-2442; **r**, Megalovis guangxiensis, left M2, CLBBD201011-19-1808; **t**, Sinomastodon yangziensis, molar fragment, CLBBD201011-23-1913; **u**, Stegodon huananensis, right M3, CLBBD201011-22-1903.**Yixiantian**: **f**, *Gigantopithecus blacki*, right M2, CFLSY201104-2-17; **g**, *Pongo weidenreichi*, left M2, CFLSY201011-23-871; **i**, Ailuropoda baconi, left M2, CFLSY201011-20-2166; **l**, Megatapirus augustus, left M1, CFLSY201104-2-3; **p**, Sus peii, left M3, CFLSY201011-1-9; **q**, Sus xiaozhu, left M3, CFLSY201011-6-385; **s**, Megalovis guangxiensis, left M2, CFLSY201011-9-667; **v**, Stegodon orientalis, left dM4, CFLSY201011-22-2278; **w**, Elephas maximus, left M3 CFLSY201104-2-1. Scale bar = 2 cm for **a**–**t**, 6 cm for **u**–**w**.

Our pollen analysis indicates that during the pre-EW the environment was dominated by arboreal species (*Pinaceae*, *Fagaceae* and *Betulaceae*) with patches of grassland (Figs. 2b and 3b). However, before the EW during the transitional phase there was a change in forest plant communities and an increase in forest disturbance taxa with more open forests dominating. Post-EW about 200 ka, there was a large decrease in arboreal cover, an increase in ferns (for example,* Moraceae* and *Podocarpus*), a large increase in grassland (for example,* Poaceae*) and increased evidence of charcoal in the landscape (Extended Data Fig. 7 and Supplementary Information section 10).

**Extended Data Fig. 8:**
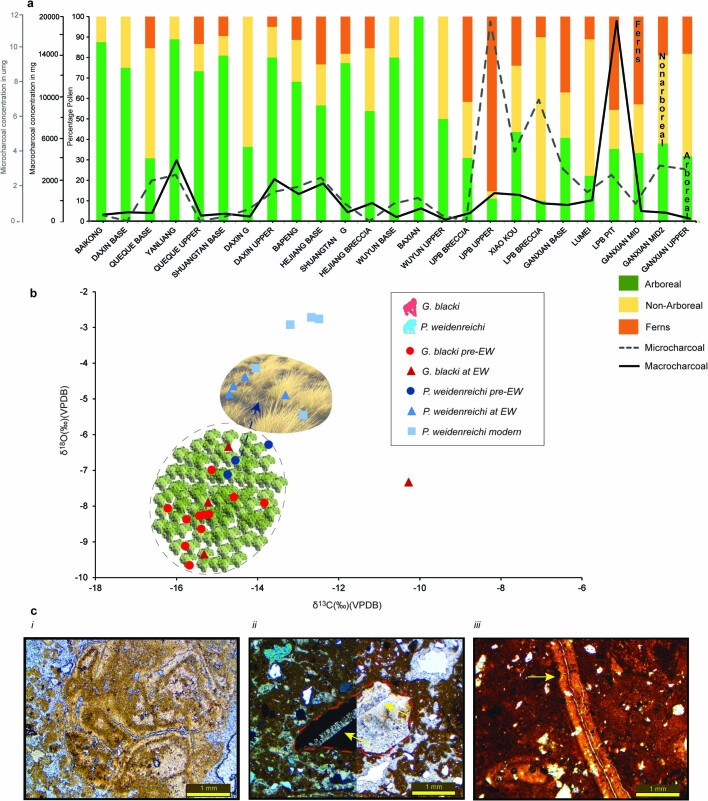
Palaeoenvironmental proxies used in this study. **a**, Pollen and charcoal analysis plotted as percentage pollen divided into arboreal, non-arboreal and ferns. Micro (grey dashed line) and macrocharcoal (black solid line) have been plotted over the top to aid comparison. **b**, Stable isotope analysis of fossil and modern *P. weidenreichi* and *G. blacki* teeth. The data points have been divided into arboreal dominated and more open grasslands dominated isotopic values and display a shift in ecological preference by *P. weidenreichi* but not *G. blacki*. **c**, A selection of micrographs from the pre-EW (Queque Cave), EW (Shuangtan Cave) and post-EW (Mafeng Cave). ***i***: Weathered coprolite in calcite rich-clay matrix, indicating faunal habitation at Queque Cave during stable, lower energetic, environmental conditions (2.5×, PPL). We used the point-counting method to assess the relative abundance of a particular feature and test for reproducibility between point-counts. Micrograph from thin section CQQ-A. ***ii***: Phosphatized bone fragment with calcite inclusion (arrows) in a sandy matrix at Shuangtan Cave, indicating unstable, higher energetic, environmental conditions (2.5×, XPL left and PPL right). Micrograph from thin section CSHT-A. ***iii***: Lighter coloured sandy-silty banded remnant feature with (Fe, Ti and/or Al) oxide stains in clayish matrix at Mafeng Cave, indicating a recovery from unstable, higher energetic to stable, lower energetic, environmental conditions (2.5×, PPL). Micrograph from thin section CMF-B.

Detailed faunal analysis indicates that the pre-EW sites were characterized by *G. blacki* (in relatively large numbers) (Fig. 3c), *Ailuropoda microta, Procynocephalus, Sinomastodon, Stegodon, Hesperotherium* and *Hippopotamodon*, which shifted to *G. blacki* (in relatively small numbers) (Fig. 3c), *Ailuropoda baconi, Stegodon* and *Elephas* before the EW and an absence of *G. blacki* post-EW (Supplementary Information section 3). The microstratigraphic analyses of five caves show pre-EW microfacies dominated by fine grains, higher clays and oxides, bioturbation and guano-induced phosphatization. At the EW, grain sizes increased, with lower oxides, bioturbation and bone/tooth alteration enabling better fossil preservation. During the post-EW, this reverted back to pre-EW features (Extended Data Fig. 8c and Supplementary Information section 11).

The stable isotope data indicate that for the pre-EW period the δ^13^C and δ^18^O of *G. blacki* range between −16.2 to −13.8‰ and −9.7 to −7.0‰, respectively. During during the EW, this increases slightly to −15.3 to −10.3‰ and −9.3 to −6.3‰, respectively. In the case of *P. weidenreichi*, the pre-EW δ^13^C and δ^18^O ranges are similar at −14.7 to −13.7‰ and −7.1 to −6.3‰, extending to −14.7 to −13.3‰ and changing to −4.9 and −4.4‰ during the EW period (Fig. 3d,e, Extended Data Fig. 8b and Supplementary Information section 12).

**Extended Data Fig. 9:**
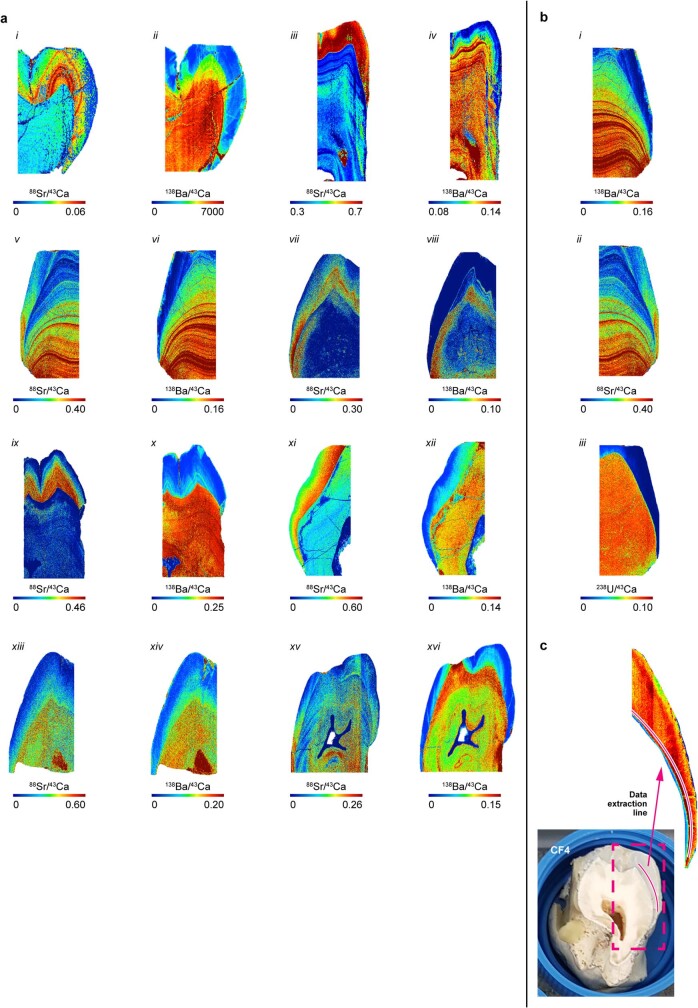
A selection of strontium and barium elemental maps. Produced from *Gigantopithecus blacki*. Fossil teeth from various cave sites. **a**, *i-ii*: Chuifeng 1; *iii-iv* Chuifeng 2; v-vi Queque 44; *vii-viii*: Queque 5; *xi-xii*: Shuangtan 50; *xiii-xiv*: Shuangtan 109; *xiv-xvi*: Hejiang 1573.8. **b**, Uranium as a marker of diagenesis. Queque 44 shows the expected distribution of uranium, mirroring the barium and strontium. **c**, Data extraction location of each sample, taken along the enamel, parallel to the enamel-dentine junction.

**Extended Data Fig. 10:**
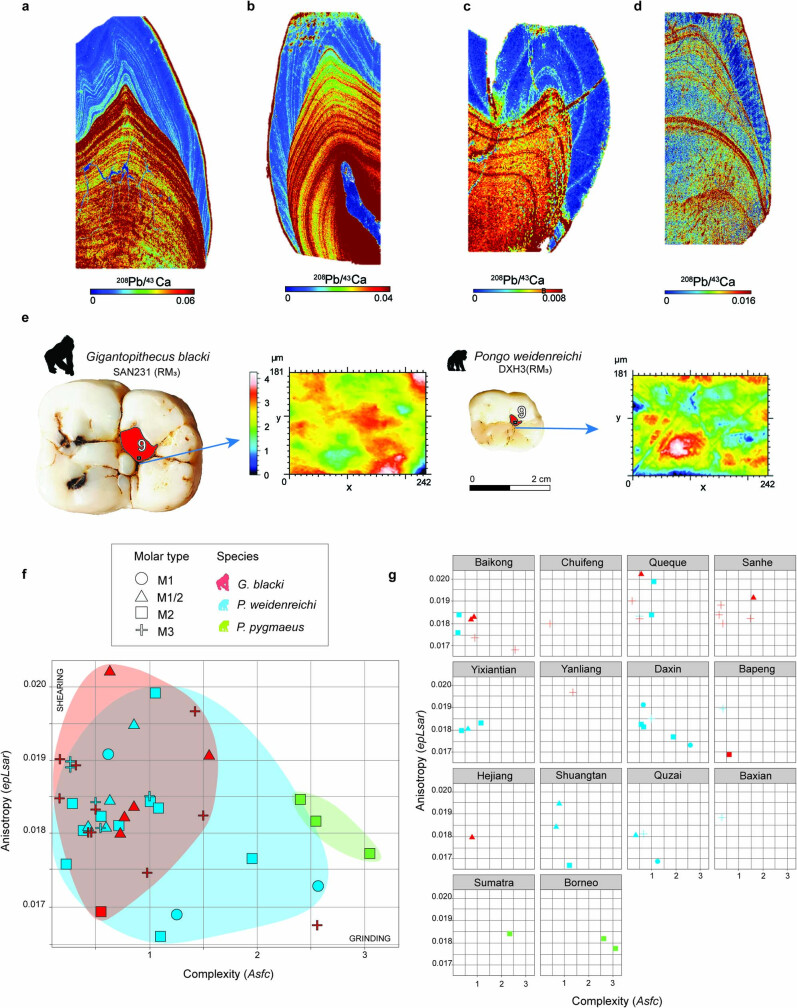
Behavioural proxies used in this study. **a**, A ^208^Pb/^43^Ca elemental map of a left M3 *G. blacki* tooth (CSQ0811-5) from the early site of Queque showing distinct lead banding. **b**, The same scan of a male right P3 (CSQSN-31) also showing the distinct banding. **c**, Teeth from Shuangtan and **d**, Hejiang, showing the banding has lessened and become more blurred. **e**, Lower right M_3_ of *G. blacki* (left, SAN231) and *P. weidenreichi* (right, DXH3) teeth showing facet 9 (red shade), close the tip crushing point. 2D occlusal maps of the scanned areas are indicated by the blue arrows. **f**, Scatterplots with DMTA results for complexity (Asfc, x axis) versus anisotropy (epLsar, y axis). Symbols indicate the molar type and colour shades represent the three different species (*G. blacki, P. weidenreichi* and *P. pygmaeus*). All individual molars (n = 41) are plotted to visualize the dietary differences at species level. **g**, Individual molars are plotted per site (n = 14 of which 12 from China), following the geochronology from Early Pleistocene (top left) to Holocene (bottom right). Data visualization was performed in R Studio 1.4.1717.

The trace element analysis of the pre-EW *G. blacki* teeth shows several, distinct and synchronous Sr/Ca and Ba/Ca bandings in the enamel and dentine that change to significantly less visible diffuse banding closer to the EW (Fig. 2d, Extended Data Figs. 9 and 10a and Supplementary Information section 13). In addition, distinct lead banding can be seen in the pre-EW, which becomes less distinct during the EW (Extended Data Fig. 10a). The microwear analysis reveals no statistically significant dietary differences between *G. blacki*- and *P. weidenreichi*-bearing sites (Supplementary Information section 14). There are, however, significant dietary differences in four *G. blacki*-bearing sites between the pre-EW and just before the EW. *G. blacki* tends to show slightly higher fluctuations in mean anisotropy and complexity trend lines, whereas those of *P. weidenreichi* seem more stable, especially for anisotropy over and beyond the EW (Figs. 2c and 3f,g, Extended Data Fig. 10b and Supplementary Information section 14).

For the first time, the largest collection of in situ evidence of *G. blacki* spanning its entire range has been robustly dated to provide a precise timeline for the presence and absence of *G. blacki* from the fossil record. Previous dating has mostly focused on the earlier *G. blacki* evidence^2,8^ and site-specific chronologies (for example, ref. ^9^). In contrast, by constraining caves within the entire age range in both Chongzuo and Bubing Basin we have more accurately established a regional window of extinction at 295–215 ka.

The pollen and faunal data indicate that the early mosaic landscapes were interrupted by enhanced environmental variability (Fig. 3b) before the EW in the transitional phase as suggested by the change in forest communities and structures and post-EW as suggested by a decline in arboreal cover and an increase in ferns and grasslands associated with fire. This variability started in a stepwise manner between 1,100 and 350 ka, with dramatic increases from about 200 ka (Fig. 3b). We have interpreted this variability as shifts towards increased seasonality and drier environments, which caused a shift to seasonal subtropical/tropical moist lowland forests and an increase in shrubs and open grassland environments before and during the EW (Supplementary Information section 10). This environmental variability is also seen in the sedimentary record as the stable low-energy environments of pre-EW were replaced by unstable high-energy environments of the EW with water availability restricted to the wet seasons (Extended Data Fig. 8c and Supplementary Information section 11).

The decline in forest cover during this period is documented in China^21^, Southeast Asia^22^ and Australasia^23^. However, our pollen study demonstrates that the key to *G. blacki* extinction is not the deterioration in arboreal cover but rather the influence of environmental variability in changing the composition of forest communities, particularly the increase in disturbance taxa. Our stable isotope and trace element data provide new insights into the extent of this variability and the impacts on *G. blacki* (Supplementary Information sections 12 and 13). Pre-EW, *G. blacki* and *P. weidenreichi* both lived in closed canopy forested environments (Fig. 3b and Extended Data Fig. 8b), with stronger biogenic banding (Fig. 2d(i)–(iii)), probably reflecting a larger diversity of food sources, including seasonal fruits and flowers and periodic water consumption, as indicated by the clear lead banding (Extended Data Fig. 10a,b). The most likely food sources would have been in greater availability all year round causing only discrete stress in the population (Fig. 2d(i)–(iii)). With the exception of one individual, throughout the EW period *G. blacki* seems to have maintained a more specialized closed canopy niche, reliant on perhaps a mixture of forest plants (Extended Data Fig. 8b). This specialization during an environmental shift may have caused a more diffused biogenic signal in individuals’ dental tissue (Fig. 2d(iv)–(v)), thus suggesting a greatly reduced dietary diversity, less regular water consumption (Extended Data Fig. 10c,d) and increased chronic stress in the population (Fig. 2d(iv)–(v)). This is the first insight into the behaviour of *G. blacki* as a species on the brink of extinction, which is in stark contrast to *P*. *weidenreichi* (Fig. 2d(vi)) that shows much less stress at this time. Beyond the EW, *P. weidenreichi* seems to have shifted to exploit the more open, seasonal habitats (Extended Data Fig. 8b), perhaps continuing to exploit the seasonal masting of fruit as modern *Pongo* does in Borneo today^24^.

The changes in microwear values in *G. blacki* and *P. weidenreichi* teeth may also be linked to periods of fruit scarcity. *G. blacki* tends to show more specific dietary preferences (in both fruits and fibrous foods) indicating greater reliance on fibrous fall-back foods (Fig. 2c), such as over the EW when the climate became more seasonal and less fruits were available. This might have forced *G. blacki* to adapt its diet from higher nutritionally preferred components in lower supply to less nutritional fall-back foods in plentiful supply. The increased consumption of fibrous foods in *P. weidenreichi* over the EW may indicate a better switch to fall-back foods and an overall more flexible and balanced diet (Fig. 2c and Extended Data Fig. 10). This first DMTA analysis on the entire range of *G. blacki* material provides a unique insight into its inability to adapt and its potentially poor choice in fall-back foods.

Our study presents a precise timeline for *G. blacki* presence and extinction. During the pre-EW period, *G. blacki* flourished alongside other primates as a successful specialist (Fig. 3c), enjoying a large diversity of food in a rich evergreen-deciduous forest (Fig. 2d(i)–(ii)) and plentiful water sources (Extended Data Fig. 10a–d) within stable environmental conditions (Fig. 2b). Around 700–600 ka in the transitional phase, there was a shift towards increased seasonality causing a change in forest communities (Fig. 3b), less diversity in food sources (Fig. 2d(iii)–(iv)), unstable high-energy environments (Extended Data Fig. 8c), changes in the composition of the fauna and widespread faunal turnovers (Fig. 3c and Supplementary Information section 2), a shift towards seasonal habitats by *P. weidenreichi* (Extended Data Fig. 8b) and a shift in the dietary diversity and behaviour of *G. blacki* (Figs. 2d and 3f,g).

Despite sharing similar environments with *P. weidenreichi* pre-EW, from 600 to 300 ka there is evidence of the inability of *G. blacki* to adapt to this transitional period, which had a greater impact on its resilience to the changing ecology*.* The reliance of *G. blacki* on fruits and lower nutritious fall-back foods (Fig. 2c) created a higher-risk foraging strategy and, combined with its much larger, less mobile body size made it more vulnerable to changes in forest structures^25^ (Fig. 2c). Moreover, *G. blacki* was exclusively terrestrial, possibly with a small geographic range^20^ but periodically travelled down the valley for water consumption (Extended Data Fig. 10a–d), whereas *P. weidenreichi* was more arboreal, mobile and semisolitary collecting water in the leaf canopy. Furthermore, the unique dentognathic features^13,14^ and giant body size^4,5^ of *G. blacki* suggest a higher demand in food uptake and slower and more delayed growth pattern, which may imply a lower reproduction rate^26^. Although *G. blacki* increased in tooth size over the Pleistocene, implying an increase in body size also, *P. weidenreichi* decreased^27^ making it a more agile adaptor. *P. weidenreichi* also demonstrated a flexibility towards the open habitats (Extended Data Fig. 8b) potentially moving in smaller groups and was able to adjust its behaviour in response to the environmental variability, causing a less stressed population (Fig. 3d).

By about 300 ka there is evidence of a struggling *G. blacki* population as the number of *G. blacki* caves and teeth reduced (Fig. 3c), indicating a dwindling population. The stark change in the teeth banding of *G. blacki* indicates chronic stress in the population (Fig. 2d(iv)–(v)) and changes from its preferred dietary behaviour (Fig. 2c and Extended Data Fig. 10f,g) indicate that *G. blacki* was struggling to respond to the environmental changes on a potentially shrinking territory^20^. It would seem that its forest refugia changed its structure and became too open and disturbed for this species to sustain itself. When compared to other well-known extinction events in North America and Australia influenced by *Homo sapiens*^28–30^, there is no evidence to suggest that archaic hominins played a role in this earlier megafaunal extinction event in southern China.

Presenting a defined cause for extinction is a feat that has seldom been achieved for many extinct species as it requires a genus- and species-specific approach^28^. Although determining the exact drivers of megafaunal extirpation and extinction can be highly challenging^29,30^, our multiproxy record of *G. blacki* timing, environment and behaviour provides robust regional insights into the ecological context of this species. *G. blacki* was the ultimate specialist and, when the arboreal environments changed, its struggle to adapt sealed its fate. In comparison, the generalist *Homo* extended and diversified across Southeast Asia during this period and seemed to have flexibly exploited the new mosaic environments that posed such a problem to *G. blacki*. Overall, our dataset provides important context for the changing fortunes of different primate species in Southeast Asia, shedding new light onto the demise of the largest primate ever to have roamed the planet.

## Methods

### Speleology and excavation techniques

Caves were discovered using a combination of local knowledge, field survey, drone mapping surveys and targeted reconnaissance with a dedicated caving team. Excavation grids were set up on the basis of the shape of cave passages and distribution of fossil-containing deposits. Jackhammers were used to break blocks of fossil-breccia and the fossils were extracted using geological hammers. Fine cleaning, identification and cataloguing were conducted in the Institute of Vertebrate Paleontology and Paleoanthropology (IVPP) laboratories.

### Luminescence dating with pIR-IRSL/SG quartz

Large bulk samples of the fossil-bearing breccia were sampled in situ under subdued red-light conditions from each cave site (Supplementary Fig. S1a–v) and processed using the standard sample purification procedures for quartz and feldspar separation including a 40% and 10% wash in hydrofluoric acid for 45 and 10 min, respectively^31^. All luminescence analysis was conducted at the ‘Traps’ luminescence dating facility at Macquarie University in Sydney, Australia. Single aliquots of 90–125 µm feldspar and 180–212 µm single grains (SGs) of quartz or feldspar were processed in a Riso TL-DA-20 containing an automated DASH set up with a dual laser single-grain attachment and a blue/UV sensitive photomultiplier tube (PDM9107Q-AP-TTL-03) using either the blue filter pack (Schott BG-39 (2 mm) and Corning 7-59 (4 mm) filters for feldspar or U340s (2× Hoya U340 3.5 mm) for quartz. Feldspar equivalent doses were corrected according to the results of the anomalous fading tests (using a weighted mean fading rate of 2.0 ± 0.2% per decade)^32^ but no residual corrections were undertaken and both feldspar and quartz *D*_e_s were then run through a minimum age model^33^ to identify the population that had the most bleaching before burial.

Measurements of ^238^U, ^235^U, ^232^Th and ^40^K were estimated using Geiger-Muller multicounter beta counting and thick source alpha counting of dried and powdered sediment samples in the laboratory, combined with in situ gamma spectrometry in the field. The corresponding (dry) beta and gamma dose rates were obtained using the conversion factors of ref. ^34^ and the beta-dose attenuation factors of ref. ^35^. Cosmic-ray dose rates were estimated from published relationships^36^, making allowance for the sediment overburden at the sample locality (ranging from 3.99 to 0.20 m), the altitude (ranging from 2,000 to 176 m above sea level) and geomagnetic latitude and longitude of the sampling sites.

### U-series dating of teeth

A total of 22 *G. blacki* and 9 *P. weidenreichi* sp. fossil teeth were analysed for U-series dating. Uranium series measurements were undertaken by laser ablation combined with multicollector inductively coupled plasma mass spectrometer (MC-ICP-MS) at GARG-SCU according to protocols in ref. ^37^. Laser ablation was performed with a New Wave Research 213 nm laser and thorium (^230^Th, ^232^Th) and uranium (^234^U, ^235^U, ^238^U) isotopes were measured on a Thermo Neptune XT MC-ICP-MS. Teeth were ablated using rasters of 5 min each and were measured with standards before and after.

### Coupled US-ESR dating of teeth

Enamel fragments from each tooth dated by coupled US-ESR techniques were separated using a hand-held diamond saw following the protocol developed by ref. ^38^. Fragments were then measured at room temperature on a Freiberg MS5000 ESR X-band spectrometer at a 0.1 mT modulation amplitude, ten scans, 2 mW power, 100 G sweep and 100 KHz modulation frequency. Each fragment was irradiated, following exponentially increasing irradiation times. Sediment elemental concentrations, external beta and gamma dose rate contributions and water content were obtained from in situ measurements. The external beta-dose rates have been extrapolated from the U, Th and K contents measured on a portion of sediment subsample (about 8 g). The external gamma dose rates were determined using a portable gamma spectrometer at each site.

### ESR dating of quartz

A total of seven samples of purified quartz (previously prepared at Macquarie University) were analysed for ESR dating purpose. For a couple of them (CBAK10 and CZW2), two grain-size fractions were measured. Quartz grains were dated by means of the multiple aliquots additive dose method and following the multiple ventre approach initially proposed by ref. ^39^. In each sample, the ESR signals of both the aluminium (Al) and titanium (Ti) centres were either acquired in separate spectra using specifically optimized parameters (standard CENIEH procedure, for example, ref. ^40^) or in a single spectrum (for example, ref. ^41^). Gamma irradiations and ESR measurements were performed at the National Research Centre on Human Evolution (CENIEH), Spain, using a Gammacell-1000 and an EMXmicro 6/1Bruker X-band ESR spectrometer, respectively.

### U-series dating of carbonates

Separate subsamples were drilled from the fresh cross-section of a hand specimen of the in situ flowstone using a hand-drill. The powdered subsamples were subjected to chemical treatment and isotopic measurements by mass spectrometry^42^. U-series dating of most speleothem samples was conducted in the Radiogenic Isotope Facility (The University of Queensland) using a Nu Plasma MC-ICP-MS. Analytical procedures followed previous publications for MC-ICP-MS^43–45^. ^230^Th/^234^U ages were calculated using Isoplot EX 3.75 (ref. ^41^) and half-lives of 75,690 years (^230^Th) and 245,250 years (^234^U)^46^. Analyses were also undertaken by laser ablation MC-ICP-MS at the Wollongong Isotope Geochronology Laboratory, University of Wollongong^47^. Laser ablation was performed with a New Wave Research ArF 193 nm Excimer laser, equipped with a TV2 cell.

### Modelling

To evaluate the uncertainties of the integrated dating approach to the site (Supplementary Tables 1–4), Bayesian modelling was performed on all independent age estimates using the OxCal (v.4.4) software 52 (https://c14.arch.ox.ac.uk/oxcal.html)^48^. The analyses incorporated the probability distributions of individual ages, constraints imposed by stratigraphic relationships and the reported minimum or maximum nature of some of the individual age estimates. Each individual age was included as a Gaussian distribution (with mean and s.d. defined by the age estimate and their associated uncertainties) and the resulting age ranges for each unit were presented at 1*σ*. The code used for each site is publicly available in Zenodo (10.5281/zenodo.10077255). 

### Pollen analysis

Pollen analysis followed a modified standard methodology described by ref. ^49^, in which sediment was dispersed in Calgon (3%) treated with HCl (10%) and sieved at >125 µm, allowed to settle in HL (heavy liquid/LST-lithium heteropolytungstates) at a density of 2.01 SG and centrifuged, then acetolysis which removes cellulose and stains the pollen followed. The remaining sample was then mounted on slides with glycerol. Pollen identification was aided by the Australasian Pollen and Spore Atlas (online resource^50^) and a handbook of quaternary pollen and spores in China^51^. Macrocharcoal analysis followed the methodology outlined by ref. ^52^.

### Microstratigraphy and spectroscopy

Five intact cave blocks were sampled for the purposes of a range of synergistic microcontextualized analyses. First, a microstratigraphic study was undertaken using petrographic microscopy (for example, refs. ^53–55^). Sediment blocks were prepared at the Flinders University Microarchaeology Laboratory and ten glass thin sections (76 mm × 50 mm × 30 µm) were made by Adelaide Petrographics. Thin sections were analysed using a Leica DM2700 P (Wetzlar) polarizing microscope following the terminology of ref. ^56^. Alkalinity (pH), X-ray diffraction (XRD)^57,58^ in an Aeris Malvern Panalytical benchtop X-ray diffractometer (2018, The Netherlands) and X-ray fluorescence (XRF)^59,60^ in an Axios Malvern Panalytical WD-XRF spectrometer tests were applied to the microsampled bulk sediments subsamples at Macquarie University.

### Stable isotope analysis

A total of 27 teeth (15 fossil *G. blacki* and 7 fossil and 5 modern *P. weidenreichi* teeth) were cleaned using an air abrasion system. Enamel powder for bulk analysis was obtained using a diamond-tipped drill. All enamel powder was pretreated following established protocols^23,61^. Following reaction with 100% phosphoric acid, gases evolved from the samples were analysed for their stable carbon and oxygen isotopic measurements using a Thermo Gas Bench 2 connected to a Thermo Delta V Advantage Mass Spectrometer at the Max Planck Institute for Geoanthropology (formerly for the Science of Human History). The δ13C and δ18O values were compared against International Standards. Overall measurement precision was studied through the measurement of repeat extracts from a bovid tooth enamel standard (*n* = 30, ±0.2‰ for both δ13C and δ18O values).

### Trace element analysis of teeth

Fossil teeth were sectioned with a high-precision diamond saw and polished to more than 10 µm smoothness. Laser ablation ICP-MS was used for trace elemental mapping analyses of the samples according to the published protocol from ref. ^62^. The GARG facility at Southern Cross University uses an ESI NW213 coupled to an Agilent 7700 ICP-MS, using rastered laser beams run along the sample surface in a straight line. A laser spot size of 40 μm, a scan speed of 80 μm s^−1^, laser intensity of 80% and a total integration time of 0.50 s were used to produce data points.

### DMTA

DMTA was applied to facet 9, as close as possible to the (ante mortem) tip crushing point of moderately worn (wear stages 2 to 4; ref. ^63^) first (m1), second (m2) and third (m3) lower molars of extinct *G. blacki* (*n* = 16), extinct *P. weidenreichi* (*n* = 22) (IVPP) and extant *P. pygmaeus* (*n* = 3) (South Australian Museum). Sample size was restricted by fossil availability. Cleaning, moulding with polyvinylsiloxane and casting with epoxy resin followed standard DMTA procedures^64–68^. Scanning of 242 × 181 μm^2^ areas was conducted on a Sensofar PLμ neox confocal profiler at the Flinders University Palaeontology Microscopy facility. Axonometric digital elevation models were fabricated in SensoMAP Premium 8.2.9564 following the ‘soft filter procedure’^68^ and analysed with the embedded scale-sensitive fractal analysis module. Statistical analyses and data visualization were carried out in Minitab 19.2020.1 and R Studio 1.4.1717.

### Reporting summary

Further information on research design is available in the Nature Portfolio Reporting Summary linked to this article.

## Online content

Any methods, additional references, Nature Portfolio reporting summaries, source data, extended data, supplementary information, acknowledgements, peer review information; details of author contributions and competing interests; and statements of data and code availability are available at 10.1038/s41586-023-06900-0.

### Supplementary information

Supplementary Information Supplementary Sections 1–14 and Supplementary References – see contents pages for details.

Reporting Summary

Peer Review File

### Source data

Source Data Fig. 2

Source Data Fig. 3

## Data Availability

The data that support the findings of this study are included in the Supplementary Information. More raw data are available from publicly available Zenodo data repositories: dating 10.5281/zenodo.10080908, and environment and behaviour 10.5281/zenodo.10080973. Source data are provided with this paper.

## Code availability

Custom codes for the OxCal program used for the Bayesian modelling in this study are publicly available in Zenodo: 10.5281/zenodo.10077255. An example of this code is included in Supplementary Fig. 11.

## References

[1] Von Koenigswald GHR (1935). Eine fossile Säugetierfauna mit Simia aus Südchina. Proc. Sect. Sci. K. Ned. Akad. Wet. Amst. B.

[2] Rink WJ, Wang W, Bekken D, Jones HL (2008). Geochronology of *Ailuropoda–Stegodon* fauna and *Gigantopithecus* in Guangxi Province, southern China. Quat. Res..

[3] Jin C (2014). Chronological sequence of the Early Pleistocene *Gigantopithecus* faunas from cave sites in the Chongzuo, Zuojiang River area, South China. Quat. Int..

[4] Zhang Y, Harrison T (2017). *Gigantopithecus blacki*: a giant ape from the Pleistocene of Asia revisited. Am. J. Phys. Anthropol..

[5] Ciochon RL, Piperno DR, Thompson RG (1990). Opal phytoliths found on the teeth of the extinct ape *Gigantopithecus blacki*: implications for paleodietary studies. Proc. Natl Acad. Sci. USA.

[6] Zhao LX, Zhang LZ (2013). New fossil evidence and diet analysis of *Gigantopithecus blacki* and its distribution and extinction in South China. Quat. Int..

[7] Welker F (2019). Enamel proteome shows that *Gigantopithecus* was an early diverging pongine. Nature.

[8] Sun L (2014). Magnetochronological sequence of the Early Pleistocene *Gigantopithecus* faunas in Chongzuo, Guangxi, southern China. Quat. Int..

[9] Zhang Y, Kono RT, Jin C, Wang W, Harrison T (2014). Possible change in dental morphology in *Gigantopithecus blacki* just prior to its extinction: evidence from the upper premolar enamel–dentine junction. J. Hum. Evol..

[10] Pei WC, Woo JK (1956). New materials of *Gigantopithecus* teeth from South China. Acta Palaeontol. Sin..

[11] Shao Q (2014). ESR, U-series and paleomagnetic dating of *Gigantopithecus* fauna from Chuifeng Cave, Guangxi, southern China. Quat. Res..

[12] Takai M, Zhang Y, Kono RT, Jin C (2014). Changes in the composition of the Pleistocene primate fauna in southern China. Quat. Int..

[13] Zhang Y (2015). Evolutionary trend in dental size in *Gigantopithecus blacki* revisited. J. Hum. Evol..

[14] Olejniczak AJ (2008). Molar enamel thickness and dentine horn height in *Gigantopithecus blacki*. Am. J. Phys. Anthropol..

[15] Kono RT, Zhang Y, Jin C, Takai M, Suwa G (2014). A 3-dimensional assessment of molar enamel thickness and distribution pattern in *Gigantopithecus blacki*. Quat. Int..

[16] Dean MC, Schrenk F (2003). Enamel thickness and development in a third permanent molar of *Gigantopithecus blacki*. J. Hum. Evol..

[17] Kupczik K, Dean MC (2008). Comparative observations on the tooth root morphology of *Gigantopithecus blacki*. J. Hum. Evol..

[18] Lovell, N. C. *Patterns of Injury and Illness in Great Apes* (Smithsonian Institution Press, 1990).

[19] Jablonski NG, Whitfort MJ, Roberts-Smith N, Xu Q (2000). The influence of life history and diet on the distribution of catarrhine primates during the Pleistocene in eastern Asia. J. Hum. Evol..

[20] Cheng L (2020). Environmental fluctuation impacted the evolution of Early Pleistocene non-human primates: biomarker and geochemical evidence from Mohui Cave (Bubing, Guangxi, southern China). Quat. Int..

[21] Li SP (2020). Pleistocene vegetation in Guangxi, south China, based on palynological data from seven karst caves. Grana.

[22] Louys J, Roberts P (2020). Environmental drivers of megafauna and hominin extinction in Southeast Asia. Nature.

[23] Hocknull SA, Zhao JX, Feng YX, Webb GE (2007). Responses of middle Pleistocene rainforest vertebrates to climate change in Australia. Earth Planet. Sci. Lett..

[24] Louys J (2021). Sumatran orangutan diets in the Late Pleistocene as inferred from dental microwear texture analysis. Quat. Int..

[25] Crooks KR (2017). Quantification of habitat fragmentation reveals extinction risk in terrestrial mammals. Proc. Natl Acad. Sci. USA.

[26] Zhao L, Zhang L, Zhang F, Wu X (2011). Enamel carbon isotope evidence of diet and habitat of *Gigantopithecus blacki* and associated mammalian megafauna in the Early Pleistocene of South China. Chin. Sci. Bull..

[27] Harrison T, Zhang Y, Yang L, Yuan Z (2021). Evolutionary trend in dental size in fossil orangutans from the Pleistocene of Chongzuo, Guangxi, southern China. J. Hum. Evol..

[28] Price GJ, Louys J, Faith JT, Lorenzen E, Westaway MC (2018). Big data little help in megafauna mysteries. Nature.

[29] Rule S (2012). The aftermath of megafaunal extinction: ecosystem transformation in Pleistocene Australia. Science.

[30] Barnosky AD, Koch PL, Feranec RS, Wing SL, Shabel AB (2004). Assessing the causes of late Pleistocene extinctions on the continents. Science.

[31] Aitken, M. J. *An Introduction to Optical Dating: The Dating of Quaternary Sediments by the Use of Photon-Stimulated Luminescence* (Oxford Univ. Press, 1998).

[32] Lamothe M, Auclair M, Hamzaoui C, Huot S (2003). Towards a prediction of long-term anomalous fading of feldspar IRSL. Radiat. Meas..

[33] Galbraith RF, Roberts RG, Laslett GM, Yoshida H, Olley JM (1999). Optical dating of single and multiple grains of quartz from Jinmium rock shelter, northern Australia. Part 1, Experimental design and statistical models. Archaeometry.

[34] Guérin G, Mercier N, Adamiec G (2011). Dose rate conversion factors: update. Anc. TL.

[35] Mejdahl V (1979). Thermoluminescence dating: beta-dose attenuation in quartz grains. Archaeometry.

[36] Prescott JR, Hutton JT (1994). Cosmic ray contributions to dose rates for luminescence and ESR dating: large depths and long-term time variations. Radiat. Meas..

[37] Demeter, F. et al. A Middle Pleistocene Denisovan molar from the Annamite Chain of northern Laos. *Nat. Commun.* **13**, 2557 (2022). 10.1038/s41467-022-29923-z PMC9114389 35581187

[38] Joannes-Boyau R, Grün R, Bodin T (2010). Decomposition of the laboratory gamma irradiation component of angular ESR spectra of fossil tooth enamel fragments. Appl. Radiat. Isot..

[39] Toyoda S, Voinchet P, Falguères C, Dolo JM, Laurent M (2000). Bleaching of ESR signals by the sunlight: a laboratory experiment for establishing the ESR dating of sediments. Appl. Radiat. Isot..

[40] Duval M, Sancho C, Calle M, Guilarte V, Peña-Monné JL (2015). On the interest of using the multiple center approach in ESR dating of optically bleached quartz grains: some examples from the Early Pleistocene terraces of the Alcanadre River (Ebro basin, Spain). Quat. Geochronol..

[41] Bartz M (2020). Testing the potential of K-feldspar pIR-IRSL and quartz ESR for dating coastal alluvial fan complexes in arid environments. Quat. Int..

[42] Zhou HY, Zhao JX, Wang Q, Feng YX, Tang J (2011). Speleothem-derived Asian summer monsoon variations in Central China during 54–46 ka. J. Quat. Sci..

[43] Clark TR (2014). Discerning the timing and cause of historical mortality events in modern Porites from the Great Barrier Reef. Geochim. Cosmochim. Acta.

[44] Cheng H (2000). The half-lives of uranium-234 and thorium-230. Chem. Geol..

[45] Ludwig, K. R. *User’s Manual for Isoplot 3.75. A Geochronological Toolkit for Microsoft Excel* (Berkeley Geochronology Center, 2012).

[46] Eggins SM (2005). In situ U-series dating by laser-ablation multi-collector ICPMS: new prospects for Quaternary geochronology. Quat. Sci. Rev..

[47] Ma L, Dosseto A, Gaillardet J, Sak PB, Brantley S (2019). Quantifying weathering rind formation rates using in situ measurements of U-series isotopes with laser ablation and inductively coupled plasma-mass spectrometry. Geochim. Cosmochim. Acta.

[48] Bronk Ramsey C (1995). Radiocarbon calibration and analysis of stratigraphy: the OxCal program. Radiocarbon.

[49] Bennett, K. D. & Willis, K. J. in *Tracking Environmental Change Using Lake Sediments: Terrestrial, Algal and Siliceous Indicators* (eds Smol, J. B. et al.) 5–32 (Springer, 2001).

[50] Haberle SG (2021). A new version of the online database for pollen and spores in the Asia-Pacific region: the Australasian Pollen and Spore Atlas (APSA 2.0). Quat. Aust..

[51] Tang, L. et al. *An Illustrated Handbook of Quaternary Pollen and Spores in China* (Science Press, 2016).

[52] Whitlock, C. & Larsen, C. in *Tracking Environmental Change Using Lake Sediments: Terrestrial, Algal and Siliceous Indicators* (eds Smol, J. B. et al.) 75–97 (Springer, 2001).

[53] Goldberg P, Berna F (2010). Micromorphology and context. Quat. Int..

[54] Morley MW (2017). Initial micromorphological results from Liang Bua, Flores (Indonesia): site formation processes and hominin activities at the type locality of *Homo floresiensis*. J. Archaeolog. Sci..

[55] Morley, M. W. et al. Hominin and animal activities in the microstratigraphic record from Denisova Cave (Altai Mountains, Russia). *Sci. Rep.* **9**, 13785 (2019). 10.1038/s41598-019-49930-3 PMC6763451 31558742

[56] Stoops, G. *Guidelines for Analysis and Description of Soil and Regolith Thin Sections* (Soil Science Society of America, 2003).

[57] Moore, D. M. & Reynolds, R. C. *X-Ray Diffraction and the Identification and Analysis of Clay Minerals* 2nd edn (Oxford Univ. Press, 1997).

[58] Ohishi T, Terakawa M (2019). Characteristics of weathered mudstone with X-ray computed tomography scanning and X-ray diffraction analysis. Bull. Eng. Geol. Environ..

[59] Ryan CG (2018). Maia Mapper: high definition XRF imaging in the lab. J. Instrum..

[60] Zougrou IM (2016). Characterization of fossil remains using XRF, XPS and XAFS spectroscopies. J. Phys. Conf. Ser..

[61] Roberts, P. et al. Isotopic evidence for initial coastal colonization and subsequent diversification in the human occupation of Wallacea. *Nat. Commun.* **11**, 2068 (2020). 10.1038/s41467-020-15969-4 PMC7190613 32350284

[62] Joannes-Boyau R (2019). Elemental signatures of *Australopithecus africanus* teeth reveal seasonal dietary stress. Nature.

[63] Smith T (2018). Wintertime stress, nursing and lead exposure in Neanderthal children. Sci. Adv..

[64] Calandra I, Schulz E, Pinnow M, Krohn S, Kaiser TM (2012). Teasing apart the contributions of hard dietary items on 3D dental microtextures in primates. J. Hum. Evol..

[65] Schulz E, Calandra I, Kaiser TM (2010). Applying tribology to teeth of hoofed mammals. Scanning.

[66] Schulz E, Calandra I, Kaiser TM (2020). Tracing chewing mechanisms in hoofed mammals: 3D tribology of enamel wear. Mamm. Biol..

[67] Schulz E, Calandra I, Kaiser TM (2013). Feeding ecology and chewing mechanics in hoofed mammals: 3D tribology of enamel wear. Wear.

[68] Arman, S. D. et al. Minimizing inter-microscope variability in dental microwear texture analysis. *Surf. Topogr. Metrol. Prop*. **4**, 024007 (2016).

