# Supplementary material for: The demise of the giant ape Gigantopithecus blacki
Source: Nature. 2024 Jan 10;625(7995):535–9. doi: 10.1038/s41586-023-06900-0 (PMC10794149; doi:10.1038/s41586-023-06900-0)
Supplement: Supplementary file 1 — Supplementary Sections 1–14 and Supplementary References – see contents pages for details. [file 41586_2023_6900_MOESM1_ESM.docx]

**The demise of the giant ape *Gigantopithecus blacki***

**SUPPLEMENTARY INFORMATION**

**SI section 1: Background to *G. blacki* research_______________________________ 4**

Supplementary Discussion │ Previous research in Chongzuo and Bubing Basin

Table S1 │ Location and age of *G. blacki* cave sites in China

**SI section 2: Study area and cave sites____________________________________ 11**

Supplementary Discussion │ Background to the region

Supplementary Discussion │ Caves included in this study

Table S2 │ Location, altitude and description of the 22 caves included in this study

Fig. S1a-v│ Plan, profile, stratigraphy, fossil location and dating for all 22 caves used in this study.

**SI section 3: Fossil finding and analysis___________________________________ 40**

Supplementary Discussion │ Cave finding and excavation techniques

Supplementary Discussion │ Faunal analysis

Table S3 **│** Faunal assemblages for Baikong

Table S4 **│** Faunal assemblages for Yixiantian

Table S5 **│** Faunal assemblages for Baxian

Table S6 **│** Compositional comparisons among the Baikong, Yixiantian, Baxian and extant faunas

Table S7 **│** Habitat types of extant species and fossil genera in Guangxi Zhuang Autonomous Region

Fig. S2 **│** Compositional changes and events of extinction, migration, and faunal turnovers in the selected Chongzuo Pleistocene faunas.

Fig. S3 **│** Predominant habitats reflected by the Baikong, Yixiantian, and Baxian faunas.

**SI section 4: Luminescence dating of breccia_______________________________ 54**

Supplementary Discussion │ Discussion of Luminescence methods and ages

Table S8 │ pIR-IRSL and OSL procedures

Table S9 │ Procedural test results for single-grains of feldspar from sample CSHT1

Table S10 │ The classification of all single-grain rejections for selected samples

Table S11 │ pIR-IRSL and single-grain quartz dating of sediments from the cave sites of southern China: dose rate data, equivalent doses, and ages

Table S12│High resolution gamma spectrometry of selected samples from Southern China

Fig. S4│ pIR-IRSL single aliquot radial plots

Fig. S5│ pIR-IRSL and OSL single-grain radial plots

Fig. S6│ Comparison of luminescence ages with independent age estimates

**SI section 5: U-series dating of teeth______________________________________ 70**

Supplementary Discussion │ Discussion of U-series dating of teeth

Table S13 │ U-series DAD dating of *G. blacki* and *P. weidenreichi* teeth

**SI section 6: Coupled US-ESR dating of teeth______________________________ 78**

Supplementary Discussion │ U-series and ESR combined dating of teeth

Table S14 │ Coupled US-ESR dating table

Table S15 │ Age results for all coupled US-ESR and US dating analyses

**SI section 7: ESR dating of quartz_______________________________________ 83**

Supplementary Discussion │ Discussion of ESR method, results and ages

Table S16 │ List of the quartz samples analysed in the present study

Table S17 │ Results of the bulk XRD analyses reformed on several samples

Table S18 │ ESR data derived from the measurement of the Al centre.

Table S19 │ ESR data derived from the measurement of the Ti centre

Table S20 │ ESR age estimates and dose rate components

Fig. S7│ ESR spectra obtained from the measurement of various quartz samples

Fig. S8│ ESR spectra obtained from the measurement of various quartz samples

Fig. S9│ ESR dose response curves obtained from the measurement of the Al centre.

Fig. S10│ ESR dose response curves obtained from the measurement of the Ti centre.

**SI section 8: U-series dating of carbonates and bone___________________________ 98**

Supplementary Discussion │ Discussion of methods and results of U-series dating

Table S21 │ U-series data from flowstones sampled in the caves of Southern China using the ICP-MS

Table S22 │ U-series data from flowstones sampled in the caves of Southern China using the laser ablation ICP-MS

**SI section 9: Modelling of caves and EW______________________________________ 106**

Supplementary Discussion │ Modelling of age estimates and implications

Fig. S11 │An example of an Ox-cal Bayesian code used for Bapeng Cave chronology

Fig. S12│ An example of the modelling process for Bapeng Cave

Table S23 │ Modelled age range for the caves and EW

**SI section 10: Pollen analysis_______________________________________________ 110**

Supplementary Discussion │ Discussion of pollen analysis methods and results

Fig. S13a-b │ Pollen Diagrams for all caves

Fig. S14 │ A comparison with a pollen record from Leizhou Peninsula

**SI section 11: Microstratigraphy____________________________________________ 116**

Supplementary Discussion │ Discussion of cave microstratigraphic methods and results

Fig. S15 │ Stratigraphic location of block sampling

Fig. S16 │ Block sampling procedures and techniques

Table S24 │ Microstratigraphic results

Fig. S17a-e │Thin section micrographs

Fig. S18 │PSA-XRD-XRF data

Fig. S19 │Sand silt and clay ternary diagram for all five sites

**SI section 12: Stable isotope analysis of teeth__________________________________ 136**

Supplementary Discussion │ Discussion of stable isotopes C, O from teeth

Table S25 │ Stable isotope raw data

Fig. S20 │ Scatterplot of δ^13^C and δ^18^O data for fossil *G. blacki* and fossil and modern Pongo.

**SI section 13: Trace element analysis of teeth_________________________________ 141**

Supplementary Discussion │ Discussion of extended methods and results

Fig. S21 │ A selection of *G. blacki* teeth and their corresponding maps

**SI section 14: Dental microwear texture analysis (DMTA)______________________ 144**

Supplementary Discussion │ Background and methods

Table S26 │ Overview of lower hominid molars used in this study

Fig. S22 │DMTA methodology

Table S27 │Descriptive statistics through three MANOVAs and six One-Way ANOVAs of two standard DMTA variables

Table S28 │Twelve Tukey’s HSD and Fisher’s LSD pairwise tests comparisons of two standard DMTA variables

**References_______________________________________________________________ 157**

**SI section 1: Background to *Gigantopithecus* research**

The largest known ape, *Gigantopithecus blacki,* is an enigmatic hominid that captures the imagination. This massive ape is known from the Early to Middle Pleistocene cave deposits in southern China. Intriguingly, *Gigantopithecus* is the only hominid genus that went extinct in the Pleistocene. Why did this giant ape not survive the era? The limited fossil record for *Gigantopithecus* makes this question difficult to answer. Even with over 85 years of searching, the *Gigantopithecus* fossil record is restricted to four mandibles and almost two thousand isolated teeth. The large collection of teeth and well documented excavation sites, however, does allow for substantial research into this mysterious, gigantic ape.

a. Initial Discovery

The unusual story of *Gigantopithecus* starts in an apothecary shop in Hong Kong. Dragon bones, or dragon teeth, have been commonly used in traditional Chinese medicine for thousands of years to treat a wide range of illnesses^71,72^. Western scholars recognized these dragon bones as fossils and started purchasing them for study^73,71^. Early in his career, Ralph von Koenigswald was employed by a Munich museum which housed the Haberer Collection of these dragon bone fossils^74,71^. The Haberer Collection contained a single human upper molar which initiated the search for Peking Man. Ralph von Koenigswald was inspired to start his own collection of dragon bones. After he joined the East Indies Geological Survey in 1931, von Koenigswald began collecting fossils from Chinese apothecaries during his travels. He visited Chinese drugstores across southeast Asia including Java, Sumatra, Bali, Borneo, the Philippines, and Thailand, as well as more distant stores in San Francisco and New York. While the majority of the fossils von Koenigswald initially examined came from Pliocene assemblages, he also identified a Pleistocene assemblage which the drugstores classified as lower quality dragon teeth. In a Manila drugstore, von Koenigswald found his first fossil orangutan tooth which originally came from a drug store in Hong Kong. Von Koenigswald searched the drugstores in Hong Kong for more orangutan fossils with an unexpected result. Among the hundreds of orangutan teeth was a single worn third lower molar that was substantially larger than the orangutan teeth. Based on the size and morphology, von Koenigswald described a new genus and species, *Gigantopithecus blacki*, in honor of Davidson Black^1^. Von Koenigswald was able to obtain three more gigantic hominid teeth between 1935 and 1939, before his research was interrupted by World War II. Von Koenigswald was taken as a prisoner of war by the Japanese during the invasion of Java. Prior to his arrest, von Koenigswald entrusted his fossil collection to his wife and three friends to hide^75^. To prevent the fossils from being seized, the small collection of *Gigantopithecus* teeth were stored in a milk bottle and buried in a friend’s yard^76^. Due to health problems from his imprisonment, von Koenigswald moved to New York after the war to work with his friend Weidenreich at the American Museum of Natural History instead of continuing his research in southeast Asia^76^. From the Hong Kong drugstore records and associated fossils, von Koenigswald was able to establish that the *Gigantopithecus* fossils came from cave sites in Guangdong or Guangxi, but he was not able to conduct excavations for *in situ* fossils^71^.

b. Discovery of *in situ* fossils

Locating *in situ Gigantopithecus* fossils was an important goal of early Chinese paleontologists. Initial surveys of cave sites in southern China were conducted by the Cenozoic Laboratory of the Geological Survey of China (the predecessor of IVPP, namely Institute of Vertebrate Paleontology and Paleoanthropology, Chinese Academy of Sciences) in 1935^77,78^. These excavations identified two stratigraphic units, a Lower or Middle Pleistocene and an Upper Pleistocene. The Lower or Middle Pleistocene unit contained yellow deposits that were associated with a *Stegodon-Ailuropoda* faunal assemblage. This unit matched the drugstore *Stegodon-Ailuropoda* assemblage that contained the *Gigantopithecus* fossils. Yellow earth was attached to the drugstore fossils, and fossils had similar damage from porcupines gnawing^79,71^.

In 1955, an IVPP survey team led by Wen-Chung Pei (Wen-Zhong Pei) and Lan-Po Jia traveled to Nanning, Guangxi, to determine the source of the *Gigantopithecus* fossils. The team sorted through the records and inventory of dragon bone suppliers. They were able to find new *Gigantopithecus* teeth as well as the locations of numerous cave sites that yielded mammal fossils^80,81,82^. In 1956, the researchers followed the dragon bone inventory records and local informants to a cave site in Daxin County, Guangxi (Daxin *Gigantopithecus* Cave)^81,82^. The first *in situ* *Gigantopithecus* tooth was discovered on the first day of fieldwork in the cave by Lanpo Jia^83^. Two more *Gigantopithecus blacki* teeth were recovered from the cave.

Also in 1956, the first *Gigantopithecus* mandible to be discovered was collected by a farmer while he was digging for fertilizer in Xiaoyan Cave in Liucheng County, Guangxi (Liucheng *Gigantopithecus* Cave). Pei’s team conducted excavations in this cave between 1957 and 1963. They were able to recover two more mandibles and over a thousand isolated teeth^4^,^83^. One of the mandibles is significantly larger than the other specimens and is believed to be an adult male while the other specimens were identified as a female and a juvenile. The excavations also recovered numerous other mammalian fossils including a small, short muzzled panda, which is an ancestor to modern giant pandas.

Excavations expanded from these initial cave sites with several new sites being identified in the 1960s and 1970s. The large sample of fossils collected during this time improved understanding of *Gigantopithecus blacki* morphology, age, and provenance. In 1965, twelve *Gigantopithecus* teeth were discovered in Wuming, Guangxi^84^. These teeth were significant because they were significantly larger and younger than previous finds indicating the *Gigantopithecus blacki* increased in size over time. In 1973, von Koenigswald was invited to China where he met with Pei, Young, and Woo and was able to examine the new *Gigantopithecus* *blacki* fossils^76^. Excavations continue, and there are now at least 22 known *Gigantopithecus blacki* sites in southern China, with 13 of those located within Guangxi ZAR.

c. Biogeography

*Gigantopithecus blacki* sites are distributed across southern China between the Yangtze River and the South China Sea. Cave sites have been located in Chongqing Municipality, Guangxi ZAR, Guizhou Province, Hainan Province, and Hubei Province. However, the majority of *G. blacki* sites are located in the region of Guangxi ZAR in Southern China (Fig. 1), which seems to represent an important evolutionary and zoogeographic center for *G. black**i.*^3^. Over time their distribution changed with early *G. blacki* occupying a wider distribution including Yangtze River (e.g.,^85^), and Guizhou Plateau (e.g., ^6,86^) to the north, while later *G. blacki* retreated to the warmer and wetter southern Guangxi ZAR, and further south to Hainan possibly to escape the cold and drier northern regions^3,6^. However, there is a paucity of evidence for past environmental conditions^21^ especially for the younger sites.

Karst topography dominates this region with fossil rich caves dotting the steep-sided limestone towers. Von Koenigswald was able to associate *Gigantopithecus blacki* with the Pleistocene *Stegodon-Ailuropoda* fauna from his examination of drugstore collections^1,72^. This association has been confirmed from *in situ Gigantopithecus* fossil excavations^80,87^. Despite the *Stegodon-Ailuropoda* fauna extending into Vietnam and Thailand, there is limited evidence of *Gigantopithecus blacki* outside China^88,89,90,100^. Excavations in northern Vietnam started during the 1960s. Tham Khuyen became well known for the purported evidence of the co-occurrence of *Homo erectus* and *Gigantopithecus blacki*, but reanalysis has assigned these specimens and disproven this association^4,88 89^.

Former paleoenvironmental studies indicate that *Gigantopithecus blacki* inhabited a humid subtropical vegetated forest^6,86,101^. The fauna associated with *Gigantopithecus* contains a majority of forest-dwelling and arboreal taxa associated with subtropical to tropical forested habitats including a diverse catarrhine community^12,102^. A palynological study from the site of Queque indicates a mixed evergreen and deciduous forest dominated by birch, oak, and chinkapin trees with a diverse understory^21,103^.

Porcupines were the major taphonomy force in the creation of fossil rich deposits in southeast Asian caves and were commonly represented the in Pleistocene faunas^3,104^. Von Koenigswald first documented the presence of gnaw marks on the tooth roots in his descriptions of *Gigantopithecus*^1,71^. Porcupines drag remains into their dens where they consume the bones. They are capable of eating everything except hard enamel caps. Any *Gigantopithecus* postcranial remains that were in these caves would have been eaten by the porcupines. Despite destroying the majority of the remains, without porcupines there would be no large deposits of fossil teeth.

**Table S1: Location and ages of the known *G. blacki* cave sites in China.** The teeth refer to the number of *G. blacki* teeth found at that site with the number in brackets referring to the number of mandibles found at that site.

| **Localities Region** | **Estimated Age** | **Teeth** | **References** |
| --- | --- | --- | --- |
| Early Pleistocene Sites |  |  |  |
| Baikong Cave Chongzuo, Guangxi ZAR | ~2.0 Ma | 214 | 3,8,12 |
| Longgupo Cave Wushan, Chongqing Municipality | 1.8-1.4 Ma | 16 | 85,105 |
| Liucheng, Guangxi ZAR | 1210-940 ka | 1006 (3) | 2,106 |
| Chuifeng Cave Tiandong, Guangxi ZAR | 1.97±0.19 to 1.38±0.17 Ma | 92 | 11,107 |
| Mohui Cave Tiandong, Guangxi ZAR | 1.69±0.22 to 1.38±0.11 Ma | 16 | 108-110 |
| Late Early Pleistocene Sites |  |  |  |
| Ba’eryan Cave Bijie, Guizhou Province | Early Pleistocene | 1 | 111,112 |
| Yanliang Cave Chongzuo, Guangxi ZAR | Early Pleistocene | 22 (1) | 113 |
| Boyue Cave Chongzuo, Guangxi ZAR | ~1.8 Ma | 43 | 12 |
| Longgu Cave Jianshi, Hubei Province | 2.15-1.95 Ma | 33 | 114-116 |
| Sanhe Cave Chongzuo, Guangxi ZAR | ~1.2 Ma | 74 | 3,8,12 |
| Queque Cave Chongzuo, Guangxi ZAR | ~1.0 Ma | 22 | 3,8,12 |

| Middle Pleistocene Sites |  |  |  |  |
| --- | --- | --- | --- | --- |
| Bulalishan Wuming, Guangxi ZAR | 750-480 ka |  | 12 | 117 |
| Nomoshan Bama, Guangxi ZAR | Middle Pleistocene |  | 1 | 118 |
| Hei Cave Daxin, Guangxi ZAR | 383±20 ka |  | 3 | 82,119 |
| Hejiang Cave Chongzuo, Guangxi ZAR | 400-320 ka |  | 17 | 3,15 |
| Xinchong Cave Changjiang, Hainan Province | 600-300 ka |  | 1 | 4 |

d. Size estimates

The lack of postcranial elements makes determining the mass of *Gigantopithecus* difficult. The dental remains of *Gigantopithecus* were larger than any other known ape, so it was inferred that *Gigantopithecus* was the largest ape. The average area of the upper and lower molars of *Gigantopithecus blacki* are 57.8% and 33% larger (respectively) when compared to *Gorilla gorilla*^4^. Weidenreich estimated that *Gigantopithecus blacki* would have been twice the size of a *Gorilla,* or 338 kg using an average male gorilla body weight of 169 kg^79,120^. Unfortunately, dietary adaptations can confound body mass calculations based on tooth size. *Gigantopithecus* is a relatively megadont species, so body mass calculations based on molar size overestimate total mass. Therefore, more recent studies have reduced this estimate down to 200-300 kg ^4,121,122^. Due to its large size and phylogeny, *Gigantopithecus* was most likely ground-dwelling, fist-walking quadruped.

e. Phylogenetic position

In his initial description of *Gigantopithecus blacki*, von Koenigswald classified the new genus as a specialized ape and potential side branch of *Sivapithecu**s*^1^. Weidenreich also initially considered *Gigantopithecus* to be an ape^123^. Broom positioned *Gigantopithecus* close to the common ancestor of human and apes or directly off the *Australopithecus* group^124,125^. Then, Weidenreich revised his position and reclassified *Gigantopithecus* as an ancestor of *Homo erectus* during World War II^79^. At that point, the original fossils were hidden and von Koenigswald was a prisoner of war, so Weidenreich worked from casts of the fossils. Weidenreich incorrectly dated *Gigantopithecus* to arrive at this ancestral relationship to *Homo erectus*. After the war, von Koenigswald also revised his position and reclassified *Gigantopithecus* as a side branch to human evolution related to “*Indopithecus*” *giganteus* ^71,126^. The uncertainty about the classification of *Gigantopithecus* as either a human or ape lasted for several decades.

There were several shared dental traits between *Gigantopithecus* and the early hominins which obscured the classification of *Gigantopithecus*^71,79,106,127,128^. Some of these traits are retentions of primitive traits while others are the result of homoplasy. For example, when comparing *Paranthropus* to *Gigantopithecus*, the robust mandible and molarized premolars are superficially very similar, however these features are the result of convergent evolution. As more fossils have been discovered and methods of analysis have been refined, researchers have been able to resolve these issues and gradually refine phylogenetic models^6,122,129^.

Current phylogenies group *Gigantopithecus* with the pongines based on a common link to *Sivapithecus*, but the morphological links between the *Gigantopithecus* and *Pongo* *weidenreichi* could be debated^4,14,131-133^. However, a recent enamel proteome study provides new evidence^7^, which concluded that *Gigantopithecus blacki* was a pongine that diverged during the Miocene which supported von Koenigswald’s original position. Relationships to other pongines could potentially be clarified once additional proteome studies have been completed.

f. Relationship to “*Indopithecus*”

In 1969, a new species of *Gigantopithecus* was named, *Gigantopithecus bilaspurensis*^130^. This species was named using a partial mandible from the Late Miocene site of Haritalyangar in the Siwalik Hills, India. The partial mandible was discovered by a farmer when he was a child, 24 years prior to the publication. It was determined that this partial mandible belonged to the same species as an isolated molar that von Koenigswald named “*Indopithecus*” *giganteus*. Therefore, *Gigantopithecus bilaspurensis* became a junior synonym of *Gigantopithecus giganteus*^71^*,*^126,134-137^. The Haritalyangar mandible was smaller than the later *Gigantopithecus blacki* and lacks molarized premolars but it had larger dentition than *Sivapithecus*. *Gigantopithecus giganteus* was about half the size of the later *Gigantopithecus blacki*. *Gigantopithecus giganteus* was the likely ancestor to the *Gigantopithecus blacki* and served as a connection to the pongines. Cameron^138^ proposed to resurrect the name “*Indopithecus*” *giganteus* to separate the Indian/Pakistan specimens from the Chinese fossils based on mandibular shape, but this change was disputed as the level of support was not strong enough to justify different genera when both extant and fossil groups were included in the analysis^139^.

g. Sexual dimorphism and behavior

The large variation in size seen in the *Gigantopithecus blacki* mandibles has been interpreted as sexual dimorphism. The level of sexual dimorphism seen in *Gigantopithecus blacki* was high when compared to the extant great apes. Mandible III has a corpus depth that is 40% greater than in Mandible I compared to the 16-20% difference between male and female gorillas^4^. Oxnard analyzed a collection of 1,094 *Gigantopithecus* teeth from three Chinese cave sites^140^. The level of sexual dimorphism in the teeth was high enough that they would be sorted based on noticeable differences in size, so predictably, the teeth followed a bimodal distribution indicating substantial sexual dimorphism.

High sexual dimorphism in primates was associated with polygyny, single male, multi-female group structure, as seen in gorillas. Based on this polygyny group structure, Oxnard assumed that there would be a ratio of two females for each male in species with high sexual dimorphism. So, when the male to female ratio in *Gigantopithecus* were almost equal, Oxnard concluded, based on the sex ratio, that *Gigantopithecus* was not polygynous and that an alternative explanation for the high sexual dimorphism must exist. However, long term primate behavior studies have disproven Oxnard’s assumption. A study of seven wild primate groups, including chimpanzees and gorillas, found that at birth all the groups had a 1:1 female to male ratio^141^. Solitary male gorillas and all male groups have been observed along with polygyny groups which clarifies the equal sex ratios^142^. Due to the level of sexual dimorphism, *Gigantopithecus* likely had a group structure similar to gorillas.

High levels of sexual dimorphism in primates has also been associated with male-male competition^143^. In particular, canine size in apes has been linked with male-male competition. *Gigantopithecus blacki* was unusual for having the highest level of sexual dimorphism, but relatively small canine size. The canines of *Gigantopithecus blacki* only project slightly above the occlusal surface of the cheek teeth. By necessity, *Gigantopithecus blacki* aggressive behaviors would be different from other apes, because the canines would not have been prominent enough to display^4^.

h. Morphological changes

The large sample size of fossils from dated cave sites allows for analysis of morphological changes in *Gigantopithecus blacki* over time. Zhang analyzed changes in size over time using fossils from five cave sites as well as drug store collections^144,145^. These initial studies determined that *Gigantopithecus* tooth size increased over time with the youngest fossil being the largest. This hypothesis was tested again in 2015 with a larger sample size from 14 sites in southern China which supported the conclusion that *Gigantopithecus* tooth size increased over time^13^. Other mammals followed a similar trend of growing larger during the Pleistocene^83^. Additional studies of the dental morphology using micro-CT scans indicate increased dental complexity in the late surviving *Gigantopithecus*^3,15^. These dental modifications could indicate changing diet in response to ecological pressure^13^.

i. Reconstruction of diet

Due to the unique dental morphology, significant research has been dedicated to reconstructing *Gigantopithecus* *blacki*’s diet. The dental anatomy of *Gigantopithecus blacki* indicated a specialized herbivore with flat molars, molarized premolars, broad and flat canines, and small, closely packed incisors. The thick enamel may indicate adaptation for an abrasive diet^14,15^. The molarized premolars and very large molars may indicate heavy mastication of fibrous food^17^. These dietary adaptations have been directly compared to the diet of the mountain gorilla^146^. The dental characteristics have also led to comparisons to giant pandas and a bamboo specialization^5,17,134,147^. *Gigantopithecus* and giant pandas both had thick, deep, and massive jaws required for heavy chewing. Giant pandas and *Gigantopithecus* also had high rates of dental caries^106^. Conversely, the high rate of dental caries has also been interpreted as evidence for a high fruit diet similar to chimpanzees^6,18,107^.

An analysis of phytoliths, microscopic pieces of silica from plants, bonded to dental enamel indicated a more diverse diet that still included bamboo^5^. The shape of the phytoliths differ depending on the type of plant they come from. More than half of the phytoliths found on the *Gigantopithecus* teeth were long, needlelike which come from the vegetative parts of grasses such as bamboo. The rest were hat-shaped and were attributed to fruits and seeds of dicotyledons, such as durian or jackfruit. Microwear studies also support a diet more similar to chimpanzees than hard object feeders such as an orangutans^148^. The presence of starch grains indicates that *Gigantopithecus* might have also eaten tubers^101^. Carbon and oxygen isotope from tooth enamel have a broad range of values similar to the associated ungulate fauna indicating a broad diet from a diverse C_3_ plants^86,100,101^.

j. Coexistence with *Homo erectus*

Evidence from multiple sites have been purported to support the conclusion that *Homo erectus* and *Gigantopithecus* co-occurred. However, this evidence has since been refuted. The supposed *Gigantopithecus* fossils from Tham Khuyen have been reassigned to *Pongo weidenre**ichi* ^4,88,89^. The proposed *Homo erectus* mandibular fragment from Longgupo Cave and provisionally *Homo erectus* teeth from Mohui, Jianshi, and Sanhe has been identified as the “mystery ape” of China^87,149,105^. *Homo erectus* and *Gigantopithecus blacki* lived in different habitats, so coexistence at the same site would have been unlikely. Irrefutable *Homo erectus* fossils have been found in more open environments such as coastal and marches areas, river valleys, and forest edge environments instead of the more closed canopy forests of *Gigantopithecus* and the *Stegodon-Ailuropoda* fauna^87^.

k. Claims for *G. blacki* evidence outside of China

There have been a few claims of *G. blacki* fossil evidence outside of China but so far most of these fossils have been misindentified and are actually *P. weidenreichi*, for example Tham Khyuen in northern Vietnam^4,88,89^. There have been claims that the Lang Trang fossil assemblage from Vietnam also contained *G. blacki* teeth^150^, but we also consider these specimens to be *P. weidenreichi*. The latest find, sections of two jaws containing teeth from Java Indonesia^151^, appear to be *G. blacki* based on preliminary reports, but their provenance, age and context have not been satisfactorily established. The same local residents that found a *Homo erectus* cranial fragment in 2011, also found these two jaw fragments in 2014 in the same area, both surface finds with no stratigraphic context nor clear provenance^151^. We are doubtfull that *G. blacki* could survive in a tropical/sub-tropical environment with high seasonality, especially associated with fauna typical of open environments. Our study shows that *G. blacki* in China was associated with a mosaic forest environment with a distinctive fauna. Until these issues of provenance and context are fully addressed, the specimen cannot be properly considered. Therefore, based on our current knowledge we are unable to discuss these fossils and potential implications of their discovery location.

**SI section 2: Study area and cave sites**

**1. Background to the region**

Guangxi Zhuang Autonomous Region (ZAR), near the Vietnamese border in southern China, is tectonically situated in the Greater Youjiang basin, roughly bounded by the Shizon-Mile, Ziyun-Dachi and Bobai-Cengxi faults in the north and the Vietnamese Dai Nui Con Voi and Red river faults in the south^152^. The bedrock consists predominantly of cherts and cherty limestone, ranging from 350-250 Ma, or Carboniferous-Permian to Early Triassic, in age^8,152^. Tectonic uplift, river incision and denudation have resulted in a unique landscape that is dominated by conical series of *fencong* or ‘cone’ karst and isolated peaks of *fengling* or ‘tower’ karst situated in wide, flat alluvial plain basins^153^. The caves, rock shelters and fissures throughout the area display a complex but largely common stratigraphy of flowstones, speleothems, fossil-rich karstic breccias and conglomerates^8,151,154-155^.

Karst caves are very common in southern China because of the vast distribution of bare karst^156^. Exogenous clastic sediments are usually transported into these caves by seasonal floodwater and deposited. In certain circumstances, mammalian remains could also be transported into caves this way and/or through certain agencies and fossilized^157^. Different from other bare karst areas, karst landform in Guangxi is characterized by peak forests and peak clusters^156^.

Due to the tectonic uplift of Guangxi area during Quaternary, caves were formed below the water table in the phreatic zone and subsequently lifted into the vadose zone due to a combination of uplift and alluvial incision to form stacked cave systems^156^. Consequently, higher-level caves are older than lower-level caves in the same cone karst system^158^. So are the cave deposits contained within the caves. Generally, this is the case for two of the karstic zones that contain *G. blacki* evidence in this region; the Chongzuo area and the Bubing Basin (Fig 1), where the *Gigantopithecus*-bearing caves are usually higher than non-bearing *Gigantopithecus* caves^3^.

**2. Background to the study areas**

The two study areas are located in a radius of ~100-40 km from Nanning – first around Chongzuo city (22°37’N, 107°37’E), southwest of Nanning (Fig. 1), and the second close to Bubingzhen town in Bubing Basin (23°35’N, 106°59’E) to the north west of Nanning both in Guangxi ZAR (Fig. 1).

Chongzuo is essentially an area of bare karst. Much of the Chongzuo area consists of isolated, steep limestone residuals rising from the planed limestone surfaces, covered quite often by recent alluvium or Quaternary deposits. The karst of Chongzuo is made up of interdigitated peak forest and peak cluster. Due to the heterogeneity of carbonate rocks in this area, there are also patches of low rounded hills formed by karstification of dolomites. Karst caves are usually formed at the base of the peak forest and peak cluster along the path of groundwater discharge in the phreatic zone below the water table^156^. However, the karst towers are constantly growing and transforming by the lowering of the bedrock floor around their bases, the regional tectonic uplift, and the karst denudation^156^. As a result of these complex processes, layers of caves or stacked caves can be formed in these karst towers. When a cave is newly formed, the entrance to it is still at the base of the karst tower and within the zone of seasonal fluctuation, and allogenic clastic sediments can be transported into the cave^158^. In some cases, mammalian fossils can also be transported into the caves this way and calcified into fossiliferous breccia together with the clastic sediments. Consequently, the sediments and the fossils buried in them in the higher caves are in general older than those in the lower caves^158^.

Previous studies have identified several layers of caves in the Chongzuo area^2,3,4^. Mammalian fossils of the Pleistocene *Stegodon* – *Ailuropoda* fauna are frequently preserved in the sediments of these caves. According to previous studies, no cave sediments older than Early Pleistocene have been found in the Chongzuo area. This is because the caves that can hold the older sediments and fossils have been removed from the top of karst towers by denudation. The caves are predominantly narrow solution passageways with only a few caves containing large chambers (e.g., Yixiantian), or narrow fissures (e.g., Shuangtan).

Bubing Basin is a wide open basin surrounded by peak forest landforms and is a tributary of the larger Bose Basin to the north west^159^. The basin is oriented northwest to southeast and is approximately 16 km long by 2 km wide and at its margins more than 50 cave have been discoverted in the late Paleozoic limestone cones between 140-215 masl^160^. This represents a significant number for such a small basin. These caves were formed along paths of basin groundwater discharge, which descenced to lower levels creating dissolution and sedimentary infilling during periods of river incision^161^.

The peak cluster and peak forest landform of the Chongzuo and Bubing Basin areas is a result of many factors, tectonic, lithological, time, fluvial, karst denudation, and climate particularly rainfall as the alluvial plains is lowered by erosion. Thus, each karst mountain reflects unique hydrological processes^156^. There is a good correlation between limestone denudation rates and precipitation, but it is not so clear with respect to temperature. The average annual precipitation of the Chongzuo area is 1400-1800 mm and strongly seasonal^162,163^. The mean temperature in July is about 28˚C and that in January is about 12˚C. The southeast monsoon rainy season in South China begins in early April and there are further rains in July and August. As in other parts of China, the seasonal distribution of precipitation is not homogeneous. Over 60% of the annual precipitation falls from April to August ^162,163^. This high degree of seasonality forces high denudation rates in limestone, rapid dissolution and karstification and affects landscape incision and cave sequences as they can be flushed out more readily causing truncation/erosional episodes.

**2. Caves included in this study**

A total of 22 caves were analysed as a part of this study over two areas known to contain *G. blacki* evidence; Chongzuo vicinity (15 caves) and Bubing Basin (7 caves). Eleven of these caves contained evidence of *G. blacki* within the fossil breccia layer and eleven contained a dominant fossil breccia but did not bear *G. blacki* evidence. The details of each of these caves are provided in Table S2 along with plans, profiles, stratigraphy and 3D models where appropriate (Fig. S1a-v).

Spatially, the caves can be grouped into areas. In Chongzuo, there is a cluster of caves to the north and south of Chongzuo city. Around 30 km to the north Bapeng cave (22°36’23.76”N, 107°16’00.42”E)(Fig. S1i) is situated on the Heishui river, which is a tributary of the larger Zuojiang river. Approximately 3 km to the northeast is Mafeng (22°34’32.00” N, 107°17’03.45”E) (Fig. S1p) and Xiaokou caves (N22°36.875’E 107° 18.825’) (Fig. S1s), with Baxian Cave (N22°34’24.3” E107°21’00.9”) (Fig. S1n) to the east. Ten kilometers to the north is the famous Daxin Cave (N22°41’23.09” E107°14’083.00”) (Fig. S1h) where^10^ first found *insiti* *G. blacki* remains. The second cluster is located to the south of Chongzuo in and around the Chongzuo Ecological Park, ~12 km southeast of Chongzuo city. It contains the Eco Park caves of Queque (22°19’25.79”N, 107°28’23.2”E) (Fig. S1d), Sanhe (N22°16'26.42", E107°30'27.27")(Fig. S1c) Baikong (N22°17'43.49", E107°30'0.08") (Fig. S1a) and Gongjishan (N22°17'35.14", E107°29'47.17")(Fig. S1l) and the Mulan Mountain cluster including Shuangtan cave (22°19’23.38”N, 107°27’44.69”E) (Fig. S1k), and Hejiang (22°15’22.47”N, 107°28’43.53”E) (Fig. S1j). To the northeast of this cluster is the lone cave of Quzai (N22°27’43.06” E107°46’02.02”) (Fig. S1m) and to the southeast of the Eco Park are the twin caves of Yanliang (N22°12’83.01” E107°36’35.02”) (Fig. S1g) and Yixiantian (N22°13'57.78", E107°36'37.50") (Fig. S1f) on either sides of a cone karst. In between the north and south clusters lies Zhanwang cave (N22° 26.850' E107° 24.115') (Fig. S1e) on a terrace of the Zouliang River.

**Table S2**: Location, altitude and description of the 22 caves included in this study. The altitude refers to the height of the cave entrance with the height of the fossil breccia in brackets

|  | **CAVES** | **LAT/**  **LONG** | **ALT**  **(m)** | **DESCRIPTION** | **FOSSIL BRECCIA** | ***G. blacki*?** |
| --- | --- | --- | --- | --- | --- | --- |
| **CHONGZUO** | SHUANGTAN  (CSHT) | N22°19’23.38” E107°27’44.69” | 194 | Located on the east side of Mulan Mountain, 3 narrow connected shaft passageways ~1.0m wide, 7 m high and 26 m long – Corridor A is longest and contains G | In corridor A – hard cemented breccia with a silty clay matrix with overlying and underlying flowstones (three) – pre-excavation spanned the width of the passage evident on both sides. Can be traced in corridor B | Y |
|  | BAPAENG (CBAP) | N22°36’23.76” E107°16’0.42” | ~214 | A small passageway leads into a larger chamber with 2 floors divided by a thick flowstone. The stratigraphy is complex -the breccia covers one wall and seems to have forcefully tilted the existing laminated clay deposits, and then undercut by more recent erosion | The breccia is thick ~4 m with a coarse sandy silt matrix with large angular clasts and is overlain by a flowstone and upper silty sand unit. Breccia contains G teeth and other fossils | Y |
|  | HEJIANG (CHEJ) | N22°15’22.47” E107°28’43.53” | ~179 | Located to the west of SHT in Mulan Mt it has a large entrance opening into one small but tall chamber, the breccia is capped by flowstone and underlain by silty layer with interbedded sands | The breccia is thick ~3 m homogenous silty sand matrix containing fossils – mostly teeth | Y |
|  | BAXIAN  (CBAX) | N22°34’24.3” E107°21’00.9” | 185 | Large wide entrance opens up into long solution chamber that rises up at the rear and narrows into a small fossil chamber | Remnant breccia with silty sand matrix preserved in 4 areas all capped with a flowstone ledge and a sharp LBC. This unit was eroded out and replaced by a younger fill below | N |
|  | DAXIN  (CDAX) | N22°41’23.09” E107°14’083.00” | 448 | Situated in an upland valley at the top of a cone karst the entrance is a small solution tube that extends 20 m and opens into a larger chamber containing breccia. The deep sediments contain a number of layers. | The reddish sandy clay breccia containing G is underlain by a sterile silty clay unit and overlain by a yellowish sandy clay breccia | Y |
|  | MAFENG  (CMF) | N22°34’32.00” E107°17’3.45”   \|  \| \| --- \| | ~220 | Large entrance leading to a long 25 m chamber with small passageways leading away. Fossils are located at rear in a shelf and in the centre of the chamber both capped by flowstone | Breccia is soft with two distinct layers – upper yellowish clay with some fossils and lower more cemented reddish silty clay containing more fossils – mostly teeth. Both layers can be traced from rear to centre. | N |
|  | YANLIANG  (CYAN) | N22°12’83.01” E107°36’35.02” | 179 | Wide open entrance leading to a main chamber and a smaller side passage containing a G tooth in-situ in the wall. The passage winds back around 20 m but the fossils were all found towards to the front of the cave | The breccia with a yellowish silty sand matrix formerly filled the 3 m tall side passage and was capped by a flowstone and underlain by a sterile yellowish silty sand unit | Y |
|  | YIXIANTIAN  (CYIX) | N22°13'57.78", E107°36'37.50" | 160 | The small entrance lies on a steep cliff and opens out into a long chamber with a high roof that extends for ~1 km into the karst. The fossil chamber is located at the end of a side passage and contains 2 main units | The 2.5 m breccia contains an upper sandy clay matrix and a lower silty clay matrix both capped by flowstones and both contain fossils | Y |
|  | GONGJISHAN  (CGONG) | N22°17'35.14", E107°29'47.17" | 170 (166) | Behind Mulan Mt small entrance extends into a long winding solution chamber for ~500 m. Two areas contain breccia with fossils – near the entrance and deep into the cave | The breccia near the entrance is a fissure flow capped by a flowstone and the rear breccia has a sandy clay matrix cemented by calcite | N |
|  | QUZAI  (CQUZ) | N22°27’43.06”  E107°46’02.02” | 143 (160) | Small cave entrance connects to a long phreatic tube for ~200m and opens \into a small fossil chamber containing two main units | The original silty clay unit is overlain by a conglomerate with a sharp LBC and many rounded clasts and fossils – derived from a fluvial source | N |
|  | ZHANWANG  (CZW) | N22° 26.850' E107° 24.115' | 173 | A series of 5 caves at same level. Sheer cliff face for ~20 m leads into a small entrance and a series of small chambers divided by long winding solution passages. Breccia fossil layer can be traced throughout the chambers at same height but most found at the rear of the first chamber and is capped by a flowstone | Breccia has a reddish brown sandy silt matrix and is dense with teeth including G, blacki and *P. weidenreichi* | Y |
|  | QUEQUE  (CQQ) | N22°19’25.79”, E107°28’23.22” | 198 | Located in Eco Park near Sanhe. The chamber is open and the breccia adheres high up on the walls | The breccia is thick ~6 m with two distinct layers – upper yellowish grey clay matrix capped by a 2 m flowstone and lower yellowish silty breccia also capped by flowstone – both contain G fossils | Y |
|  | SANHE  (CSAN) | N22°16'26.42", E107°30'27.27" | 203 | 16 km northeast of Chongzuo in Chongzuo Ecological Park. Large tubular karst cave 13 m wide and 16 m high – cave is 156 m long. The breccia is located in a side chamber to the southwest of the main chamber | Two depositional units – upper unit 160 m calcareous breccia – lower unit is yellow sands and silts up to 11.3 m thick capped by a flowstone and contain G. blacki fossils | Y |
|  | BAIKONG  (CBAIK) | N22°17'43.49", E107°30'0.08" | 212 | Steep entrance leads into a narrow passageway and a small chamber divided by columns – the breccia is remnant on the cave walls and capped by a thick flowstone | Hard cemented breccia with sandy silt matrix and clasts of cemented clay – contained many G teeth and other fossils | Y |
|  | XIAO KOU (CXK) | N22°36.875’  E 107° 18.825’ | ~259 | Small entrance opening into a large chamber divided into upper and lower by thick 1.5 m flowstone – breccia and conglomerate deposits in lower | Breccia is sterile but homogenous conglomerate is ~2 m thick containing silty sand matrix with river cobbles, bones (mostly post cranial) and teeth | N |
| **BUBING BASIN** | GANXIAN  (CGAN) | N23° 35.965' E107° 00.096 | 191 | Small entrance with a long narrow winding passage with small fossil chamber at the rear with 2m of cave earth | Fossil bearing unit is a 2 m thick silty clay that coarsens up section | N |
|  | UPPER PUBU  (CUPB) | N23° 35.705' E106° 59.392' | 155 | Small entrance leads to a narrow and low winding passageway that contains a small fossil chamber, then connects to a steep chamber and the lower Pubu cave. | Remanent cemented breccia with silty sand matrix and capping flowstone adheres to the wall and looser unconsolidated breccia also capped is found on the opposite wall and floor – both contain fossils | N |
|  | LOWER PUBU  (CLPB) | N23° 35.717' E106° 59.414' | 143 | Entrance located directly below UPB – small tight entrance that turns into a small winding passageway for ~800 m to the rounded fossil chamber at the rear. Contains ~2m of unconsolidated sediments and remanent breccia adhering to roof | Breccia has a silty sand matrix and is dense with fossils | N |
|  | ZHONGSHAN  (CZS) | N23° 34.328' E107° 00.525 | 145 | Located close to Mohui cave – long passageway that extends into a large fossil chamber – 3-4 m above the plain. Breccia blocks with capping flowstone surrounded by younger sandy clay unit | Breccia is a cemented sandy clay containing fossils and stone tools in the capping flowstone | N |
|  | CHUIFENG  (CCFG) | N23°34′27, E107°00′22 | 227 | Bubing Basin 150 km northwest of Nanning, and Chuifeng is in the southeast of the Basin. 20 m long 0.2-2 m wide 1.5-5 m high with two entrances | The cave infill completed filled the rear of the cave to a height of 1.3 m thick containing sandy silts and clays with small limestone breccias | Y |
|  | LUMEI  (CLUM) | N23° 40.103' E106° 53.485' | 150 | Large wide solution chamber that winds back ~200 m a small passageway winds back to a small fossil chamber at the rear capped by a flowstone | Breccia accumulated from a fissure at the rear of the cave that was capped by a flowstone | N |
|  | WUYUN  (CWUY) | N23° 35.272' E107° 00.189' | 165 | Large and wide entrance that extends into a series of large chambers for ~300 m. Breccia in rear chamber | Breccia is very cemented capped by overlying and underlying flowstones | N |

In the Bubing Basin there is a cluster of caves in the south east corner of the basin close to the town of Bubing; Wuyun (N23° 35.272' E107° 00.189') (Fig. S1q), Ganxian (N23° 35.965' E107° 00.096’) (Fig. S1u), Upper (N23° 35.705' E106° 59.392') (Fig. S1r) and Lower Pubu (N23° 35.717' E106° 59.414') (Fig. S1t) and two caves on the same karst cone Chuifeng (N23°34′27, E107°00′22) (Fig. S1b) and Zhongshan (N23° 34.328' E107° 00.525) (Fig. S1o). One last cave is located to the north east of the basin; Lumei (N23° 40.103' E106° 53.485') (Fig. S1v).

Fig. S1 a-v provides a plan, profile, stratigraphy, fossil and sampling locations and dating results of all these 22 cave sites from both the Chongzuo (CZ) and Bubing Basin (BB) areas. The cave name is followed by the sample code used for that cave in brackets. The sampling locations on the plan and profile for each dating techniquies have been presented according to the following symbols; pIR-IRSL dating of feldspars (red triangle), OSL dating of quartz (yellow triangle), U-series dating of carbonates (blue circle), Electron Spin Resonance Dating of quartz (green triangle), Combined U-series-ESR dating of teeth (green hexagon), palaeomagnetism (yellow circle). These symbols correspond with the symbols plotted on the models to represent each dating result, with age in ka on the x- axis and the individual age estimates (a.u.) on the y-axis. Note the age estimates have been plotted within the correct sedimentary unit but the order within the unit does not necessarily represent the sampling depth of each sample. The right pointing arrow represents a minimum age estimate and a left pointing arrow represents a maximum age estimate. All resulting age estimates have been converted to 2 σ uncertainty limit before being entered into the model and the modelled age ranges have also been plotted at 2 σ uncertainty limit. The ‘F1’ refers to an overlying flowstone, ‘G’ refers to the fossil breccia layer that bears *G. blacki* evidence, while NON-G refers to a fossil unit that does not contain *G. blacki* evidence. The thick black horizontal boxes with dashed lines represent the Bayesian modelled age for the boundary of each unit (see Supp 9). Thus, the modelled age range for fossil evidence is constrained by the age range of the upper and lower boundary of the breccia unit. These age ranges have been included in each model.

**
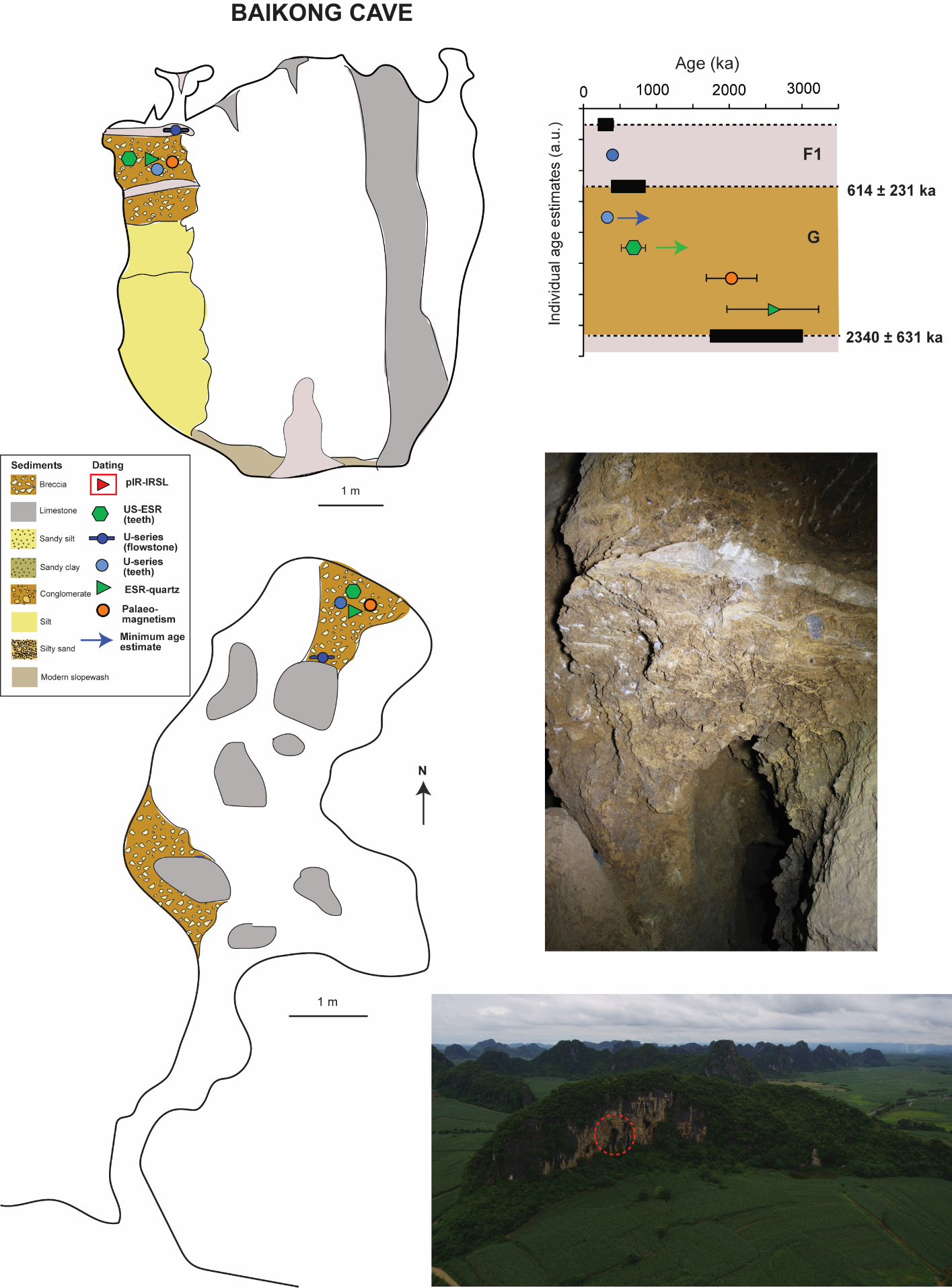
**

**Fig. S1a –** Baikong cave (CBAIK) in Chongzuo – plan, profile, composite stratigraphy, fossil location and dating results. The green hexagons and light blue circles indicate the location of the dated fossil teeth. The palaeomagnetic result is estimated age by combining magnetostratigraphic and biochronologic data and is taken from^8^ (with a 1 σ uncertainity included).

**
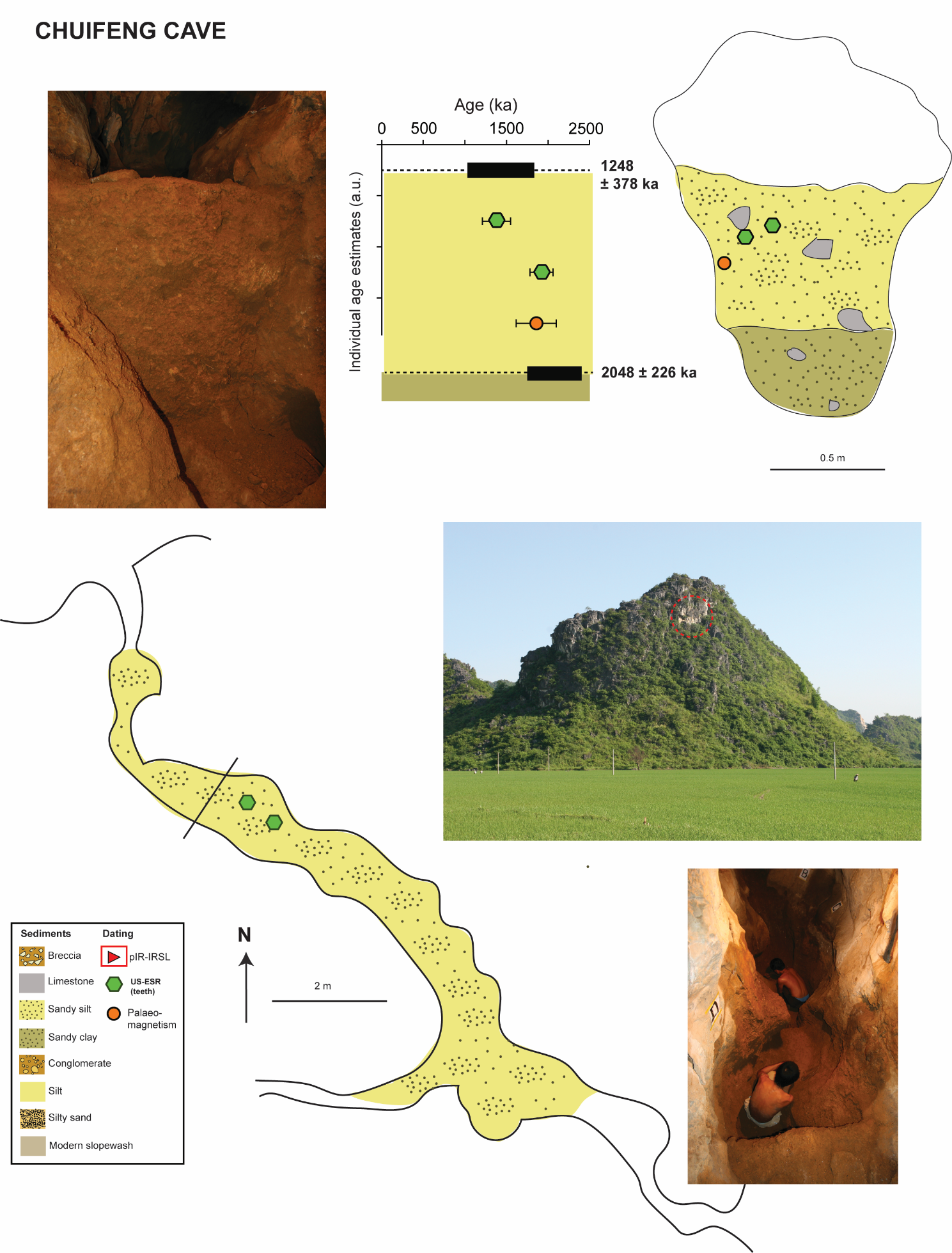
Fig. S1b –** Chuifeng Cave (CCF) in Bubing Basin– plan, profile, composite stratigraphy, fossil location and dating results. The green hexagons indicate the location of the dated fossil teeth. The palaeomagnetic result is estimated age by combining magnetostratigraphic and biochronologic data and is taken from^11^ (with a 1 σ uncertainity included).

**
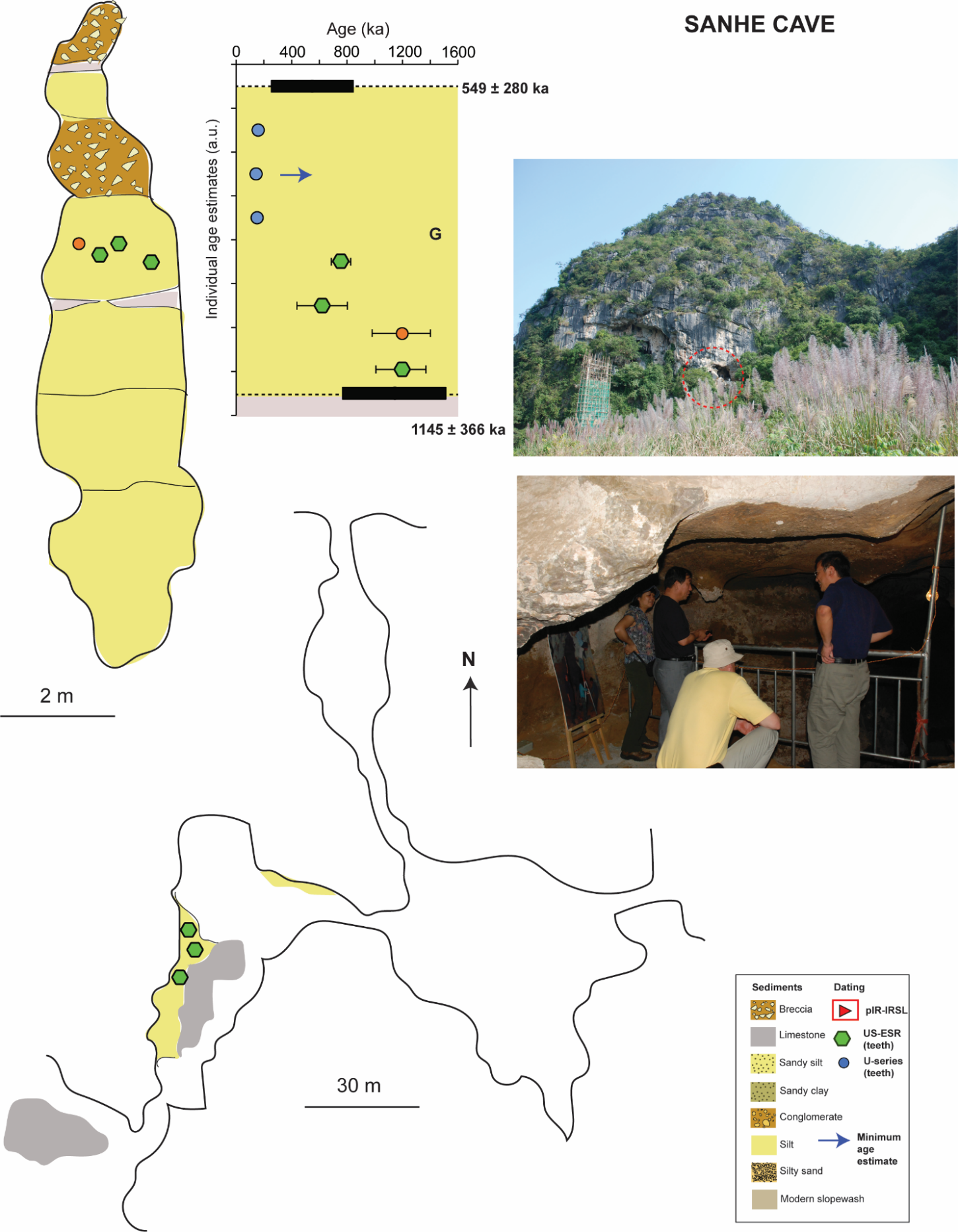
**

**Fig. S1c** - Sanhe cave (CSAN) Chongzuo region– plan, profile, composite stratigraphy, fossil location and dating results. The green hexagons and light blue circles indicate the location of the dated fossil teeth. The palaeomagnetic result is estimated age by combining magnetostratigraphic and biochronologic data and is taken from^8^ (with a 1 σ uncertainity included).

**
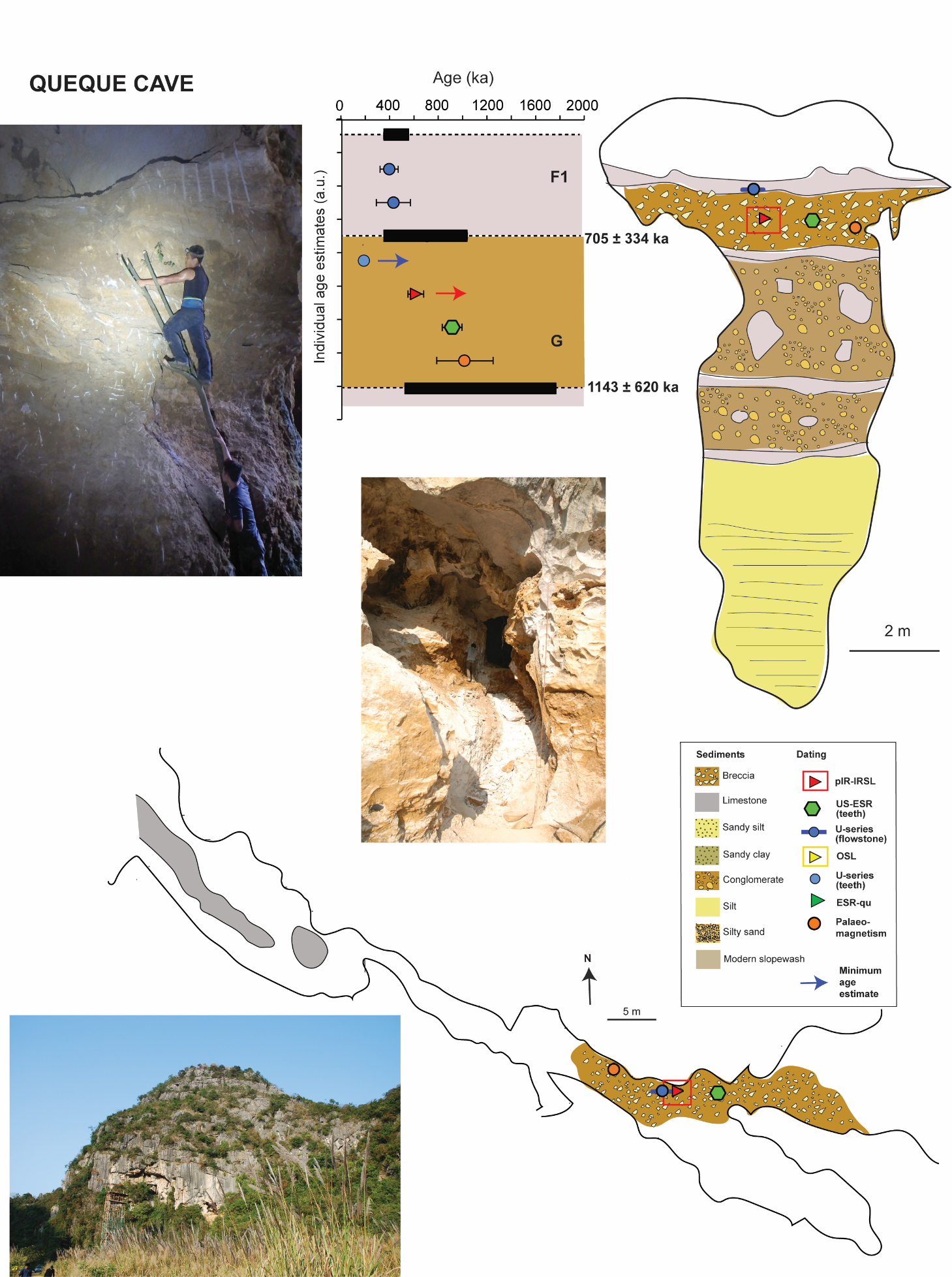
**

**Fig. S1d** - Queque Cave (CQQ) in Chongzuo– plan, profile, composite stratigraphy, fossil location and dating results. The green hexagon indicates the location of the dated fossil teeth. The palaeomagnetic result is estimated age by combining magnetostratigraphic and biochronologic data and is taken from^8^ (with a 1 σ uncertainity included).

**
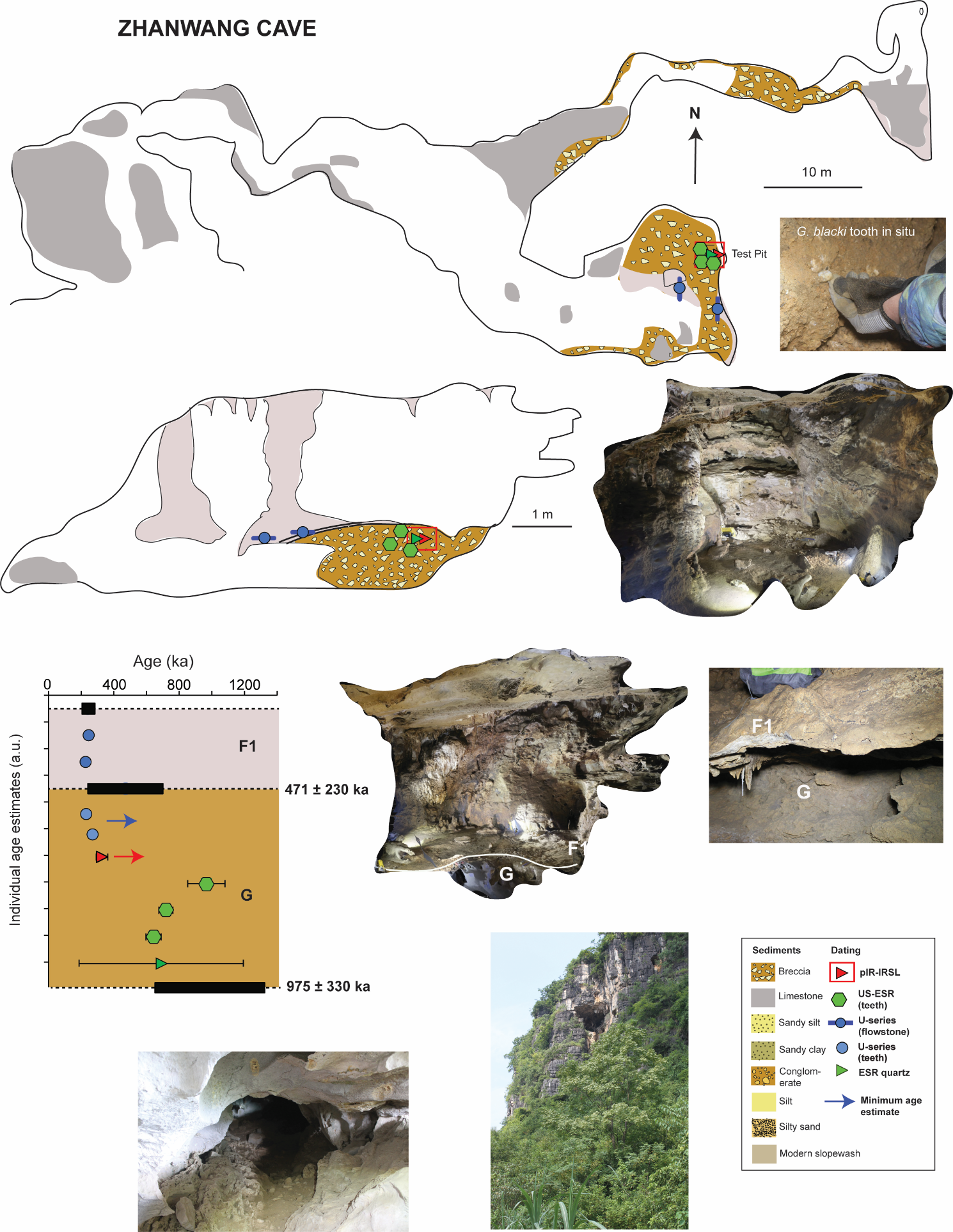
Fig. S1e** - Zhanwang Cave (CZW) Chongzuo– plan, profile, composite stratigraphy, fossil location and dating results. The green hexagons and light blue circles indicate the location of the dated fossil teeth.

**
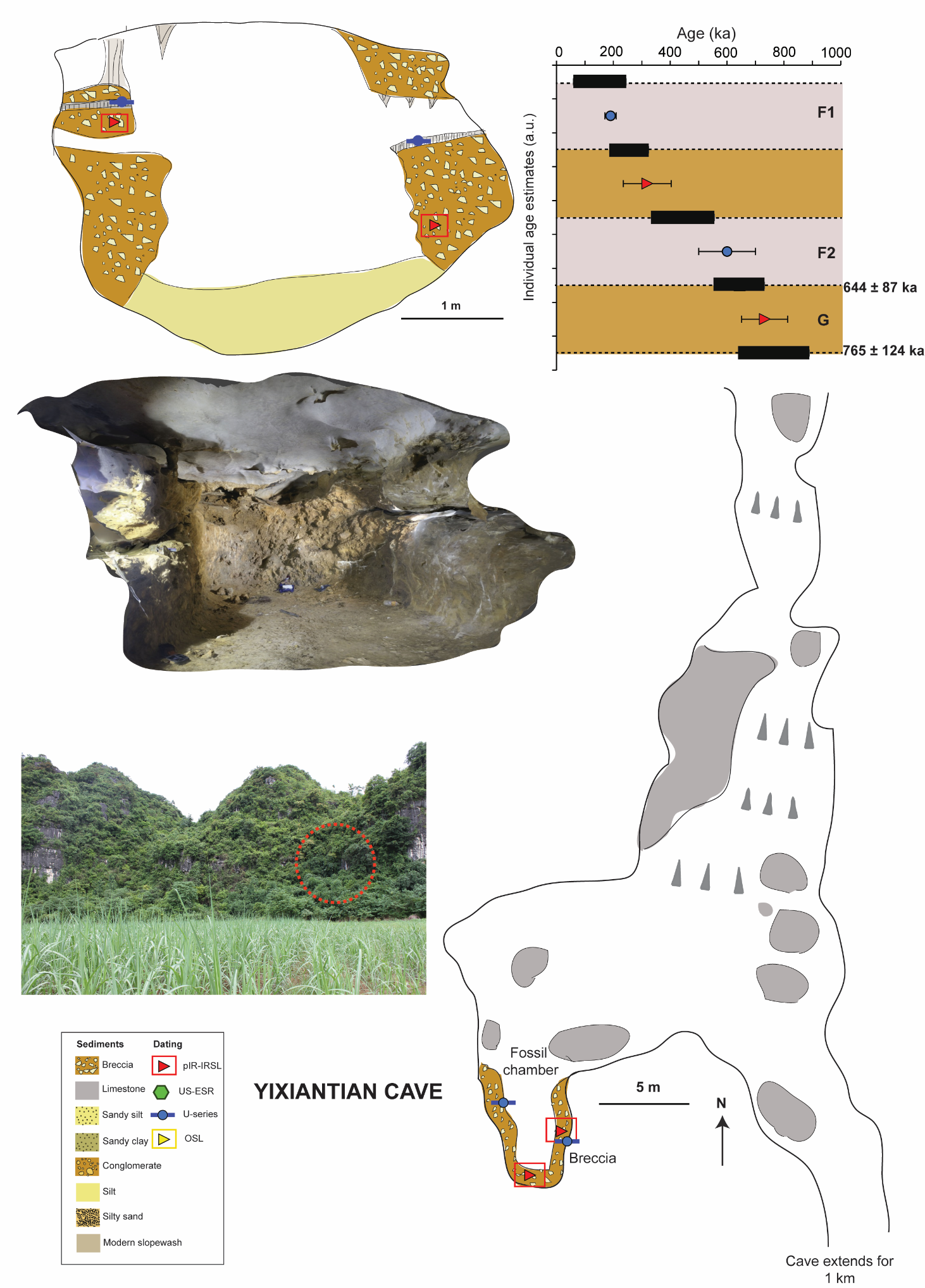
**

**Fig. S1f** - Yixiantian Cave (CYIX) Chongzuo– plan, profile, composite stratigraphy, fossil location and dating results.

**
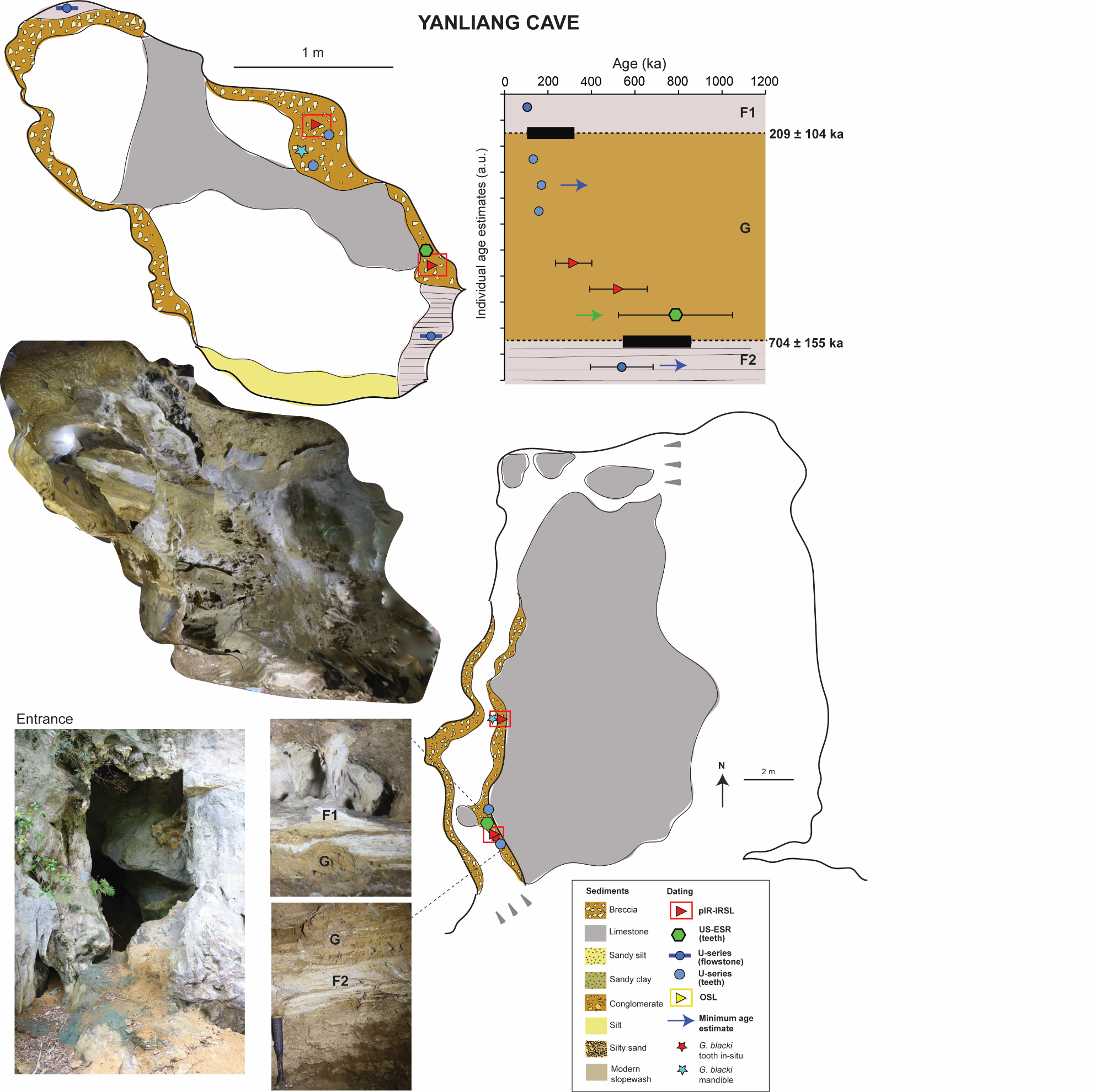
**

**Fig. S1g** - Yanliang Cave (CYAN) Chongzuo – plan, profile, composite stratigraphy, fossil location and dating results. The green hexagon and light blue circles indicate the location of the dated fossil teeth.

**
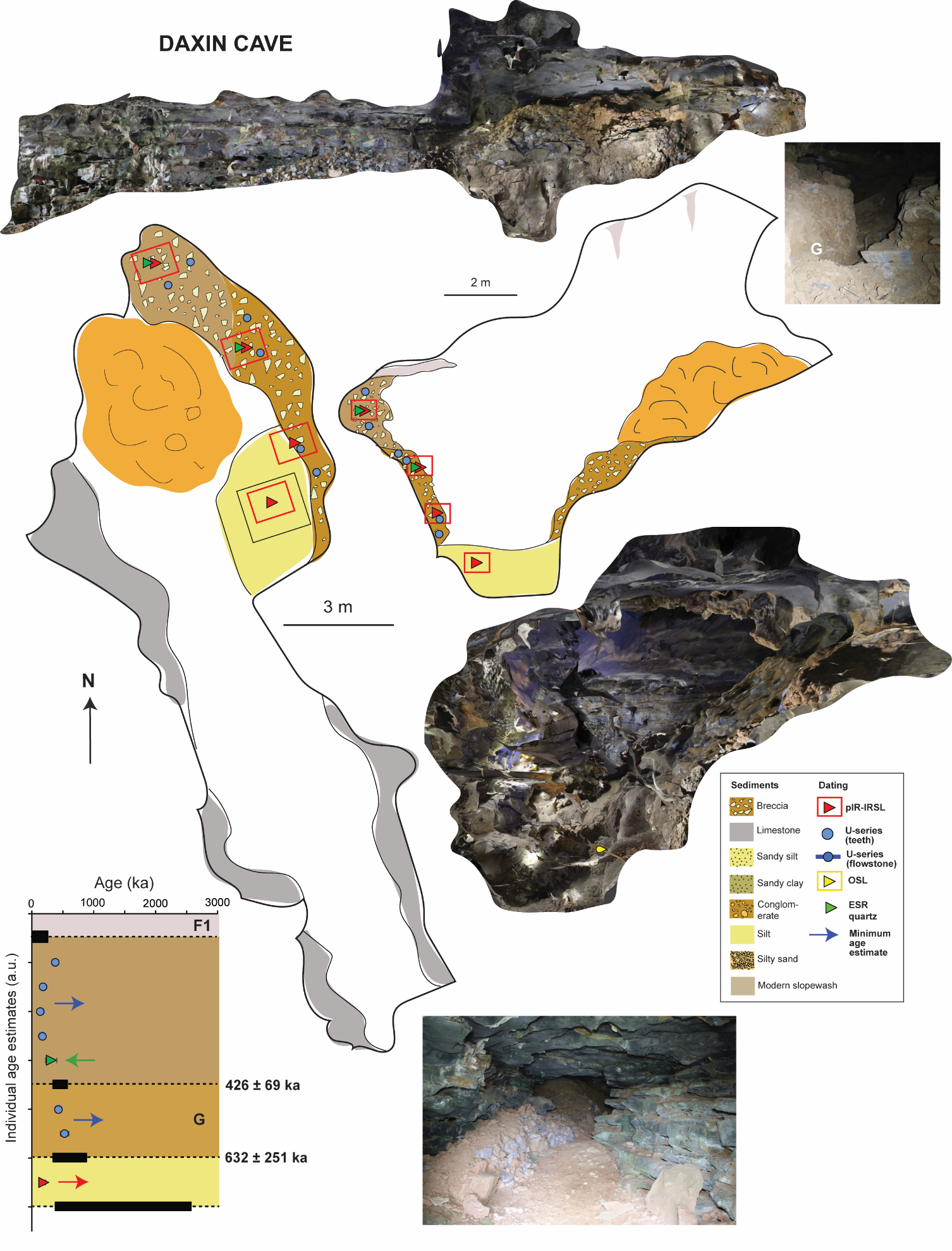
Fig. S1h** - Daxin Cave (CDAX) Chongzuo– plan, profile, composite stratigraphy, fossil location and dating results. The light blue circles indicate the location of the dated fossil teeth.

**
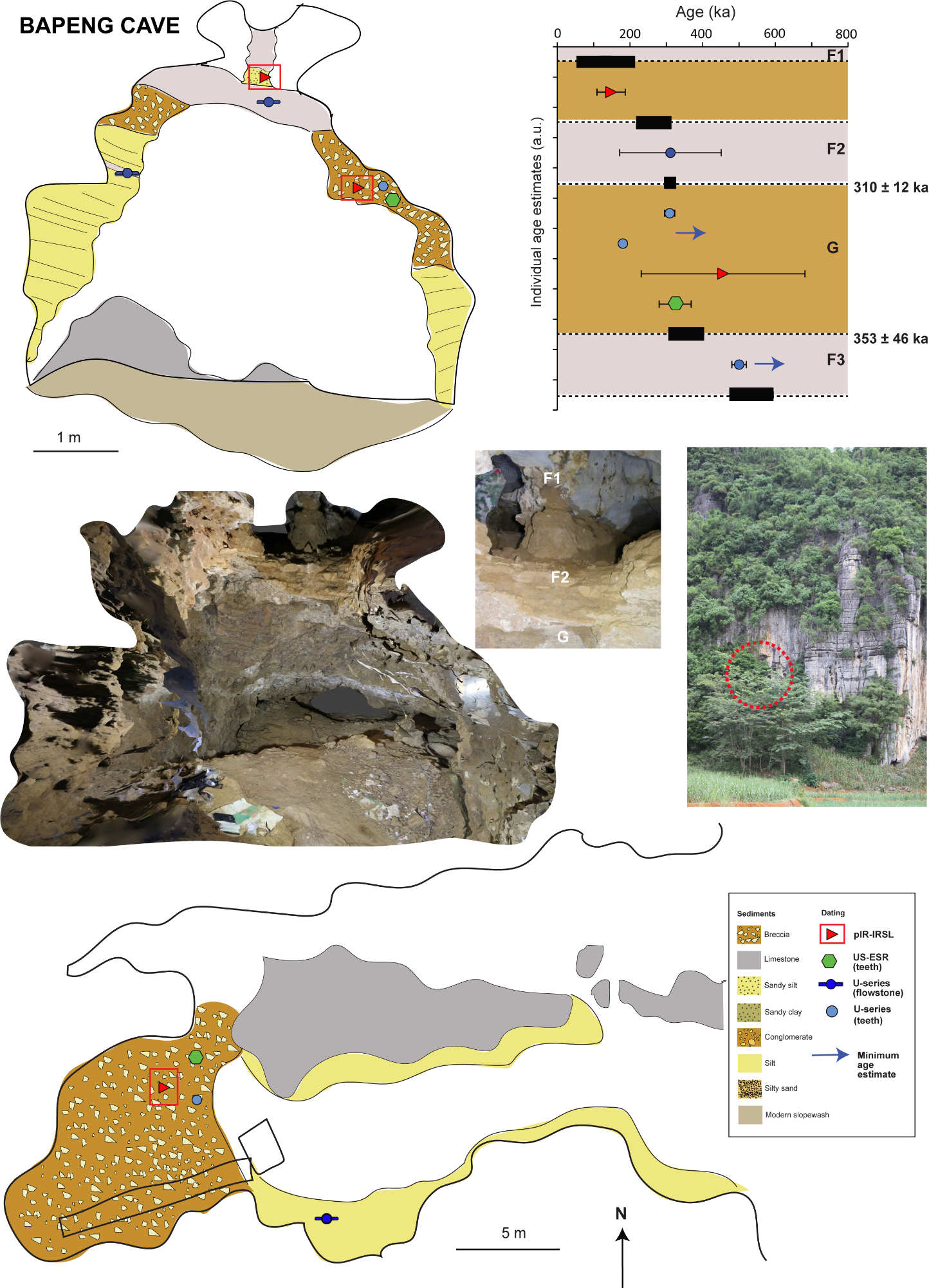
**

**Fig. S1i** - Bapeng Cave (CBAP) in Chongzuo– plan, profile, composite stratigraphy, fossil location and dating results. The green hexagon and light blue circle indicate the location of the dated fossil teeth.

**
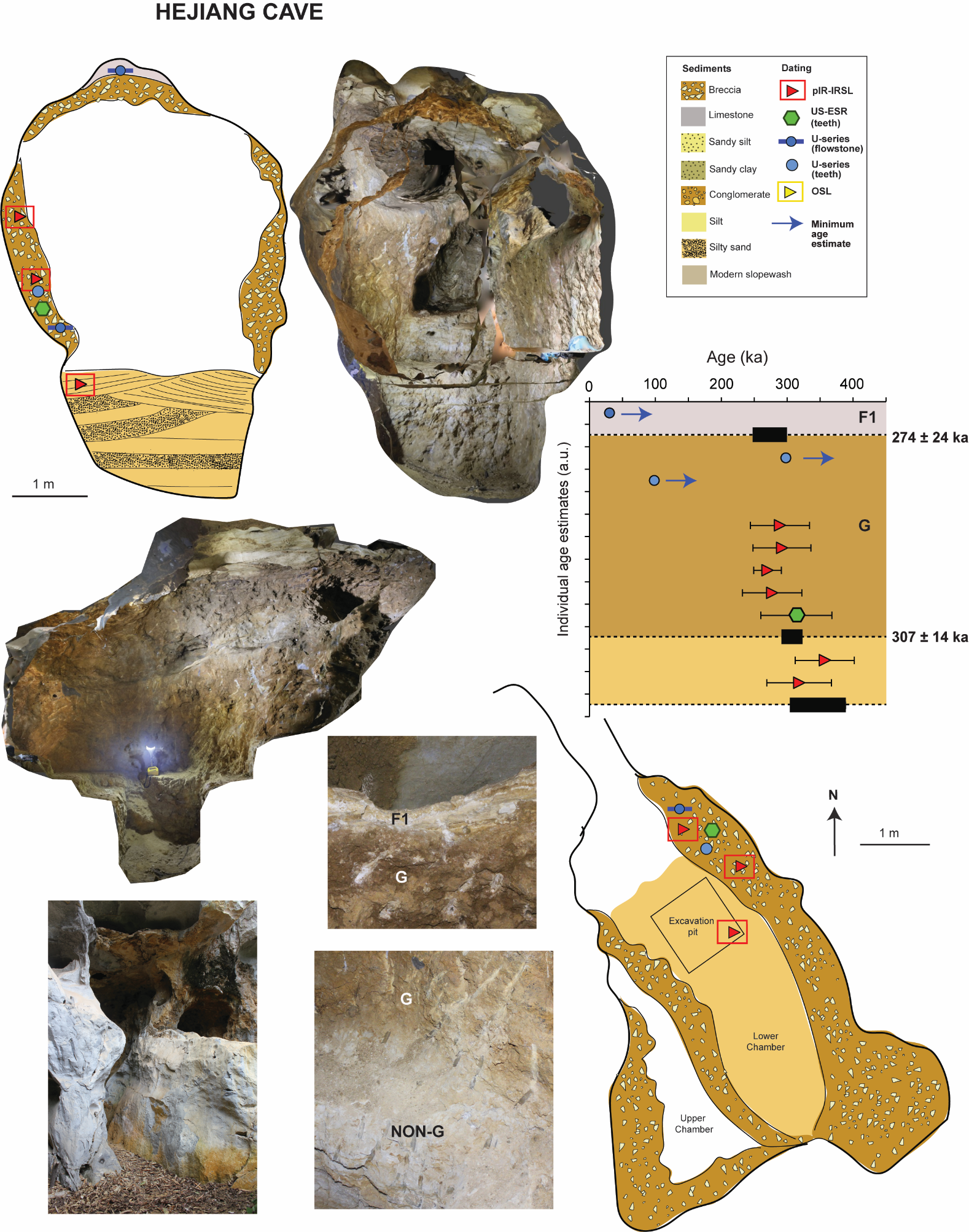
Fig. S1j** - Hejiang Cave (CHEJ) in Chongzuo– plan, profile, composite stratigraphy, fossil location and dating results. The green hexagon and light blue circles indicate the location of the dated fossil teeth.

**
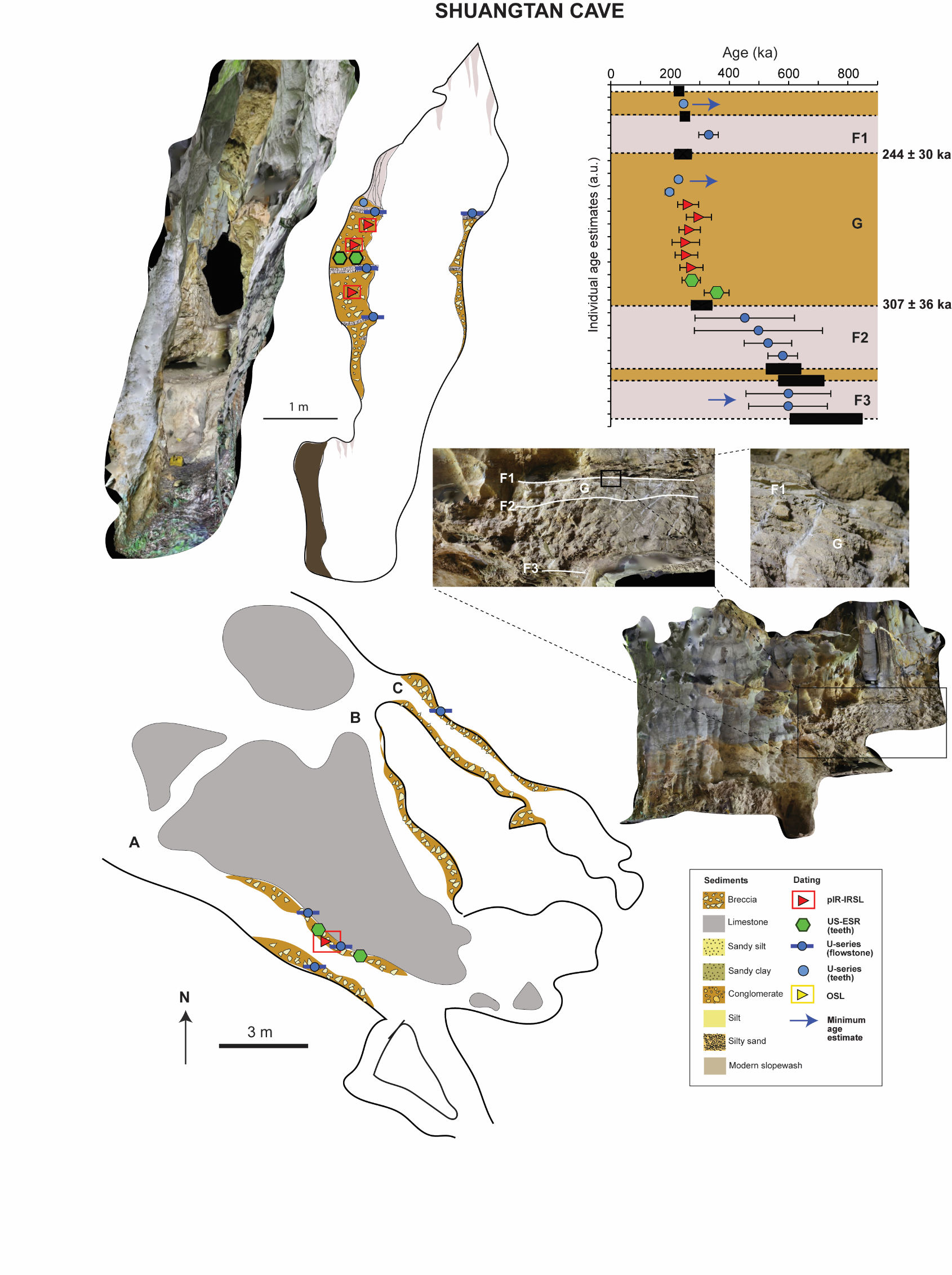
**

**Fig. S1k** - Shuangtan Cave (CSHT) in Chongzuo– plan, profile, composite stratigraphy, fossil location and dating results. The green hexagons indicate the location of the dated fossil teeth.

**
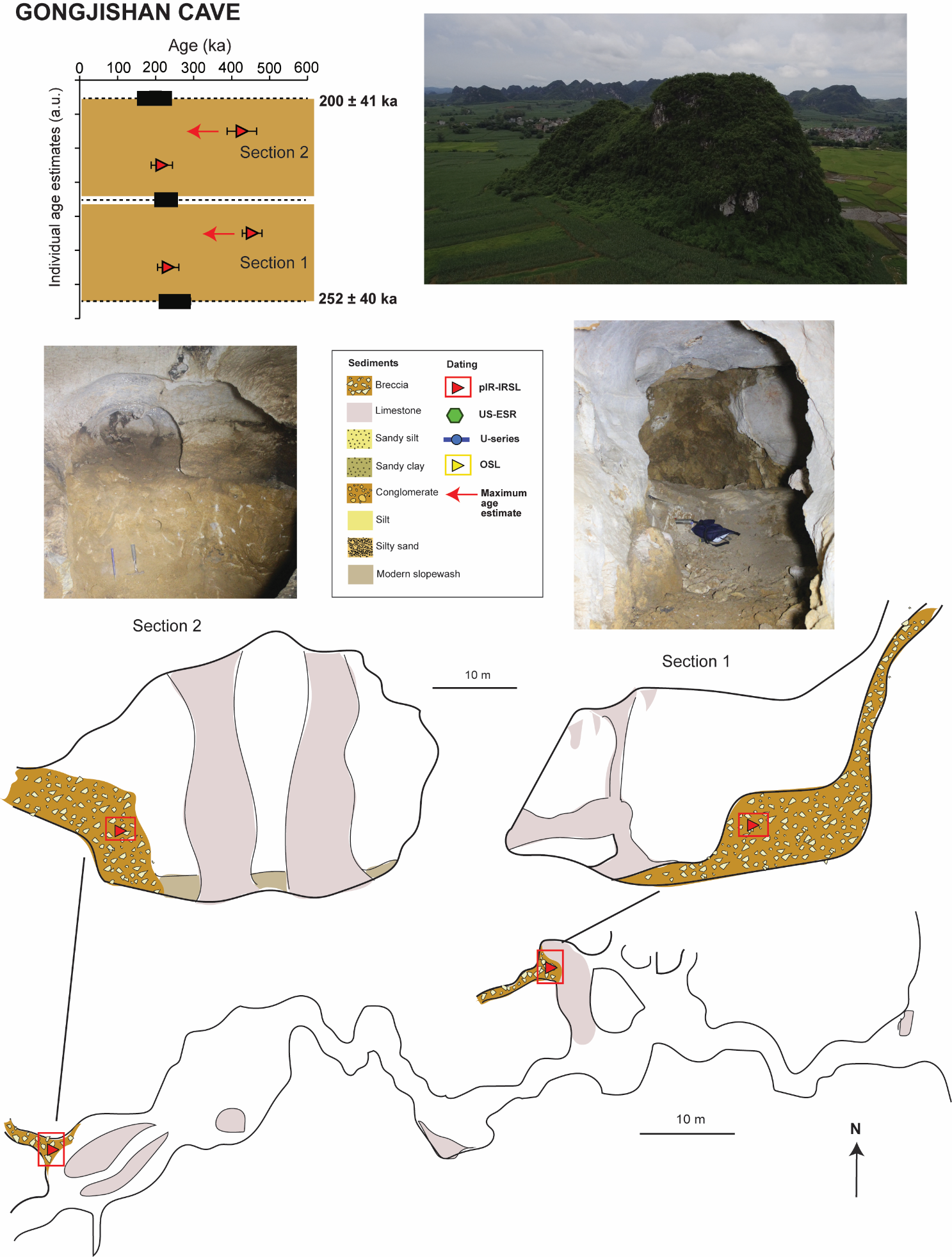
Fig. S1l** – Gongjishan Cave (CGONG) in Chongzuo– plan, profile, composite stratigraphy, fossil location and dating results.

**
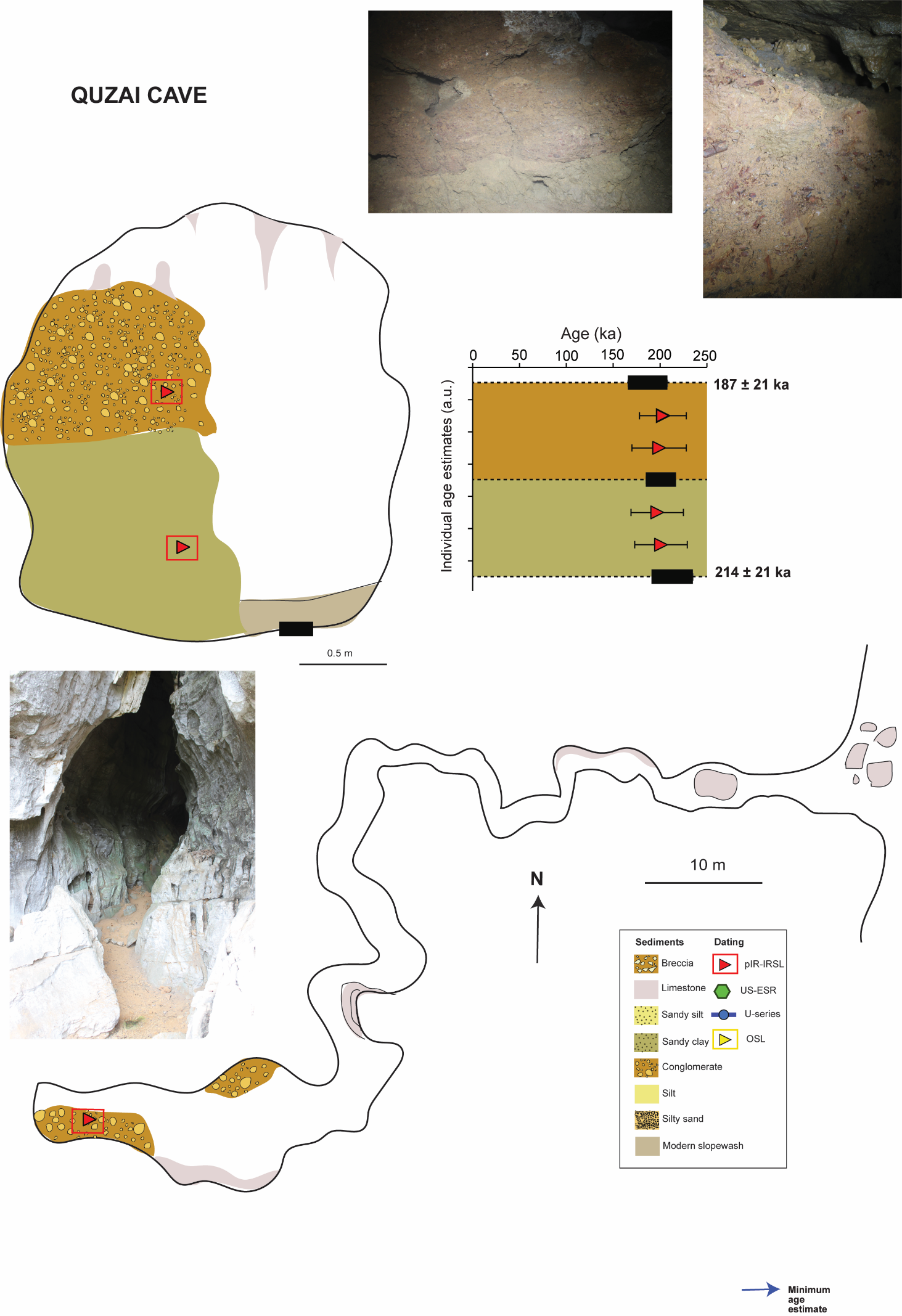
Fig. S1m** - Quzai Cave (CQUZ) in Chongzuo– plan, profile, composite stratigraphy, fossil location and dating results.

**
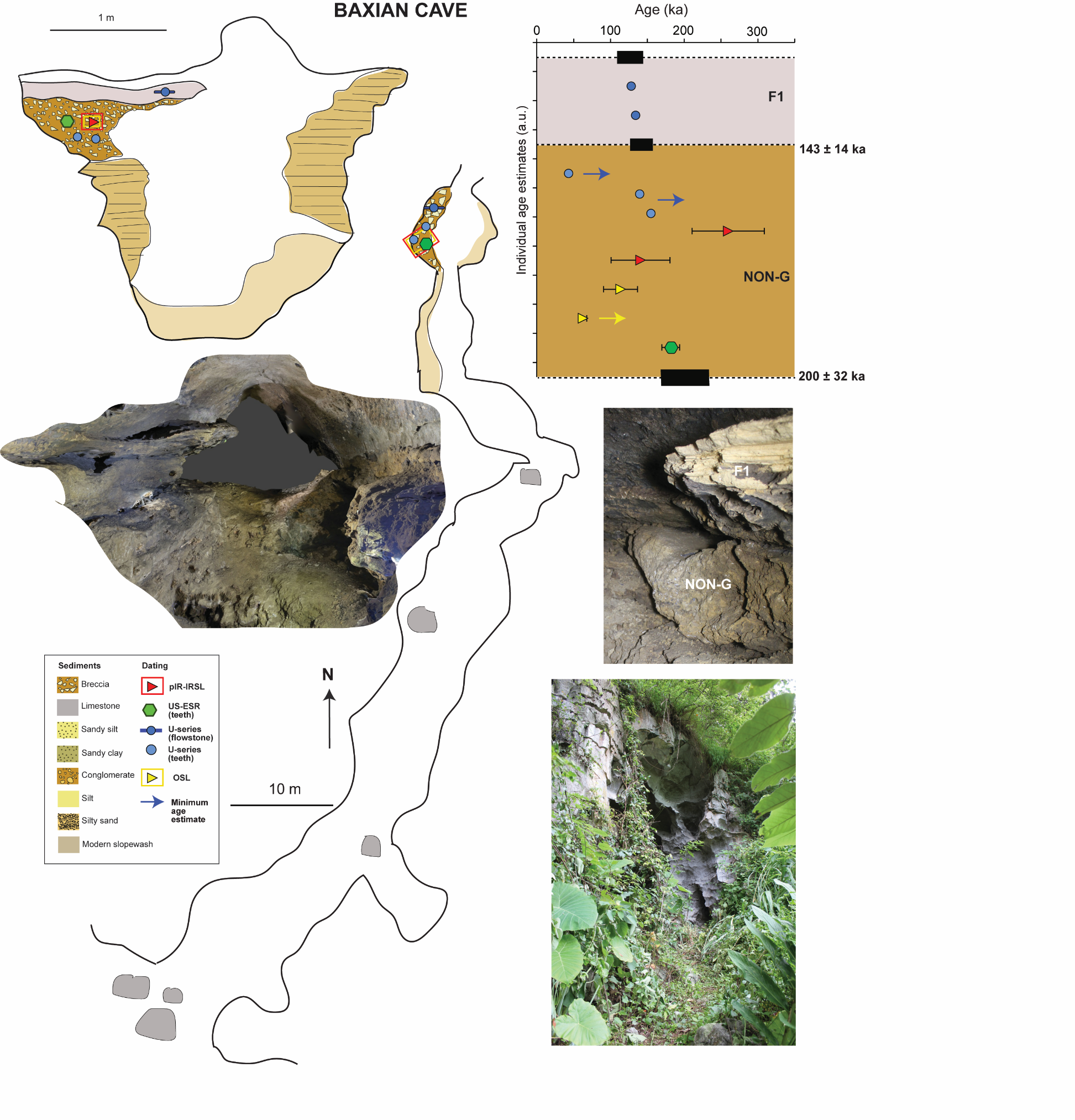
**

**Fig. S1n** – Baxian Cave (CBAX) in Chongzuo– plan, profile, composite stratigraphy, fossil location and dating results. The green hexagon and light blue circles indicate the location of the dated fossil teeth.

**
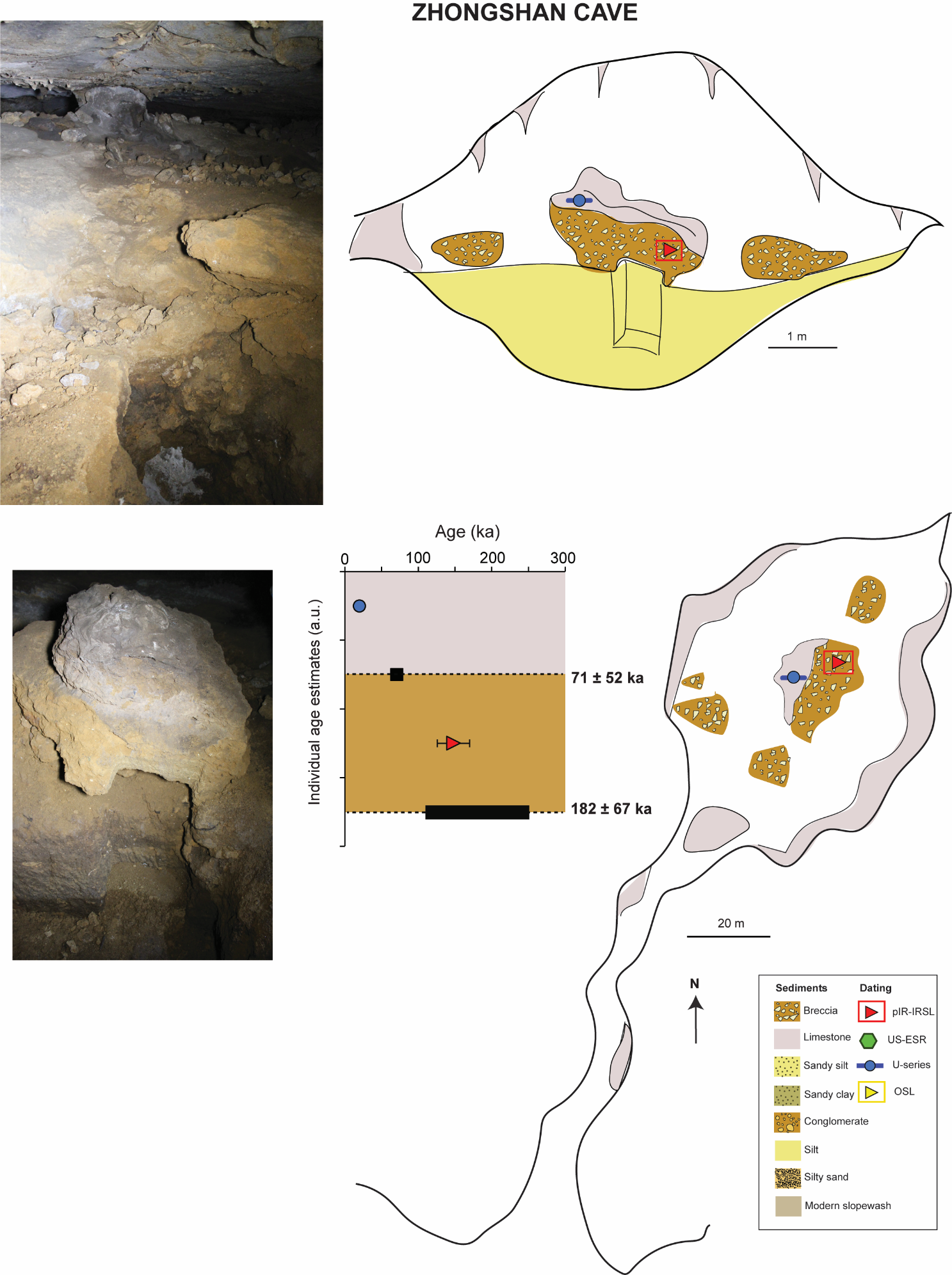
Fig. S1o** – Zhongshan Cave (CZS) In Bubing Basin– plan, profile, composite stratigraphy, fossil location and dating results.

**
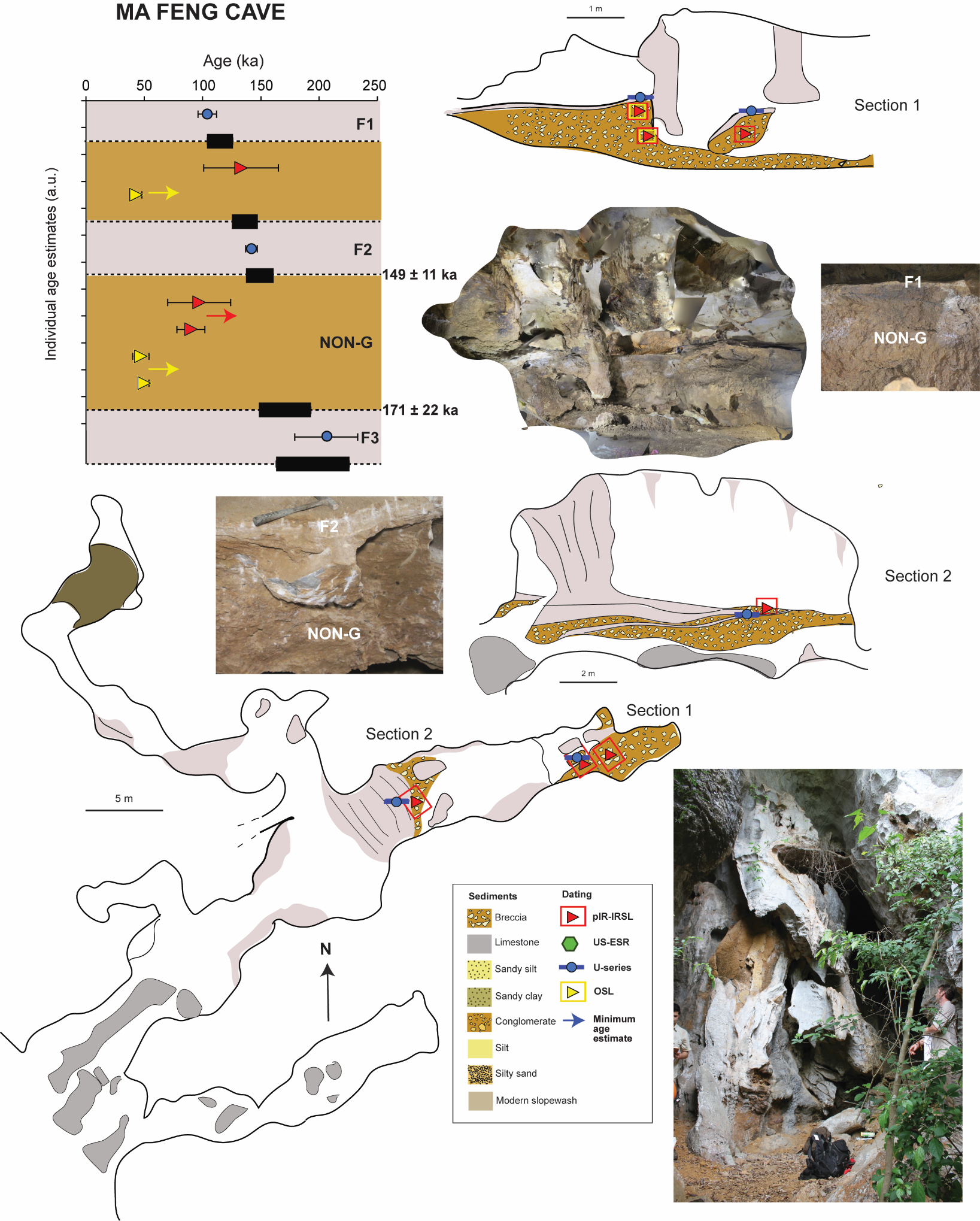
Fig. S1p** – Mafeng Cave (CMF) in Chongzuo– plan, profile, composite stratigraphy, fossil location and dating results.

**
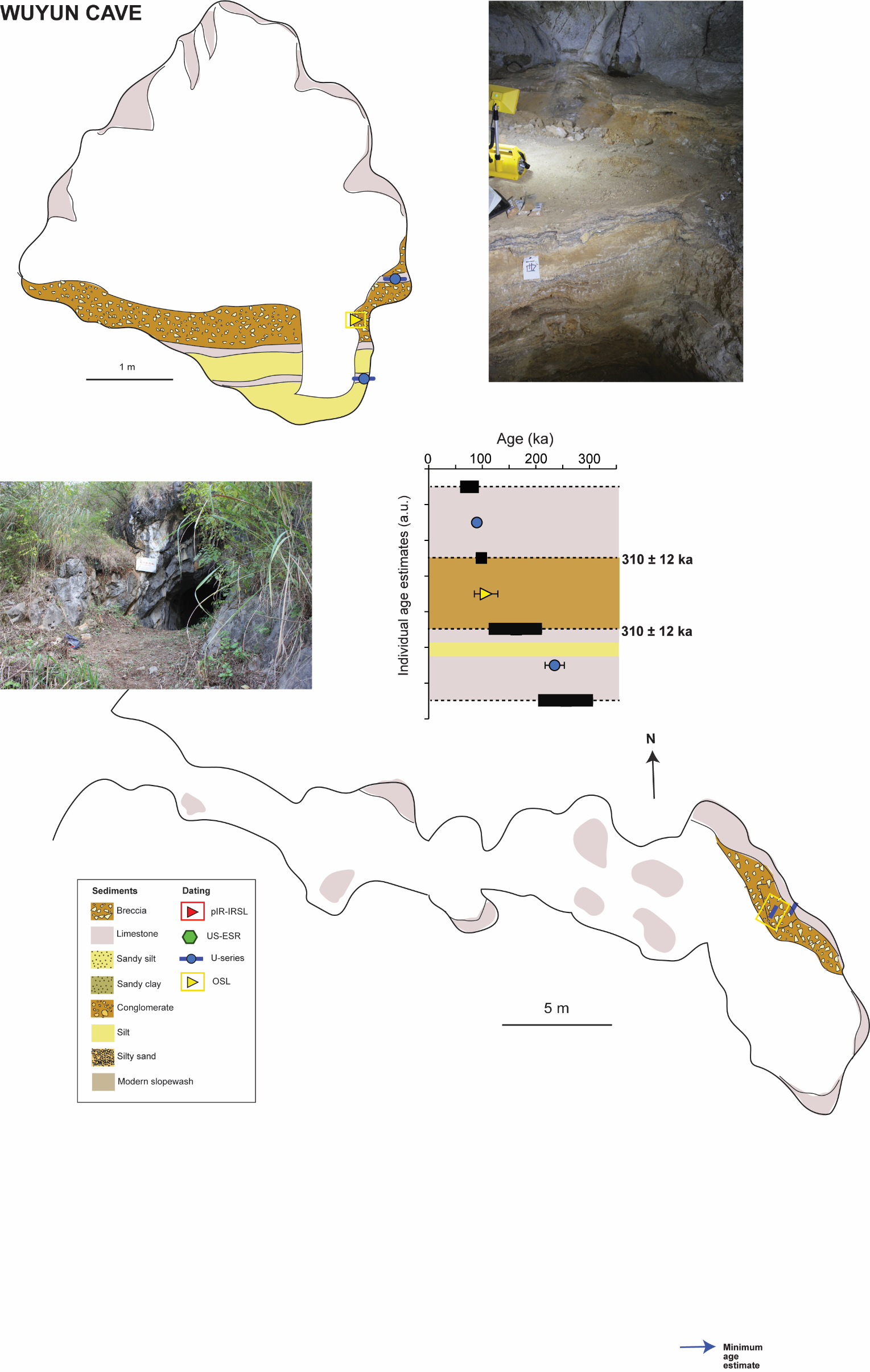
**

**Fig. S1q** – Wuyun Cave (CWUY) in Chongzuo– plan, profile, composite stratigraphy, fossil location and dating results.

**
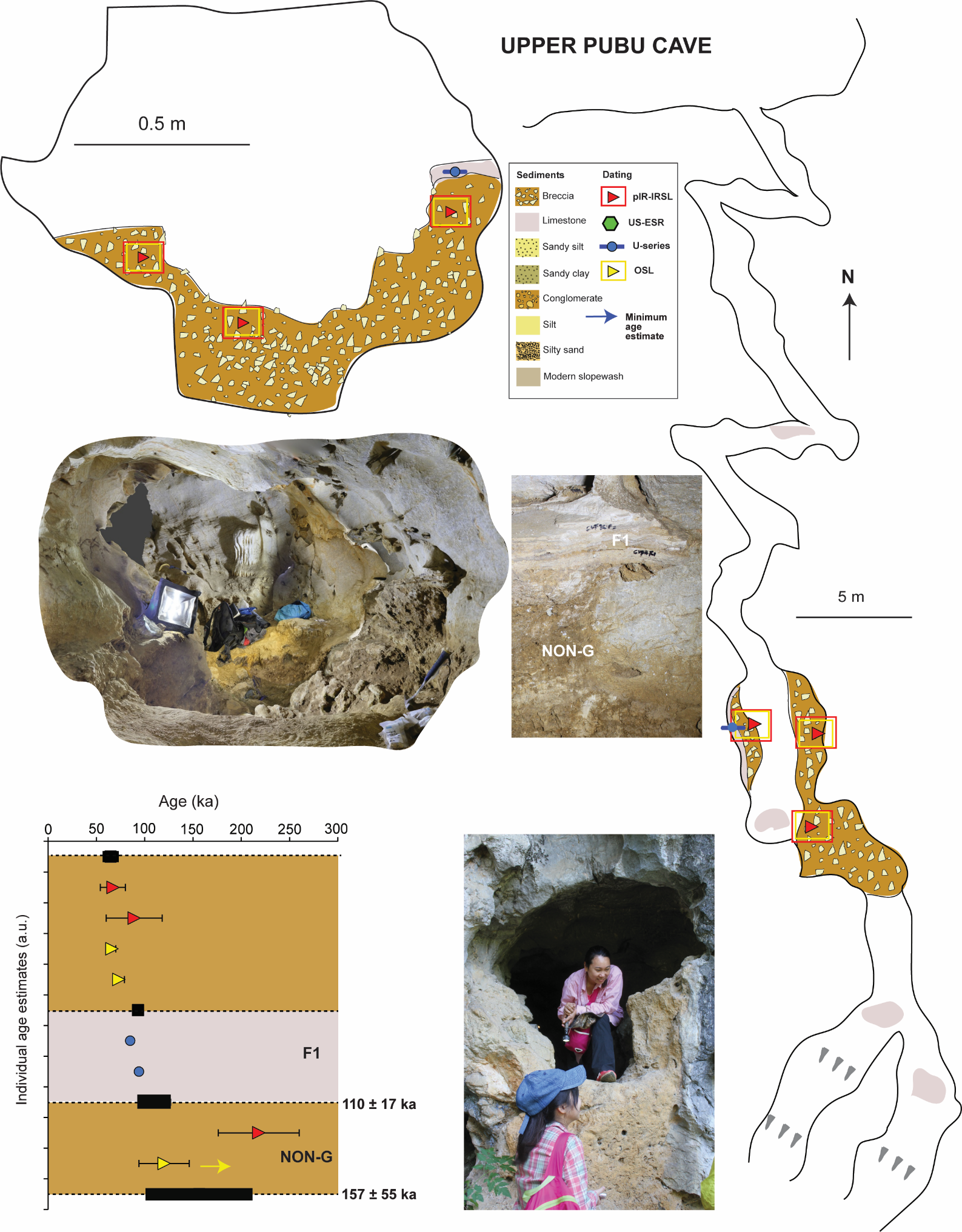
Fig. S1r** – Upper Pubu Cave (UPB) in Bubing Basin– plan, profile, composite stratigraphy, fossil location and dating results.

**
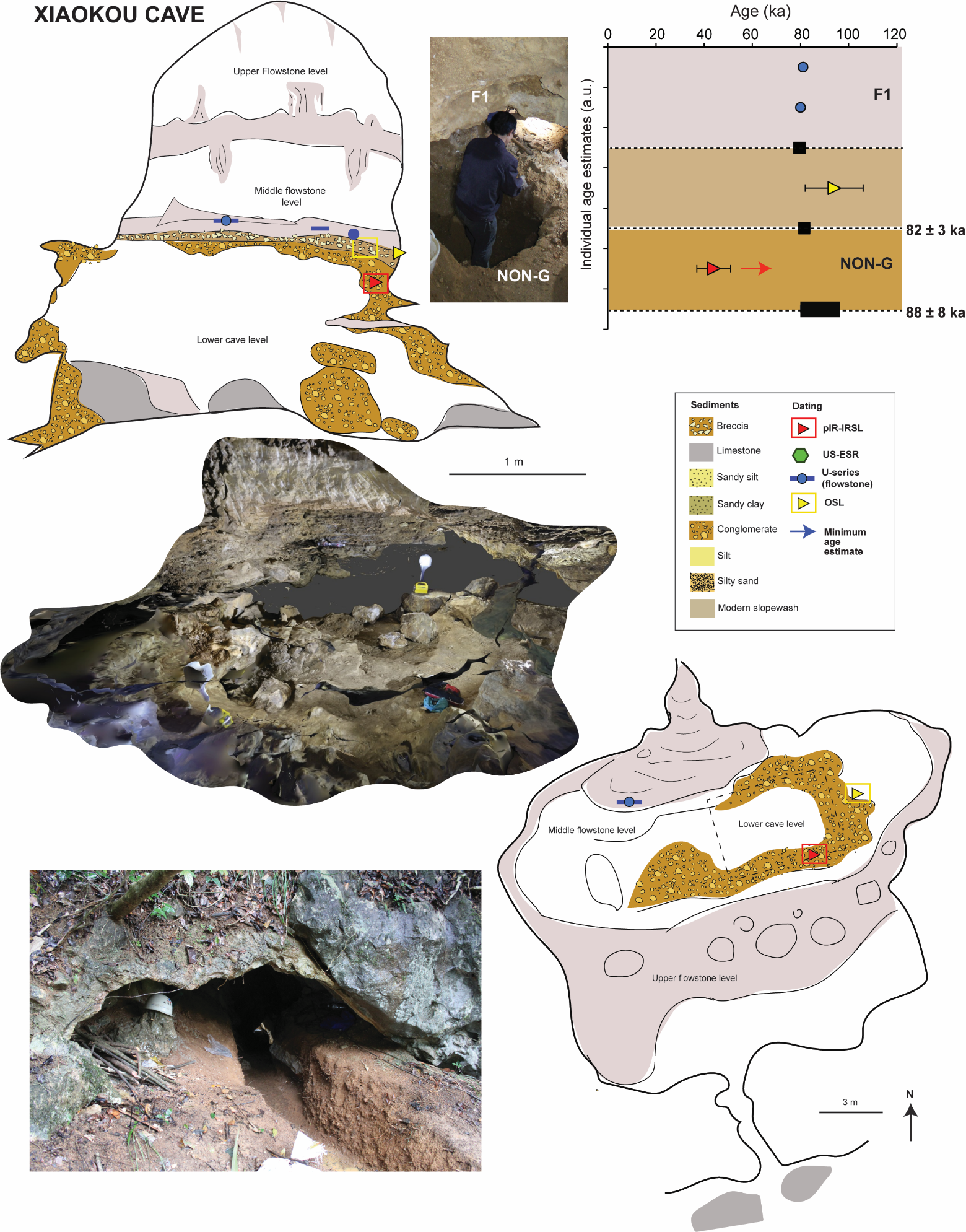
Fig. S1s** – Xiaokou Cave (CXK) in Chongzuo– plan, profile, composite stratigraphy, fossil location and dating results.

**
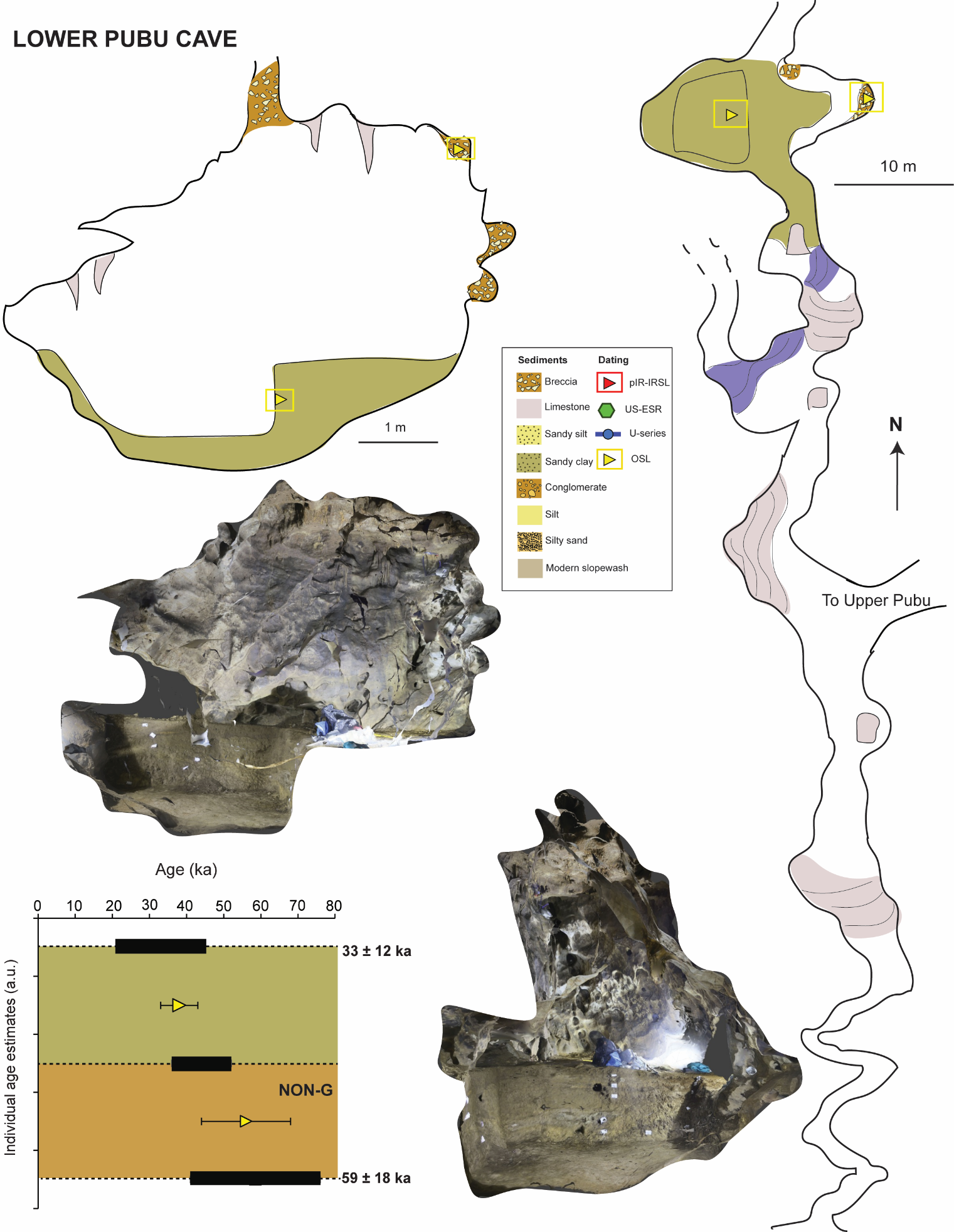
Fig. S1t** - Lower Pubu Cave (LPB) in Bubing Basin– plan, profile, composite stratigraphy, fossil location and dating results

**
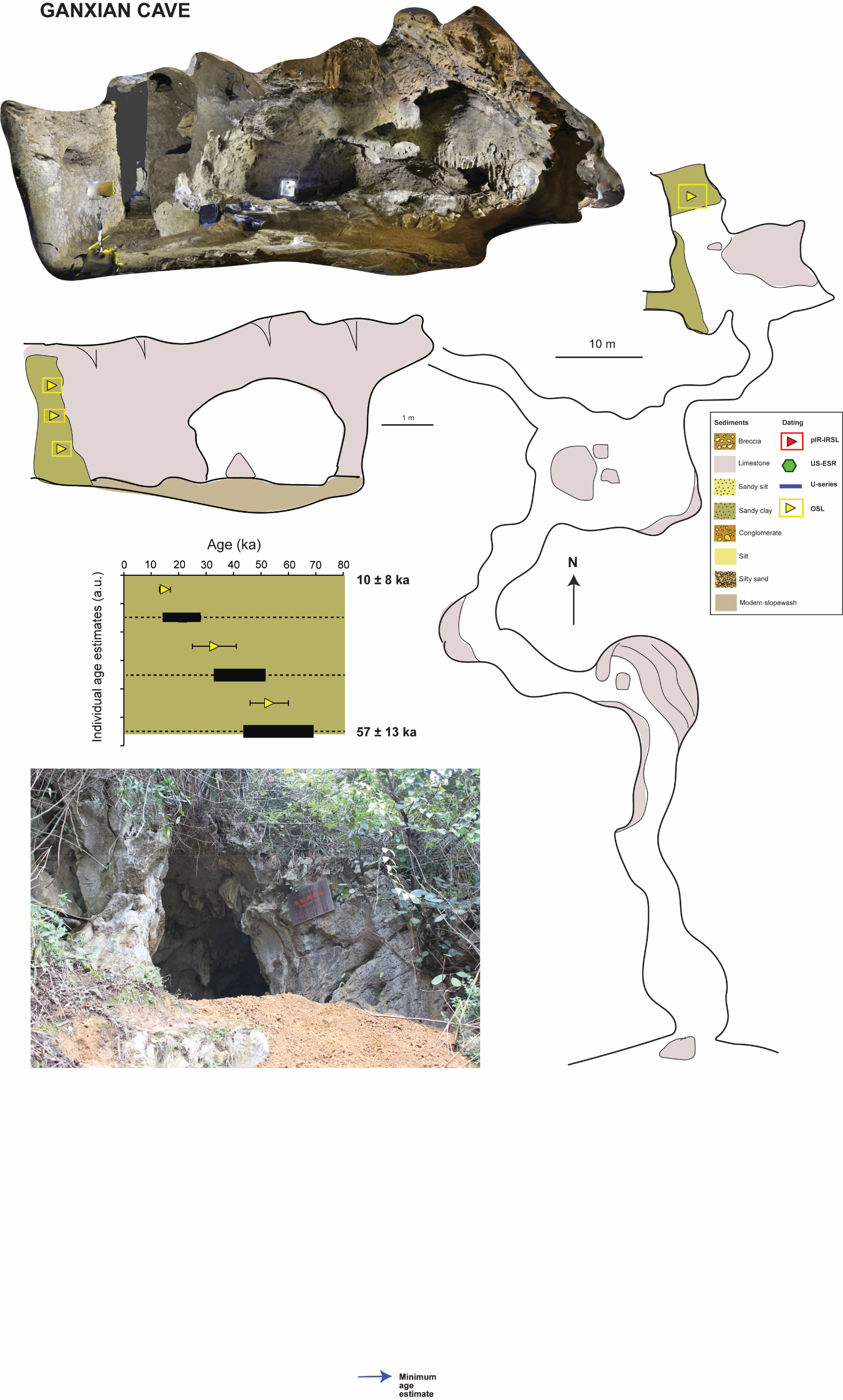
**

**Fig. S1u** – Ganxian Cave (CGAN) in Bubing Basin– plan, profile, composite stratigraphy, fossil location and dating results

**
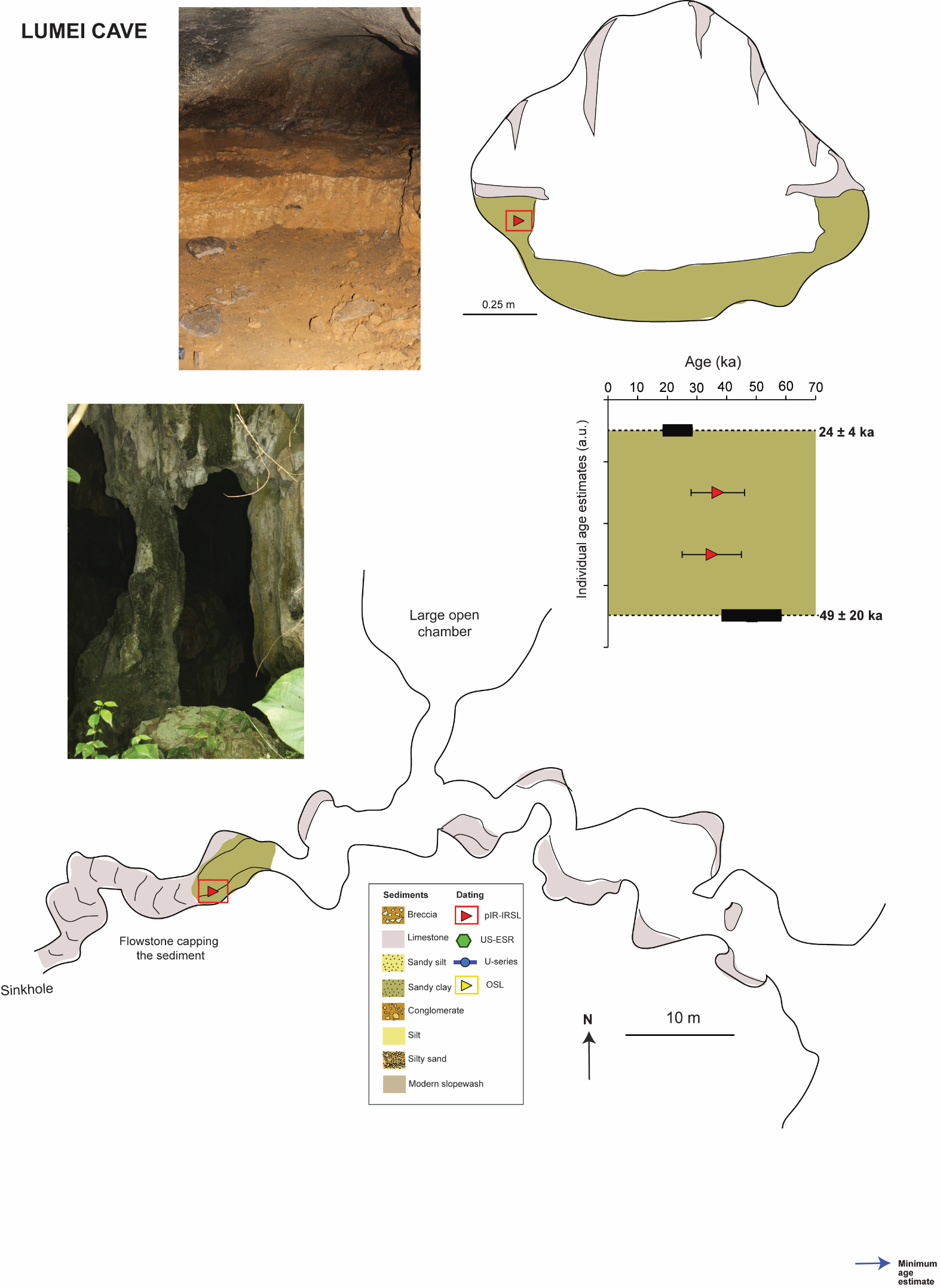
Fig. S1v** – Lumei Cave (CLUM) in Bubing Basin– plan, profile, composite stratigraphy, fossil location and dating result

**SI section 3: Fossil finding and analysis**

**1. Cave finding strategies**

Uplift plays a key role in separating the antiquity of the caves in this region, the early *G. blacki* cave sites ~1.5 Ma lie at ~200 m asl and the younger cave sites ~400-200 ka lie at an elevation of 170-180 m asl. Mulan mountain situated in the Zuojiang River area close to Chongzuo city contains 17 caves over six horizons of karst, with each horizon, based on the elevation of the main cave entrance, representing a different time period. Horizon five at ~200 m contains the earliest *G. blacki* remains, and the upper 3 horizons contain the remaining *G. blacki* evidence^3^. Horizon three, thought to be close to the extinction window, contains the *G. blacki* bearing caves of Shuangtan and Hejiang with an elevation of ~180 m absl^164^. These horizons may also extend to the nearby Queque cave in Wuming Mountain^164^. This provides a local baselevel for *G. blacki* cave exploration, although due to the localised processes of karstic evolution the horizon levels don’t extend much past the local area. For example, Yixiantian is a *G. blacki*-bearing cave situated at 160 m in the Liuqiao area, while Gongjishan is a non *G. blacki*-bearing cave situated at 170 m in the Luobai area.

Empirically, there are few caves inside peak clusters, so the possibility to find fossils is greater in caves in inselbergs or karst peaks on the brim of floodplains or along the flanks of valleys. In these places, caves form galleries accompanied by bigger chambers, where it is easy for fossil-containing clastic sediments to be deposited.

Contrary to the completely vegetated karst cones in Guizhou Province, the karst peaks in this area have unvegetated sheer sides, which makes it easy to spot cave entrances. Some of the caves in this study were found this way during field survey. However, not all caves are developed on the sheer sides of karst peaks. For some caves, their entrances are located on slopes below the sheer sides and covered by vegetation. These caves are usually found by chance on the way to higher caves on the karst peaks, or by getting information from locals who like to explore caves. For this project we built on the local knowledge of cave locations by the use of reconnaissance surveys from cars and on foot during targeted expeditions. We also teamed up an experienced group of Beijing cavers (who had been trained in fossil and breccia identification) with local caving groups and employed members to consistently search for new caves. We supplemented these expeditions with drone flybys in an attempt to spot cave entrances that weren’t visible from the ground. During the project period 2017-19 we built on the knowledge gained from the 18 known caves and discovered hundreds of new caves but only three (Zhanwang, Ma Feng and Xiaokou) were suitable for excavation.

**2. Excavation strategies**

The depositional sequence of clastic sediments in caves is quite complex. Unlike well stratified fluvio-lacustrine deposits, different depositional units of cave deposits usually have irregular boundaries and cut and fill processes are common. The stratigraphic division mainly relies on cautious observation of lithology and an appreciation of remnant deposits preserved by flowstones.

In most cases, space restrictions inside the caves and the physical characteristics of cave deposits means that the usual methods for archaeological excavation are not applicable to these specialised paleontological excavations. Excavation grids were set up based on the shape of cave passages or chambers and distribution of fossil-containing deposits. Jackhammers are necessary especially when dealing with overlying calcite-cemented deposits. Big blocks of fossil-containing deposits are broken off using jackhammers and subsequently processed by workers using geological hammers to locate and remove the fossils. Fine cleaning of fossils is usually conducted back in the laboratories.

| **Table S3** Faunal list and composition of the Baikong fauna at genus level | | | | | | | | |  |  |  |
| --- | --- | --- | --- | --- | --- | --- | --- | --- | --- | --- | --- |
| Taxa\Tooth types | I | C | P | M1/2 | M3 | i | c | p | m1/2 | m3 | subtotal |
| **Primates** | 21 | 14 | 59 | 83 | 67 | 11 | 17 | 60 | 66 | 44 | 442 |
| Cercopithecidae | 5 | 3 | 30 | 55 | 44 | 1 | 9 | 12 | 19 | 13 | 191 |
| †*Procynocephalus* | 0 | 0 | 0 | 0 | 0 | 0 | 0 | 0 | 1 | 1 | 2 |
| *Macaca* | 5 | 3 | 25 | 43 | 36 | 1 | 2 | 11 | 14 | 10 | 150 |
| *Trachypithecus* | 0 | 0 | 2 | 8 | 1 | 0 | 4 | 0 | 3 | 0 | 18 |
| *Rhinopithecus* | 0 | 0 | 3 | 4 | 7 | 0 | 3 | 1 | 1 | 2 | 21 |
| Hylobatidae | 0 | 0 | 1 | 0 | 2 | 2 | 0 | 0 | 0 | 0 | 5 |
| *Nomascus* | 0 | 0 | 1 | 0 | 2 | 2 | 0 | 0 | 0 | 0 | 5 |
| Hominidae | 16 | 11 | 28 | 28 | 21 | 8 | 8 | 48 | 47 | 31 | 246 |
| †*Gigantopithecus* | 8 | 5 | 27 | 22 | 17 | 4 | 3 | 41 | 38 | 25 | 190 |
| *Pongo* | 8 | 6 | 1 | 4 | 3 | 4 | 5 | 7 | 8 | 4 | 50 |
| †“mystery ape” | 0 | 0 | 0 | 2 | 1 | 0 | 0 | 0 | 1 | 2 | 6 |
| **Canivora** | 4 | 5 | 33 | 22 | - | 0 | 7 | 18 | 8 | 1 | 98 |
| Hyaenidae | 1 | 0 | 7 | 0 | - | 0 | 0 | 6 | 0 | - | 14 |
| †*Pachycrocuta* | 1 | 0 | 7 | 0 | - | 0 | 0 | 6 | 0 | - | 14 |
| Felidae | 2 | 3 | 13 | 0 | - | 0 | 2 | 7 | 2 | - | 29 |
| *Felis* | 0 | 0 | 4 | 0 | - | 0 | 1 | 2 | 0 | - | 7 |
| *Panthera* | 2 | 2 | 9 | 0 | - | 0 | 1 | 5 | 2 | - | 21 |
| *Neofelis* | 0 | 1 | 0 | 0 | - | 0 | 0 | 0 | 0 | - | 1 |
| Viverridae | 0 | 0 | 2 | 0 | - | 0 | 0 | 0 | 0 | - | 2 |
| *Viverra* | 0 | 0 | 1 | 0 | - | 0 | 0 | 0 | 0 | - | 1 |
| *Paguma* | 0 | 0 | 1 | 0 | - | 0 | 0 | 0 | 0 | - | 1 |
| Herpestidae | 0 | 0 | 1 | 1 | - | 0 | 0 | 0 | 0 | - | 2 |
| *Herpestes* | 0 | 0 | 1 | 1 | - | 0 | 0 | 0 | 0 | - | 2 |
| Canidae | 0 | 0 | 1 | 1 | - | 0 | 1 | 0 | 0 | - | 3 |
| *Cuon* | 0 | 0 | 1 | 1 | - | 0 | 1 | 0 | 0 | - | 3 |
| Ursidae | 1 | 1 | 3 | 10 | - | 0 | 1 | 5 | 4 | 1 | 26 |
| *Ursus* | 1 | 1 | 0 | 5 | - | 0 | 1 | 5 | 4 | 0 | 17 |
| *Ailuropoda* | 0 | 0 | 3 | 5 | - | 0 | 0 | 0 | 0 | 1 | 9 |
| Mustelidae | 0 | 1 | 6 | 10 | - | 0 | 3 | 0 | 2 | - | 22 |
| *Martes* | 0 | 0 | 0 | 2 | - | 0 | 1 | 0 | 0 | - | 3 |
| *Arctonyx* | 0 | 1 | 6 | 8 | - | 0 | 2 | 0 | 2 | - | 19 |
| **Proboscidea** | 62 | | | | | | | | | | 62 |
| †Gomophotheriidae | 18 | | | | | | | | | | 18 |
| †*Sinomastodon* | 18 | | | | | | | | | | 18 |
| †Stegodontidae | 44 | | | | | | | | | | 44 |
| †*Stegodon* | 44 | | | | | | | | | | 44 |
| **Perissodactyla** | 142 | | | | | | | | | | 142 |
| Rhinocerotidae | 89 | | | | | | | | | | 89 |
| *Rhinoceros* | 89 | | | | | | | | | | 89 |
| Tapridae | 49 | | | | | | | | | | 49 |
| *Tapirus* | 49 | | | | | | | | | | 49 |
| †Chalicotheriidae | 4 | | | | | | | | | | 4 |
| †*Hesperotherium* | 4 | | | | | | | | | | 4 |
| **Artiodactyla** | 10 | 13 | 235 | 312 | 145 | 50 | 29 | 371 | 328 | 176 | 1669 |
| Suidae | 10 | 13 | 170 | 156 | 129 | 48 | 29 | 261 | 158 | 103 | 1077 |
| †*Hippopotamodon* | 0 | 0 | 14 | 17 | 37 | 0 | 0 | 19 | 5 | 16 | 108 |
| *Sus* | 10 | 13 | 156 | 139 | 92 | 48 | 29 | 242 | 153 | 87 | 969 |
| *Sus peii* | 10 | 13 | 151 | 110 | 77 | 47 | 29 | 242 | 127 | 81 | 887 |
| *Sus xiaozhu* | 0 | 0 | 5 | 29 | 15 | 1 | 0 | 0 | 26 | 6 | 82 |
| Moschidae | - | 0 | 0 | 2 | 0 | 0 | 0 | 1 | 2 | 0 | 5 |
| *Moschus* | - | 0 | 0 | 2 | 0 | 0 | 0 | 1 | 2 | 0 | 5 |
| Cervidae | - | 0 | 56 | 88 | 5 | 1 | 0 | 54 | 31 | 12 | 247 |
| *Cervus* | - | - | 19 | 49 | 2 | 1 | 0 | 37 | 30 | 8 | 146 |
| *Muntiacus* | - | 0 | 20 | 33 | 3 | 0 | 0 | 8 | 1 | 2 | 67 |
| *Elaphodus* | - | 0 | 17 | 6 | 0 | 0 | 0 | 9 | 0 | 2 | 34 |
| Bovidae | - | - | 9 | 66 | 11 | 1 | 0 | 55 | 137 | 61 | 340 |
| *Bos* | - | - | 4 | 28 | 1 | 1 | 0 | 31 | 78 | 36 | 179 |
| *Capricornis* | - | - | 3 | 28 | 4 | 0 | 0 | 12 | 31 | 17 | 95 |
| †*Megalovis* | - | - | 2 | 10 | 6 | 0 | 0 | 12 | 28 | 8 | 66 |
| **Rodentia** | 0 | - | 1 | 64 | 16 | 0 | - | 25 | 56 | 17 | 179 |
| Hystricidae | 0 | - | 1 | 64 | 16 | 0 | - | 25 | 56 | 17 | 179 |
| *Atherurus* | 0 | - | 0 | 8 | 2 | 0 | - | 1 | 7 | 4 | 22 |
| *Hystrix* | 0 | - | 1 | 56 | 14 | 0 | - | 24 | 49 | 13 | 157 |
| Total |  |  |  |  |  |  |  |  |  |  | 2592 |
| Note: † indicates fossil genus | |  |  |  |  |  |  |  |  |  |  |

All the fossils are catalogued based on dates and grid numbers, so they can be securely related to plan and section drawings and traced back to their original strata.

**3. Faunal analysis methods**

Three established mammalian faunas were selected as chronological planes of reference to investigate the extinction of *Gigantopithecus blacki*. The Early Pleistocene Baikong fauna (Table S3, ED7) represented by 2,592 identifiable specimens, which lived in a period of time when *Gigantopithecus blacki* thrived. The Yixiantian fauna (Table S4, ED7) represented by 4,815 identifiable specimens with a Middle Pleistocence age and the Baxian fauna (Table S5, ED7) represented by 11,796 identifiable specimens with a late Middle Pleistocene in age sandwiching the extinction window of *Gigantopithecus blacki* with the former prior to and the latter after its extinction. They represent the time period during which the giant ape perished. The specimens of the three faunas are mostly made up of isolated teeth. To avoid subjectivity, all the teeth were identified to genus level and to different tooth types.

The extant mammalian fauna of Guangxi Zhuang Autonomous Region is also summarized (Table S6) to reconstruct the habitats for the fossil faunas based on their common genera. The IUCN habitats classification scheme (Ver. 3.1) is adopted here to categorize different habitat types (Table S7). The

**Table S4** Faunal list and composition of the Yixiantian fauna at the genus level

| Taxa\Tooth types | I | C | P | M1/2 | M3 | i | c | p | m1/2 | m3 | subtotal |
| --- | --- | --- | --- | --- | --- | --- | --- | --- | --- | --- | --- |
| **Primates** | 21 | 33 | 94 | 173 | 69 | 10 | 38 | 76 | 74 | 28 | 616 |
| Cercopithecidae | 0 | 24 | 64 | 121 | 63 | 0 | 29 | 42 | 46 | 22 | 411 |
| *Macaca* | 0 | 20 | 56 | 59 | 33 | 0 | 19 | 32 | 34 | 10 | 263 |
| *Macaca* sp. | 0 | 20 | 56 | 59 | 33 | 0 | 19 | 32 | 34 | 10 | 263 |
| *Pygathrix* | 0 | 0 | 2 | 20 | 4 | 0 | 0 | 0 | 0 | 0 | 26 |
| *Pygathrix* sp. | 0 | 0 | 2 | 20 | 4 | 0 | 0 | 0 | 0 | 0 | 26 |
| *Trachypithecus* | 0 | 2 | 6 | 23 | 6 | 0 | 5 | 8 | 6 | 1 | 57 |
| *Trachypithecus* sp. | 0 | 2 | 6 | 23 | 6 | 0 | 5 | 8 | 6 | 1 | 57 |
| *Rhinopithecus* | 0 | 2 | 0 | 19 | 20 | 0 | 5 | 2 | 6 | 11 | 65 |
| *Rhinopithecus* sp. | 0 | 2 | 0 | 19 | 20 | 0 | 5 | 2 | 6 | 11 | 65 |
| Hylobatidae | 7 | 1 | 2 | 9 | 1 | 1 | 0 | 0 | 2 | 0 | 23 |
| *Nomascus* | 7 | 1 | 2 | 9 | 1 | 1 | 0 | 0 | 2 | 0 | 23 |
| *Nomascus* sp. | 7 | 1 | 2 | 9 | 1 | 1 | 0 | 0 | 2 | 0 | 23 |
| Hominidae | 14 | 8 | 28 | 43 | 5 | 9 | 9 | 34 | 26 | 6 | 182 |
| †*Gigantopithecus* | 0 | 0 | 0 | 0 | 0 | 0 | 0 | 0 | 1 | 0 | 1 |
| *Gigantopithecus blacki* | 0 | 0 | 0 | 0 | 0 | 0 | 0 | 0 | 1 | 0 | 1 |
| *Pongo* | 14 | 8 | 28 | 43 | 5 | 9 | 9 | 34 | 25 | 6 | 181 |
| *Pongo weidenreichi* | 14 | 8 | 28 | 43 | 5 | 9 | 9 | 34 | 25 | 6 | 181 |
| **Canivora** | 10 | 30 | 90 | 80 | - | 8 | 30 | 34 | 55 | - | 337 |
| Felidae | 1 | 7 | 28 | 0 | - | 0 | 7 | 9 | 6 | - | 58 |
| *Felis* | 1 | 2 | 9 | 0 | - | 0 | 2 | 0 | 0 | - | 14 |
| *Felis chaus* | 1 | 2 | 9 | 0 | - | 0 | 2 | 0 | 0 | - | 14 |
| *Catopuma* | 0 | 1 | 7 | 0 | - | 0 | 0 | 0 | 0 | - | 8 |
| *Catopuma temminckii* | 0 | 1 | 7 | 0 | - | 0 | 0 | 0 | 0 | - | 8 |
| *Panthera* | 0 | 2 | 8 | 0 | - | 0 | 2 | 4 | 5 | - | 21 |
| *Panthera tigris* | 0 | 2 | 6 | 0 | - | 0 | 2 | 2 | 3 | - | 15 |
| *Panthera pardus* | 0 | 0 | 2 | 0 | - | 0 | 0 | 2 | 2 | - | 6 |
| *Neofelis* | 0 | 2 | 4 | 0 | - | 0 | 3 | 5 | 1 | - | 15 |
| *Neofelis nebulosa* | 0 | 2 | 4 | 0 | - | 0 | 3 | 5 | 1 | - | 15 |
| Viverridae | 1 | 1 | 18 | 4 | - | 0 | 1 | 11 | 3 | - | 39 |
| *Viverra* | 1 | 1 | 8 | 3 | - | 0 | 1 | 10 | 2 | - | 26 |
| *Viverra* sp. | 1 | 1 | 8 | 3 | - | 0 | 1 | 10 | 2 | - | 26 |
| *Paguma* | 0 | 0 | 10 | 1 | - | 0 | 0 | 1 | 1 | - | 13 |
| *Paguma larvata* | 0 | 0 | 10 | 1 | - | 0 | 0 | 1 | 1 | - | 13 |
| Herpestidae | 0 | 0 | 0 | 0 | - | 0 | 1 | 1 | 1 | - | 3 |
| *Herpestes* | 0 | 0 | 0 | 0 | - | 0 | 1 | 1 | 1 | - | 3 |
| *Herpestes* sp. | 0 | 0 | 0 | 0 | - | 0 | 1 | 1 | 1 | - | 3 |
| Canidae | 0 | 0 | 2 | 5 | - | 0 | 0 | 0 | 2 | - | 9 |
| *Cuon* | 0 | 0 | 2 | 5 | - | 0 | 0 | 0 | 2 | - | 9 |
| *Cuon alpinus* | 0 | 0 | 2 | 5 | - | 0 | 0 | 0 | 2 | - | 9 |
| Ursidae | 5 | 18 | 25 | 35 | - | 8 | 13 | 8 | 28 | 0 | 140 |
| *Ursus* | 3 | 13 | 15 | 25 | - | 8 | 10 | 0 | 19 | 0 | 93 |
| *Ursus thibetanus* | 2 | 13 | 11 | 21 | - | 8 | 9 | 0 | 13 | 0 | 77 |
| *Ursus malayanus* | 1 | 0 | 4 | 4 | - | 0 | 1 | 0 | 6 | 0 | 16 |
| *Ailuropoda* | 2 | 5 | 10 | 10 | - | 0 | 3 | 8 | 9 | 0 | 47 |
| *Ailuropoda melanoleuca baconi* | 2 | 5 | 10 | 10 | - | 0 | 3 | 8 | 9 | 0 | 47 |
| Mustelidae | 3 | 4 | 17 | 36 | - | 0 | 8 | 5 | 15 | - | 88 |
| *Melogale* | 0 | 0 | 0 | 0 | - | 0 | 0 | 0 | 1 | - | 1 |
| *Melogale moschata* | 0 | 0 | 0 | 0 | - | 0 | 0 | 0 | 1 | - | 1 |
| *Arctonyx* | 3 | 4 | 16 | 36 | - | 0 | 8 | 5 | 14 | - | 86 |
| *Arctonyx collaris* | 3 | 4 | 16 | 36 | - | 0 | 8 | 5 | 14 | - | 86 |
| *Lutra* | 0 | 0 | 1 | 0 | - | 0 | 0 | 0 | 0 | - | 1 |
| *Lutra lutra* | 0 | 0 | 1 | 0 | - | 0 | 0 | 0 | 0 | - | 1 |
| **Proboscidea** | 19* | | | | | | | | | | 19 |
| †Stegodontidae | 13* | | | | | | | | | | 13 |
| †*Stegodon* | 13* | | | | | | | | | | 13 |
| *Stegodon orientalis* | 13* | | | | | | | | | | 13 |
| Elephantidae | 6* | | | | | | | | | | 6 |
| *Elephas* | 6* | | | | | | | | | | 6 |
| *Elephas maximus* | 6* | | | | | | | | | | 6 |
| **Perissodactyla** | 73* | | | | | | | | | | 73 |
| Rhinocerotidae | 59* | | | | | | | | | | 59 |
| *Rhinoceros* | 59* | | | | | | | | | | 59 |
| *Rhinoceros sondaicus* | 59* | | | | | | | | | | 59 |
| Tapridae | 3 | 1 | 1 | 2 | 0 | 1 | 1 | 3 | 2 | 0 | 14 |
| †*Megatapirus* | 3 | 1 | 1 | 2 | 0 | 1 | 1 | 3 | 2 | 0 | 14 |
| †*Megatapirus augustus* | 3 | 1 | 1 | 2 | 0 | 1 | 1 | 3 | 2 | 0 | 14 |
| **Artiodactyla** | 30 | 9 | 1082 | 370 | 166 | 120 | 65 | 859 | 313 | 214 | 3228 |
| Suidae | 30 | 9 | 353 | 155 | 83 | 107 | 59 | 522 | 198 | 96 | 1612 |
| *Sus* | 30 | 9 | 353 | 155 | 83 | 107 | 59 | 522 | 198 | 96 | 1612 |
| *Sus xiaozhu* | 1 | 0 | 0 | 1 | 3 | 5 | 0 | 5 | 0 | 0 | 15 |
| *Sus peii* | 29 | 9 | 353 | 154 | 80 | 102 | 59 | 517 | 198 | 96 | 1597 |
| Cervidae | - | 0 | 157 | 168 | 59 | 9 | 5 | 188 | 42 | 67 | 695 |
| *Cervus* | - | - | 24 | 26 | 5 | 9 | 5 | 70 | 42 | 25 | 206 |
| *Cervus* (*Rusa*) *unicolor* | - | - | 24 | 26 | 5 | 9 | 5 | 70 | 42 | 25 | 206 |
| *Muntiacus* | - | 0 | 58 | 96 | 25 | 0 | 0 | 51 | 0 | 28 | 258 |
| *Muntiacus muntjak* | - | 0 | 58 | 69 | 16 | 0 | 0 | 46 | 0 | 28 | 217 |
| *Muntiacus reevisi* | - | 0 | 0 | 27 | 9 | 0 | 0 | 5 | 0 | 0 | 41 |
| *Elaphodus* | - | 0 | 75 | 46 | 29 | 0 | 0 | 67 | 0 | 14 | 231 |
| *Elaphodus cephalophus* | - | 0 | 75 | 46 | 29 | 0 | 0 | 67 | 0 | 14 | 231 |
| Bovidae | - | - | 572 | 47 | 24 | 4 | 1 | 149 | 73 | 51 | 921 |
| *Bos* | - | - | 512 | 6 | 2 | 4 | 1 | 23 | 14 | 14 | 576 |
| *Bos* (*Bibos*) *gaurus* | - | - | 512 | 6 | 2 | 4 | 1 | 23 | 14 | 14 | 576 |
| *Capricornis* | - | - | 60 | 39 | 22 | 0 | 0 | 126 | 56 | 36 | 339 |
| *Capricornis sumatraensis* | - | - | 60 | 39 | 22 | 0 | 0 | 126 | 56 | 36 | 339 |
| †*Megalovis* | - | - | 0 | 2 | 0 | 0 | 0 | 0 | 3 | 1 | 6 |
| *Megalovis guangxiensis* | - | - | 0 | 2 | 0 | 0 | 0 | 0 | 3 | 1 | 6 |
| **Rodentia** | 27 | - | 12 | 220 | 27 | 42 | - | 56 | 126 | 32 | 542 |
| Hystricidae | 27 | - | 12 | 220 | 27 | 42 | - | 56 | 126 | 32 | 542 |
| *Atherurus* | 0 | - | 0 | 74 | 10 | 0 | - | 18 | 38 | 4 | 144 |
| *Atherurus macrourus* | 0 | - | 0 | 74 | 10 | 0 | - | 18 | 38 | 4 | 144 |
| *Hystrix* | 27 | - | 12 | 146 | 17 | 42 | - | 38 | 88 | 28 | 398 |
| *Hystrix subscristata* | 27 | - | 12 | 146 | 17 | 42 | - | 38 | 88 | 28 | 398 |
| Total |  |  |  |  |  |  |  |  |  |  | 4815 |
| Note: † indicates fossil genus  Reproduced from^165^ |  |  |  |  |  |  |  |  |  |  |  |

habitat data for extant species on the IUCN red list website (https://www.iucnredlist.org/) are used to represent the preferred habitats of the common genera in the fossil faunas. Only the habitats that are marked as suitable on the website are taken into consideration, and the habitats that are marked as marginal and the artificial habitats are all ignored. The habitats of the fossil genera, such as *Procynocephalus*, *Pachycrocuta*, *Sinomastodon*, and *Stegodon* etc., are cited from published literatures (Fig. S3).

**Table S5** Faunal list and composition of the Baxian fauna at the genus level

| Taxa\Tooth types | I | C | P | M1/2 | M3 | i | c | p | m1/2 | m3 | subtotal |
| --- | --- | --- | --- | --- | --- | --- | --- | --- | --- | --- | --- |
| **Primates** | 63 | 71 | 214 | 427 | 302 | 50 | 109 | 119 | 218 | 136 | 1709 |
| Cercopithecidae | 45 | 56 | 159 | 343 | 270 | 20 | 90 | 76 | 168 | 114 | 1341 |
| *Macaca* | 43 | 45 | 153 | 239 | 203 | 18 | 66 | 58 | 100 | 80 | 1005 |
| *Pygathrix* | 0 | 0 | 6 | 27 | 12 | 0 | 0 | 0 | 12 | 1 | 58 |
| *Trachypithecus* | 2 | 9 | 0 | 66 | 40 | 2 | 19 | 16 | 30 | 12 | 196 |
| *Rhinopithecus* | 0 | 2 | 0 | 11 | 15 | 0 | 5 | 2 | 26 | 21 | 82 |
| Hylobatidae | 9 | 0 | 0 | 0 | 0 | 1 | 0 | 0 | 1 | 2 | 13 |
| *Nomascus* | 9 | 0 | 0 | 0 | 0 | 1 | 0 | 0 | 1 | 2 | 13 |
| Hominidae | 9 | 15 | 55 | 84 | 32 | 29 | 19 | 43 | 49 | 20 | 355 |
| *Pongo* | 9 | 15 | 54 | 82 | 32 | 28 | 19 | 42 | 49 | 20 | 350 |
| *Homo* | 0 | 0 | 1 | 2 | 0 | 1 | 0 | 1 | 0 | 0 | 5 |
| **Canivora** | 31 | 64 | 120 | 306 | - | 12 | 113 | 51 | 198 | 37 | 932 |
| Hyaenidae | 0 | 0 | 1 | 0 | - | 0 | 0 | 0 | 0 | - | 1 |
| *Crocuta* | 0 | 0 | 1 | 0 | - | 0 | 0 | 0 | 0 | - | 1 |
| Felidae | 5 | 18 | 24 | 0 | - | 5 | 9 | 9 | 4 | - | 74 |
| *Felis* | 1 | 2 | 19 | 0 | - | 4 | 1 | 4 | 1 | - | 32 |
| *Catopuma* | 0 | 3 | 0 | 0 | - | 0 | 1 | 0 | 0 | - | 4 |
| *Panthera* | 4 | 1 | 4 | 0 | - | 1 | 3 | 5 | 3 | - | 21 |
| *Neofelis* | 0 | 5 | 0 | 0 | - | 0 | 0 | 0 | 0 | - | 5 |
| *Prionailurus* | 0 | 7 | 1 | 0 | - | 0 | 4 | 0 | 0 | - | 12 |
| Viverridae | 0 | 0 | 6 | 8 | - | 0 | 0 | 2 | 3 | - | 19 |
| *Viverra* | 0 | 0 | 4 | 4 | - | 0 | 0 | 2 | 2 | - | 12 |
| *Paguma* | 0 | 0 | 2 | 4 | - | 0 | 0 | 0 | 0 | - | 6 |
| *Chrotogale* | 0 | 0 | 0 | 0 | - | 0 | 0 | 0 | 1 | - | 1 |
| Herpestidae | 0 | 3 | 5 | 2 | - | 0 | 1 | 4 | 4 | - | 19 |
| *Herpestes* | 0 | 3 | 5 | 2 | - | 0 | 1 | 4 | 4 | - | 19 |
| Canidae | 0 | 2 | 0 | 5 | - | 0 | 0 | 0 | 3 | - | 10 |
| *Cuon* | 0 | 2 | 0 | 5 | - | 0 | 0 | 0 | 3 | - | 10 |
| Ursidae | 26 | 30 | 45 | 131 | - | 7 | 86 | 36 | 98 | 37 | 496 |
| *Ursus* | 26 | 20 | 32 | 111 | - | 7 | 82 | 4 | 74 | 37 | 393 |
| *Ursus thibetanus* | 25 | 20 | 30 | 108 | - | 7 | 80 | 4 | 70 | 35 | 379 |
| *Ursus malayanus* | 1 | 0 | 2 | 3 | - | 0 | 2 | 0 | 4 | 2 | 14 |
| *Ailuropoda* | 0 | 10 | 13 | 20 | - | 0 | 4 | 32 | 24 | 0 | 103 |
| Mustelidae | 0 | 11 | 39 | 160 | - | 0 | 17 | 0 | 86 | - | 313 |
| *Martes* | 0 | 0 | 0 | 0 | - | 0 | 0 | 0 | 1 | - | 1 |
| *Melogale* | 0 | 0 | 2 | 0 | - | 0 | 0 | 0 | 2 | - | 4 |
| *Arctonyx* | 0 | 11 | 32 | 158 | - | 0 | 17 | 0 | 83 | - | 301 |
| *Mustela* | 0 | 0 | 5 | 0 | - | 0 | 0 | 0 | 0 | - | 5 |
| *Lutra* | 0 | 0 | 0 | 2 | - | 0 | 0 | 0 | 0 | - | 2 |
| **Proboscidea** | 224 | | | | | | | | | | 224 |
| †Stegodontidae | 45 | | | | | | | | | | 45 |
| †*Stegodon* | 45 | | | | | | | | | | 45 |
| Elephantidae | 179 | | | | | | | | | | 179 |
| *Elephas* | 179 | | | | | | | | | | 179 |
| **Perissodactyla** | 121 | | | | | | | | | | 121 |
| Rhinocerotidae | 101 | | | | | | | | | | 101 |
| *Rhinoceros* | 101 | | | | | | | | | | 101 |
| Tapridae | 20 | | | | | | | | | | 20 |
| †*Megatapirus* | 20 | | | | | | | | | | 20 |
| **Artiodactyla** | 54 | 32 | 1411 | 1510 | 460 | 319 | 63 | 1250 | 1202 | 592 | 6893 |
| Suidae | 54 | 32 | 672 | 499 | 297 | 279 | 57 | 570 | 639 | 297 | 3396 |
| *Sus* | 54 | 32 | 672 | 499 | 297 | 279 | 57 | 570 | 639 | 297 | 3396 |
| Moschidae | - | - | 0 | 0 | 0 | 0 | 0 | 0 | 0 | 1 | 1 |
| *Moschus* | - | - | 0 | 0 | 0 | 0 | 0 | 0 | 0 | 1 | 1 |
| Cervidae | - | - | 537 | 822 | 84 | 18 | 4 | 390 | 226 | 188 | 2269 |
| *Cervus* | - | - | 147 | 208 | 12 | 18 | 4 | 234 | 226 | 71 | 920 |
| *Muntiacus* | - | - | 182 | 312 | 38 | 0 | 0 | 65 | 0 | 32 | 629 |
| *Muntiacus muntjak* | - | - | 171 | 233 | 33 | 0 | 0 | 65 | 0 | 32 | 534 |
| *Muntiacus reevesi* | - | - | 11 | 79 | 5 | 0 | 0 | 0 | 0 | 0 | 95 |
| *Elaphodus* | - | - | 208 | 302 | 34 | 0 | 0 | 91 | 0 | 85 | 720 |
| Bovidae | - | - | 202 | 189 | 79 | 22 | 2 | 290 | 337 | 106 | 1227 |
| *Bos* | - | - | 7 | 33 | 3 | 5 | 2 | 87 | 93 | 25 | 255 |
| *Capricornis* | - | - | 195 | 156 | 76 | 17 | 0 | 203 | 244 | 81 | 972 |
| **Rodentia** | 161 | - | 159 | 589 | 163 | 134 | - | 182 | 412 | 117 | 1917 |
| Hystricidae | 161 | - | 159 | 589 | 163 | 134 | - | 182 | 412 | 117 | 1917 |
| *Atherurus* | 0 | - | 23 | 11 | 4 | 0 | - | 9 | 12 | 0 | 59 |
| *Hystrix* | 161 | - | 136 | 578 | 159 | 134 | - | 173 | 400 | 117 | 1858 |
| Total |  |  |  |  |  |  |  |  |  |  | 11796 |
| Note: † indicates fossil genus |  |  |  |  |  |  |  |  |  |  |  |

**Table S6** Compositional comparisons among the Baikong, Yixiantian, Baxian and extant faunas

| Taxa | Common name | Baikong | Yixiantian | Baxian | Extant |
| --- | --- | --- | --- | --- | --- |
| **Eulipotyphla** |  |  |  |  | √ |
| Soricidae |  |  |  |  | √ |
| *Suncus* | House Shrew |  |  |  | √ |
| *Crocidura* | White-toothed Shrew |  |  |  | √ |
| *Anourosorex* | Mole Shrew |  |  |  | √ |
| *Chimmarogale* | Water Shrew |  |  |  | √ |
| Talpidae |  |  |  |  | √ |
| *Euroscaptor* | Long-nosed Mole |  |  |  | √ |
| *Mogera* | Insular Mole |  |  |  | √ |
| *Parascaptor* | White-tailed Mole |  |  |  | √ |
| **Scandentia** |  |  |  |  | √ |
| Tupaiidae |  |  |  |  | √ |
| *Tupaia* | Treeshrew |  |  |  | √ |
| **Chiroptera** |  |  |  |  | √ |
| Pteropodidae |  |  |  |  | √ |
| *Rousettus* | Rousette |  |  |  | √ |
| *Cynopterus* | Short-nosed Fruit Bat |  |  |  | √ |
| *Eonycteris* | Dawn Bat |  |  |  | √ |
| Rhinolophidae |  |  |  |  | √ |
| *Rhinolophus* | Horseshoe Bat |  |  |  | √ |
| Hipposideridae |  |  |  |  | √ |
| *Hipposideros* | Leaf-nosed Bat |  |  |  | √ |
| *Aselliscus* | Trident Bat |  |  |  | √ |
| *Coelops* | Tailless Leaf-nosed Bat |  |  |  | √ |
| Megadermatidae |  |  |  |  | √ |
| *Megaderma* | False Vampire |  |  |  | √ |
| Emballonuridae |  |  |  |  | √ |
| *Taphozous* | Tomb Bat |  |  |  | √ |
| Molossidae |  |  |  |  | √ |
| *Chaerephon* | Free-tailed Bat |  |  |  | √ |
| *Tadarida* | Free-tailed Bat |  |  |  | √ |
| Vespertilionidae |  |  |  |  | √ |
| *Pipistrellus* | Pipistrelle |  |  |  | √ |
| *Falsistrellus* | Pipistrelle |  |  |  | √ |
| *Ia* | Great Evening Bat |  |  |  | √ |
| *Vespertilio* | Particolored Bat |  |  |  | √ |
| *Nyctalus* | Noctule |  |  |  | √ |
| *Tylonycteris* | Bamboo Bat |  |  |  | √ |
| *Scotomanes* | Harlequin Bat |  |  |  | √ |
| *Scotophilus* | House Bat |  |  |  | √ |
| *Myotis* | Mouse-eared Bat |  |  |  | √ |
| *Miniopterus* | Long-fingered Bat |  |  |  | √ |
| *Murina* | Tube-nosed Bat |  |  |  | √ |
| *Kerivoula* | Woolly Bat |  |  |  | √ |
| **Primates** |  | √ | √ | √ | √ |
| Lorisidae |  |  |  |  | √ |
| *Nycticebus* | Slow Loris |  |  |  | √ |
| Cercopithecidae |  | √ | √ | √ | √ |
| †*Procynocephalus* | Prior Yellow Baboon | √ |  |  |  |
| *Macaca* | Macaque | √ | √ | √ | √ |
| *Pygathrix* | Douc Langur |  | √ | √ |  |
| *Trachypithecus* | Langur | √ | √ | √ | √ |
| *Rhinopithecus* | Snub-nosed Monkey | √ | √ | √ |  |
| Hylobatidae |  | √ | √ | √ | √ |
| *Nomascus* | Gibbon | √ | √ | √ | √ |
| Hominidae |  | √ | √ | √ | √ |
| †*Gigantopithecus* |  | √ | √ |  |  |
| *Pongo* | Orangutan | √ | √ | √ |  |
| *Homo* | Human |  |  | √ | √ |
| †“mystery ape” |  | √ |  |  |  |
| **Pholidota** |  |  |  |  | √ |
| Manidae |  |  |  |  | √ |
| *Manis* | Pangolin |  |  |  | √ |
| **Canivora** |  | √ | √ | √ | √ |
| Hyaenidae |  | √ |  | √ |  |
| †*Pachycrocuta* | Giant Hyaena | √ |  |  |  |
| *Crocuta* | Spotted Hyaena |  |  | √ |  |
| Felidae |  | √ | √ | √ |  |
| *Felis* | Cat | √ | √ | √ |  |
| *Catopuma* | Golden Cat |  | √ | √ | √ |
| *Panthera* | Tiger, Jaguar, Leopard, Lion | √ | √ | √ | √ |
| *Neofelis* | Clouded Leopard | √ | √ | √ | √ |
| *Prionailurus* | Leopard Cat, Fishing Cat |  |  | √ | √ |
| Viverridae |  | √ | √ | √ |  |
| *Cynogale* | Otter Civet |  |  |  | √ |
| *Paradoxurus* | Palm Civet |  |  |  | √ |
| *Viverra* | Civet | √ | √ | √ | √ |
| *Viverricula* | Small Indian Civet |  |  |  | √ |
| *Paguma* | Masked Palm Civet | √ | √ | √ | √ |
| *Arctictis* | Binturong |  |  |  | √ |
| *Chrotogale* | Owston's Civet |  |  | √ | √ |
| *Prionodon* | Linsang |  |  |  | √ |
| Herpestidae |  | √ | √ | √ | √ |
| *Herpestes* | Mongoose | √ | √ | √ | √ |
| Canidae |  | √ | √ | √ | √ |
| *Canis* | Wolf |  |  |  | √ |
| *Vulpes* | Fox |  |  |  | √ |
| *Nyctereutes* | Raccoon Dog |  |  |  | √ |
| *Cuon* | Dhole | √ | √ | √ | √ |
| Ursidae |  | √ | √ | √ | √ |
| *Ursus* (including *Helarctos*) | Bear | √ | √ | √ | √ |
| *Ailuropoda* | Giant Panda | √ | √ | √ |  |
| Mustelidae |  | √ | √ | √ | √ |
| *Martes* | Marten | √ |  | √ | √ |
| *Melogale* | Ferret Badger |  | √ | √ | √ |
| *Meles* | Badger |  |  |  | √ |
| *Arctonyx* | Hog Badger | √ | √ | √ | √ |
| *Mustela* | Weasel |  |  | √ | √ |
| *Lutra* | Otter |  | √ | √ | √ |
| *Aonyx* | Small-clawed Otter |  |  |  | √ |
| **Proboscidea** |  | √ | √ | √ |  |
| †Gomophotheriidae |  | √ |  |  |  |
| †*Sinomastodon* | Chinese Mastodont | √ |  |  |  |
| †Stegodontidae |  | √ | √ | √ |  |
| †*Stegodon* |  | √ | √ | √ |  |
| Elephantidae |  |  | √ | √ |  |
| *Elephas* | Asian Elephant |  | √ | √ |  |
| **Perissodactyla** |  | √ | √ | √ |  |
| Rhinocerotidae |  | √ | √ | √ |  |
| *Rhinoceros* | Rhino | √ | √ | √ |  |
| Tapridae |  | √ | √ | √ |  |
| *Tapirus* | Tapir | √ |  |  |  |
| †*Megatapirus* | Giant Tapir |  | √ | √ |  |
| †Chalicotheriidae |  | √ |  |  |  |
| †*Hesperotherium* |  | √ |  |  |  |
| **Artiodactyla** |  | √ |  | √ | √ |
| Suidae |  | √ |  | √ | √ |
| †*Hippopotamodon* |  | √ |  |  |  |
| *Sus* | Wild Boar | √ | √ | √ | √ |
| Moschidae |  | √ |  | √ | √ |
| *Moschus* | Musk Deer | √ |  | √ | √ |
| Cervidae |  | √ | √ | √ | √ |
| *Cervus* | Deer | √ | √ | √ | √ |
| *Muntiacus* | Muntjac | √ | √ | √ | √ |
| *Elaphodus* | Tufted Deer | √ | √ | √ | √ |
| Bovidae |  | √ | √ | √ | √ |
| *Bos* | Gaur | √ | √ | √ |  |
| *Naemorhedus* | Goral |  |  |  | √ |
| *Capricornis* | Serow | √ | √ | √ | √ |
| †*Megalovis* |  | √ | √ |  |  |
| **Rodentia** |  | √ | √ | √ | √ |
| Sciuridae |  |  |  |  | √ |
| *Ratufa* | Giant Squirrel |  |  |  | √ |
| *Belomys* | Hairy-footed Flying Squirrel |  |  |  | √ |
| *Hylopetes* | Flying Squirrel |  |  |  | √ |
| *Petaurista* | Giant Flying Squirrel |  |  |  | √ |
| *Trogopterus* | Complex-toothed Flying Squirrel |  |  |  | √ |
| *Callosciurus* | Pallas's Squirrel |  |  |  | √ |
| *Dremomys* | Long-nosed Squirrel |  |  |  | √ |
| *Tamiops* | Striped Squirrel |  |  |  | √ |
| *Sciurotamias* | Rock Squirrel |  |  |  | √ |
| Platacanthomyidae |  |  |  |  | √ |
| *Typhlomys* | Tree Mouse |  |  |  | √ |
| Spalacidae |  |  |  |  | √ |
| *Rhizomys* | Bamboo Rat |  |  |  | √ |
| Cricetidae |  |  |  |  | √ |
| *Eothenomys* | Red-backed Vole |  |  |  | √ |
| *Microtus* | Reed Vole |  |  |  | √ |
| Muridae |  |  |  |  | √ |
| *Hapalomys* | Marmoset Rat |  |  |  | √ |
| *Chiropodomys* | Pencil-tailed Tree Mouse |  |  |  | √ |
| *Micromys* | Harvest Mouse |  |  |  | √ |
| *Apodemus* | Field Mouse |  |  |  | √ |
| *Rattus* | House Rat |  |  |  | √ |
| *Niviventer* | Niviventer |  |  |  | √ |
| *Berylmys* | White-toothed Rat |  |  |  | √ |
| *Leopoldamys* | Long-tailed Giant Rat |  |  |  | √ |
| *Mus* | Mouse |  |  |  | √ |
| *Bandicota* | Bandicoot Rat |  |  |  | √ |
| Hystricidae |  | √ | √ | √ | √ |
| *Atherurus* | Brush-tailed Porcupine | √ | √ | √ | √ |
| *Hystrix* | Porcupine | √ | √ | √ | √ |
| **Lagomorpha** |  |  |  |  | √ |
| Leporidae |  |  |  |  | √ |
| *Lepus* | Hare |  |  |  | √ |

Notes: † indicates fossil genus; Elipotyphla, Scandentia, Chiroptera, and most Rodentia are absent from the fossil faunas due to sampling limitations.

**Table S7:** Habitat types of extant species and fossil genera in Guangxi Zhuang Autonomous Region (on a separate excel file).

**4. Discussion**

*Gigantopithecus blacki* is a common element in the primate communities of the Early Pleistocene Baikong fauna. It represents 41.6% of all Primates and is 3.6 times larger than the *P. weidenreichi* population. The Middle Pleistocene Yixiantian fauna has only one specimen of *Gigantopithecus blacki*, vastly outnumbered by the coexisting *P. weidenreichi*. But it completely disappears in the late Middle Pleistocene Baxian fauna (Fig. S2). The three faunas represent three distinct stages in the evolutionary history of *Gigantopithecus blacki*, namely the prevalence, pre-extinction, and post-extinction stages, respectively. The Early Pleistocene Baikong fauna includes several elements that prefer open grassland habitats, such as *Procynocephalus*^166-167^, *Pachycrocuta*^168^, *Sinomastodon*^169^, *Hesperotherium*^170^, *Hippopotamodon*^171^, and *Megalovis*^172^. These elements either completely disappear in the later Yixiantian fauna and Baxian fauna, such as *Procynocephalus*, *Sinomastodon*, *Hesperotherium*, and *Hippopotamodon*, or shrink greatly in number, such as *Megalovis*, or are replaced by their descendants but also shrink greatly in number, such as *Pachycrocuta* and *Crocuta* (Fig. S2).

The habitat reconstruction also shows that more individuals preferred temperate forest (1.4 in Fig. S3) and conspicuously larger proportion of individuals preferred dry savanna (2.1 in Fig. S3) in the Baikong fauna than in the Yixiantian fauna and Baxian fauna. However, more individuals preferred various kinds of subtropical/tropical forests and various kinds of shrublands in the Yixiantian fauna and Baxian fauna than in the Baikong fauna (1.5, 1.6, 1.9, 3.4, 3.5, 3.6, and 3.7 in Fig. S3). It seems that *Gigantopithecus blacki* favours the Early Pleistocene cooler environment than the Middle Pleistocene warmer environment. And this is probably one of the key factors that lead to its extinction.


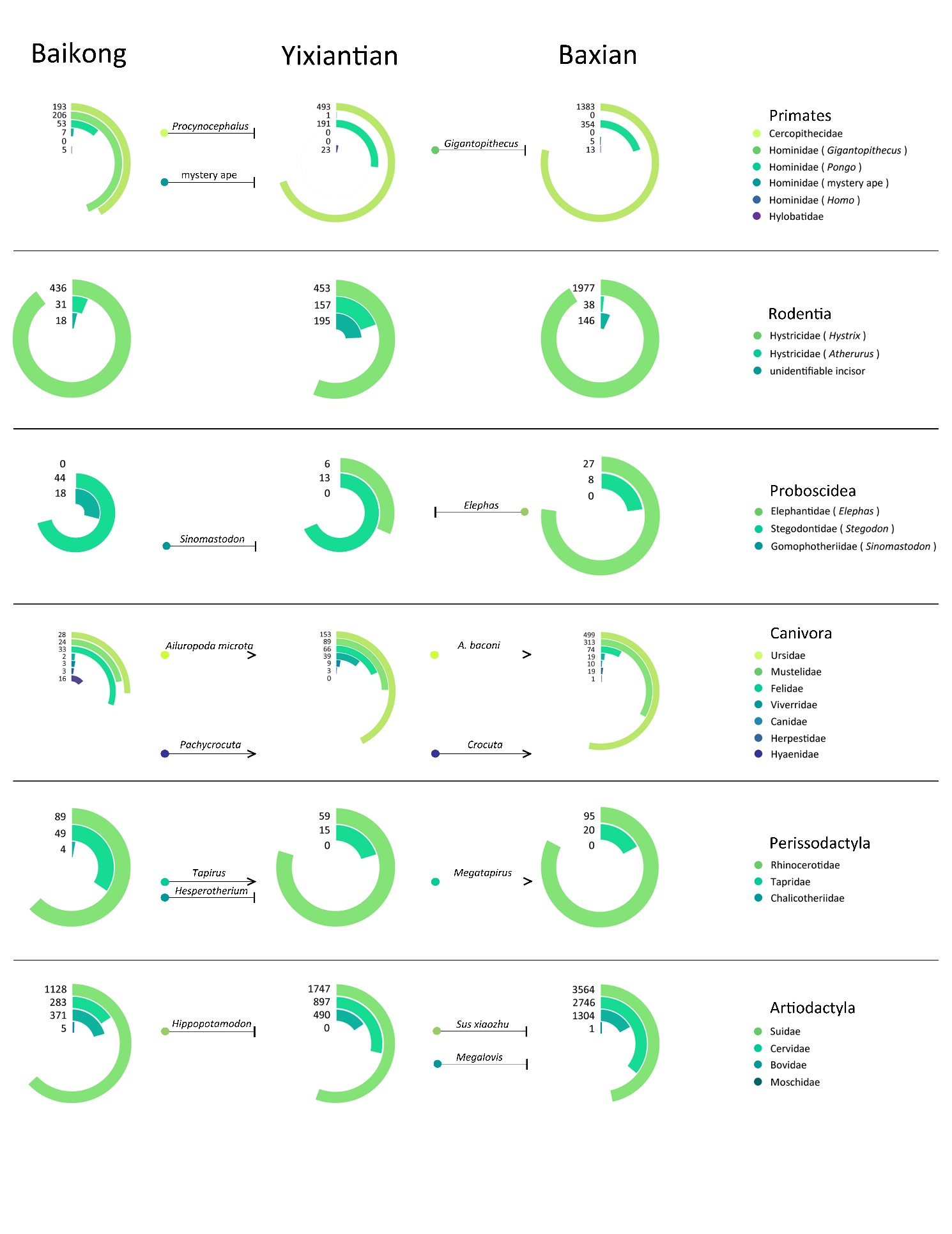


**Figure S2**: Compositional changes and events of extinction, migration, and faunal turnovers in the selected Chongzuo Pleistocene faunas. Numbers on each donut chart: specimen number; ┤: event of extinction; ├: event of migration; **→**: event of replacement

**Figure S3**. Predominant habitats reflected by the Baikong, Yixiantian, and Baxian faunas. For the habitat types on the horizontal axis, see IUCN Habitats Classification Scheme (Ver. 3.1). The procedure for calculating the habitat predominance index for a fauna (HPIF) is as follows: 1) Individual Number Index for a Genus (INIG) = (the specimen number of a genus) / (the number of teeth of the genus), 2) Individual Number Index for a Fauna (INIF) = the sum of INIGs of all the genera in a fauna, 3) Habitat Predominance Index for a Genus (HPIG) = INIG × (the counts of a habitat type preferred by all the extant species of the genus in Guangxi) ÷ (the counts of all habitat types preferred by all the extant species of the genus in Guangxi) ÷ INIF × 100, and 4) Habitat Predominance Index for a Fauna (HPIF) = the sum of HPIGs of all the genera in a fauna.

**SI section 4: Luminescence dating of breccia matrix**

**Sample collection and processing**

Large blocks of the hard-cemented fossil-bearing breccia from each cave were cut from the section in situ and wrapped in black plastic. Within subdued red-light conditions, the light exposed outer layer was removed using a chisel and hammer and was retained as the dosimetry sample. These layers were gently broken up using a pestle and mortar and oven dried, then the entire fraction was milled and used for environmental dose estimation. The unexposed inner core was also gently broken up using a pestle and mortar and was processed using the standard sample purification procedures for quartz and feldspar separation^31^ including a 40%/10% wash in hydrofluoric acid for 45/10 mins, respectively, to remove the external alpha-dosed rinds^173^. All luminescence analyses were conducted at the ‘Traps’ luminescence dating facility at Macquarie University in Sydney, Australia.

Preliminary luminescence analysis in this region of southern China revealed high dose rates (~3-4 Gy/kyr), and a saturating quartz signal for caves sites greater than 100 kyr in age. However, feldspar minerals have a high dose response and stable signal that could be isolated from the unstable fading signal^174,175^, but can in some regions yield a very low number of grains with a measurable luminescence signal (e.g.,^176^). Most samples also recovered a higher quartz than feldspar yield, but as the expected age range of ~300 ka would push quartz substantially past the 200 Gy saturation limit we decided that feldspars presented a better opportunity for establishing the time since burial. Using past experience of feldspar applications in Southeast Asia^176-178^, we decided to focus on applying post-infrared infra-red-stimulated-luminescence (pIR-IRSL) techniques to the feldspars in the caves thought to be older than 100 kyr and applied quartz single-grain techniques to the sediments from caves thought to be younger than 100 kyrs and that yielded very low quantities of feldspars.

**Sample measurements**

**Single-grains -** Individual 180-212 µm quartz or feldspar grains were mounted onto coated single grain discs in a 10 by 10 grid. The discs were loaded onto a carousel and processed in a Riso TL-DA-20 containing an automated Detection and Stimulation Head (DASH) set up with a Dual laser single grain attachment with a Blue/UV sensitive Electron Tube PMT (PDM9107Q-AP-TTL-03) with maximum detection efficiency between 200 and 400 nm. The filters in the automated detection changer were set on the blue filter pack for feldspars (Schott BG-39 and Corning 7-59 filters to transmit wavelengths of 320–480 nm (53) and UV filter pack (Hoya 7.5 mm U-340 filter) for quartz. Using a modified SAR procedure^179^ (Table S8 for pIR-IRSL protocol) the feldspar grains were stimulated for 2.5 s, first at 50 °C and secondly at 270 °C after a 300°C preheat according to the procedures of the pIR-IRSL protocol selected using an IR (830 nm) 140 mW TTL modulated laser with a 3 mm RG-780 longpass filter (mounted directly in front of the IR laser). The signal was integrated over the first 0.21-0.46 s with the last 1.88-2.5 s used as a background, with a standard exponential fit and monte carlo simulation for error determination.

The quartz grains were stimulated for 2 s at 125ºC using a 10 mW 532 nm Nd:YV04 solid-state diode-pumped green laser with 90% power corresponding to 25 W/cm^2^ according to the standard SAR protocols of ^179^. The single-grain disc locating process was programmed to occur before any disc heating to stimulation temperature to ensure that differences in the disc locating procedures would not result in grains receiving an extended heating duration within the SAR cycles. The signal was integrated over the first 0.1-0.32 s with the last 1.6-2 s used as a background, and the dose response was fitted with an exponential fitting function and the error on the fit was generated using a Montecarlo simulation.

**Single-aliquots –** The 90-180 um feldspar grains were mounted onto stainless steel single-aliquot discs using Silkospray and a 3 mm mask. The discs were loaded into the same Riso unit containing the same DASH, PM and filter setup as described above but the quartz window was installed. Using a modified SAR procedure^179^ the feldspar grains were stimulated for 250 s using IR diodes (850 nm and 300 mW/cm^2^), and the same temperature procedures as outlined in Table S8. The signal was integrated over the first 6 s of stimulation with the last 20 s used as a background, and the dose response was also fitted with an exponential fitting function and Montecarlo simulation in Analyst.

**Table S8** pIR-IRSL SAR procedure

| **Step** | **Treatment** | **Data collected** |
| --- | --- | --- |
| 1 | Give Dose D_0_, D_i_ |  |
| 2 | Preheat 300 ºC for 60s |  |
| 3 | IR stimulation; SA IR diodes - 250 s, SG laser 2.5 s at 50 ºC |  |
| 4 | IR stimulation; SA IR diodes - 250 s, SG laser 2.5 s at 270 ºC | L_x_ |
| 5 | Give test dose D_t_ |  |
| 6 | Preheat 300 ºC for 60s |  |
| 7 | IR stimulation; SA IR diodes - 250 s, SG laser 2.5 s at 50 ºC |  |
| 8 | IR stimulation; SA IR diodes - 250 s, SG laser 2.5 s at 270 ºC | T_x_ |
| 9 | IR stimulation; IR diodes - 100 s at 310 ºC |  |
| 10 | Return to step 1 for R1, R2, R3, R4 zero, R5 double regen |  |

**Dosimetry**

To obtain an estimate of the environmental dose rate for each of the samples firstly we measured beta dose rates using a Geiger-Muller multi-counter beta counting of dried and powdered sediment samples^180^ in the laboratory. Allowance was made for the effect of sample moisture content^31^, different grain sizes^181^ and HF etching^182^ on attenuation of the beta dose and the total beta dose-rate contribution was calculated by comparing the beta count rate to a standard beta source (SHAP with a dose rate of 5.99 Gy/ka) and magnesium oxide as a non-beta emitting background material. Secondly, thick source alpha counting using a Daybreak 583 intelligent alpha counter was used to obtain estimates of Uranium and Thorium^183^ to estimate the gamma dose rate, and thirdly the difference between beta and alpha counting was used to estimate potassium values. These estimates were then converted to gamma and beta dose rates using the conversion factors of^34^. Fourthly, in situ gamma measurements were conducted using an Inspector 1000 from Canberra at selected sites (Table S11) to estimate the in-situ contribution of the gamma dose using the threshold technique. This can differ from the sedimentary estimations depending on the composition of material within a 30 cm radius of the sampling location. Finally, samples were collected for high resolution gamma spectrometry to measure the activities of radionuclides in the ^238^U, ^235^U, ^232^Th decay chains, and of ^40^K at the SGS laboratory in Melbourne. These activities were converted to beta and gamma dose rates using the conversion factors of ^34^ and adjusted for long-term water content. These activities provide an alternate dosimetry and were also used to estimate the potential disequilibrium in this cave environment.

Allowance was made for the effect of sample moisture content^31^ on the external beta and gamma dose rates using a long-term water content of between 5 ± 2 - 25 ± 5 %, which is similar to the measured (field) water content of between 4-41%, and allows for an initial period of saturation when first deposited in the karst environment. The total dose rate was then calculated using an effective internal beta dose rate of 0.032 Gy/ka for 180-212 µm quartz^184^ and 0.84 Gy/ka for 180-212 µm feldspars (due to the radioactive decay of ^40^K and ^87^Rb), which were made assuming K (12.5 ± 0.5 %^185^ and ^87^Rb 400 ± 100 μg g^–1 186^) concentrations and included in the total dose rate (see below for mineralogy tests). Cosmic-ray dose rates were estimated from published relationships^36^, making allowance for the thickness of limestone above the cave (~20-80 m with an assumed density of 1.2 g/cm^3^), sediment overburden at the sample locality (0.25-3.99 m with an assumed density of 2.0 g/cm^3^), the altitude (between 143-448 m above sea level) and geographic latitude and longitude (22°N /107°E - 23°N /106°E) of the sampling sites. The final dose rates varied from 1.1-4.2 Gy/ka with the majority being around the 2-3 Gy/ka level.

**Investigations into sample mineralogy using XRD and XRF -** Sample mineralogy was determined using X-ray Diffractometry. Samples were mounted on a silicon crystal low background holder, and diffractograms were collected from 5° to 90° 2θ using a PANalytical X’ Pert pro MPD diffractometer, using 45 kV, 40 mA and CuKα radiation at 5° 2θ.min-1. Identification of minerals was undertaken using PANalytical’s High Score Plus v2.2.4 software, with ICDD PDF2 and PAN-ICSD databases. Detection limits depend on crystallinity but are typically around 0.1-0.5 wt%. In addition, the K content of the samples was estimated using a PANalytical Epsilon 3 XL X-ray Fluorescence spectrometer with conditions of 50 kV for 80 seconds with 100 µm Ag filter, 50 kV for 80 seconds with 300 µm Cu filter, 20 kV for 300 seconds with 200 µm Al filter, 12 kV for 120 seconds with 50 µm Al filter and 5 kV for 200 seconds with no filter. The estimated K content of the samples varied from ~10-14% with some samples containing small amounts of Na feldspars and others containing some quartz (despite rigorous mineral separation procedures).

**Procedural tests**

**pIR-IRSL of feldspars**

Initial feldspar procedural runs incorporated single-aliquots (90-180 um) to maximise signal counts and to test the suitability for single grain analysis. Due to low yields in potassium feldspar grains at these sites, we decided to only apply single-grain pIR–IRSL techniques to a select number of suitable samples. These samples were chosen based on the number of single-aliquots that produced measurable IRSL decays. Due to the small amount of grains in the 180-212 μm size fraction we saved this for the single-grain D_e_ estimation and instead used the smaller 90-180 μm size fraction for all the procedural tests conducted on sample CSH1 (Table S9). To determine the correct procedures, we applied the same tests described in ^176^ to single-aliquots, but then modified them slightly to test single-grains of feldspar for certain samples that yielded a slightly larger amount of the 180-212 um size fraction (using 200 µm single-grain discs) in line with^187^; 1) a preheat plateau test; 2) fading tests; 3) bleaching tests and 4) dose recovery tests. Using the following preheat and IR stimulation combinations we tested: 1) 250 and 225°C (pIR-IRSL_50,225_)^174,188^, 2) 280 and 250°C (pIR-IRSL_50,250_) ^189^, 3) 300 and 270°C (pIR-IRSL_50,270_)^189^, 4) 320 and 290°C (pIR-IRSL_50,290_)^175^ and as the expected D_e_ is >450 Gy we also tested 5) 320 and 200/290°C (pIR-IRSL_200,290_)^190^.

As many of the discs contained very few feldspar decays we used four single grains discs with a total of 24 accepted grains and recycled these discs for all the tests, with one disc per preheat/stimulation combination. This is not ideal and the use of fresh grains would have been preferred but the low feldspar yield meant that we had to prioritise the remaining sample for the actual measurements. All tests mostly followed the procedures outlined in ^176^ with the following differences;

1. preheat plateau and dose recovery tests - the different preheat/IR laser stimulation combination was applied to each SG disc and to a different number of grains depending on how many grains luminesced on each disc. We then added a surrogate dose of 20 Gy to try and recovered this known dose from these discs.

2. Bleaching tests - the same 4 discs had a dose of 20 Gy applied and were bleached in a solar simulator and measured using the same preheat/IR laser stimulation combinations.

3. Following the protocols of ^187^ for testing anomalous fading in SG of feldspars we employed 1) individual single-grain fading using the 4 discs for multiple delay times, 2) multiple grain measurements by using the SG discs as single-aliquots, and 3) the use of a standard fading value assessment by the degree of saturation for an infinite aged sample derived from the weathered granite clast found in the deposit and analysed using small single-aliquots.

**Table S9:** Procedural test results for single-grains of feldspar from sample CSHT1

| **Tests** | (pIR-IRSL  _50,225_) | (pIR-IRSL  _50,250_) | (pIR-IRSL  _50,270_) | (pIR-IRSL  _50,290_) | (pIR IRSL  _200,290_) |
| --- | --- | --- | --- | --- | --- |
| Preheat range (Gy) | 180 | 95 | 23 | 54 | 50 |
| Dose recovery ratio | 0.945 | 0.975 | 0.982 | 1.020 | 1.252 |
| Fading SG % per decade | 7.84 | 5.76 | 3.21 | 4.34 | 6.39 |
| Fading SA % per decade | 6.02 | 5.31 | 2.31 | 3.92 | 4.94 |
| Residuals (Gy) | 6.3 | 7.4 | 11.4 | 46 | 34 |
| Recuperation % | 1.0 ± 0.002 | 0.9 ± 0.001 | 0.5 ± 0.001 | 1.3 ± 0.02 | 2.8 ± 0.002 |
| Recycling ratio | 1.04 ± 0.03 | 1.07 ± 0.02 | 1.01 ± 0.02 | 0.87 ± 0.03 | 1.32 ± 0.03 |
| Average D_0_ (Gy) | 840 ± 79 | 780 ± 68 | 956 ± 102 | 1033± 89 | 823 ± 76 |

From these tests it was determined that the 270°C stimulation and 300°C preheat combination plot within the flattest part of the preheat plateau provided the best recovery of the surrogate dose, with the least fading of all the pIR-IRSL signals (average g values of ~ 2.3 % per decade) and one of the lowest residual value (<12 Gy) (Extended Data Fig. 3).

The tests revealed that the flattest plateau was provided by the pIR-IRSL_50,270_, pIR-IRSL_50,290_ and pIR-IRSL_200,290_ signals, while the pIR-IRSL_50,270_ signal provided the best recovery of the surrogate dose (with a dose recovery ratio of 1.020) and lowest residual values after bleaching (<12 Gy) (although calculated, these residual dose were not subtracted from the D_e_) (ED3). The pIR-IRSL_50,290_ and pIR-IRSL_200,290_ signals produced the largest residuals doses, which raises concerns over signal overestimation in these cave environments where there is a strong likelihood for partial bleaching. The fading results from the single aliquot tests (2-6%) were lower than from the single-grain tests (3-7%), which revealed a range of fading from the highest IR_50_ (~12 %) to the lowest pIR-IRSL_50,270_ (2-3%), pIR-IRSL_50,290_ (3-4%) and pIR-IRSL_200,290_ signals (4-6%). We also observed a variation in fading rates amongst the single-grains (ranging from negative values to higher values that would not allow for any signal in the natural sample, with an average over the 24 grains of 3.6 % per decade) but this was independent of the grains sensitivity. When using the pIR-IRSL_50,270_ signal on the single-aliquot discs this *g* value was reduced to an average of 2.3 % per decade. Surprisingly the pIR-IRSL_200,290_ signal displayed the same amount of fading as seen in the pIR-IRSL_50,270_ and pIR-IRSL_50,290_ signals, indicating no apparent advantage in a higher initial temperature for IR stimulation in these samples.

In an effort to understand the extent of fading in these samples we wanted to investigate the field saturation of the feldspar grains. To do this we had to find an outcrop of rock that contained feldspars. As the dominant geology in this region is Triassic and Carboniferous cherty limestone, the feldspars in these samples must have been transported via fluvial process from the late Palaeozoic granite lithology found far to the south of these caves^151^. Thus, to represent the local granite geology from the region a small weathered granitic clast found in the cave breccias was used to estimate the field saturation as the closest representation of the provenance of the feldspar grains. Using 12 very small single-aliquots with a mask size of 0.5 mm (as the signal was too insensitive to use single-grains), large doses of 200 and 400 Gy were added to the natural signal for eight discs and zero dose for the final four discs. All discs were measured using the ph/stimulation temperature combinations described above. In all the discs the IR_50_ signal grows with dose and the pIR-IRSL_50,225_ signal to a lesser degree, but the pIR-IRSL_50,290_ and pIR-IRSL_200,290_ signals change by only a small amount. We assumed that this field saturation must be greater than or equal to the 2 x D_0_, which in this test sample was 1920 ± 204 Gy. This indicates that the field saturation is fairly close to the laboratory saturation using these signals, thus the fading is neglible, but however to be conservative we used a fading rate of 2.0 % per decade. We used the fading correction method by^32^. This field saturation value also provides a comparison to test the D_0_ values obtained from the procedural tests and provides an upper limit for testing the saturation in these sample. All the D_e_ values obtained are well within this range.

Single aliquot and single grains were rejected based on the rejection criteria of ^191^ (see Table S10) and the later were plotted on radial plots to visualise the grain populations. The intrinsic sources of uncertainty were estimated using a modern-day analogue sample collected from outside Shuangtan Cave. Dose recovery tests were conducted on this sample to estimate the natural range of variability between the grains as an estimation of the instrinsic sources of uncertainty including instrument reproducibility^192^. Prior to the the application of the MAM model^192^ to the accepted equivalent doses this additional uncertainty of 7% for single aliquots and 12% for single grains was added as a percentage of the individual dose estimates to the uncertainty of all individual dose estimates (calculated from counting statics and curve fitting errors). MAM was applied to the equivalent doses and the MAM D_e_ generated age was corrected according to the results of the anomalous fading tests (using a weighted mean fading rate of 2.0 ± 0.2 % per decade).

**Table S10** - The classification of all single-grain rejections for selected samples. A - rejections of pIR-IRSL feldspars, B – rejections for single-grain OSL quartz.

**A**

| **Rejections pIR-IRSL feldspars** | **CSHT11** | **HEJ1** | **CGONG16** | **CGONG17** | **CQUZ18** | **CXK5** |
| --- | --- | --- | --- | --- | --- | --- |
| Total grains run | 2500 | 2000 | 600 | 600 | 800 | 800 |
| Accepted | 79 | 25 | 33 | 36 | 20 | 66 |
| Acceptance rate (%) | 3.16 | 1.25 | 5.50 | 6.00 | 2.50 | 8.25 |
| Poor signals <3 >sd bg^a^ | 2130 | 1867 | 534 | 502 | 675 | 741 |
| Recycling ratios > or < 0.8-1.2^b^ | 243 | 76 | 9 | 32 | 50 | 8 |
| Super saturated^c^ | 4 | 0 | 0 | 2 | 1 | 0 |
| Recuperation >5 %^d^ | 28 | 28 | 11 | 13 | 29 | 6 |
| D_e_ = 1 sig 0^e^ | 95 | 29 | 46 | 51 | 45 | 45 |

a Net natural signals less than three times above the standard deviation of the background

b Recycling ratios outside of the range 0.8-1.2,

c Yielded natural signals that did not intersect the regeneration growth curves,

d Signal larger than 5% of the natural signal after a zero dose

e Produced a D_e_ within 1-sigma of zero.

**B**

| **Rejections OSL quartz** | **CXK9** | **CMF2** | **CLB4** | **CLPB5** | **CWUY7** | **CGAN1** | **CGAN2** |
| --- | --- | --- | --- | --- | --- | --- | --- |
| Total grains run | 500 | 700 | 800 | 600 | 1000 | 800 | 500 |
| Accepted | 24 | 25 | 47 | 41 | 59 | 51 | 35 |
| Acceptancew rate | 4.80 | 3.57 | 5.88 | 6.83 | 5.90 | 6.38 | 7.00 |
| Poor signals <3 >sd bg^a^ | 389 | 351 | 659 | 542 | 843 | 657 | 411 |
| Recycling ratios > or < 0.8-1.2^b^ | 31 | 45 | 38 | 3 | 65 | 44 | 14 |
| Super saturated^c^ | 0 | 6 | 2 | 0 | 9 | 0 | 0 |
| Recuperation >5 %^d^ | 24 | 52 | 14 | 4 | 23 | 10 | 8 |
| D_e_ = 1 sig 0^e^ | 56 | 246 | 87 | 51 | 60 | 89 | 67 |

a Net natural signals less than three times above the standard deviation of the background

b Recycling ratios outside of the range 0.8-1.2,

c Yielded natural signals that did not intersect the regeneration growth curves,

d Signal larger than 10% of the natural signal after a zero dose

e Produced a D_e_ within 1-sigma of zero.

**Single-grain OSL of quartz**

We used a standard SAR protocol^179^ for between 1000-1500 single-grains of quartz. The first 0.2 s of luminescence decay was used for signal integration and the last 0.3 s for background integration. Feldspar contamination was checked during analysis using an infrared wash (stimulation with IR diodes at 50°C for 100 s) plus an additional IR depletion cycle on the end of the SAR run. For each sample a preheat plateau test was conducted on 3 single aliquots of quartz grains at temperatures of 220, 240, 260 and 280 °C, revealing the widest plateau at 260 °C and the chosen preheat. A dose recovery run (eight aliquots with signals removed at room temperature and bleached with blue diodes) at this preheat temperature tested the legitimacy of these measurement parameters, with all aliquots returning the surrogate dose within errors.

Despite only measuring the quartz grains from the younger caves the high dose rate meant that saturation was an issue with the measurement of these samples. We played close attention to the 2 x D_0_ rule for saturation and rejected grains that produced dose responses that exceed this 2 x D_0_ value. The acceptance rate of grains was also low for these samples but as they yielded higher quantities of quartz a more statistically significant number of grains could be obtained. Nevertheless, some samples (e.g., CXK5) produced age estimates that significantly under estimated the expected value according to the age of the overlying flowstone.

**Results**

Overall, 55 feldspar and quartz single-aliquot and single-grain D_e_ were produced for 37 breccia samples (Table S11,12). These D_e_ values ranged from 38-1756 Gy. The feldspars had low sensitivities yielding very low acceptance rates in both the single-aliquots and single-grains. For example, for sample HEJ1 over 2000 grains were processed with only 25 accepted grains (1.25 % acceptance rate) and this was one of the samples that yielded enough grains to run multiple discs. Most of the grains had no decay whatsoever. The quartz produced more viable decays and acceptance rates were slightly better at on average 5.76 %, but quartz saturation played a role in some of the rejections. Dose rates ranged from 1.134 ± 0.203-2.561 ± 0.241 Gy/ka for quartz and 1.947 ± 0.111- 4.243 ± 0.277 Gy/ka for feldspars.

For the single-aliquots that luminesced, the feldspar data provided strong shinedown curves, with exponential dose response and average D_0_ values in the range of ~800 Gy. The overdispersion between aliquots ranged from 14-34% with only two caves (Yanliang and Yixiantian) producing OD values in the range of 60-80%. The single-grain feldspar data contained some very bright grains (see ED3b) which also provided an exponential dose response curve and slightly higher D_0_ values of between 900-1000 Gy. The overdispersion on the single grain distributions ranged between 25-33% with only one sample slightly higher at 47%. The OSL quartz single grains results displayed dimmer luminescence (see ED3c) and on average slightly higher overdispersion (37-51 %) with much lower D_0_ values at ~150 Gy.

**Table S11**: pIR-IRSL and single-grain quartz dating of sediments from the cave sites of southern China: dose rate data, equivalent doses, and ages

|  |  |  |  |  |  |  |  |  |  |  |  |  |  |  |  |  |  |  |  |  |  |  |  |  |
| --- | --- | --- | --- | --- | --- | --- | --- | --- | --- | --- | --- | --- | --- | --- | --- | --- | --- | --- | --- | --- | --- | --- | --- | --- |
|  |  | **Sample** | **Depth** | | **Grain** | | **Beta** | | **Gamma** | | **Cosmic-ray** | | **Internal** | | **Water** | | **Total** | | **Technique^g^** | | **Equivalent** | | **Age ^i^** |  |
|  | **CAVES** | **code** | **sediment** | **Size** | | **dose rate^a^** | | **dose rate^b^** | | **dose rate^c^** | | **dose rate^d^** | | **content^e^** | | **dose rate^f^** | |  | | **dose^h^** | |  | | |
|  |  |  | **(m)** | **(µm)** | | **(Gy/ka)** | | **(Gy/ka)** | | **(Gy/ka)** | |  | | **(%)** | | **(Gy/ka)** | |  | | **(Gy)** | | **(ka)** | | |
|  | **CHONGZUO** | |  |  | |  | |  | |  | |  | |  | |  | |  | |  | |  | | |
|  | SHUANGTAN | **CSHT-1** | 0.53 | 90-180 | | 1.485 ± 0.090 | | 0.469 ± 0.004* | | 0.016 | | 0.720 | | 6 / 5 ± 2 | | 2.690 ± 0.144 | | **pIR-IRSL-SA** | | 702 ± 85 | | **261 ± 35** | | |
|  |  |  |  | 180-212 | | 1.404 ± 0.089 | | 0.469 ± 0.004* | | 0.016 | | 0.840 | | 6 / 5 ± 2 | | 2.730 ± 0.225 | | **pIR-IRSL-SG** | | 690 ± 111 | | **253 ± 46** | | |
|  |  | **CSHT-2** | 0.39 | 90-180 | | 1.482 ± 0.068 | | 0.431 ± 0.004* | | 0.015 | | 0.720 | | 9 / 10 ± 2 | | 2.647 ± 0.162 | | **pIR-IRSL-SA** | | 785 ± 98 | | **297 ± 42** | | |
|  |  |  |  | 180-212 | | 1.401 ± 0.066 | | 0.431 ± 0.004* | | 0.015 | | 0.840 | | 9 / 10 ± 2 | | 2.687 ± 0.235 | | **pIR-IRSL-SG** | | 684 ± 79 | | **255 ± 38** | | |
|  |  | **CSHT-11** | 0.15 | 90-180 | | 1.482 ± 0.068 | | 0.431 ± 0.004* | | 0.015 | | 0.720 | | 9 / 10 ± 2 | | 2.647 ± 0.162 | | **pIR-IRSL-SA** | | 705 ± 82 | | **266 ± 36** | | |
|  |  |  |  | 180-212 | | 1.401 ± 0.066 | | 0.431 ± 0.004* | | 0.015 | | 0.840 | | 9 / 10 ± 2 | | 2.687 ± 0.235 | | **pIR-IRSL-SG** | | 730 ± 82 | | **272 ± 39** | | |
|  | BAPENG | **CBAP-3** | 0.63 | 90-180 | | 1.011 ± 0.054 | | 0.519 ± 0.004* | | 0.015 | | 0.720 | | 9 / 10 ± 2 | | 2.265 ± 1.20 | | **pIR-IRSL-SA** | | 335 ± 87 | | **148 ± 39** | | |
|  |  |  |  |  |  |  |  |  |  |  |  |  |  |  |  |  |  |  |  |  |  |  |  |  |
|  |  | **CBAP-6** | 2.36 | 180-212 | | 1.222 ± 0.046 | | 0.330 ± 0.004* | | 0.018 | | 0.840 | | 7 / 5 ± 2 | | 2.410 ± 0.209 | | **pIR-IRSL-SG** | | 1099 ± 533 | | **456 ± 225** | | |
|  |  |  |  |  |  |  |  |  |  |  |  |  |  |  |  |  |  |  |  |  |  |  |  |  |
|  | HEIJIANG | **CHEJ -1** | 3.99 | 90-180 | | 0.966 ± 0.033 | | 0.398 ± 0.004* | | 0.016 | | 0.720 | | 5 / 5 ± 2 | | 2.100 ± 0.107 | | **pIR-IRSL-SA** | | 749 ± 85 | | **357 ± 45** | | |
|  |  |  |  | 180-212 | | 0.913 ± 0.031 | | 0.398 ± 0.005* | | 0.016 | | 0.840 | | 5 / 5 ± 2 | | 2.167 ± 0.203 | | **pIR-IRSL-SG** | | 690 ± 84 | | **318 ± 49** | | |
|  |  | **CHEJ -2** | 2.44 | 90-180 | | 1.150 ± 0.041 | | 0.268 ± 0.004* | | 0.016 | | 0.720 | | 7 / 5 ± 2 | | 2.154 ± 0.110 | | **pIR-IRSL-SA** | | 581 ± 65 | | **270 ± 21** | | |
|  |  |  |  | 180-212 | | 1.088 ± 0.039 | | 0.268 ± 0.004* | | 0.016 | | 0.840 | | 7 / 5 ± 2 | | 2.211 ± 0.205 | | **pIR-IRSL-SG** | | 613 ± 82 | | **277 ± 45** | | |
|  |  | **CHEJ -3** | 0.91 | 90-180 | | 1.114 ± 0.039 | | 0.253 ± 0.005* | | 0.016 | | 0.720 | | 19 / 15 ± 5 | | 2.102 ± 0.152 | | **pIR-IRSL-SA** | | 613 ± 77 | | **292 ± 44** | | |
|  |  |  |  | 180-212 | | 1.053 ± 0.038 | | 0.253 ± 0.004* | | 0.016 | | 0.840 | | 19 / 15 ± 5 | | 2.162 ± 0.228 | | **pIR-IRSL-SG** | | 626 ± 68 | | **289 ± 45** | | |
|  | YAN  LIANG | **CYAN -12** | 0.70 | 90-180 | | 0.965 ± 0.033 | | 0.245 ± 0.002* | | 0.016 | | 0.720 | | 7 / 5 ± 2 | | 1.947 ± 0.111 | | **pIR-IRSL-SA** | | 619 ± 160 | | **318 ± 84** | | |
|  |  |  |  |  |  |  |  |  |  |  |  |  |  |  |  |  |  |  |  |  |  |  |  |  |
|  |  | **CYAN -13** | 2.00 | 90-180 | | 1.488 ± 0.049 | | 0.233 ± 0.004* | | 0.015 | | 0.720 | | 7 / 5 ± 2 | | 2.456 ± 0.120 | | **pIR-IRSL-SA** | | 1286 ± 318 | | **524 ± 132** | | |
|  | YIXIANTIAN | **CYIX -14** | 0.43 | 90-180 | | 1.559 ± 0.057 | | 0.350 ± 0.004* | | 0.012 | | 0.720 | | 24 / 15 ± 5 | | 2.641 ± 0.154 | | **pIR-IRSL-SA** | | 842 ± 215 | | **319 ± 84** | | |
|  |  |  |  |  |  |  |  |  |  |  |  |  |  |  |  |  |  |  |  |  |  |  |  |  |
|  |  | **CYIX -15** | 1.90 | 90-180 | | 1.344 ± 0.047 | | 0.388 ± 0.005* | | 0.012 | | 0.720 | | 14 / 10 ± 5 | | 2.464 ± 0.147 | | **pIR-IRSL-SA** | | 1803 ± 160 | | **732 ± 81** | | |
|  | BAXIAN | **CBAX -4** | 0.57 | 90-180 | | 1.211 ± 0.049 | | 0.400 ± 0.015* | | 0.014 | | 0.720 | | 13 / 10 ± 2 | | 2.346 ± 0.119 | | **pIR-IRSL-SA** | | 608 ± 111 | | **259 ± 49** | | |
|  |  |  |  | 180-212 | | 1.146 ± 0.047 | | 0.400 ± 0.015* | | 0.014 | | 0.032 | | 13 / 10 ± 2 | | 1.592 ± 0.062 | | **OSL-SG** | | 180 ± 35 | | **113 ± 23** | | |
|  |  | **CBAX -5** | 0.38 | 90-180 | | 1.377 ± 0.051 | | 0.354 ± 0.003* | | 0.014 | | 0.720 | | 10 / 10 ± 2 | | 2.465 ± 0.120 | | **pIR-IRSL-SA** | | 346 ± 96 | | **140 ± 40** | | |
|  |  |  |  | 180-212 | | 1.302 ± 0.049 | | 0.354 ± 0.003* | | 0.014 | | 0.032 | | 10 / 10 ± 2 | | 1.702 ± 0.062 | | **OSL-SG** | | 105 ± 6 | | **62 ± 5** | | |
|  | GONGJISHAN | **CGONG -16** | 0.50 | 90-180 | | 0.751 ± 0.035 | | 0.222 ± 0.003* | | 0.020 | | 0.720 | | 10 / 10 ± 2 | | 1.713 ± 0.110 | | **pIR-IRSL-SA** | | 732 ± 44 | | **427 ± 39** | | |
|  |  |  |  | 180-212 | | 0.710 ± 0.034 | | 0.222 ± 0.003* | | 0.020 | | 0.840 | | 10 / 10 ± 2 | | 1.792 ± 0.205 | | **pIR-IRSL-SG** | | 388 ± 23 | | **216 ± 28** | | |
|  |  | **CGONG-17** | 0.68 | 90-180 | | 1.333 ± 0.051 | | 0.344 ± 0.004* | | 0.020 | | 0.720 | | 10 / 10 ± 2 | | 2.417 ± 0.120 | | **pIR-IRSL-SA** | | 1097 ± 21 | | **454 ± 26** | | |
|  |  |  |  | 180-212 | | 1.261 ± 0.049 | | 0.344 ± 0.004* | | 0.020 | | 0.840 | | 10 / 10 ± 2 | | 2.465 ± 0.225 | | **pIR-IRSL-SG** | | 573 ± 40 | | **233 ± 28** | | |
|  | QUZAI | **CQUZ -18** | 1.00 | 90-180 | | 1.711 ± 0.057 | | 0.639 ± 0.003* | | 0.015 | | 0.720 | | 12 / 8 ± 2 | | 3.085 ± 0.129 | | **pIR-IRSL-SA** | | 626 ± 73 | | **203 ± 25** | | |
|  |  |  |  | 180-212 | | 1.618 ± 0.054 | | 0.639 ± 0.003* | | 0.015 | | 0.840 | | 12 / 8 ± 2 | | 3.112 ± 0.214 | | **pIR-IRSL-SG** | | 619 ± 77 | | **199 ± 29** | | |
|  |  | **CQUZ -19** | 1.30 | 90-180 | | 2.223 ± 0.083 | | 0.685 ± 0.001* | | 0.014 | | 0.720 | | 24 / 15 ± 5 | | 3.642 ± 0.201 | | **pIR-IRSL-SA** | | 717 ± 92 | | **197 ± 28** | | |
|  |  |  |  | 180-212 | | 2.102 ± 0.079 | | 0.685 ± 0.003* | | 0.014 | | 0.840 | | 24 / 15 ± 5 | | 3.641 ± 0.261 | | **pIR-IRSL-SG** | | 732 ± 90 | | **201 ± 28** | | |
|  | XIAOKOU | **CXK -5** | 1.38 | 180-212 | | 1.235 ± 0.050 | | 0.729 ± 0.041* | | 0.02 | | 0.840 | | 6 / 5 ± 2 | | 2.824 ± 0.224 | | **pIR-IRSL-SG** | | 125 ± 17 | | **44 ± 7** | | |
|  |  |  |  |  |  |  |  |  |  |  |  |  |  |  |  |  |  |  |  |  |  |  |  |  |
|  |  | **CXK -9** | 0.50 | 180-212 | | 0.599 ± 0.028 | | 0.729 ± 0.041* | | 0.018 | | 0.032 | | 5 / 5 ± 2 | | 1.378 ± 0.076 | | **OSL-SG** | | 129 ± 15 | | **94 ± 12** | | |
|  |  |  |  |  |  |  |  |  |  |  |  |  |  |  |  |  |  |  |  |  |  |  |  |  |
|  | MA FENG | **CMF -1** | 0.25 | 180-212 | | 1.453 ± 0.061 | | 1.937 ± 0.050* | | 0.012 | | 0.840 | | 41 / 25 ± 5 | | 4.243 ± 0.277 | | **pIR-IRSL-SG** | | 411 ± 111 | | **97 ± 27** | | |
|  |  |  |  | 180-212 | | 1.453 ± 0.061 | | 1.937 ± 0.050* | | 0.012 | | 0.032 | | 41 / 25 ± 5 | | 3.435 ± 0.192 | | **OSL-SG** | | 309 ± 38 | | **90 ± 12** | | |
|  |  | **CMF -2** | 0.75 | 180-212 | | 1.124 ± 0.045 | | 1.385 ± 0.049* | | 0.014 | | 0.032 | | 20 / 15 ± 5 | | 2.555 ± 0.159 | | **OSL-SG** | | 126 ± 6 | | **50 ± 4** | | |
|  |  |  |  |  |  |  |  |  |  |  |  |  |  |  |  |  |  |  |  |  |  |  |  |  |
|  |  | **CMF-3** | 0.60 | 180-212 | | 1.424 ± 0.051 | | 2.000 ± 0.125* | | 0.014 | | 0.032 | | 20 / 15 ± 5 | | 3.470 ± 0.247 | | **OSL-SG** | | 162 ± 21 | | **47 ± 7** | | |
|  |  |  |  |  |  |  |  |  |  |  |  |  |  |  |  |  |  |  |  |  |  |  |  |  |
|  |  | **CMF-4** | 0.20 | 180-212 | | 1.453 ± 0.047 | | 0.601 ± 0.039* | | 0.015 | | 0.840 | | 4 / 5 ± 2 | | 2.910 ± 0.223 | | **pIR-IRSL-SG** | | 386 ± 89 | | **133 ± 32** | | |
|  |  |  |  | 180-212 | | 1.453 ± 0.047 | | 0.601 ± 0.039* | | 0.015 | | 0.032 | | 4 / 5 ± 2 | | 2.102 ± 0.099 | | **OSL-SG** | | 91 ± 9 | | **43 ± 5** | | |
|  | QUE QUE |  |  |  | |  | |  | |  | |  | |  | |  | |  | |  | |  | | |
|  |  | **CQQ1** | 0.45 | 180-212 | | 0.937 ± 0.143 | | 1.092 ± 0.053 | | 0.055 | | 0.840 | | 7 / 5 ± 2 | | 2.924 ± 0.284 | | **pIR-IRSL-SG** | | 1756 ± 75 | | **600 ± 65** | | |
|  |  |  |  |  |  |  |  |  |  |  |  |  |  |  |  |  |  |  |  |  |  |  |  |  |
|  | ZHAN WANG |  |  |  | |  | |  | |  | |  | |  | |  | |  | |  | |  | | |
|  |  | **CZW-2** | 0.45 | 180-212 | | 0.885 ± 0.143 | | 1.309 ± 0.039* | | 0.052 | | 0.840 | | 11 / 10 ± 2 | | 3.086 ± 0.204 | | **pIR-IRSL-SG** | | 998 ± 75 | | **323 ± 35** | | |
|  |  |  |  |  |  |  |  |  |  |  |  |  |  |  |  |  |  |  |  |  |  |  |  |  |
|  | **BUBING BASIN** | |  |  | |  | |  | |  | |  | |  | |  | |  | |  | |  | | |
|  | UPPER PUBU | **CUPB -2** | 0.45 | 90-180 | | 1.166 ± 0.041 | | 0.198 ± 0.002* | | 0.045 | | 0.720 | | 13 / 10 ± 2 | | 2.129 ± 0.114 | | **pIR-IRSL-SA** | | 465 ± 85 | | **218 ± 42** | | |
|  |  |  |  | 180-212 | | 1.103 ± 0.039 | | 0.198 ± 0.002* | | 0.045 | | 0.032 | | 13 / 10 ± 2 | | 1.378 ± 0.053 | | **OSL-SG** | | 165 ± 35 | | **120 ± 26** | | |
|  |  | **CUPB -3** | 0.41 | 90-180 | | 1.775 ± 0.061 | | 0.503 ± 0.028* | | 0.045 | | 0.720 | | 13 / 10 ± 2 | | 3.043 ± 0.130 | | **pIR-IRSL-SA** | | 270 ± 87 | | **89 ± 29** | | |
|  |  |  |  | 90-180 | | 1.775 ± 0.061 | | 0.503 ± 0.028* | | 0.045 | | 0.030 | | 13 / 10 ± 2 | | 2.353 ± 0.083 | | **OSL-SA** | | 172 ± 11 | | **73 ± 6** | | |
|  |  | **CUPB -4** | 0.48 | 90-180 | | 2.049 ± 0.070 | | 0.503 ± 0.028* | | 0.045 | | 0.720 | | 13 / 10 ± 2 | | 3.317 ± 0.137 | | **pIR-IRSL-SA** | | 223 ± 43 | | **67 ± 13** | | |
|  |  |  |  | 180-212 | | 2.049 ± 0.070 | | 0.503 ± 0.028* | | 0.045 | | 0.032 | | 13 / 10 ± 2 | | 2.517 ± 0.090 | | **OSL-SG** | | 174 ± 9 | | **66 ± 4** | | |
|  | LOWER PUBU | **CLPB -4** | 0.27 | 180-212 | | 1.410 ± 0.058 | | 0.460 ± 0.030 | | 0.013 | | 0.032 | | 36 / 20 ± 5 | | 2.202 ± 0.229 | | **OSL-SG** | | 83 ± 5 | | **38 ± 5** | | |
|  |  |  |  |  |  |  |  |  |  |  |  |  |  |  |  |  |  |  |  |  |  |  |  |  |
|  |  | **CLBP -5** | 0.67 | 180-212 | | 0.671 ± 0.024 | | 0.128 ± 0.004 | | 0.014 | | 0.032 | | 11 / 10 ± 2 | | 1.134 ± 0.203 | | **OSL-SG** | | 64 ± 7 | | **56 ± 12** | | |
|  | GANXIAN | **CGAN-1** | 1.40 | 180-212 | | 1.583 ± 0.068 | | 0.513 ± 0.032 | | 0.011 | | 0.032 | | 34 / 20 ± 5 | | 2.428 ± 0.236 | | **OSL-SG** | | 128 ± 11 | | **53 ± 7** | | |
|  |  |  |  |  |  |  |  |  |  |  |  |  |  |  |  |  |  |  |  |  |  |  |  |  |
|  |  | **CGAN-2** | 0.50 | 180-212 | | 1.321 ± 0.052 | | 0.469 ± 0.003 | | 0.012 | | 0.032 | | 27 / 15 ± 5 | | 2.121 ± 0.228 | | **OSL-SG** | | 71 ± 16 | | **33 ± 8** | | |
|  |  |  |  |  |  |  |  |  |  |  |  |  |  |  |  |  |  |  |  |  |  |  |  |  |
|  |  | **CGAN-3** | 2.22 | 180-212 | | 1.754 ± 0.063 | | 0.475 ± 0.004 | | 0.012 | | 0.032 | | 20 / 15 ± 5 | | 2.561 ± 0.241 | | **OSL-SG** | | 38 ± 4 | | **15 ± 2** | | |
|  | ZONG SHAN |  |  |  | |  | |  | |  | |  | |  | |  | |  | |  | |  | | |
|  |  | **CZS-6** | 0.35 | 180-212 | | 0.530 ± 0.032 | | 0.224 ± 0.002 | | 0.045 | | 0.840 | | 13 / 10 ± 2 | | 1.639 ± 0.204 | | **pIR-IRSL-SG** | | 242 ± 18 | | **148 ± 22** | | |
|  |  |  |  |  |  |  |  |  |  |  |  |  |  |  |  |  |  |  |  |  |  |  |  |  |
|  | WUYUN | **CWUY-7** | 0.50 | 180-212 | | 0.584 ± 0.028 | | 0.166 ± 0.003 | | 0.013 | | 0.032 | | 8 / 5 ± 0.2 | | 1.084 ± 0.202 | | **OSL-SG** | | 116 ± 10 | | **107 ± 22** | | |
|  |  |  |  |  |  |  |  |  |  |  |  |  |  |  |  |  |  |  |  |  |  |  |  |  |
|  | LUMEI | **CLUM-1** | 0.30 | 90-180 | | 1.502 ± 0.055 | | 0.416 ± 0.037 | | 0.043 | | 0.720 | | 20 / 15 ± 2 | | 2.681 ± 0.123 | | **pIR-IRSL-SA** | | 100 ± 24 | | **37 ± 9** | | |
|  |  |  |  | 180-212 | | 1.420 ± 0.052 | | 0.416 ± 0.022 | | 0.043 | | 0.032 | | 20 / 15 ± 2 | | 1.912 ± 0.069 | | **OSL-SG** | | 139 ± 38 | | **35 ± 10** | | |
|  |  | **a** | Concentrations determined from beta counter measurements of dried and powdered sediment samples. | | | | | | | | | | | | | | |  | |  | |  | | |
|  |  | **b** | Determined from U, Th and K concentrations measured using a combination of portable gamma-ray spectrometer at field water content (marked with a *) and thick source alpha counting using a Daybreak 583 intelligent alpha counter (combined with the beta counter data) | | | | | | | | | | | | | | | | |  | |  | | |
|  |  | **c** | Time-averaged cosmic-ray dose rates (for dry samples), each assigned an uncertainty of ± 10%. | | | | | | | | | | | | | | |  | |  | |  | | |
|  |  | **d** | An internal dose rate of 0.032 Gy/ka was assumed for quartz^184^ and 0.072 Gy/ka for feldpsars assuming K of 13%^185^ and Rb 400 +-100 ug^186^ | | | | | | | | | | | | | | | | | | | | | |
|  |  | **e** | Field / time-averaged water contents, expressed as (mass of water/mass of dry sample) x 100. The latter values were used to calculate the total dose rates and TL/IRSL ages | | | | | | | | | | | | | | | | | | | | | |
|  |  | **f** | Mean ± total (1σ) uncertainty, calculated as the quadratic sum of the random and systematic uncertainties | | | | | | | | | | | | | | |  | |  | |  | | |
|  |  | **g** | pIR-IRSL-post infra-red infrared stimulated luminescence on single-grains (SG) or single aliquots (SA) of feldspars, OSL-SG - optically stimulated luminescence on single grains of quartz | | | | | | | | | | | | | | | | | | | | | |
|  |  | **h** | Palaeodoses include a ± 2% systematic uncertainty associated with laboratory beta-source calibrations and uncertainties presented at 68% confidence interval (1σ) | | | | | | | | | | | | | | |  | |  | |  | | |
|  |  | **i** | Uncertainties at 68% confidence interval (1σ) | | | | | | |  | |  | |  | |  | |  | |  | |  | | |

**Table S12**: High resolution gamma spectrometry of selected samples from Southern China

|  |  |  |  |  | |  | |  | |  |  |
| --- | --- | --- | --- | --- | --- | --- | --- | --- | --- | --- | --- |
| **Sample** | **^238^U** | **^226^Ra** | **^210^Pb** | **^228^Ra** | **^228^Th** | | **^40^K** | | **Water** | | **Total** | |
| **code^a^** |  |  |  |  |  | |  | | **content** | | **dose rate^b^** | |
|  | **(Bq/kg)** | **(Bq/kg)** | **(Bq/kg)** | **(Bq/kg)** | **(Bq/kg)** | | **(Bq/kg)** | | **(%)** | | **(Gy ka^-1^)** | |
|  |  |  |  |  |  | |  | |  | |  | |
| **CSHT** | 74 ± 10 | 89.7 ± 7.2 | 82.4 ± 12.0 | 86.9 ± 8.0 | 90.6 ± 8.6 | | 98 ± 21 | | 9 / 10 ± 2 | | 2.865 ± 0.295 | |
|  |  |  |  |  |  | |  | |  | |  | |
| **CBAP** | 66 ± 11 | 57.0 ± 5.0 | 39.4 ± 8.7 | 73.0 ± 7.2 | 75.9 ± 7.4 | | 73 ± 23 | | 7 / 5 ± 2 | | 2.672 ± 0.294 | |
|  |  |  |  |  |  | |  | |  | |  | |
| **CBAX** | 89 ± 12 | 89.2 ± 7.3 | 51.8 ± 9.7 | 48.3 ± 5.6 | 44.6 ± 4.6 | | 74 ± 21 | | 13 / 10 ± 2 | | 2.098 ± 0.265 | |
|  |  |  |  |  |  | |  | |  | |  | |
| **CHEJ** | 38 ± 10 | 29.2 ± 2.7 | 37.7 ± 9.6 | 66.4 ± 5.9 | 72.6 ± 7.5 | | 46 ± 12 | | 7 / 5 ± 2 | | 2.539 ± 0.286 | |

^a^ High resolution gamma spectrometry to estimate the U, Th and K concentrations in the dried and powdered sample, provide a comparison of dose rate, and to test the degree of disequilibrium occurring at the site. HRGS was measured for only four samples. All other parameters such as cosmic dose rate, internal dose rate and number of grains remains the same as Table S11.

^b^ The total dose rate for these samples is slightly higher than Table S11 resulting in a lower age estimates.

**Discussion**

As cave breccias are deposited in notorious environments for partial bleaching, the majority of the resulting single-aliquots and single-grains have been analysing using a minimum age model (MAM). This is justifiable for samples producing high overdispersion values of between 60-80% e.g., Yanliang cave (YAN12,13), which is outside of a normal Gaussian population distribution indicating that partial bleaching has occurred. We attribute this overdispersion to partial bleaching in these samples, thus we have applied the MAM to the majority of samples to take the most conservative approach to age estimation. Surprisingly, a few of the samples produced over dispersion values of between 21-32% for single-aliquots and 25-37% for single-grain, which are considered to be within the range of a normal bleached population. For these samples, such as CYIX14, CBAP3/6 and CXK-5, the MAM could not be applied as it severly underestimated the age according to the results of the independent age estimates. Thus, the CAM was used instead, as justified by the lower overdispersion values, which produced age estimates that agreed with the independent age estimates for those deposits.

These samples were challenging considering the small amounts of sample yield, which meant that for some samples we were unable to get the number of accepted grains to a statistically meaningful number before running out of sample, for example samples CBAP3,4 and CBAX4,5. We were able to analyse most of the samples using the MAM but some of the result relied on only 1-2 aliquots for D_e_ determination, such as CSHT1,11 CHEJ2,3 CYAN13 and CGONG16. However, statistically significant data was obtained from pIR-IRSL single-grain analysis for the CSHT, CHEJ and CGONG samples, and OSL single-grain analysis for the younger CBAX, CLPB and CXK samples. For some samples such as CYIX and CYAN we were unable to proceed with a single-grain investigation and thus these single-aliquot age estimates are viewed as maximum ages.

For the single-grain analysis, many samples also did not yield high number of accepted grains (e.g., CBAX6, QQ1, CZW2, CZS6) but the value of the single-grain approach is that it can be used to isolate individual dose populations rather than an average of a few populations that result in a maximum age. Thus, the age estimate derived from the single-grain method (even with a small number of grains) is much closer to the true burial age of the sediments than the multiple-grain single-aliquot results. For some samples, such as HEJ1,2 and CMF3 the MAM is based on only 1-2 grains, but when used in conjunction with the coeval single-aliquot results and the favourable comparison with independent age estimates the reliability of these results is improved.

Most of the resulting quartz and feldspar age estimates are consistent with their stratigraphic location and independent age estimates within their error margins (within 1 σ). The differing dose saturation


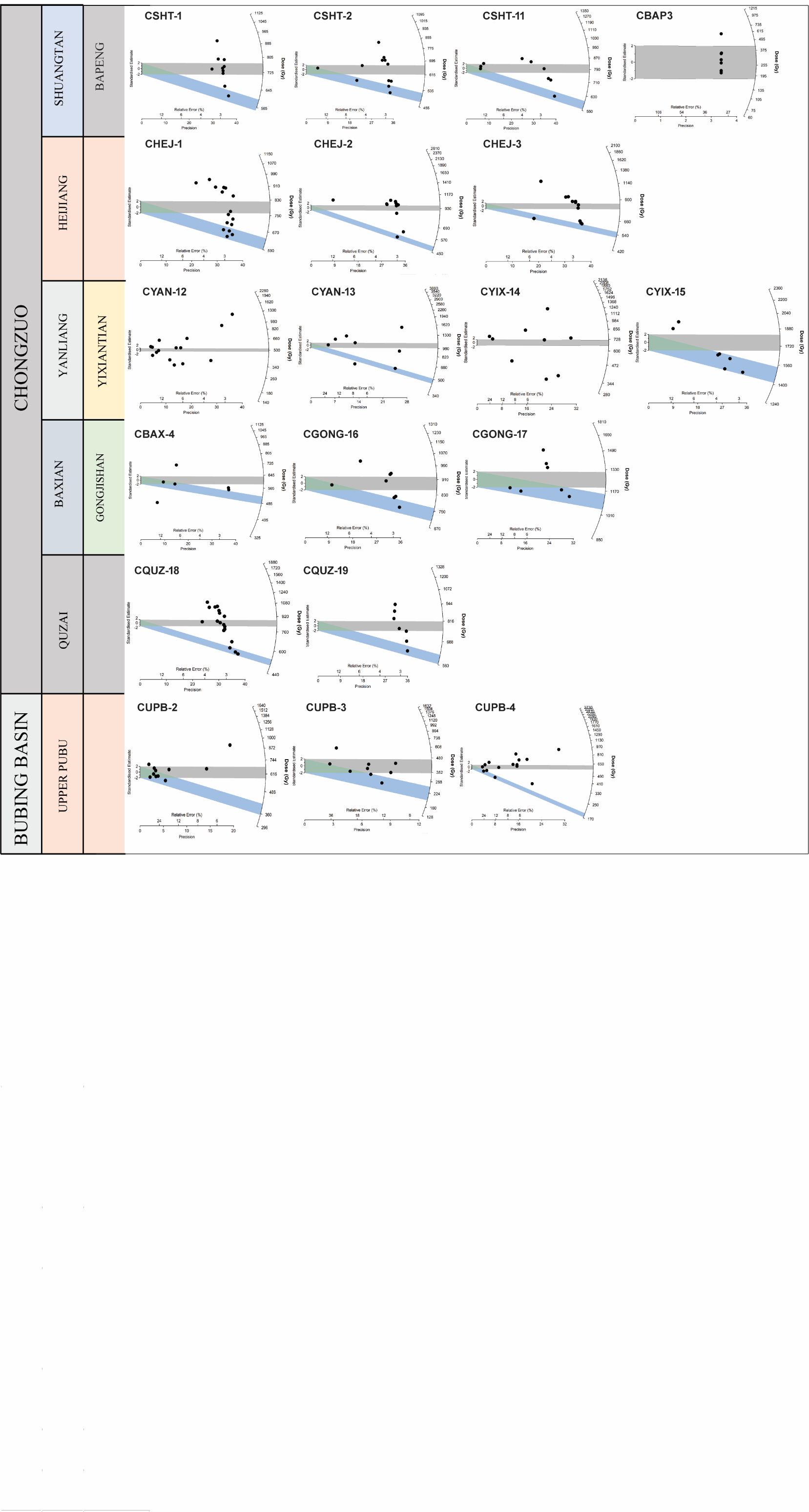


**Fig S4**: **pIR-IRSL single aliquot radial plots** divided into region and cave, the sample names are situated in the left-hand corner of each plot. The horizontal grey banded region denotes a range within 2 sigma of the central point determined using a central age model (CAM). The blue banded region denotes a range within 2 sigma of the minimum age as determined using the minimum age model (MAM). The equivalent dose (D_e_) can be obtained by defining a line that starts on the zero point on the x-axis and projects to the curved axis on the right hand side of the plot, while the uncertainty and precession are read vertically projected onto the y axis. Most of the samples produced single-aliquots results, but the caves that produced decays for all aliquots were selected for further single-grain analysis (Fig. S5)


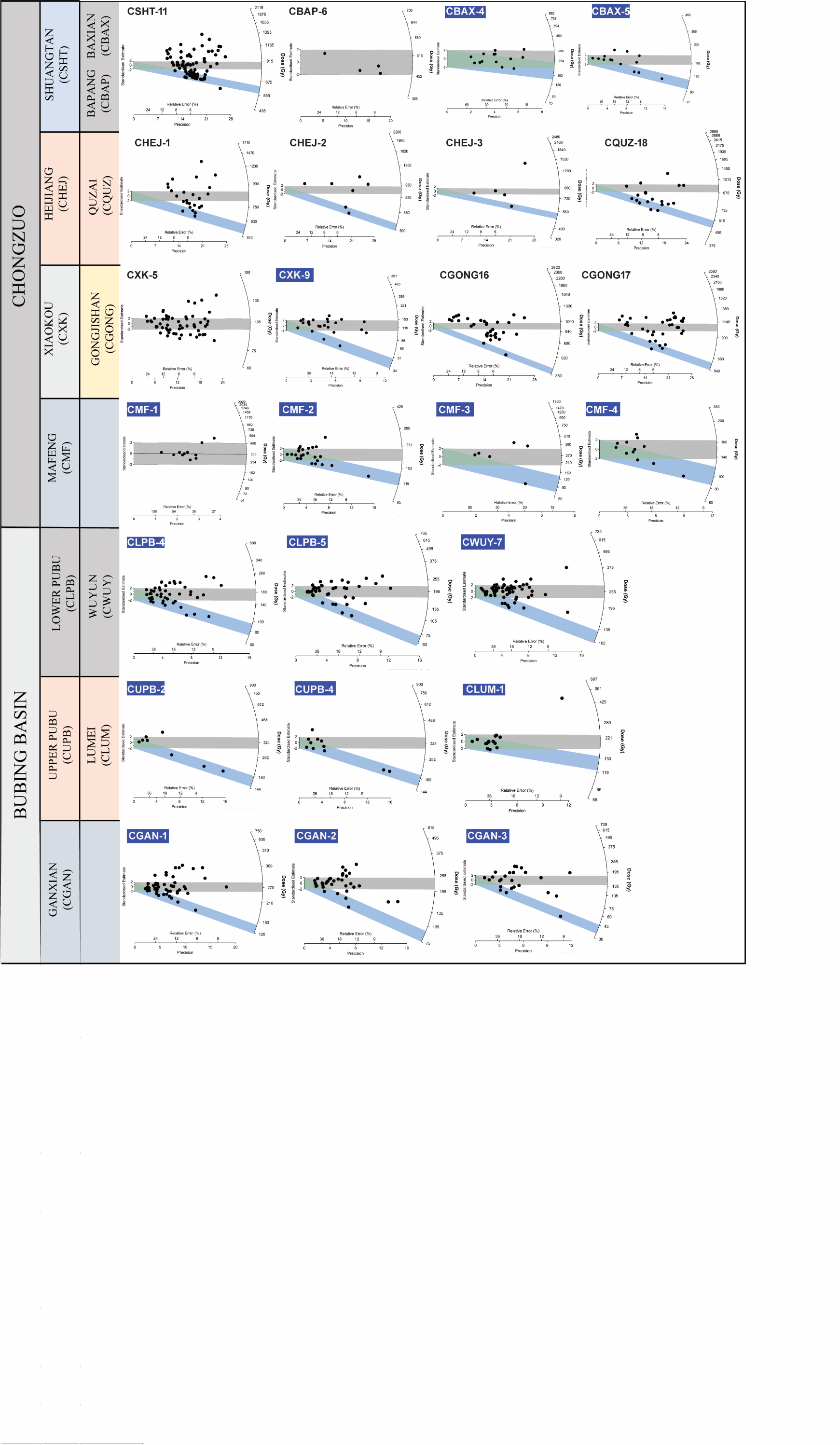


**Fig. S5** - **pIR-IRSL and OSL single-grain radial plots** divided into region and cave, the sample names are situated in the left-hand corner of each plot. The horizontal grey banded region denotes a range within 2 sigma of the central point determined using a central age model (CAM). The blue banded region denotes a range within 2 sigma of the minimum age as determined using the minimum age model (MAM). The OSL single grain results have been denotated by a solid blue background around the sample name.

characteristics between quartz and feldspar were evident in these samples with the single-grain quartz procedures underestimating the independent age estimates, mostly from the overlying flowstones. This was most noticeable in Xiaokou cave with sample CXK-5 underestimating the age of the overlying flowstone by ~40 kyr. Unusually, this unit is a conglomerate rather than the more commonly sampled breccia deposits. This differing composition may have some bearing on the contrasting sample dose characteristics, which appear to saturate ~130 Gy. Alternatively, some of the breccia samples produce quartz single grain age estimates that are coeval with the single-grain feldspar results, for example CUPB3-4, and can easily reach ~240 Gy without saturating. In comparison, the feldspar analysis easily produced D_e_’s in the range 600-1000 Gy with one sample (CYIX-15) exceeding 2000 Gy. This demonstrates the vastly higher dose saturation characteristics of feldspars in comparison to quartz, and makes feldspars the mineral of choice in these high dose rate environments. This is only hampered by the lower feldspar yields in these cave breccias from the dominantly limestone and chert lithology. This limitation creates a precedent for the collection of large volume samples to maximise the feldspar yield.

Some of the older cave sites such as Baikong, Sanhe and Chuifeng were too old for pIR-IRSL feldspar dating. The result for Queque cave (sample QQ1) and Daxin (DAX7) underestimate the coupled US-ESR age for fossils by ~200-300 kyrs suggesting that at this point even feldspars have reached their saturation point and should be viewed as minimum ages. In lower dose rate environments of 1-2 Gy/ka it might be possible to establish ages for samples with large dose saturation characteristics such as seen in Yixiantian cave (CYIX-15). For the older caves we relied on ESR dating of quartz and coupled US-ESR dating of fossils. Daxin cave proved to be the most challenging deposits to establish a reliable chronology – the age range of the cave sediments is thought to span from 1 Ma-200 ka. This age range was too old for quartz OSL and the samples yielded very small quantities of feldspars with limited decays so no luminescence dating results could be obtained. Instead we focused on the ESR dating of quartz from the sediment and coupled US-ESR dating of the fossils.

In all 22 caves sampled the sediment age is slightly younger than the fossil age obtained by coupled US-ESR, but most are coevel within error uncertainties. In some circumstances, organisms die within the cave system and are rapidly encased in the burial sedimements, in which case the difference in age between the fossils and the burial sediments is neglible, however this is not the case with *G. blacki* as they wouldn’t have entered the cave due to their size. A more likely scenario for the *G. blacki* fossils is that after death the bones were present on the landscape for a certain period of time before being transported into the cave systems. This extended time between death and burial would account for the slightly younger sediment ages observed. Within these cave sediments the single-grain values are significantly lower than the single-aliquot results for both the feldspar and quartz results. In some samples, the single-grain age reduces the single-aliquot age estimate by almost half (e.g., samples CGONG16-17). This could indicate a significant averaging effect from the number of grains on the disc, however as the signal is dominated by only 1-2 grains a more likely explanation is a partial bleaching effect, which is also to be expected in cave environments. This occurs because of the difficulty in ensuring that all of the minerals are fully bleached before being transported down through fissures and sinkholes into the cave. Thus, while the single-aliquot results provide a useful ball park figure they must be viewed as maximum ages with the single-grain results indicate the most accurate representation of the time since burial. However, the difference between single-aliquot and single-grain results is more pronounced in some caves (e.g., Gongjishan) than other caves (e.g., Hejiang, Shuangtan and Quzai), where the results for these two techniques are coeval within errors. This is reflected in the higher overdispersion values for Gongjishan (47%) compared to Quzai (32%) and indicates that partial bleaching is not such an issue in the later cave environment. This difference in partial bleaching is related to the origins of the deposits. The Gongjishan sediment is most likely a colluvial deposit that entered the cave via fissures with limited opportunity for bleaching prior to deposition (See Fig S3). In contrast the Quzai deposits have a fluvial origin and were most likely had more opportunity for bleaching in the river bed environment prior to deposition in the cave.

In regards to the dosimetry, the cave with the highest dose rates were MaFeng and Daxin that are both located to the north of Chongzuo close to Leiping (see Fig. 1). The high dose rates (between 3.7-4.2 Gy/ka) are probably related to the weathered granite clasts found inside the breccia deposits. The dose rates across the caves were fairly consistent when considering the differing composition (breccia vs sandy sediments) and minerals used for dosimetry estimation. The three methods employed; 1) alpha and beta counting combination, 2) insitu gamma and beta counting, and 3) high resolution gamma spectrometry produced somewhat similar results. However, the high-resolution gamma spectrometry results (Table S12) are consistently between 0.2-0.3 Gy/ka higher than the alpha/beta and insitu gamma/beta techniques for three of the samples (HEJ, BAP, and SHT) apart from the younger Baxian cave (BAX).

Looking at the high resolution ^238^U and ^232^Th decay chains; three potential disequilibrium states have been identified: 1) in the ^238^U chain a deficiency of 14 and 24% in ^226^Ra compared to ^238^U for samples CSHT and CBAP respectively; 2) a ^210^Pb a deficiency of between 8-42% compared to ^226^Ra for samples CSHT, CBAP, CBAX; and 3) in the ^232^Th chain, a small excess of between 4-8.5% in ^228^Th compared to ^228^Ra for samples CSHT, CBAP and CHEJ. The level of disequilibrium is variable across the samples, with some samples displaying large deficiencies in ^210^Pb compared to ^226^Ra but smaller deficiencies in ^226^Ra compared to ^238^U (CBAX). Overall, the disequilibrium can be explained by water seepage through the cave causing the leaching of the more mobile radionuclides such as uranium, and by radon loss. The variability in disequilibrium can be attributed to the difference in the composition, number and size of the clasts and fossils in the breccia and the subsequent capacity of the breccia to allow water seepage. Shuangtan cave (CSHT) is the most cemented of all the breccias reducing its capacity for water seepage and leaching and subsequently has the lowest deficiencies in the ^238^U and ^232^Th decay chains. In contrast Bapang cave (CBAP) contains an unconsolidated breccia that allows some water seepage and has resulted in deficiencies ^238^U and excesses in the ^232^Th decay chains.

Thorium is thought to be immobile in most sediments^193^, and the short half-lives (t) of the daughter nuclides (^228^Ra = 5.7 yrs and ^228^Th = 1.91 yrs) and ^210^Pb (22 years) means that this effect of excesses in these radionuclides is negligible. The leaching of uranium appears to be more of a problem in Bapeng (CBAP) and Hejiang (CHEJ) caves, but a deficiency of between 14-24% would only have a small effect on the total dose rate and has been incorporated into the error estimate for that sample. Three of the samples (CSHT, CBAP and CBAX) display a loss of radon as represented by a ^210^Pb deficiency compared to ^226^Ra, but the loss is much greater in Bapeng and Baxian than Shuangtan caves. Radon loss was also measured by the thick source alpha counting, and supports this observation with Bapeng and Baxian recording between 1.2-4% radon loss. This loss can be attributed to the presence of larger clasts in the Bapeng and Baxian breccias than found in Shuangtan that has allowed radon loss through the more matrix supported units. Shuangtan also has a higher dose rate (2.6-2.7 Gy/ka) than Bapeng (2.2-2.3 Gy/ka) or Baxian (2.3-2.4 Gy/ka). However, Shuangtan dose contain the highest values of ^40^K in all the samples (double that of Hejiang cave), which could explain the higher dose rate. Limestone clasts could also be a source of the disequilibrium, which are more abundant in Bapeng and Baxian caves. As the mineralogical assemblage of the clay fraction does not vary between samples, the variation in ^226^Ra deficiency could reflect the proportion of limestone clasts in the sedimentary layers. Due to the presence of these clasts we decided to use the insitu gamma and betta counting estimations of dose rate to account for the unseen limestone found within a 30 cm radius of each sample, and we have incorporated an additional 2% into the error estimate for all samples to account for the measured disequilibrium.

Overall the 55 luminescence age estimates provide the backbone for these cave chronologies and will provide vital data to feed into the models for each cave. Without an understanding of the sedimentary context of the burial sediments, the fossil context can prove more challenging to establish. At these caves the sedimentary and fossil context go hand in hand to produce the more accurate data to establish the window of extinction for *G.blacki.*

**Fig S6**: A comparison of luminescence results with independent age estimates (n=157). **a** a comparison of pIR-IRSL single aliquot and single grain results with three independent age estimates; U-series dating of teeth (green circles), U-series dating of calcite (orange circles) and coupled US-ESR dating of teeth (yellow circles) from the same levels in stratigraphic sections. The solid black line represents a one-in-one line, which the majority of samples touch within error margins. **b** the same comparison as seen in (a) but just for pIR-IRSL single-grain results. This plot has the addition of a trend line (blue dashed line) with a trend line R^2^ value to indicate the strength of the trend. **c** the same comparison as seen in (a) but this time for pIR-IRSL single aliquot data with the same trend line. Note that both b and c show a high R^2^ value with the single aliquot results comparison to the independent age estimates slightly weaker than the single grain results as would be expected for a multi-grain technique. Data are presented as mean ages with s.d. at 1σ uncertainties.


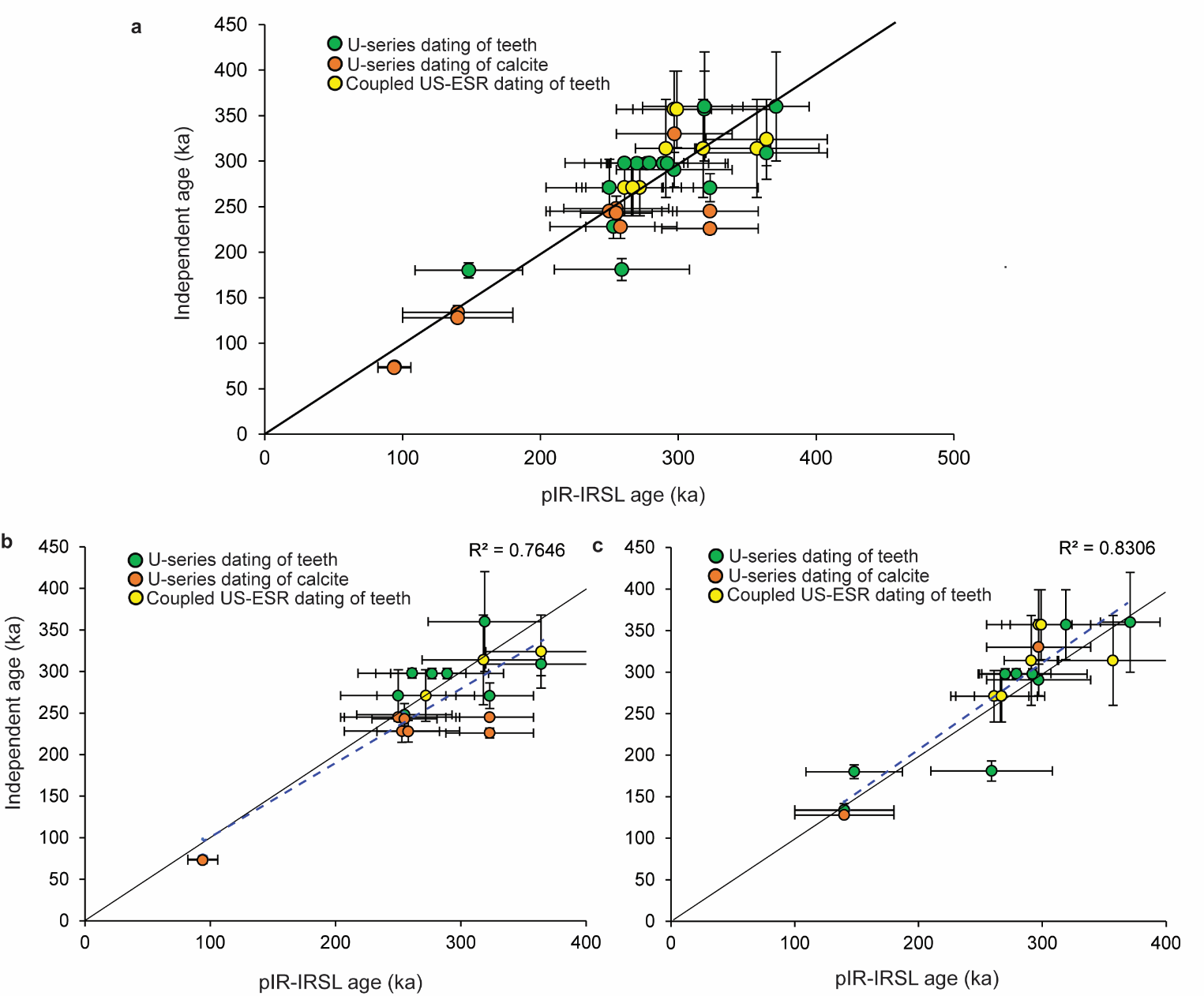


**SI section 5: U-series dating of teeth**

A total of 22 *G. blacki*, 9 *P. weidenrieichi* sp. and 8 faunal fossil teeth were analysed for U-series dating (see Table S13). Teeth were sectioned in half using a high precision slow speed rotary diamond saw, exposing dentine and enamel tissues. The exposed surface of each sample was then polished to >10 microns smoothness to offer a clean ablation surface.

Uranium-series measurements were undertaken by laser ablation Multi Collector-Inductively Coupled Plasma Mass Spectrometer (MC-ICPMS) at the Geoarchaeology and Archaeometry Research Group (GARG) Biomics facility, Southern Cross University. Laser ablation was performed with a New Wave Research 213 nm laser, equipped with a TV2 cell. Thorium (^230^Th, ^232^Th) and uranium (^234^U, ^235^U, ^238^U) isotopes were measured on a Thermo Neptune XT MC-ICPMS mounted with jet sample and x-skimmer cones. All five isotopes were collected in static mode, with both ^234^U and ^230^Th collected in the ion counter and CDD respectively. Helium flow rate and ICP-MS parameters were tuned with NIST610 element standard to derive a ^232^Th/^238^U ratio greater than 0.85 and thus minimising differences in fractionation between Th and U. Tuning was achieved with a fluence of 13.1 J/cm2, pulse rate of 20 Hz, spot size of 110 μm and scan speed of 5 μm/s, yielding 2.27V of ^238^U and 1.98V of ^232^Th on NIST610.

Teeth were ablated using rasters of 5min each (twice ~750 μm long). Before and after each sample, NIST612, MK10 and MK16^194^ standards were measured, as well as a fossil Hippopotamus tooth with known isotopic ratios. ^234^U/^238^U and ^230^Th/^238^U isotopic ratios were corrected for elemental fractionation and Faraday cup/SEM yield by comparison with MK10 coral for which ratios were previously characterised internally by solution analysis. Detrital-corrected ^230^Th-U ages were calculated for each analysis using IsoPlotR^195^ with an assumed detrital (^230^Th/^232^Th) activity ratio of 0.8 ± 0.8. Concentrations of U and Th were determined using NIST612 glass as a calibration standard. Background subtraction, concentration quantification and ratio corrections were performed using Iolite™ software^196^. The corrected (^234^U/^238^U) and (^230^Th/^238^U) isotope ratios for the secondary standard (MK16 coral) within error of the value determined by solution analysis. The hippopotamus tooth was used as a control on matrix effect. Measurements were undertaken with rasters parallel to each other but perpendicular to the buccal lingual direction (from pulp cavity to enamel outer surface). For most teeth a Diffusion-adsorption (D-A) model of U uptake for performed D-A is a computational and mathematical approach used to simulate and analyze the behavior of uranium diffusion in porous materials. This modeling technique describe how substances disperse and evolve over time in response to concentration gradients^197^.

Most teeth show variable uranium distribution, with heterogenous age distribution, uranium accumulation in cracks and along the enamel-dentine junction as well as diffusion hot-spots and leaching zones^198^.

Heijang samples CLMH0904-89 and CLMH0904-93, both from *Gigantopithecus blacki* individuals show very different isotopic values and age estimation. Similar observations can be made for Baikong samples. More than an heterogenous deposition, it is believed that local hydrology within the caves, including proximity to cave walls, would be responsible for the heterogenous diffusion of isotopes within the dental tissues of the samples. This becomes particularly apparent when looking at geochemical map of uranium distribution across the dentine and enamel.

**Table S13 –** Laser ablation U-series dating of *G. blacki* and *P. weidenrieichi* teeth

| **Site** | **Sample name^a^** | **(^232^Th/^238^U)** | **2Std.Err.** | **(^234^U/^238^U)** | **2Std.Err.** | **(^230^Th/^238^U)** | **2Std.Err.** | **Age (kyr)^b^** | **2Std.Err.** |
| --- | --- | --- | --- | --- | --- | --- | --- | --- | --- |
| **Yangliang** | CFLGYL201111-806 | 1.1E-03 | 8.0E-04 | 1.406 | 0.052 | 1.209 | 0.088 | 180.5 | 29.2 |
|  |  | 3.0E-04 | 1.6E-03 | 1.394 | 0.090 | 1.200 | 0.150 | 181.5 | 51.5 |
|  |  | 9.0E-04 | 1.5E-03 | 1.240 | 0.079 | 0.830 | 0.120 | 114.7 | 27.3 |
|  |  | 5.0E-04 | 1.0E-03 | 1.090 | 0.059 | 0.728 | 0.075 | 117.5 | 21.4 |
|  |  | 1.1E-04 | 2.7E-04 | 1.187 | 0.030 | 0.816 | 0.047 | 121.2 | 12.1 |
|  |  | 2.1E-05 | 5.8E-05 | 1.560 | 0.190 | 1.410 | 0.560 | 196.0 | 18.7 |
|  |  | 1.3E-05 | 4.5E-05 | 1.523 | 0.012 | 1.190 | 0.027 | 145.0 | 5.9 |
|  |  | 9.0E-05 | 1.6E-04 | 1.610 | 0.220 | 1.360 | 0.540 | 167.1 | 14.0 |
|  |  | 1.2E-05 | 2.3E-05 | 1.467 | 0.012 | 1.057 | 0.026 | 126.1 | 5.2 |
|  |  | 4.0E-06 | 1.9E-05 | 1.334 | 0.016 | 1.053 | 0.026 | 152.7 | 7.5 |
|  |  | 8.0E-06 | 9.8E-06 | 1.245 | 0.006 | 1.070 | 0.017 | 189.0 | 7.1 |
|  |  | -2.5E-06 | 8.0E-06 | 1.253 | 0.007 | 1.060 | 0.022 | 181.2 | 8.5 |
|  |  | -2.0E-06 | 9.5E-06 | 1.271 | 0.007 | 1.060 | 0.025 | 174.3 | 8.8 |
|  |  | 5.2E-06 | 7.2E-06 | 1.256 | 0.005 | 1.019 | 0.024 | 164.9 | 8.1 |
|  |  | 2.2E-06 | 8.7E-06 | 1.260 | 0.006 | 1.017 | 0.027 | 162.8 | 8.9 |
|  |  | 2.0E-06 | 6.5E-06 | 1.247 | 0.004 | 1.035 | 0.020 | 173.6 | 7.3 |
|  |  | 7.0E-06 | 1.0E-05 | 1.274 | 0.042 | 1.110 | 0.120 | 193.3 | 50.2 |
|  |  | 2.0E-05 | 7.8E-06 | 1.247 | 0.006 | 1.068 | 0.020 | 187.2 | 8.2 |
|  |  | 2.3E-05 | 1.5E-05 | 1.253 | 0.007 | 1.139 | 0.028 | 218.6 | 14.6 |
|  |  | 2.1E-05 | 1.2E-05 | 1.241 | 0.007 | 1.099 | 0.027 | 204.0 | 12.7 |
|  |  | 5.5E-05 | 1.1E-05 | 1.217 | 0.006 | 1.112 | 0.016 | 225.7 | 9.4 |
|  |  | 8.0E-03 | 1.1E-03 | 1.235 | 0.007 | 0.982 | 0.018 | 158.6 | 5.9 |
|  | **CFLGYL201111-806** |  | | | | | | **169.8** | **15.6** |
|  | FLGY201104-Gy-1-3 | -9.0E-05 | 1.9E-04 | 1.400 | 0.024 | 0.788 | 0.031 | 85.9 | 3.5 |
|  |  | 7.0E-05 | 1.6E-04 | 1.419 | 0.033 | 0.893 | 0.062 | 101.4 | 9.5 |
|  |  | 2.4E-04 | 1.5E-04 | 1.419 | 0.018 | 0.929 | 0.068 | 108.0 | 12.3 |
|  |  | 2.3E-04 | 2.5E-04 | 1.253 | 0.011 | 0.964 | 0.037 | 147.6 | 10.8 |
|  |  | 1.2E-03 | 9.1E-04 | 1.259 | 0.026 | 1.070 | 0.012 | 182.8 | 10.6 |
|  |  | 8.9E-05 | 5.4E-05 | 1.248 | 0.010 | 0.951 | 0.027 | 145.1 | 7.6 |
|  |  | 1.1E-04 | 5.0E-05 | 1.238 | 0.009 | 0.901 | 0.022 | 133.2 | 5.5 |
|  |  | 1.5E-04 | 9.7E-05 | 1.233 | 0.008 | 0.928 | 0.028 | 142.1 | 8.0 |
|  |  | 7.1E-05 | 3.3E-05 | 1.223 | 0.008 | 0.907 | 0.020 | 138.3 | 5.3 |
|  | **FLGY201104-Gy-1-3** |  | | | | | | **131.6** | **8.1** |
|  | FLGY201104-GY-1-1 | 1.8E-04 | 9.6E-05 | 1.133 | 0.022 | 0.737 | 0.034 | 111.3 | 8.3 |
|  |  | 9.0E-06 | 1.4E-05 | 1.590 | 0.210 | 1.390 | 0.630 | 180.4 | 17.9 |
|  |  | 1.1E-05 | 1.3E-05 | 1.478 | 0.008 | 1.043 | 0.023 | 121.6 | 4.4 |
|  |  | 1.7E-05 | 1.3E-05 | 1.485 | 0.008 | 1.051 | 0.024 | 121.9 | 4.5 |
|  |  | 1.0E-05 | 6.9E-06 | 1.417 | 0.016 | 1.029 | 0.026 | 128.7 | 5.7 |
|  |  | 6.4E-06 | 5.9E-06 | 1.269 | 0.005 | 1.100 | 0.024 | 191.2 | 10.0 |
|  |  | 5.8E-06 | 3.8E-06 | 1.274 | 0.004 | 1.061 | 0.018 | 173.7 | 6.4 |
|  |  | 1.0E-05 | 1.2E-05 | 1.287 | 0.015 | 1.085 | 0.036 | 177.9 | 13.0 |
|  |  | 5.9E-06 | 4.1E-06 | 1.258 | 0.004 | 0.991 | 0.017 | 154.8 | 5.2 |
|  |  | 2.4E-05 | 1.8E-05 | 1.244 | 0.004 | 0.964 | 0.019 | 150.0 | 5.8 |
|  |  | 1.2E-05 | 2.7E-06 | 1.244 | 0.005 | 0.984 | 0.020 | 156.6 | 6.4 |
|  |  | 3.9E-05 | 5.7E-06 | 1.244 | 0.006 | 1.066 | 0.024 | 187.6 | 9.9 |
|  |  | 2.1E-04 | 1.1E-04 | 1.222 | 0.006 | 1.064 | 0.026 | 196.5 | 11.9 |
|  | **FLGY201104-GY-1-1** |  | | | | | | **157.9** | **8.4** |
| **Baikong** | CLBBD201011-1127 | -1.0E-05 | 1.2E-04 | 1.750 | 0.210 | 1.610 | 0.180 |  |  |
|  |  | 6.0E-04 | 1.4E-03 | 2.020 | 0.180 | 1.890 | 0.260 |  |  |
|  |  | 2.1E-04 | 4.0E-04 | 2.153 | 0.044 | 1.730 | 0.066 | 143.9 | 7.4 |
|  |  | -5.5E-05 | 7.3E-05 | 2.195 | 0.044 | 1.878 | 0.064 | 161.2 | 7.7 |
|  |  | 4.3E-05 | 5.7E-05 | 2.513 | 0.009 | 2.223 | 0.023 | 168.6 | 3.2 |
|  |  | 2.9E-06 | 7.0E-06 | 2.516 | 0.006 | 2.217 | 0.017 | 167.2 | 2.3 |
|  |  | 1.0E-05 | 8.7E-06 | 2.514 | 0.007 | 2.265 | 0.016 | 174.9 | 2.2 |
|  |  | 2.1E-05 | 2.0E-05 | 2.505 | 0.007 | 2.273 | 0.012 | 177.4 | 1.5 |
|  | **CLBBD201011-1127** |  |  |  |  |  |  | **165.5** | **4.1** |
|  | CLBBD201011-MT35 | 4.0E-03 | 1.1E-02 | 2.200 | 0.360 | 0.930 | 0.950 |  |  |
|  |  | 1.1E-02 | 2.0E-02 | 0.900 | 0.670 | -1.900 | 5.100 |  |  |
|  |  | -4.0E-03 | 3.2E-02 | -1.200 | 2.100 | -1.000 | 23.000 |  |  |
|  |  | -3.7E-02 | 6.6E-02 | -1.900 | 1.500 | -2.400 | 5.500 |  |  |
|  |  | 1.6E-05 | 1.3E-05 | 2.475 | 0.005 | 2.304 | 0.017 | 187.6 | 2.8 |
|  |  | 1.3E-05 | 7.3E-06 | 2.471 | 0.004 | 2.276 | 0.015 | 183.4 | 2.4 |
|  |  | 7.0E-06 | 6.6E-06 | 2.470 | 0.004 | 2.303 | 0.020 | 188.4 | 3.4 |
|  |  | 1.2E-05 | 2.1E-05 | 2.467 | 0.006 | 2.289 | 0.020 | 186.4 | 3.3 |
|  | **CLBBD201011-MT35** |  |  |  |  |  |  | **186.4** | **3.0** |
|  | CLBBD201011-1179 | 2.1E-03 | 5.2E-03 | 1.110 | 0.240 |  |  |  |  |
|  |  | 2.3E-03 | 2.2E-03 | 1.160 | 0.120 |  |  |  |  |
|  |  | 1.7E-04 | 5.0E-04 | 1.402 | 0.069 |  |  |  |  |
|  |  | 1.9E-05 | 1.4E-05 | 1.761 | 0.010 | 1.911 | 0.023 | 326.2 | 15.7 |
|  |  | 2.2E-05 | 2.9E-05 | 1.780 | 0.005 | 1.899 | 0.016 | 302.3 | 9.4 |
|  |  | 8.2E-05 | 9.4E-05 | 1.771 | 0.006 | 1.860 | 0.016 | 286.3 | 8.2 |
|  |  | 2.0E-05 | 1.7E-05 | 1.769 | 0.005 | 1.835 | 0.014 | 274.1 | 6.7 |
|  |  | 1.6E-06 | 5.0E-06 | 1.770 | 0.004 | 1.855 | 0.014 | 283.9 | 7.2 |
|  | **CLBBD201011-1179** |  |  |  |  |  |  | **294.5** | **9.4** |
|  | CLBBD201011-858 | 1.4E-05 | 5.4E-05 | 1.948 | 0.034 | 1.680 | 0.034 | 167.2 | 0.4 |
|  |  | 1.2E-05 | 4.3E-05 | 1.751 | 0.043 | 1.607 | 0.093 | 196.1 | 23.6 |
|  |  | 3.5E-05 | 5.0E-05 | 2.100 | 0.190 | 1.910 | 0.190 | 184.0 | 0.2 |
|  |  | 2.1E-05 | 5.1E-05 | 2.178 | 0.066 | 2.024 | 0.075 | 191.4 | 7.4 |
|  |  | 1.5E-05 | 1.2E-05 | 2.798 | 0.007 | 2.817 | 0.038 | 219.1 | 7.0 |
|  |  | 2.2E-05 | 1.3E-05 | 2.805 | 0.009 | 2.688 | 0.048 | 195.6 | 7.5 |
|  |  | 1.3E-05 | 1.0E-05 | 2.797 | 0.010 | 2.751 | 0.024 | 207.4 | 3.8 |
|  |  | 2.1E-05 | 1.3E-05 | 2.787 | 0.010 | 2.725 | 0.021 | 204.7 | 3.2 |
|  | **CLBBD201011-858** |  |  |  |  |  |  | **195.7** | **6.6** |
|  | CLBBD201011-1064 | 2.4E-04 | 5.2E-04 | 1.476 | 0.080 | 1.160 | 0.110 | 147.6 | 18.7 |
|  |  | -3.0E-05 | 5.1E-05 | 1.709 | 0.054 | 1.432 | 0.066 | 162.1 | 8.7 |
|  |  | -3.0E-06 | 2.2E-05 | 1.777 | 0.050 | 1.517 | 0.055 | 166.9 | 5.2 |
|  |  | -1.3E-05 | 2.3E-05 | 1.760 | 0.037 | 1.541 | 0.046 | 176.3 | 6.7 |
|  |  | 2.2E-05 | 1.1E-05 | 1.704 | 0.003 | 1.766 | 0.008 | 278.4 | 3.9 |
|  |  | 1.5E-05 | 7.0E-06 | 1.708 | 0.003 | 1.799 | 0.009 | 295.3 | 5.5 |
|  |  | 1.6E-05 | 8.1E-06 | 1.710 | 0.003 | 1.792 | 0.011 | 289.4 | 6.3 |
|  |  | 8.7E-06 | 5.1E-06 | 1.712 | 0.003 | 1.791 | 0.010 | 287.3 | 5.5 |
|  | **CLBBD201011-1064** |  |  |  |  |  |  | **225.4** | **7.5** |
|  | **CLBBD201011-859** | 6.9E-04 | 3.5E-06 | 2.552 | 0.010 | 2.348 | 0.012 | **182.1** | **1.3** |
| **Hejiang** | CLMH0904-89 | 3.5E-05 | 3.1E-05 | 1.498 | 0.021 | 0.824 | 0.016 | 82.5 | 7.0 |
|  |  | 1.9E-05 | 1.7E-05 | 1.721 | 0.015 | 1.012 | 0.044 | 89.6 | 5.4 |
|  |  | 5.9E-06 | 6.3E-06 | 1.744 | 0.020 | 1.038 | 0.051 | 91.1 | 6.1 |
|  |  | 2.2E-06 | 3.5E-06 | 1.690 | 0.009 | 0.947 | 0.028 | 83.8 | 3.4 |
|  |  | 5.9E-06 | 2.2E-06 | 1.517 | 0.005 | 0.923 | 0.015 | 95.5 | 2.3 |
|  |  | 7.4E-06 | 1.7E-06 | 1.522 | 0.006 | 0.946 | 0.011 | 98.7 | 1.6 |
|  |  | 7.4E-06 | 1.9E-06 | 1.526 | 0.004 | 0.972 | 0.010 | 102.4 | 1.5 |
|  |  | 9.9E-06 | 1.7E-06 | 1.533 | 0.003 | 0.988 | 0.012 | 104.2 | 1.9 |
|  |  | 7.2E-06 | 1.2E-06 | 1.538 | 0.005 | 0.989 | 0.012 | 103.8 | 1.8 |
|  |  | 3.0E-05 | 3.4E-05 | 1.541 | 0.008 | 1.009 | 0.023 | 106.8 | 3.6 |
|  |  | 1.2E-05 | 1.5E-06 | 1.536 | 0.003 | 0.987 | 0.009 | 103.8 | 1.4 |
|  |  | 2.5E-05 | 3.3E-06 | 1.537 | 0.004 | 1.001 | 0.021 | 105.9 | 3.4 |
|  |  | 3.3E-05 | 2.3E-06 | 1.536 | 0.003 | 0.981 | 0.006 | 102.7 | 0.9 |
|  |  | 5.0E-05 | 6.9E-06 | 1.531 | 0.004 | 0.977 | 0.013 | 102.7 | 2.0 |
|  | **CLMH0904-89** |  |  |  |  |  |  | **98.1** | **3.0** |
|  | **CLMH0904-93** | 1.9E-03 | 6.7E-06 | 1.660 | 0.006 | 1.744 | 0.006 | **297.6** | **3.1** |
| **Bapeng** | BAPENG-6 | 4.3E-04 | 3.3E-04 | 1.906 | 0.042 | 1.740 | 0.011 | 189.5 | 9.6 |
|  |  | 6.0E-05 | 1.0E-04 | 2.128 | 0.014 | 2.448 | 0.004 | 376.1 | 14.0 |
|  |  | 7.7E-05 | 6.3E-05 | 2.139 | 0.025 | 2.462 | 0.009 | 375.8 | 25.1 |
|  |  | 1.7E-04 | 1.6E-04 | 2.045 | 0.014 | 2.207 | 0.004 | 293.2 | 7.6 |
|  | **BAPENG-6** |  |  |  |  |  |  | **308.7** | **14.1** |
|  | **BAPENG-18** | 2.4E-04 | 1.3E-04 | 2.150 | 0.017 | 2.240 | 0.006 | **180.1** | **8.2** |
| **Shuangtan** | CMLST0911-109 | 5.7E-05 | 3.8E-05 | 1.027 | 0.043 | 0.780 | 0.110 | 153.7 | 44.0 |
|  |  | 2.0E-05 | 1.0E-04 | 1.075 | 0.023 | 0.716 | 0.027 | 117.4 | 7.8 |
|  |  | 2.0E-06 | 1.1E-06 | 1.057 | 0.003 | 0.928 | 0.011 | 219.7 | 7.8 |
|  |  | 2.5E-06 | 1.1E-06 | 1.050 | 0.002 | 0.948 | 0.015 | 241.9 | 13.2 |
|  |  | 2.5E-06 | 9.8E-07 | 1.051 | 0.002 | 0.931 | 0.017 | 226.2 | 13.0 |
|  |  | 2.3E-06 | 7.0E-07 | 1.056 | 0.002 | 0.913 | 0.018 | 209.3 | 11.8 |
|  |  | 4.3E-06 | 1.2E-06 | 1.065 | 0.002 | 0.922 | 0.015 | 209.2 | 9.5 |
|  |  | 9.7E-06 | 1.4E-06 | 1.054 | 0.003 | 0.929 | 0.019 | 222.1 | 13.9 |
|  |  | 3.9E-04 | 1.7E-05 | 1.019 | 0.004 | 0.931 | 0.014 | 260.8 | 15.3 |
|  |  | 8.9E-03 | 2.1E-03 | 1.023 | 0.026 | 0.677 | 0.053 | 117.5 | 15.8 |
|  | **CMLST0911-109** |  |  |  |  |  |  | **197.8** | **15.2** |
|  | **CMLST0911-50** | 2.1E-04 | 9.6E-07 | 1.297 | 0.006 | 1.230 | 0.006 | **243.3** | **2.9** |
| **Zhanwang** | O1Z1 | 8.6E-03 | 5.0E-03 | 1.546 | 0.015 | 1.589 | 0.030 | 286.5 | 18.1 |
|  |  | 8.6E-03 | 5.6E-03 | 1.536 | 0.021 | 1.554 | 0.019 | 271.6 | 14.1 |
|  |  | 4.1E-03 | 2.4E-03 | 1.542 | 0.024 | 1.530 | 0.021 | 254.1 | 13.6 |
|  | **O1Z1** |  |  |  |  |  |  | **270.8** | **15.3** |
|  | **O1Z2** | 2.0E-02 | 9.4E-03 | 1.411 | 0.074 | 1.333 | 0.049 | **230.0** | **27.8** |
| **Queque** | **CSQZ0811-64** | 2.2E-03 | 7.4E-06 | 2.668 | 0.007 | 2.357 | 0.008 | **166.7** | **0.7** |
| **Sanhe** | **GCSD0410-252** | 2.4E-03 | 8.5E-05 | 2.311 | 0.008 | 1.970 | 0.007 | **158.8** | **5.3** |
|  | **GCSD0410-256** | 7.3E-04 | 2.7E-06 | 2.210 | 0.008 | 1.783 | 0.007 | **144.4** | **0.7** |
|  | **GCSD0410-225** | 2.0E-04 | 3.2E-04 | 2.260 | 0.011 | 1.747 | 0.008 | **141.1** | **3.4** |
| **Baxian*** | **RTK201306-77** | 8.6E-05 | 6.3E-05 | 1.880 | 0.024 | 2.055 | 0.069 | **156.7** | **8.2** |
|  | **RTK201306-103** | 3.3E-03 | 8.3E-04 | 2.021 | 0.018 | 2.410 | 0.069 | **133.9** | **7.7** |
|  | **RTK201306_45** |  |  |  |  |  |  |  |  |
| **Daxin*** | DLZNH201211-1-28 | -0.0000347 | 0.0000066 | 1.293 | 0.0045 | 1.344 | 0.02 | 361.1 | 17.2 |
|  | DLZNH201211-1-28 | -0.000033 | 0.000012 | 1.3229 | 0.0045 | 1.428 | 0.022 | 465 | 40.3 |
|  | DLZNH201211-1-28 | -0.0000242 | 0.0000095 | 1.323 | 0.0056 | 1.427 | 0.0095 | 461 | 20.9 |
|  | **DLZNH201211-1-28** |  | | | | | | **429.0** | **26.2** |
|  | DLZNH201211-1-13 | -1.4E-05 | 9.5E-06 | 1.552 | 0.007 | 1.313 | 0.014 | 169.0937 | 4.2163 |
|  | DLZNH201211-1-13 | -3.0E-05 | 1.0E-05 | 1.553 | 0.006 | 1.344 | 0.018 | 177.5057 | 5.5031 |
|  | DLZNH201211-1-13 | -2.2E-05 | 9.2E-06 | 1.564 | 0.006 | 1.352 | 0.016 | 176.8032 | 4.8262 |
|  | DLZNH201211-1-13 | -2.4E-05 | 8.2E-06 | 1.567 | 0.006 | 1.377 | 0.017 | 183.2321 | 5.3557 |
|  | DLZNH201211-1-13 | -1.9E-05 | 8.0E-06 | 1.565 | 0.006 | 1.382 | 0.011 | 185.5317 | 3.8642 |
|  | DLZNH201211-1-13 | -2.5E-05 | 6.9E-06 | 1.563 | 0.006 | 1.377 | 0.015 | 184.5547 | 4.9124 |
|  | DLZNH201211-1-13 | -2.3E-05 | 6.7E-06 | 1.560 | 0.008 | 1.369 | 0.014 | 182.976 | 4.8184 |
|  | DLZNH201211-1-13 | -2.4E-05 | 9.3E-06 | 1.564 | 0.005 | 1.371 | 0.018 | 182.4454 | 5.6144 |
|  | DLZNH201211-1-13 | -2.9E-05 | 1.3E-05 | 1.562 | 0.005 | 1.388 | 0.013 | 188.1028 | 4.3284 |
|  | DLZNH201211-1-13 | -2.9E-05 | 7.3E-06 | 1.573 | 0.007 | 1.389 | 0.015 | 185.199 | 5.0295 |
|  | DLZNH201211-1-13 | -3.1E-05 | 6.7E-06 | 1.573 | 0.007 | 1.385 | 0.020 | 183.843 | 6.3207 |
|  | DLZNH201211-1-13 | -2.3E-05 | 1.5E-05 | 1.568 | 0.008 | 1.371 | 0.019 | 181.1241 | 6.0107 |
|  | **DLZNH201211-1-13** |  |  |  |  |  |  | **181.7** | **5.1** |
|  | **DLZNH201211-740** | -3.2E-06 | 1.0E-05 | 1.669 | 0.006 | 1.919 | 0.010 | **527.8** | **34.2** |
|  | **DLZNH201211-15** | 1.8E-06 | 7.4E-06 | 1.440 | 0.005 | 1.546 | 0.007 | **379.5** | **18.7** |
|  | **DLZNH201211-6** | -4.7E-06 | 1.6E-05 | 1.337 | 0.007 | 1.010 | 0.015 | **140.4** | **3.6** |
|  | **DLZNH201211-797** | -1.4E-05 | 1.7E-05 | 1.548 | 0.007 | 1.305 | 0.013 | **168.0** | **3.0** |
| **Shuangtan**** | **Cervid 278** | 9.1E-05 | 7.1E-05 | 1.203 | 0.013 | 1.129 | 0.022 | **248.0** | **13.5** |
|  | **Cervid 279** | 1.0E-04 | 5.9E-04 | 1.281 | 0.041 | 1.172 | 0.125 | **228.9** | **21.6** |
|  | **Cervid 286** | 1.4E-04 | 9.2E-05 | 1.139 | 0.011 | 1.031 | 0.022 | **228.6** | **13.7** |
|  | **Cervid 287** | 1.1E-03 | 9.5E-03 | 1.288 | 0.176 | 0.300 | 0.470 |  |  |
|  | **Suid 258** | 1.0E-04 | 2.2E-04 | 1.131 | 0.010 | 1.089 | 0.018 | **290.6** | **19.1** |
|  | **Suid 279** | -3.9E-05 | 5.2E-05 | 1.265 | 0.021 | 1.146 | 0.025 | **214.9** | **6.3** |
|  | **Suid 281** | 3.3E-06 | 1.4E-05 | 1.046 | 0.005 | 1.032 | 0.012 | **389.1** | **37.1** |
|  | **Suid 287** | -9.8E-06 | 7.4E-05 | 1.276 | 0.014 | 1.038 | 0.025 | **164.9** | **7.0** |

^a^ Rows highlighted in grey represent the average U-series age estimation for the tooth

^b^ Cells were left empty when measured values did not permit to calculate a meaningful U-series age for this specific raster.

**SI section 6: Coupled US-ESR dating of teeth**

Enamel fragments from each tooth dated by coupled US-ESR technique were separated using a hand-held diamond saw following the protocol developed by^38^ and stripped of the outer ~100 µm ±10% on each side. Fragments were mounted into a parafilm mould within a Teflon sample holder to record the angular dependency in the ESR response^199,200^. Fragments were then measured at room temperature on a Freiberg MS5000 ESR X-band spectrometer at a 0.1mT modulation amplitude, 10 scans, 2mW power, 100G sweep, and 100KHz modulation frequency. Irradiation was performed with the Freiberg X-ray irradiation chamber for fragments, which contains a Varian VF50 X-ray gun at a voltage of 40 kV and 0.5 mA current on the fragment exposed to X-rays without shielding (apart from a 200 µm Al foil layer)^201^. Each fragment was irradiated, following exponentially increasing irradiation times (around 90, 380, 900, 1800, 3600, 7200, 14400, 25000 and 50000 s, although the exact irradiation time and dose rate varies for each sample). For each irradiation step, the energy output of the X-ray gun is recorded at the beginning and end and averaged, which allows it to correct for the dose rate received by the sample. For each irradiation step the fragment was measured over 180^o^ in x, y and z-configurations with a 20^o^ step^199-200^. Powder samples were obtained by crushing extracted enamel fragments into a powder sieved to keep only 100-200 µm grain size fraction. Each sample (see Table S14) was then divided in 8 distinct aliquots* of >50 mg each and send to the Australian Nuclear Science and Technology Organisation for gamma irradiation with a Gammacell 220 (Cobalt 60) (Dose rate 10.2Gy/min, 50.3Gy, 101Gy, 254Gy, 450Gy, 611Gy, 1170Gy, 2420Gy, 3970Gy, and 7990Gy)[*An additional dose of 14,890Gy was given to the samples, however this dose was not used for the Dose Response Curve (DRC) calculation].

ESR intensities were extracted from T1-B2 peak-to-peak amplitudes on the merged ESR signal or the powder spectra^200^. Isotropic and baseline corrections were applied uniformly across the measured spectra for fragment measurements^202^. The amount of unstable non-orientated CO_2_ radicals (NOCORs) was estimated using the protocol described in^200^. The ESR dose response curves were obtained using merged ESR intensities of all orientation and associated standard deviations from the repeated measurements over one orientation only. For powder spectra associated standard deviations were obtained using repeated measurements after shaking the tube at each irradiation step.

Fitting procedures were carried out with the MCDOSE 2.0 software using a Markov Chain Monte Carlo (MCMC) approach based on the Metropolis-Hastings algorithm^69^. D_e_ values were obtained by fitting a single saturating exponential (SSE) at the appropriate maximum irradiation dose (Dmax) following the recommendations of ^203^. The equivalent dose values are summarized in Table S14.

Sediment elemental concentrations, external beta and gamma dose rate contributions, and water content are shown in Table S14. The external beta dose rates have been extrapolated from the U, Th and K contents measured on a portion of sediment sub-sample (~8 g). The external gamma dose rates were assessed using a portable gamma spectrometer at each site, employing the threshold method (refer to the similar luminescence methods in Supplementary Section 3). These measurements were conducted as closely as possible to the locations where the teeth were originally excavated when known. Consequently, there may be some variations between gamma dosimetry utilized for ESR modelling and luminescence calculations. For detailed reference, all US-ESR values are comprehensively documented in Table S14. The cosmic dose rate was estimated at each site according to ^36^, taking into account the altitude, geomagnetic latitude, density of sediment overburden, and the time-averaged geometry of bedrock overburden.

The internal dose rate was calculated from the measurements obtained on each of the enamel fragments and surrounding dentine by laser ablation, using an ESI NW193 ArF Excimer laser coupled to a MC-ICPMS Neptune XT at Southern Cross University. Both enamel and dentine were measured using rasters (see U-series dating paragraph above for parameters). Concentration and isotopic ratio for the enamel of each sample was measured directly on the fragment used, while for the dentine all measurements were averaged to obtain one value for the entire tooth. Equilibrium of the decay chain after ^230^Th in both dental tissues was assumed. See U-series methods for baseline and drifts correction, as the same methods was applied. Both coupled US-ESR^70^ and DATA^204^ programs were used for age results estimation. All age calculations presented here were carried out with the coupled US-ESR program, which utilizes the dose rate conversion factors of ^34^. Additionally, ESR ages were recalculated using the DATA program to compare results especially for closed-system calculation (CSUS-ESR age estimation).

Some of the teeth analysed presented elevated Uranium concentration in the enamel, but always below the 10 ppm threshold. Nonetheless, the high content (see Table S14) in the dental tissues for certain fossils (e.g. CLBBD201011-858, CMLST0911-109) could explain the discrepancy between ages from the same site.

Some teeth appear to show partial uranium leaching in the dentine, with heterogenous distribution across the tissue. By averaging the U-series values, including isotopic ratios, the impact of localised leaching is minimised or even cancelled. Yet, several teeth remain problematic, including Baikong CLBBD201011-858 and Zhanwang B1MF for which both late uranium uptake and complex leaching uptake pattern can be observed respectively. We are unable to correct for either processes, and therefore the ages presented are problematic, making Baikong 858 too young with a late and important uranium uptake, and making B1MF older with uranium leaching during the burial process. Additionally, Zhanwang offers an heterogenous environment, with complex depositional context. It is therefore our opinion that O1Z1 and O1Z2 are more likely representing the true age of the deposit.

We have also investigated the potential variations of water content within the dental tissues (comparing 0% to 3% in enamel and 3% to 8% in dentine, respectively), and we found that the impact on the age calculation was marginal, with ages statistically indistinguishable from one another. Additionally, we have also considered the impact of disequilibrium in the decay chain after ^230^Th. While we have assumed a ratio of 1 after thorium, the degassing of Rn^222^ is a possibility, which could potentially have shifted some of our results to older ages. We have calculated ages with a potential 50% radon degassing in dentine and then in both dental tissues (although Rn loss is known to be atypical in enamel). We have selected three sites that had a great variation of ages, dosimetry and uranium content, such as Bapeng [equilibrium = 329+/-45 ka; assumed 50% Rn loss in Dentine = 341+/-48 ka; and assumed 50% Rn loss both dental tissues = 420+/-47 ka], Baxian [equilibrium =181+/-13 ka; assumed 50% Rn loss in Dentine = 183+/-12 ka; and assumed 50% Rn loss in both dental tissues = 192+/-12 ka]; and Yanliang [equilibrium = 784+/-252 ka; assumed 50% Rn loss in Dentine = 793+/-252 ka; and assumed 50% Rn loss both dental tissues = 858+/-256 ka]. As expected, the Rn loss increases slightly the calculated age, especially when considering a degassing in both tissues (unlikely). Regardless, the calculation shows, that impact of Rn loss (dentine) to be consistently within error of our age estimations when assuming equilibrium.

**Table S14 -** Coupled US-ESR dating of fossil *G. blacki*, *P. weidenreichi* and faunal teeth

| **Site** | **Baikong** | | **Bapeng** | **Queque** | **Sanhe** | | **Shuangtan** | | **Hejiang** | **Yanliang** | **Zhanwang** | | | **Baxian^c^** | | |
| --- | --- | --- | --- | --- | --- | --- | --- | --- | --- | --- | --- | --- | --- | --- | --- | --- |
| ***Sample*** | ***CLBBD201011***  ***-858*** | ***CLBBD201011***  ***-859*** | ***BAPENG***  ***-18*** | ***CSQZ0811***  ***-64*** | ***GCSD0410***  ***-252*** | ***GCSD0410***  ***-225*** | ***CMLST0911***  ***-109*** | ***CMLST0911-50*** | ***CLMH0904-89 (PA1573.9)*** | ***CFLGYL201111***  ***-806*** | ***O1Z1*** | ***O1Z2*** | ***B1MF*** | | ***RTK201306-77***  ***(Pongo)*** | |
| ***Enamel*** | | | | | | | | | | | | | | | |
| **Dose (Gy)^a^** | 1910 ± 72 | 1978 ± 88 | 733 ± 46 | 1667 ± 96 | 1153 ± 38 | 1543 ± 232 | 896 ± 43 | 351 ± 17 | 1422 ± 88 | 1007 ± 255 | 1129 ± 64 | 1147 ± 44 | 2143 ± 154 | | 232.7 ± 7.6 | |
| **U (ppm)^b^** | 9.7 ± 1.1 | 4.2 ± 1 | 5.8 ± 0.3 | 2.6 ± 0.09 | 1.21 ± 0.84 | 3.2 ± 1 | 7.3 ± 0.2 | 3.1 ± 0.1 | 7.3 ± 1.2 | 4.6 ± 0.4 | 0.2 ± 0.1 | 0.1 ± 0.1 | 2.3 ± 0.5 | | 4.32 ± 0.47 | |
| **^234^U/^238^U^b^** | 2.47 ± 0.009 | 1.862 ± 0.01 | 1.721 ± 0.08 | 1.915 ± 0.041 | 2.32 ± 0.009 | 2.485 ± 0.1 | 1.315 ± 0.039 | 1.285 ± 0.002 | 1.58 ± 0.009 | 1.4093 ± 0.088 | 1.54 ± 0.15 | 1.37 ± 0.15 | 1.22 ± 0.17 | | 1.575 ± 0.04 | |
| **^230^Th/^234^U^b^** | 0.8285 ± 0.029 | 0.9485 ± 0.035 | 0.63 ± 0.043 | 0.725 ± 0.002 | 0.5955 ± 0.004 | 0.834 ± 0.11 | 0.689 ± 0.002 | 0.611 ± 0.011 | 0.95 ± 0.029 | 0.5886 ± 0.043 | 0.394 ± 0.082 | 0.435 ± 0.065 | 0.9148 ± 0.076 | | 0.2789 ± 0.002 | |
| **Thickness (m)** | 2050 ± 256 | 1600 ± 190 | 1873 ± 244 | 1950 ± 190 | 1580 ± 140 | 2108 ± 299 | 1700 ± 190 | 2346 ± 283 | 1620 ± 150 | 2356 ± 285 | 1580 ± 210 | 2465 ± 320 | 2930 ± 290 | | 1800 ± 210 | |
| ***Dentine*** | | | | | | | | | | | | | | | |
| **U (ppm)^b^** | 42.7 ± 0.25 | 49.2 ± 0.7 | 18.1 ± 1.4 | 35.3 ± 0.12 | 28.1 ± 0.16 | 17.2 ± 1 | 81.4 ± 0.46 | 66.2 ± 0.2 | 53.4 ± 0.26 | 26.7 ± 1.4 | 10.3 ± 0.4 | 8.7 ± 0.5 | 38.9 ± 1.3 | | 50.5 ± 0.41 | |
| **^234^U/^238^U^b^** | 2.552 ± 0.01 | 2.418 ± 0.01 | 2.282 ± 0.009 | 2.668 ± 0.007 | 2.21 ± 0.008 | 2.26 ± 0.011 | 1.297 ± 0.006 | 1.293 ± 0.008 | 1.66 ± 0.006 | 1.2738 ± 0.009 | 1.541 ± 0.02 | 1.52 ± 0.02 | 1.266 ± 0.009 | | 1.083 ± 0.004 | |
| **^230^Th/^234^U^b^** | 0.92 ± 0.11 | 0.933 ± 0.15 | 0.901 ± 0.043 | 0.8835 ± 0.008 | 0.8069 ± 0.007 | 0.778 ± 0.11 | 0.9484 ± 0.0058 | 0.778 ± 0.008 | 1.05 ± 0.008 | 0.6369 ± 0.05 | 1.011 ± 0.011 | 0.951 ± 0.08 | 0.9075 ± 0.004 | | 0.3254 ± 0.002 | |
| ***Sediment*** | | | | | | | | | | | | | | | |
| **U (ppm)** | 8.1 ± 0.46 | 8.1 ± 0.46 | 10.1 ± 0.5 | 5 ± 0.3 | 4.99 ± 0.24 | 4.99 ± 0.24 | 3.85 ± 0.3 | 3.85 ± 0.3 | 5.1 ± 0.47 | 7.7 ± 0.4 | 10.2 ± 0.6 | 3.06 ± 0.14 | 10.2 ± 0.6 | | 7 ± 0.3 | |
| **Th (ppm)** | 13.2 ± 1.5 | 13.2 ± 1.5 | 7.6 ± 0.2 | 6.2 ± 0.1 | 7.54 ± 0.81 | 7.54 ± 0.81 | 5.96 ± 0.91 | 5.96 ± 0.91 | 12.7 ± 1.6 | 9.8 ± 0.3 | 3.9 ± 0.2 | 5.16 ± 0.46 | 3.9 ± 0.2 | | 9.3 ± 1 | |
| **K (%)** | 0.72 ± 0.12 | 0.72 ± 0.12 | 1.45 ± 0.1 | 0.41 ± 0.05 | 0.29 ± 0.07 | 0.29 ± 0.07 | 1.42 ± 0.09 | 1.42 ± 0.09 | 0.32 ± 0.1 | 1.6 ± 0.2 | 1.2 ± 0.05 | 1.83 ± 0.6 | 1.2 ± 0.05 | | 0.29 ± 0.1 | |
| ***Coupled US-ESR*** | | | | | | | | | | | | | | | |
| **Uptake parameter Enamel** | -0.39 ± 0.16 | -0.4 ± 0.12 | 0.4 ± 0.26 | 2.23 ± 0.28 | 3.46 ± 0.44 | 0.23 ± 0.40 | 0.15 ± 0.19 | 0.16 ± 0.20 | -0.82± 0.06 | 3.69 ± 1.62 | 7.4 ± 0.66 | 6.95 ± 0.50 | -0.06 ± 0.1 | | 2.57± 0.29 | |
| **Uptake parameter Dentine** | -0.64 ± 0.10 | -0.27 ± 0.16 | -0.6 ± 0.09 | 0.38 ± 0.11 | 0.74 ± 0.16 | 0.61 ± 0.53 | -0.83 ± 0.38 | -0.55 ± 0.11 | -0.98 ± 0.05 | 2.08 ± 1.33 | -0.70 ± 0.01 | -0.43 ± 0.02 | -0.04 ± 0.1 | | 1.73 ± 0.23 | |
| **Total Dose (μGy/a)^b^** | 5442 ± 1044 | 2792 ± 682 | 2228 ± 335 | 1858 ± 194 | 1523 ± 150 | 2489 ± 810 | 2510 ± 319 | 1295 ± 165 | 4529 ± 828 | 1281 ± 523 | 1767 ± 164 | 1607 ± 71 | 2228 ± 307 | | 1286 ± 95 | |
| **Age (ka)^d,e^** | **351 ± 68** | **684 ± 165** | **324 ± 44** | **897 ± 78** | **757 ± 70** | **620 ± 182** | **357 ± 42** | **271 ± 31** | **314 ± 54** | **786 ± 252** | **639 ± 47** | **714 ± 43** | **962 ± 113** | | **181 ± 12** | |

^a^ Dose equivalent D_e_ obtained using McDose 2.0, with SSE.

^b^ Uranium concentration values were obtained by LA-MC-ICPMS on the enamel measured by ESR and on dentine directly in contact with the fragment.

^c^ Baxian results are for fossil tooth attributed to *Pongo sp*., while all other results presented in this table were obtained directly on *Gigantopithecus blacki* fossil teeth.

^d^ The age was calculated using the U and Th decay from ^205^, the enamel and dentine density of 2.95 and 2.85 respectively from ^206^, alpha efficiency factor of 0.13 +/- 0.02, U8-U4 alpha dose (260 mGy/ka) from ^207^, U4-Th0 alpha dose (295 mGy/ka) from^208^. Water content was estimated to be 3% +/-1 in the enamel, 5 %+/-3 in the dentine and 15 % +/-10 in the sediment, using heat treatment at 60ºC for 24h on a *Pongo* tooth and sediment powder respectively.

^e^ Uncertainties have been presented at 1 σ

**Table S15:** Age results for all coupled US-ESR and US dating analyses.

|  | **Site** | **Specimen no.** | **Identification** | **US-ESR ages** | **err (±) 1s** | **US ages** | **err (±) 2s^b^** |
| --- | --- | --- | --- | --- | --- | --- | --- |
| ***Gigantopithecus blacki*** | **Baikong** | *CLBBD201011-1064* | P3 |  |  | **225.4** | **7.5** |
|  |  | *CLBBD201011-MT35* | P3 |  |  | **186.4** | **3.0** |
|  |  | *CLBBD201011-1179* | P3 |  |  | **294.5** | **9.4** |
|  |  | *CLBBD201011-858* | Mx | **351** | **68** | **195.7** | **6.6** |
|  |  | *CLBBD201011-859* | Mx | **684** | **165** | **182.1** | **2.9** |
|  |  | *CLBBD201011-1127* | Mx |  |  | **165.5** | **4.1** |
|  | **Bapeng** | *BAPENG-18* | M1/2 | **324** | **44** | **180.1** | **8.2** |
|  |  | *BAPENG-6* | M3 |  |  | **308.7** | **14.1** |
|  | **Queque** | *CSQZ0811-64* | M1/2 | **897** | **78** | **166.7** | **1.7** |
|  | **Sanhe** | *GCSD0410-252* | M1/2 | **757** | **70** | **158.8** | **5.3** |
|  |  | *GCSD0410-256* | M1/2 | **897** | **78** | **144.4** | **2.7** |
|  |  | *GCSD0410-225* | M1/2 | **620** | **182** | **141.1** | **3.4** |
|  | **Shuangtan** | *CMLST0911-50* | Mx | **271** | **31** | **228.2** | **13.4** |
|  |  | *CMLST0911-109* | P4 | **357** | **42** | **197.8** | **15.2** |
|  | **Hejiang** | *CLMH0904-93* | M1 |  |  | **297.6** | **6.4** |
|  |  | *CLMH0904-89* | P3 | **314** | **54** | **98.1** | **3.0** |
|  | **Yanliang** | *CFLGYL201111-806* | Mx | **786** | **252** | **169.8** | **15.6** |
|  |  | *FLGY201104-Gy-1-3* | M1 |  |  | **131.6** | **8.1** |
|  |  | *FLGY201104-GY-1-1* | M3 |  |  | **157.9** | **8.4** |
|  | **Zhanwang** | *B1MF^a^* | Mx | **962** | **113** | **-** | **-** |
|  |  | *O1Z1* | Mx | **714** | **43** | **270.8** | **15.3** |
|  |  | *O1Z2* | Mx | **639** | **47** | **230.0** | **27.8** |
| ***Pongo weidenreichi*** | **Daxin** | *DLZNH201211-1-13* | M1/2 |  |  | **181.7** | **5.1** |
|  |  | *DLZNH201211-1-28* | M1/2 |  |  | **429.0** | **26.2** |
|  |  | *DLZNH201211-740* | M1 |  |  | **527.8** | **34.2** |
|  |  | *DLZNH201211-15* | M1 |  |  | **379.5** | **18.7** |
|  |  | *DLZNH201211-6* | M1 |  |  | **140.4** | **3.6** |
|  |  | *DLZNH201211-797* | M2 |  |  | **168.0** | **3.0** |
|  | **Baxian** | *RTK201306-77* | M1/2 | **181** | **12** | **156.7** | **8.2** |
|  |  | *RTK201306-103* | M1/2 |  |  | **133.9** | **7.7** |
|  |  | *RTK201306_45* | M1 |  |  | **42.6** | **0.8** |
| ***Fauna*** | **Shuangtan** | *Cervid 278* | Mx |  |  | **248** | **13.5** |
|  |  | *Cervid 279* | Mx |  |  | **228.9** | **21.6** |
|  |  | *Cervid 286* | Mx |  |  | **228.6** | **13.7** |
|  |  | *Cervid 287* | Mx |  |  | ***-*** | ***-*** |
|  |  | *Suid 258* | Mx |  |  | **290.6** | **19.1** |
|  |  | *Suid 279* | Mx |  |  | **214.9** | **6.3** |
|  |  | *Suid 281* | Mx |  |  | **389.1** | **37.1** |
|  |  | *Suid 287* | Mx |  |  | **164.9** | **7.0** |

^a^ U-series results for B1MF and Cervid 287 could not be calculated because of program numerical violation, likely due to leaching of uranium from the dental tissues (see discussion)

b US age estimates are presented in 2 σ (Table S13) but when used in conjunction with the ESR ages estimates (1 σ) they are also reduced to 1 σ, thus, the resulting coupled US-ESR ages are presented in 1 σ

**SI section 7: ESR dating of quartz**

1. **Material & methods**
   1. *Samples*

A total of seven samples of purified quartz were analysed for ESR dating purposes (Table S16). For a couple of the samples (CBAIK10, CZW2), two grain-size fractions were measured.

**Table S16** **-** List of the quartz samples analysed in the present study and basic information about the experimental conditions employed for the ESR dose evaluation. Key: n/a = not applicable.

|  | **Sample ID** | **Cave** | **Grain size (µm)** | **Aliquot mass (mg)** | **Number of dose steps** | **D_max_ (Gy)** | **Acquisition conditions** |
| --- | --- | --- | --- | --- | --- | --- | --- |
| #1 | CQQ1 | Queque | 212-350 | 50.1 ± 0.6 | 6 | 376 | Separate spectra |
| #2 | CDAX9 | Daxin | 212-350 | 149.9 ± 0.3 | 13 | 14105 | Separate spectra |
| #3 | CDAX10 | Daxin | 90-212 | 89.4 ± 0.3 | 13 | 14162 | n/a |
| #4 | CBAIK10 | Baikong | 90-212 | 47.6 ± 1.5 | 9 | 1416 | Separate spectra |
| #5 |  |  | 212-350 | 89.8 ± 0.4 | 13 | 14105 | Single spectrum |
| #6 | CZW2 | Zhanwang | 90-212 | 39.9 ± 0.2 | 6 | 378 | Separate spectra |
| #7 |  |  | 212-350 | 60.1 ± 0.3 | 9 | 1410 | Single spectrum |

- 1. *X-ray Diffraction analyses*

Characterisation analyses using X-ray diffraction spectroscopy were performed at CENIEH on several samples in order to evaluate their mineralogical composition (Table S17).

**Table S17 -** Results of the bulk XRD analyses reformed on several samples. Composition values in %.

| Sample | Quartz | Calcite | Fluorite | Iron oxide |
| --- | --- | --- | --- | --- |
| CDAX9 (212-350 µm) | 96 | 3 | _ | 1 |
| CDAX10 (90-212 µm) | 33 | 26 | 41 | _ |
| CQQ1 (212-350 µm) | 97 | _ | 3 | _ |
| CZW2 (212-350 µm) | 100 | _ | _ | _ |

- 1. *ESR dosimetry*

Quartz grains were dated via the Multiple Aliquots Additive Dose (MAAD) method and following the Multiple Centre (MC) approach initially defined by^39^. In each sample, the ESR signals of both the Aluminium (Al) and Titanium (Ti) centres were either acquired in separate spectra using specifically optimized parameters (standard CENIEH procedure, e.g.^40^, or in a single spectrum (e.g., ^41^) (see details in Table S16).

Gamma irradiations were performed at CENIEH using a Gammacell-1000. The following irradiation doses were applied to samples CQQ1 (212-350 μm), CDAX9 (212-350 μm), CBAIK10 (212-350 μm) and CZW2 (212-350 μm) (dose rate: 6.018 ± 0.138 Gy/min): 0.0, 47.0, 94.1, 188.2, 282.2, 376.3, 564.4, 940.3, 1410.4, 2821.0, 5642.0, 8462.9 and 14104.9 Gy. Samples CDAX10 (90-180 μm), CBAIK10 (90-180 μm), CZW2 (90-212 μm) were irradiated to the following doses (dose rate: 6.042± 0.139 Gy/min): 0.0, 47.2, 94.5, 188.9, 283.4, 377.9, 566.7, 944.1, 1416.2, 2832.4, 5664.8, 8497.2 and 14162.1 Gy. For each sample, the number of irradiation dose steps was selected according to the amount of quartz available (see Table S16). One aliquot of each sample optically bleached by artificial solar light after exposure in a SOL2 (Dr Hönle) simulator for about 1500 h in order to evaluate the non-bleachable component of the ESR signals associated to the Aluminium (Al) centre.

ESR measurements were carried out at CENIEH (Burgos, Spain), with an EMXmicro 6/1Bruker X-band ESR spectrometer coupled to a standard rectangular ER 4102ST cavity. To ensure constant experimental conditions over time, the temperature of the water circulating in the magnet is controlled and stabilized at 18 ºC by a water-cooled Thermo Scientific NESLAB ThermoFlex 3500 chiller, and the temperature of the room is kept constant at 20 ºC by an air conditioning unit. ESR measurements were performed at low temperature (~90 K) using an ER4141VT Digital Temperature control system based on liquid nitrogen cooling. Further details about the setup and about its stability over time can be found in ^209,2010^.

When measured in separate spectra, ESR signals of both the Al and Ti centers were obtained from the following acquisition parameters:

- Al signal: 10 mW microwave power, 1024 points resolution, 20 mT sweep width, 342 mT centre field, 100 kHz modulation frequency, 0.1 mT modulation amplitude, 40 ms conversion time, 10 ms time constant and 1 scan.
- Ti signals: 5 mW microwave power, 1024 points resolution, 20 mT sweep width, 350 mT centre field, 100 kHz modulation frequency, 0.1 mT modulation amplitude, 60 ms conversion time, 10 ms time constant and 1 scan.

In comparison, when both Al and Ti signals were acquired together, the following conditions were employed: 5 mW microwave power, 2048 points resolution, 40 mT sweep width, 342 mT centre field, 100 kHz modulation frequency, 0.1 mT modulation amplitude, 40 ms conversion time, 10 ms time constant and 1 scan.

For each sample, all the aliquots (one natural, one optically bleached and several gamma-irradiated aliquots) were measured 3 times after a ~120° rotation in the cavity in order to consider angular dependence of the Al and Ti signals due to sample heterogeneity. Then, depending on the sample and signal considered, measurements were repeated two to four times over distinct days in order to evaluate the repeatability of the D_e_ values.

The ESR intensity of the Al signal was extracted from peak-to-peak amplitude measurements between the top of the first peak (g=2.0185) and the bottom of the 16th peak (g = 1.9928)^211^ (Fig. S8C). The ESR intensity of the Ti centres was measured following options D and E *sensu* ^212^ (Fig. S8C): option D (Ti-Li and Ti-H absorption lines around g = 1.913) was employed for dose evaluation, while the intensity derived from option E (Ti-Li absorption line around g = 1.979) was used for comparison with the Al signal.

For each aliquot, ESR intensities of Al and Ti centres were corrected by the corresponding receiver grain value, number of scans, aliquot mass and a temperature correction factor^209^. The fitting procedures were carried out with the Microcal OriginPro 8.5 software using a Levenberg-Marquardt algorithm by chi-square minimization. Final Dose Response Curves (DRCS) were obtained by using the mean ESR intensities and associated error (1 standard deviation) derived from each repeated measurement. Depending on the samples and ESR signals considered, fitting was performed using either an exponential+linear function (EXP+LIN) (see equation in ^213^) or a Single Saturating Exponential (SSE) function (see equation in ^213^), and data were weighted by the inverse of the squared ESR intensity (1/I^2^) ^213^, the inverse of the squared experimental error (1/s2) and with equal weights (EW).

- 1. *Dose rate evaluation and age calculation*

Dose rate and ESR age calculations were performed with DRAC (v1.2.)^215^, using the dose rate conversion factors from ^34^, alpha attenuation from ^216^, beta attenuations from ^217^ and etching depth attenuations from ^181^. Etching depth values of 10 ± 5 µm were considered following ^217^. Radioelement concentrations were obtained from both in-situ measurements (CDAX8-9 and CZW2) and laboratory measurements (CQQ1 and CBAIK10) using a combination of alpha and beta counting (see Supp 4). The internal dose rate was assumed to be 30 ± 10 uGy/a, based on the work from ^218^ and using an alpha efficiency of 0.07 ± 0.01^41^. The same long-term water content and cosmic dose rates values were used for ESR and Luminescence calculations ESR ages are given at 1σ.

1. **Results**
   1. *ESR data*
      1. Baikong Cave

CBAIK10 (90-212 µm)

Al and Ti signals were acquired separately (Fig. S8A and B). Given the limited amount of material available, we used 9 aliquots of about 47 mg each, with irradiations up to 1416 Gy (Table S16). Because of the limited D_max_, DRC fitting was performed using a SSE function (Fig. S9C). Goodness-of-fit is good, with adjusted values of >0.99 (Table S18). Data weighting has only a limited impact on D_e_ estimates, which vary by 7%, depending on the option considered. ESR age estimates were calculated using the D_e_ result from 1/I^2^.

The sample shows a Ti signal with an exceptionally high ESR intensity: the Ti-Li absorption line at g=1.979 (option E sensu ^211^) represents about 45% of that of the Al signal. However, the Ti signal (Ti-Li and Ti-H absorption lines around g=1.913; option D sensu ^211^) does not show any significant increase of intensity with the dose (Fig. S10B). Consequently, no meaningful dose estimate can be derived from these data.

CBAIK10 (212-350 µm)

The coarser grain size fraction (212-350 µm) of sample CBAIK10 yielded a larger amount of material, leading to the establishment of 13 data point DRC with aliquots of about 89 mg (Table S16).The ESR data associated to the Al signal are of good quality (Table S18): the repeated measurements show a limited D_e_ variability of ~13%, while the various EXP+LIN fittings performed with different data weighting options return D_e_ estimates that are all in 1-σ agreement (Fig. S9D). ESR age estimates were calculated using the D_e_ result from 1/I^2^.

Repeated measurements of the Ti signal (option D) return highly variable D_e_ estimates, whereas the goodness-of-fit achieved for the final DRC is overall regular, whatever the data weighting option considered (0.95 < adjusted r2 < 0.97). The latter has a very limited impact on the dose results (Fig. S10C). SSE fitting return massive D_e_ estimates (>6000 Gy) (Table S19), which are significantly higher than those obtained for the Al signal. Ulike the finer grain size fraction, the ESR intensity of the coarser grains does show an increase with the dose. However, we cannot exclude that this apparent behavior may simply result from the higher irradiation doses given to the coarse grains (up to 15 kGy). Indeed, the first irradiation points <2 kGy are very scattered and showed limited increase, i.e. similar to the behavior of the finer grains irradiated up to 1.5 kGy.

- - 1. Queque Cave

CQQ1 (212-350 µm)

Al and Ti signals of sample CQQ1 (212-350 µm) were acquired in separate spectra (see examples in Fig. S7A and B). Given the very limited amount of material available, ESR measurements were performed with small aliquots of ~50 mg and a DRC was built with only 6 points (Table S16). While XRD results showed the presence of a small proportion of fluorite in the sample (Table S17), no major interfering signal was observed in the ESR spectra.

The quality of the ESR data set obtained for both the Al and Ti signals is well below average. The Al signal produced somewhat scattered experimental data points, with poor goodness-of-fit, as illustrated by the very low adjusted r^2^ values (<0.92; Table S18). Repeated measurements show a significant variability of the resulting D_e_ estimates (71 %). Given the limited D_max_ value, fitting was performed with the SSE function and different data weighting options were tested: they all return very close D_e_ estimates in the range of 200-215 Gy, showing that data weighting has negligible impact on the dose values (Fig. S9A). Consequently, the D_e_ derived from SSE (1/I^2^) were used for age calculations. In summary, given the poor goodness-of-fit and D_e_ repeatability, as well as the limited number of dose steps, the reliability of the dose evaluation derived from this signal should be taken with caution.

In comparison, the Ti signal (option D) produces a surprisingly flat DRC, with very scattered experimental points, precluding the use of any fitting function (Fig. S7A). No meaningful dose estimate can be derived from this signal. The shape of the DRC indicates that either (i) the Ti signal measured in this sample shows poor radiation sensitivity, (ii) or it has reached apparent saturation. In this case, this would mean that the sample has absorbed a massive radiation dose of several thousands of Gy (Ti option D usually shows apparent saturation in the range of about 8,000 to 12,000 Gy; see ^212^. However, this hypothesis would be in clear contradiction with the dose estimate derived from the Al centre.

2.1.3 Zhanwang Cave

CZW2 (90-212 µm)

Given the limited amount of material available, aliquots of about 40 mg were made and with only 6 irradiation dose steps (Table S16). With a D_max_ of 378 Gy, DRC fitting of the Al data points was performed with the SSE function, using three different fitting options (Fig. S9E): the resulting D_e_ estimates significantly vary by about 35%, while goodness-of-fit is poor (adjusted r2 < 0.61; Table S18) and D_e_ error are huge (>100 %). Consequently, the reliability of these fitting results can be reasonably questioned and the resulting dose estimates are meaningless. No ESR age estimated was therefore calculated.

In comparison, the ESR measurement of the Ti centre show that intensities of Ti Option D does not significantly increase with the dose, precluding thus any dose response curve fitting (Table S18 & Fig. S10D).

CZW2 (212-350 µm)

The higher amount of material available for sample CZW2 (212-350 µm) compared to the lower grain size fraction, enabled to work with bigger aliquots of ~60 mg, and build a DRC with 9 points (Table S18). While Al and Ti signals were acquired in a single spectrum (Fig. S8F), no major interfering ESR signal is observed, which is consistent with DRX analyses (Table S17). The limited D_max_ (1410 Gy) precludes the use of the EXP+LIN function classically employed for the Al signal^209^, and a SSE function was used instead. The three different fitting options yield very close D_e_ estimates (within ± 6%) of around 710 Gy (Table S18). Goodness-of-fit is regular (0.95 < adjusted r^2^ < 0.98), but nevertheless much better than that achieved for CZW2 (90-212 µm). Consequently, dose estimates derived for this sample are believed to be more reliable. ESR age estimates were calculated using the D_e_ result from 1/I^2^.

Despite the limited size of the aliquots, the ESR signal of the Ti centre is relatively strong: the Ti-Li absorption line a g=1979 (=option E sensu ^212^) represents about 34% of that of the Al signal in the natural aliquot (Fig. S8F). ESR intensities show an overall increase with the dose, while very scattered. Fitting performed with the SSE function and using three different data weighting option return highly variable D_e_ estimates (Table S19), ranging from 1559 to 2873 Gy (Fig. S10E). The goodness-of-fit is poor for 2 options (adjusted r^2^ <0.90), whereas it is regular for data weighting by 1/s^2^ (adjusted r^2^ = 0.95). Furthermore, the magnitude of the D_e_ error is massive (up to 94%), which is the resulting combination of insufficient D_max_ and scattered data points ^213^. Since the quality of the ESR data set is well below usual standards, the reliability of the fitting results and dose estimates should be considered with extreme caution. ESR age estimates were calculated using the D_e_ result from 1/I^2^.

2.1.4. Daxin Cave (CDAX)

CDAX9 (212-350 µm)

ESR measurements of sample CDAX9 (212-350 µm) show a massive interfering signal, with a main peak positioned just in the middle of the Al signal, and has an intensity that is >10 times higher (Fig. S7C and D). This interfering signal is attributed to calcite, which is confirmed by the bulk XRD results (Table S16). Since this signal does not interfere with the amplitude measurement of the Al signal between the 1^st^ and 16^th^ peak, ESR measurements were performed anyway. In contrast, no Ti signal could be observed or measured for this sample (Fig. S7C and D).

The relatively large amount of material available for this sample enabled to build a proper DRC with 13 data points and have sufficient amount per aliquot (~150 mg). Repeated measurements return a D_e_ variability of about 21 %, while the bleaching coefficient is about half of that observed for sample CQQ1 (Table S18). DRC fitting was performed with the EXP+LIN function given the high D_max_ value (14162 Gy). Various data weighting options were tested and yield D_e_ estimates ranging from ~655 Gy (1/s^2^) to ~1335 Gy (EW) (Fig. 9B). This significant variability (a factor of 2), combined the presence of the massive interfering signal, indicate that the robustness and reliability of the ESR data is not optimum. D_e_ estimates should be considered as purely indicative. ESR age estimates were calculated using the D_e_ result from 1/I^2^.

**Figure S7**: ESR spectra obtained from the measurement of various quartz samples: CQQ1 (212-350 µm), Al (A) and Ti signals (B) of the natural aliquot; CDAX9 (212-350 µm), Al signal (C) and zoom on a smaller intensity window to visualize the Al signal of the natural aliquot; CDAX10 (90-212 µm), wide scan covering the windows of the Al and Ti signals of the aliquot irradiated to 48 Gy. The Al signal was acquired between 3250 and 34450 G, and the Ti signals between 3410 and 3580 G.

**
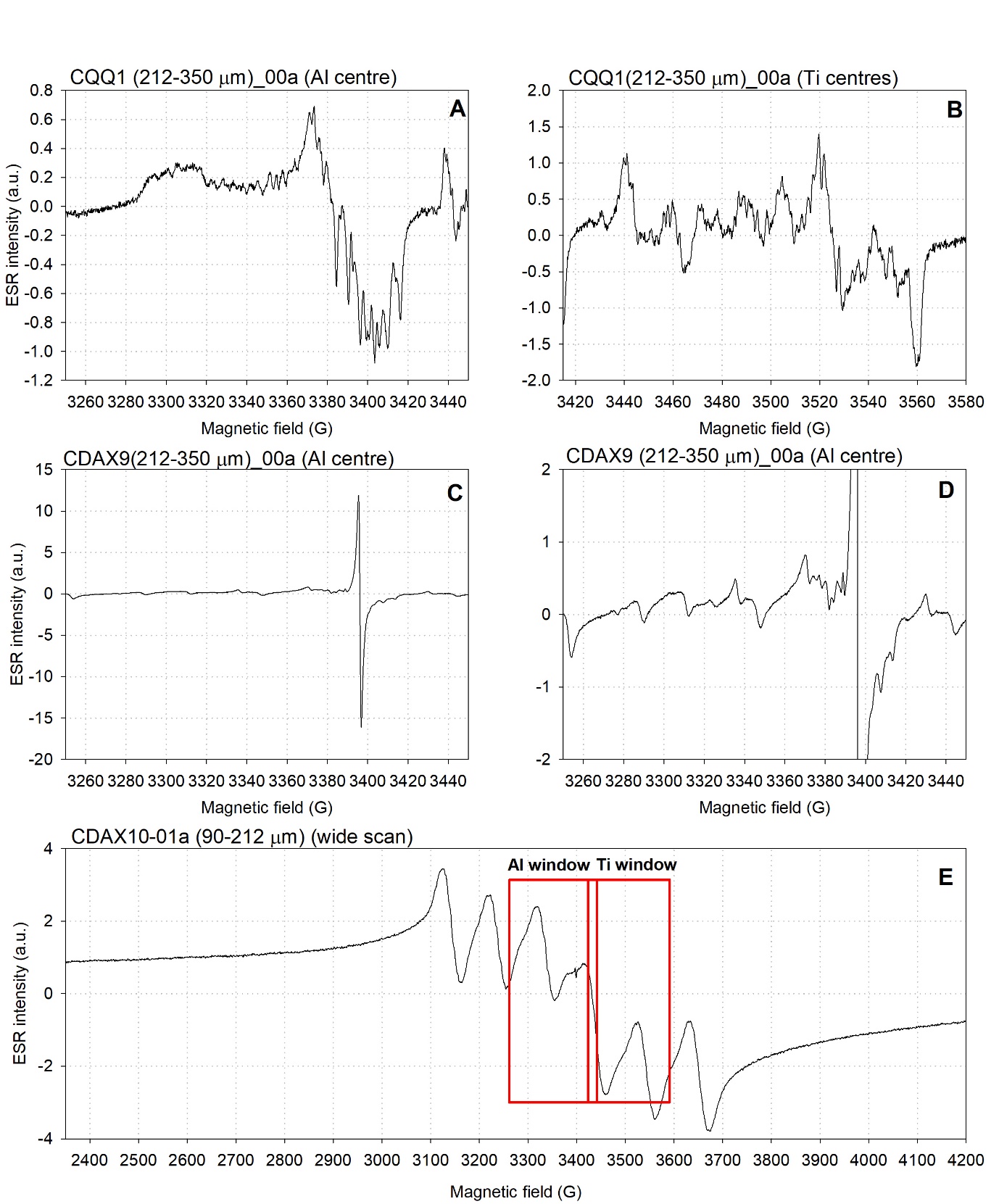
**

**Figure S8**: ESR spectra obtained from the measurement of various quartz samples: CBAIK10 (90-212 µm), Al (A) and Ti signals (B) of the natural aliquot; CBAIK10 (212-350 µm) Al and Ti signals of the natural aliquot acquired in a single spectrum (C); CZW2 (90-212 µm), Al (D) and Ti signals (E) of the natural aliquot; CZW2 (212-350 µm), Al and Ti signals of the natural aliquot acquired in a single spectrum (F). The Al signal was acquired between 3250 and 34450 G, and the Ti signals between 3410 and 3580-3600 G.


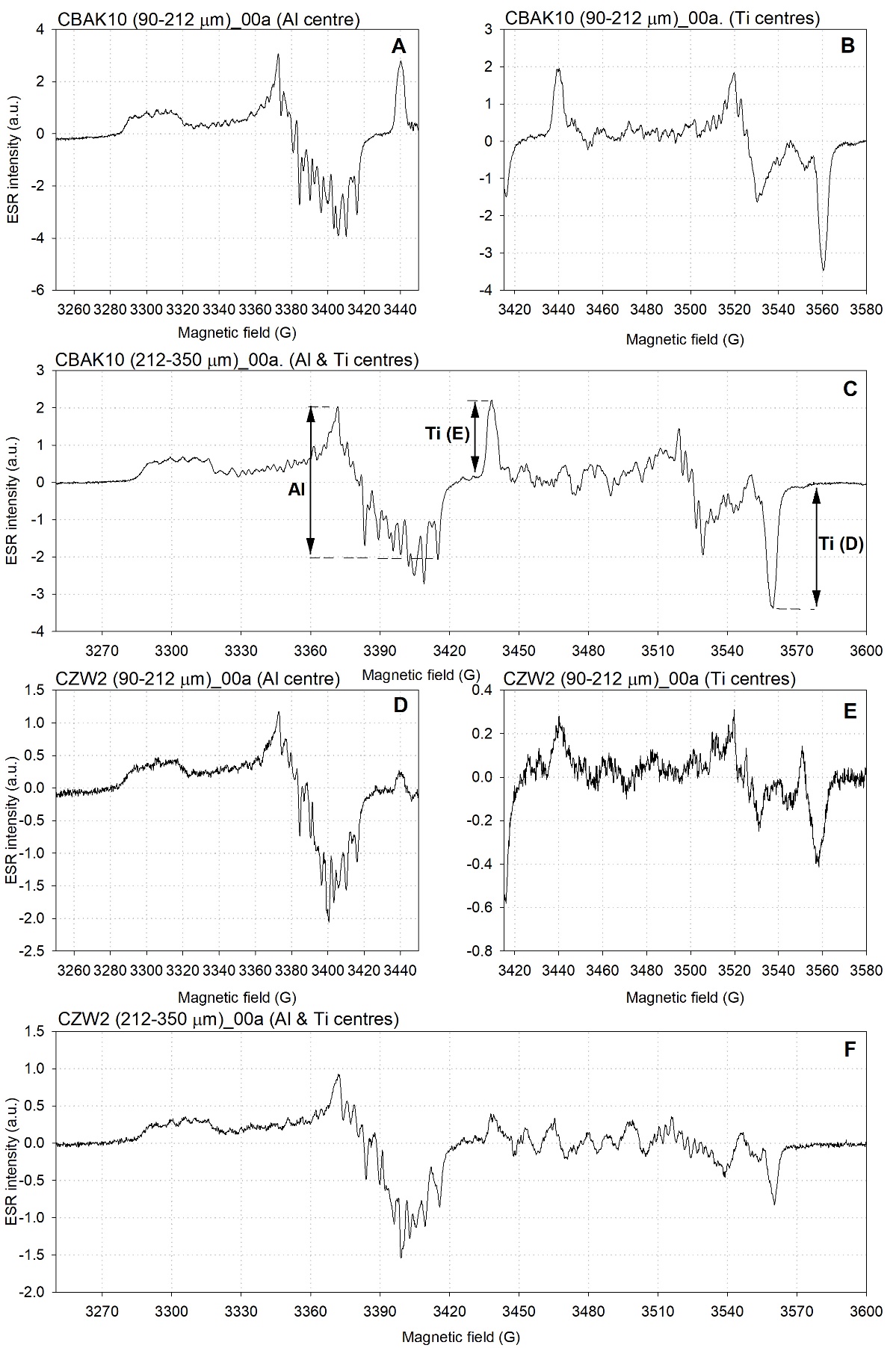


CDAX10 (90-212 µm)

Like CDAX9, CDAX10 (90-212 µm) shows a massive interfering signal as shown by the wide scan displayed in Fig. S7E. Both the Al and Ti signals are simply not visible on the ESR spectra acquired for this sample. This is confirmed by the bulk XRD analyses, which indicate a large proportion of fluorite (41 %) and calcite (26 %) in this sample. No dose estimate can be obtained for this sample.

1. **Discussion**
   1. *D_e_ estimates*

Among these 7 samples analysed in the present study, one sample did not produce any measurable Al and Ti signals (CDAX10_90-212 µm) due to interfering signals attributed to fluorite and calcite components (Table S17). These contaminations most likely result from the sample preparation procedure. Fluorite might come from an incomplete cleaning after the HF etching, while the calcite components might result from incomplete dissolution of the carbonates. Another sample shows a significant calcite contamination (CDAX9_212-350 µm), making it impossible to obtain any measurable Ti signal. Interestingly, these two samples come from the same locality, suggesting that these contamination issues may be related with the specific nature and composition of the raw sediment at the site. Additionally, the D_e_ value obtained from the Al signal measured in CDAX9_212-350 µm should be regarded as a maximum possible estimate of the true burial dose, in the absence of any comparative result from the Ti centres.

While 5/7 samples show measurable Ti centre, dose estimates could be obtained for only two of them. Three samples show an apparently flat DRC (Fig. S10), indicating that the Ti (option D) signal does not significantly increase with the dose. It is possible that this behavior may indicate that the Ti signal has reached saturation, indirectly suggesting a large environmental radiation dose. It is worth mentioning that the intensity of the Ti (option E sensu ^211^) = Ti-Li absorption line around g=1.979) signal is exceptionally high in the two natural samples CBAIK10. The intensity represents about 50% of the intensity of the Al signal on average. Samples CQQ1 and CZW2 (212-350 µm) also show high ratio values of 28 and 34%. For example, CBAIK10_212-350 µm returns a massive D_e_ value of > 6000 Gy (Table S19). However, such hypothesis is weakened by the corresponding D_e_ estimate derived from the Al signal, which does not exceed 1500 Gy for that sample. Following the basic principles of the MC approach, the D_e_ value obtained from the Al signal should be interpreted as a maximum possible estimate, given its slower bleaching kinetics compared to the Ti signals. Therefore, we presently do not have any explanation for the discrepancy observed between Al and Ti dose estimates of the two samples CBAIK10.

**Table S18**: ESR data derived from the measurement of the Al centre. Bleaching coefficient is expressed as the relative difference between the ESR intensities of the natural and bleached aliquots. Repeatability of the ESR intensities is assessed through the variability (1 relative standard deviation) of the mean ESR intensities obtained after each day of measurements. Similarly, the repeatability of the D_e_ values corresponds to the variability (1 relative standard deviation) of the D_e_ values calculated for each day of measurement.

| Sample | Repeated measurements | Bleaching  Coefficient  (%) | Repeatability of the ESR intensities (%) | Repeatability of the D_e_ estimates  (%) | Fitting function | Data weighting by 1/I^2^ | | Data weighting by 1/s^2^ | | Equal Weights | |
| --- | --- | --- | --- | --- | --- | --- | --- | --- | --- | --- | --- |
|  |  |  |  |  |  | Adjusted r^2^ | D_e_ value (Gy) | Adjusted r^2^ | D_e_ value (Gy) | Adjusted r^2^ | D_e_ value (Gy) |
| CQQ1  (212-350 µm) | 3 | 60.7 ± 4.6 | 0.9 | 71.7 | SSE | 0.918 | 215± 98 | 0.899 | 212± 116 | 0.907 | 203± 97 |
| CDAX9  (212-350 µm) | 3 | 32.5 ± 0.8 | 1.4 | 21.2 | EXP+LIN | 0.963 | 930± 260 | 0.982 | 655± 129 | 0.985 | 1334 ± 294 |
| CDAX10  (90-212 µm) | No measurable signal | | | | | | | | | | |
| CBAIK10  (90-212 µm) | 4 | 76.9 ± 5.4 | 0.6 | 18.8 | SSE | 0.992 | 927 ± 127 | 0.994 | 1015± 134 | 0.993 | 887 ± 123 |
| CBAIK10  (212-350 µm) | 3 | 78.5 ± 5.7 | 2.2 | 12.8 | EXP+LIN | 0.996 | 1120 ± 150 | 0.995 | 1027 ± 105 | 0.999 | 1320 ± 129 |
| CZW2  (90-212 µm) | 3 | 61.7 ± 4.4 | 0.4 | 70.0 | SSE | 0.582 | 1411± 7775 | 0.539 | 1848± 16139 | 0.606 | 863 ± 2498 |
| CZW2  (212-350 µm) | 3 | 59.8 ± 3.1 | 1.2 | 68.1 | SSE | 0.951 | 669± 193 | 0.984 | 704± 244 | 0.968 | 749 ± 208 |

**Figure S9**: ESR dose response curves obtained from the measurement of the Al centre. Experimental data points represent mean ESR intensities and associated 1 standard deviation (vertical error bars), derived from repeated measurements (n=3-4 depending on the samples; see Table S18).


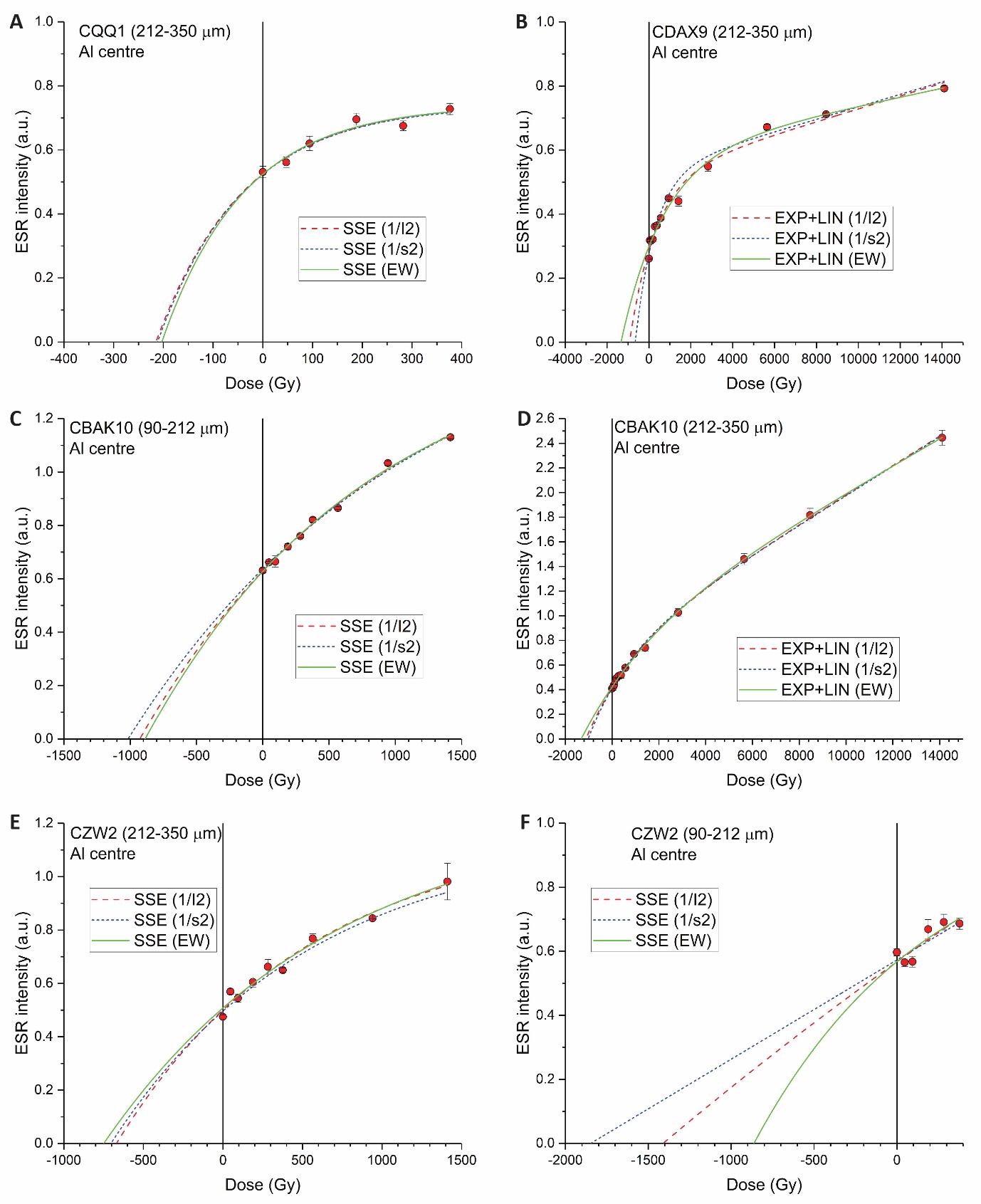


**Fig. S10**: ESR dose response curves obtained from the measurement of the Ti centre. Experimental data points represent mean ESR intensities and associated 1 standard deviation (vertical error bars), derived from the repeated measurements (n=2-3 depending on the samples; see Table S19).
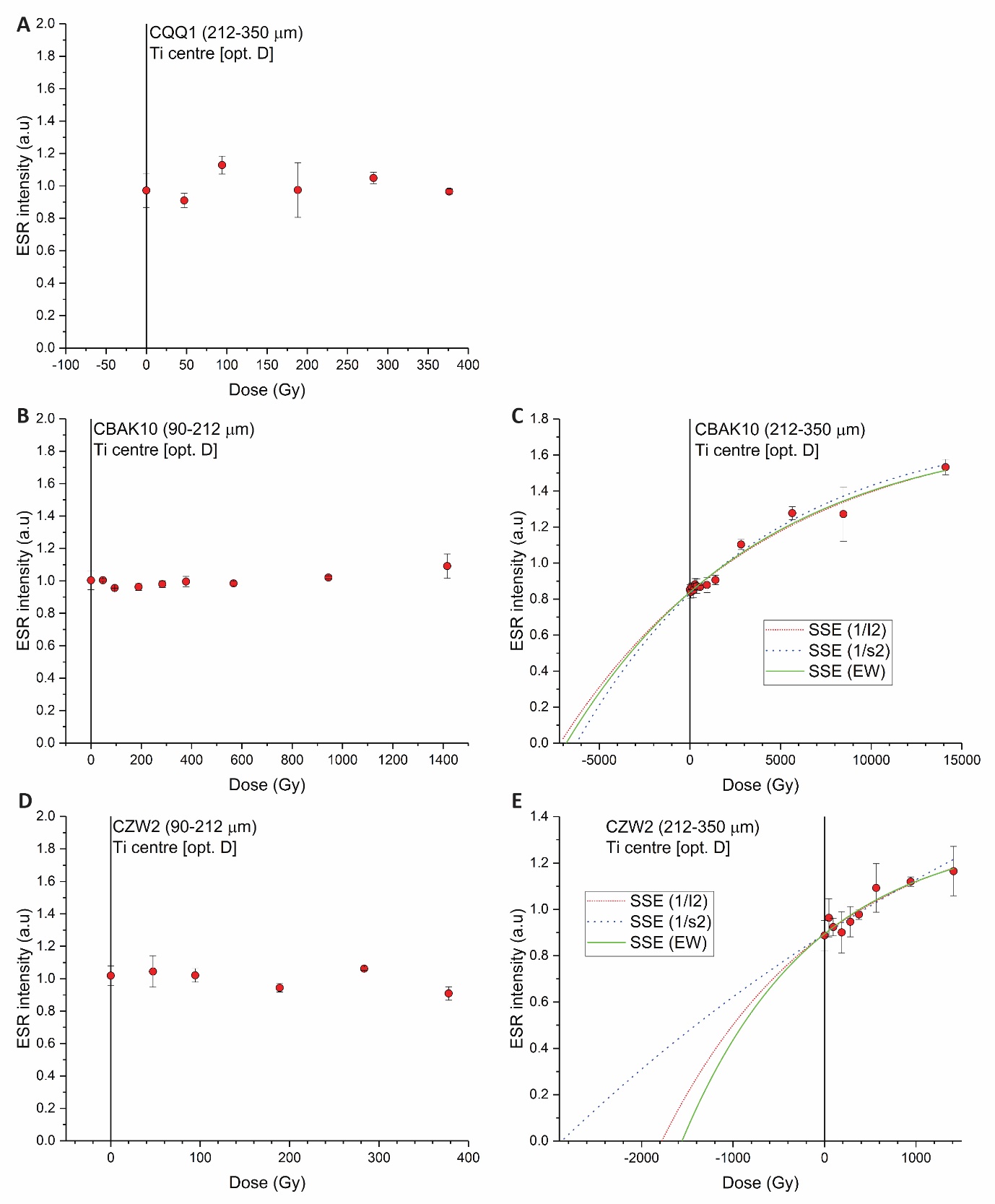


| **Sample** | **Repeated measurements** | **Repeatability of the ESR intensities (%)** | **Repeatability of the D_e_ estimates**  **(%)** | **Fitting function** | **(1/I^2^)** | | **(1/s^2^)** | | **(EW)** | |
| --- | --- | --- | --- | --- | --- | --- | --- | --- | --- | --- |
|  |  |  |  |  | **Adjusted r^2^** | **D_e_ value (Gy)** | **Adjusted r^2^** | **D_e_ value (Gy)** | **Adjusted r^2^** | **D_e_ value (Gy)** |
| CQQ1  (212-350 µm) | 2 | 3.5 | n.a. | n.a. | n.a. | n.a. | n.a. | n.a. | n.a. | n.a. |
| CDAX9  (212-350 µm) | No visible signal | | | | | | | | | |
| CDAX10  (90-212 µm) | No visible signal | | | | | | | | | |
| CBAIK10  (90-212 µm) | 2 | 0.7 | n.a. | n.a. | n.a. | n.a. | n.a. | n.a. | n.a. | n.a. |
| CBAIK10  (212-350 µm) | 3 | 2.6 | 55.0 | SSE | 0.955 | 7166 ± 1742 | 0.957 | 6262 ± 1260 | 0.967 | 6824 ± 1493 |
| CZW2  (90-212 µm) | 2 | 1.8 | n.a. | n.a. | n.a. | n.a. | n.a. | n.a. | n.a. | n.a. |
| CZW2  (212-350 µm) | 3 | 1.3 | 41.5 | SSE | 0.825 | 1779 ± 1464 | 0.953 | 2873 ± 2710 | 0.847 | 1559 ± 1106 |

**Table S19**: ESR data derived from the measurement of the Ti centre (option D). Repeatability of the ESR intensities is assessed through the variability (1 relative standard deviation) of the mean ESR intensities obtained after each day of measurements. Similarly, the repeatability of the D_e_ values corresponds to the variability (1 relative standard deviation) of the D_e_ values calculated for each day of measurement. Key: n.a. = not applicable.

**Table S20**: ESR age estimates and dose rate components. Errors are 1 sigma. Note that the D_e_ errors are a combination of the fitting errors (from Tables S1 and S2) and of the gamma source dose rate (2.3%). Dose rates were calculated with DRAC (v.1.2) ^215^. Key: n.c. = not calculated.

| Sample | CDAX9 | CDAX10 | CQQ1 | CBAIK10 | CBAIK10 | CZW2 | CZW2 |
| --- | --- | --- | --- | --- | --- | --- | --- |
| Grain size (µm) | 212-350 | 90-212 | 212-350 | 90-212 | 212-350 | 90-212 | 212-350 |
| Long-term water content (%) | 15 ± 5 | 15 ± 5 | 5 ± 2 | 2.0 ± 0.2 | 2.0 ± 0.2 | 10 ± 2 | 10 ± 2 |
| Internal dose rate (µGy/a) | 30 ± 10 | 30 ± 10 | 30 ± 10 | 30 ± 10 | 30 ± 10 | 30 ± 10 | 30 ± 10 |
| Alpha dose rate (µGy /a) | 53 ± 30 | 105 ± 64 | 30 ± 17 | 103 ±61 | 52 ± 29 | 55 ± 31 | 27 ± 14 |
| Beta dose rate (µGy/a) | 1294 ± 147 | 1390 ± 157 | 899 ± 63 | 1818 ± 114 | 1699 ± 107 | 1459 ± 58 | 1369 ± 56 |
| Gamma dose rate (µGy/a) | 1581 ± 147 | 1581 ± 144 | 936 ± 51 | 1675 ± 91 | 1675 ± 91 | 1103 ± 40 | 1103 ± 40 |
| Cosmic dose rate (µGy/a) | 16 ± 2 | 16 ± 2 | 55 ± 6 | 29 ± 3 | 29 ± 3 | 52 ± 5 | 40 ± 52 |
| Total dose rate (µGy/a) | 2974 ± 209 | 3122 ± 223 | 1950 ± 84 | 3655 ± 158 | 3486 ± 143 | 2698 ± 78 | 2581 ± 72 |
| D_e_ (Gy) Al centre | 930 ± 261 | n.c. | 215 ± 98 | 927 ± 128 | 1120 ± 152 | 1411 ± 7775 | 669 ± 194 |
| D_e_ (Gy) Ti centre (option D) | n.c. | n.c. | n.c. | n.c. | 7166 ± 1750 | n.c. | 1779 ± 1464 |
| **Age (ka) Al centre** | 312.7 ± 90.5 | n.c. | 110.3 ± 50.5 | 253.6 ± 36.7 | 321.3 ± 45.6 | n.c. | 259.2 ± 75.5 |
| **Age (ka) Ti centre (option D)** | n.c. | n.c. | n.c. | n.c. | 2055.7 ± 509.0 | n.c. | 689.3 ± 567.5 |

We cannot exclude that the apparent flat behavior of the Ti signal observed for 3 samples is an artefact resulting from an insufficient D_max_: a closer look at the low dose region of the DRCs from CBAIK10_212-350 µm (Fig. 7C) and CZW2_212-350 µm (Fig. 7E) shows that the first experimental data points are very scattered and do not show any significant increase as well (Fig. 7B and D). Consequently, it is possible that the Ti signal measured in these quartz samples are either in saturation or simply show a poor radiation sensitivity. Unfortunately, the quality of the ESR data derived from the measurement of the Ti centre is well below the standards commonly observed (e.g., ^220-221^). No meaningful dose estimate can be derived for these samples, precluding thus the use of the MC approach based on the comparison of Al and Ti signals. In first instance, the D_e_ results obtained from the Al signal should be regarded as maximum possible dose estimates for the samples: however, their reliability is directly dependent on the quality of the ESR data set collected, which is assessed by a series of proxies such as D_e_ repeatability, goodness-of-fit, impact of data weighting options, or magnitude of the error margin.

- 1. *ESR age results.*

ESR age results derived from the Al centre range from about 110 ka to 321 ka. Following the principles of the MC approach, these estimates should be regarded as maximum age constraints of the samples. They suggest that most samples have a late Middle Pleistocene age at the most. The apparent inconsistency observed for the two grain size fractions of sample CBAIK10 might simply be resulting from the regular repeatability of the ESR data set collected, although we cannot exclude that it might also reflect distinct transport and bleaching histories.

Ti ESR age estimates could be calculated for two samples only (CBAIK10_212-350 and CZW2_212-350), resulting in older results that for their Al equivalent. While this pattern cannot be explained by the MC approach, the low quality of the ESR data collected (scattered DRC, low repeatability, huge D_e_ errors) suggests that the pattern might not be significant. Consequently, the Ti age results should be treated with caution.

To sum up, the following considerations should be taken into account for the chronological interpretation of the ESR data at each site:

- Baikong Cave (CBAIK): The Al signal of sample CBAIK10_212-350 µm provides a more reliable age estimate than CBAIK10_90-212 µm since the D_e_ value is based on a much larger number of data points. From a methodological point of view, the age result of 401.0 ± 55.2 ka should be regarded as a maximum age constraint for this sample. This is at odds with the independent age control for this site.
- Queque Cave (CQQ): The Al signal of sample CQQ1 yields an age estimate of 110.3 ± 50.5 ka, which should in theory be regarded as a maximum age constraint for the deposits. However, the relatively low D_e_ repeatability and goodness-to-fit suggest that this result should be taken with caution especially considering the apparent discrepancy with the independent age control available for this site (Fig. 1d).
- Zhanwang Cave (CZW): Only one (CZW2_212-350μm) of the two samples returned finite ESR age results. The age estimates derived from the Ti centre should be taken with extreme caution given the low quality of the ESR data (poor goodness-of-fit; D_e_ repeatability >40%). The ESR result of 259.2 ± 75.5 ka based on the Al signal should be regarded as a maximum possible age constraint from a methodological point of view.
- Daxin Cave (CDAX): Only one single ESR age could be derived from the measurement of the Al and Ti centres in the two samples from that cave. The Al age calculated for CDAX_212-350μm of 312.7 ± 90.5 ka should be treated with caution given not only the massive interfering signal that most likely impacts the ESR intensity of the Al signal, but also the scatter in the D_e_ estimates derived from various fitting options (Table S18).

As a final comment, it seems like most of the ESR results derived from the Al centre may be somewhat underestimated in comparison with the independent age control available at each site. While this most likely result from an underestimated D_e_ estimate, this cannot be explained by a low thermal stability and/or saturation of the ESR signal, since the Al centre is frequently being used to date samples older than 2 Ma-old. Therfore, we suspect that the underestimation might originate from grain averaging effect. Since ESR analyses are based on multi-grain aliquots, the ESR intensities measured are naturally the result of the individual contribution of tens of thousands to hundreds of thousands of grains^220^. However, since the homogeneity of the quartz samples cannot be properly evaluated in the absence of single grain ESR analyses, we cannot exclude that if these samples were to include various populations of quartz with different bleaching histories or radiation sensitivity, the grain averaging effects on the ESR signals may lead to totally meaningless dose estimates (e.g., ^222^). Further research will evaluate the relative homogeneity of the quartz luminescence characteristics (e.g., radiation sensitivity, signal brightness) at a single grain level in order to obtain any comparative insights that could be extrapolated to the ESR data.

As of now, there is a set of evidence suggesting that the quartz samples have absorbed a massive radiation dose, as suggested by the (i) apparently flat DRCs and (ii) high ESR intensities obtained for the Ti centre in many samples. However, on the other side, the ESR intensity of the Ti signal seems to keep increasing at much higher doses above 2 kGy (Fig. S8). In other words, it does not show this non-monotonic behavior that is typically observed at high irradiation doses^212^. Moreover, the D_e_ estimates obtained from the Al signal are <1500 Gy, i.e., not exceptionally high. Consequently, we presently do not have any other explanation for the underestimated dose estimates, and ESR age results, obtained from the Al centre than some grain averaging effects due to highly heterogenous quartz samples, in addition to the fact that the robustness of the ESR data set is below usual standards, which is why data interpretation should anyway be treated with caution.

**SI section 8: U-series dating of carbonates and bone**

U-series dating of the overlying and underlying flowstones in the majority of the 22 caves provides a minimum/maximum age for the deposition of the breccia and associated *G.-blacki* and non-*G. blacki* fossils. The speleothem incorporated in situ flowstones that were sampled directly from the section using a hammer and chisel. The 40 flowstone blocks were processed using slightly different techniques in different research institutions. The majority of the samples were processed using MC-ICP-MS Radiogenic Isotope Facility at The University of Queensland (Methods 1), while five samples were processed using a laser ablation MC-ICP-MS at the Wollongong Isotope Geochronology Laboratory, University of Wollongong (Methods 2).

**Methods 1**

Separate sub-samples were drilled from the fresh cross-section of a hand specimen of the *in situ* flowstone using a hand-drill. The powdered sub-samples were subjected to chemical treatment and isotopic measurements by mass spectrometry^42^. U-series dating of the majority of speleothem samples was conducted in the Radiogenic Isotope Facility (The University of Queensland) using a Nu Plasma HR multi-collector inductively coupled mass spectrometer (MC-ICP-MS). Analytical procedures followed previous publications for MC-ICP-MS^43-45^. ^230^Th/^234^U ages were calculated using Isoplot EX 3.75^41^ and half-lives of 75,690 years (^230^Th) and 245,250 years (^234^U)^46^.

**Methods 2**

Analyses were undertaken by laser ablation multi-collector inductively-coupled plasma mass spectrometry (MC ICP-MS) at the Wollongong Isotope Geochronology Laboratory, University of Wollongong. Laser ablation was performed with a New Wave Research™ ArF 193 nm Excimer laser, equipped with a TV2 cell. Spot analyses were conducted with the following parameters: 150 µm spot size, 80% energy, 20 Hz repetition rate and 50 seconds ablation. Ablation was preceded by a pre-ablation of 2 seconds with a 150 µm spot size, 5% energy, 10 Hz repetition rate to clean the analysed location. Helium and nitrogen were used as carrier gases at a flow rate of 0.9 L/min and 10 mL/min, respectively. Thorium (^230^Th, ^232^Th) and uranium (^234^U, ^235^U, ^238^U) isotopes were measured on a Thermo Scientific™ Neptune Plus™ MC ICP-MS. All five isotopes were collected in static mode, with ^230^Th and ^234^U collected in ion counters. Helium flow rate and plasma parameters were tuned with NIST610 element standard to derive a ^232^Th/^238^U ratio for this standard greater than 0.8 and minimise differences in fractionation between Th and U ^222^.

Measured ^234^U/^238^U, ^230^Th/^238^U and ^232^Th/^238^U isotopic ratios were corrected for elemental fractionation and Faraday cup/SEM yield by comparing measured ratios with those of a 206 ka-old coral characterised independently by solution analysis. Uranium and Th concentrations were determined using NIST612 glass as calibration standard. Background subtraction and calculations of corrected ratios and concentration were performed using Iolite™ ^197^. For flowstones, detrital-corrected closed-system ^230^Th-U ages were calculated for each analysis using IsoPlotR^196^ with an assumed detrital (^230^Th/^232^Th) activity ratio of 0.8 ± 0.8. For sample CWUY-F1, because of the large amount of detrital Th, a single isochron age was calculated from the twelve analyses. Accuracy was assessed using a 124 ka-old coral (MK16;^194^) also characterised independently by solution analysis. Results yield (^234^U/^238^U) and (^230^Th/^238^U) ratios of 1.128 ± 0.012 and 0.762 ± 0.003 (1SE, *n*=44), respectively, within error of values determined by solution analysis (1.110 ± 0.002 and 0.764 ± 0.007, respectively; SD). The calculated ages for MK16 of the two analytical sessions conducted were 122.31 ± 1.07 ka (1 SE, *n*=9) and 121.16 ± 1.06 ka (1 SE, *n*=10). For the bone, 25 single analytical spot measurements were conducted along a transect perpendicular to the bone’s surface, and a single open-system ^230^Th-U model age was calculated using the R package *UThwigl* following the procedure described in ^197^, and with 10,000 iterations.

Results 1

Ages reported in Table S21 are from a total of nine caves from Chongzuo and Bubing Basin and range in age from 592-81 ka. In some samples Uranium loss was observed causing it to fall into the forbidden zone so an age could not be calculated.

**Table S21**: U-series data for flowstones, teeth and bone sampled in the caves of Southern China using the ICP-MS at the University of Queensland. Flowstone samples are denoted with a ‘F’ while teeth and bone samples are labelled as ‘Tooth’ and ‘Bone’

| **CAVES** | **Sample code** | **U (ppm)** | **^232^Th (ppb)** | **(^230^Th/ ^232^Th)** | **(^230^Th/ ^238^U)** | **(^234^U/ ^238^U)** | **Uncorr. Age (ka)** | **Corr. Age (ka)** | **Corr. Age II (ka)** | **corr. Initial (^234^U/ ^238^U)** |
| --- | --- | --- | --- | --- | --- | --- | --- | --- | --- | --- |
| SHUANGTAN | **CSHTC_F1** | 0.8484 ± 0.0004 | 1526.1 ± 16.9 | 1.96 ± 0.02 | 1.163 ± 0.005 | 1.143 ± 0.001 | 393 ± 15 | 347 ± 346 |  | 1.75 ± 0.52 |
|  | **CSHTC_F2-A** | 0.3211 ± 0.0003 | 178.45 ± 0.41 | 5.80 ± 0.02 | 1.063 ± 0.003 | 1.055 ± 0.001 | 476 ± 26 | 462 ± 68 |  | 1.24 ± 0.03 |
|  | **CSHTC_F2-B** | 0.5826 ± 0.0004 | 538.27 ± 1.82 | 3.56 ± 0.02 | 1.085 ± 0.004 | 1.067 ± 0.002 | 519 ± 43 | 495 ± 216 |  | 1.36 ± 0.18 |
|  | **CSHTC_F2-CB1** | 0.3958 ± 0.0003 | 118.26 ± 0.16 | 10.67 ± 0.05 | 1.051 ± 0.005 | 1.042 ± 0.002 | 537 ± 67 | 530 ± 80 |  | 1.20 ± 0.04 |
|  | **CSHTC_F2-CB2** | 0.6493 ± 0.0006 | 306.71 ± 0.89 | 6.77 ± 0.03 | 1.054 ± 0.004 | 1.041 ± 0.001 | 592 ± 89 | 580 ± 150 |  | 1.24 ± 0.09 |
|  | **CSHTC_F3-A** | 0.2666 ± 0.0002 | 48.15 ± 0.11 | 17.42 ± 0.07 | 1.037 ± 0.003 | 1.023 ± 0.002 | >500 ka |  |  |  |
|  | **CSHTC_F3-B** | 0.2766 ± 0.0001 | 42.28 ± 0.09 | 20.59 ± 0.1 | 1.037 ± 0.005 | 1.029 ± 0.003 | 597 ± 131 | 593 ± 133 |  | 1.16 ± 0.05 |
|  | **CSHTC_Tooth** | 20.4289 ± 0.0159 | 2.61 ± 0.03 | 27605 ± 307 | 1.162 ± 0.004 | 1.235 ± 0.002 | 245 ± 3 | 245 ± 3 |  | 1.47 ± 0.003 |
|  | **CSHTC-F1B-2017** | 0.6383 ± 0.0006 | 686.94 ± 1.6 | 3.03 ± 0.01 | 1.076 ± 0.003 | 1.053 ± 0.002 | 818 ± 469 | 795 ± 2945 |  | 1.71 ± 5.78 |
|  | **CSHTC-F1C-2017** | 0.5277 ± 0.0006 | 395.03 ± 0.88 | 4.28 ± 0.02 | 1.056 ± 0.004 | 1.074 ± 0.001 | 351 ± 9 | 330 ± 33 |  | 1.24 ± 0.02 |
|  |  |  |  |  |  |  |  |  |  |  |
| BAPENG | **CBAPC_Tooth_01** | 49.8981 ± 0.027 | 205 ± 0.57 | 596 ± 3 | 0.8102 ± 0.004 | 1.568 ± 0.002 | 75 ± 1 | 75 ± 1 |  | 1.703 ± 0.003 |
|  | **CBAPC_Tooth_02a** | 2.385 ± 0.0041 | 3096 ± 9.9 | 4.36 ± 0.2 | 1.8672 ± 0.0836 | 1.427 ± 0.005 | U loss falling into forbidden zone | | | |
|  | **CBAPC_Tooth_02b** | 7.1994 ± 0.0047 | 104 ± 0.22 | 669 ± 2 | 3.1764 ± 0.0077 | 2.119 ± 0.003 | U loss falling into forbidden zone | | | |
|  | **CBAPC_F1** | 0.1775 ± 0.0001 | 4.28 ± 0.01 | 2.29 ± 0.04 | 0.0182 ± 0.0003 | 1.143 ± 0.001 | 1.75 ± 0.03 | 1.12 ± 0.32 |  | 1.144 ± 0.001 |
|  | **CBAPC_F1-B** | 0.1048 ± 0.0001 | 5.54 ± 0.01 | 1.41 ± 0.03 | 0.0245 ± 0.0004 | 1.123 ± 0.002 | 2.41 ± 0.04 | 1 ± 0.71 |  | 1.125 ± 0.002 |
|  | **CBAPC_F2** | 0.1201 ± 0.0001 | 152 ± 0.37 | 2.67 ± 0.03 | 1.115 ± 0.0134 | 1.069 ± 0.003 | >500 ka |  |  |  |
|  | **CBAPC_F3** | 0.05368 ± 0.00002 | 10.19 ± 0.02 | 16.61 ± 0.19 | 1.0392 ± 0.0118 | 1.014 ± 0.004 | >500 ka |  |  |  |
|  | **CBAPC_Bone** | 0.0131 ± 0.00001 | 3.74 ± 0.01 | 12.73 ± 0.3 | 1.2000 ± 0.0281 | 1.079 ± 0.004 | U loss |  |  |  |
|  | **CBAPC-F1-17** | 0.0717 ± 0.00002 | 145 ± 0.13 | 1.76 ± 0.01 | 1.1777 ± 0.0079 | 1.096 ± 0.002 | ~500 | ~500 |  |  |
| YANLIANG |  |  |  |  |  |  |  |  |  |  |
|  | **CYANC-F1-17** | 0.264 ± 0.0002 | 26.23 ± 0.027 | 21.26 ± 0.05 | 0.696 ± 0.002 | 1.097 ± 0.001 | 108 ± 1 | 105 ± 1 |  | 1.134 ± 0.002 |
|  | **CYANC-F2** | 0.1887 ± 0.0001 | 93.76 ± 0.156 | 6.68 ± 0.02 | 1.094 ± 0.003 | 1.072 ± 0.001 | 551 ± 44 | 539 ± 145 |  | 1.382 ± 0.136 |
|  |  |  |  |  |  |  |  |  |  |  |
|  | A |  |  |  |  |  |  |  |  |  |
| YIXIANTIAN |  |  |  |  |  |  |  |  |  |  |
|  |  |  |  |  |  |  |  |  |  |  |
|  | **CYIXC-F1-2017** | 0.1205 ± 0.0002 | 12.14 ± 0.24 | 146 ± 3 | 0.894 ± 0.002 | 1.052 ± 0.003 | 200 ± 2 | 197 ± 3 |  | 1.052 ± 0.003 |
|  | **CYIXC-F2-2017** | 0.14994 ± 0.00005 | 51.11 ± 0.06 | 9.35 ± 0.03 | 1.050 ± 0.004 | 1.030 ± 0.002 | >500 | >500 |  |  |
|  |  |  |  |  |  |  |  |  |  |  |
| BAXIAN |  |  |  |  |  |  |  |  |  |  |
|  | **CBAXC-F1** | 0.29778 ± 0.0001 | 45.30 ± 0.056 | 3.29 ± 0.04 | 0.165 ± 0.002 | 1.130 ± 0.002 | 17 ± 0.2 | 13 ± 2 |  | 1.141 ± 0.004 |
|  | **CBAXC-F2a** | 0.9355 ± 0.0004 | 176.27 ± 0.179 | 12.15 ± 0.04 | 0.754 ± 0.003 | 1.058 ± 0.002 | 134 ± 1 | 128 ± 3 |  | 1.088 ± 0.003 |
|  | **CBAXC-F2b** | 0.7013 ± 0.0003 | 207.90 ± 0.26 | 8.08 ± 0.03 | 0.789 ± 0.003 | 1.070 ± 0.001 | 143 ± 1 | 134 ± 4 |  | 1.111 ± 0.005 |
|  |  |  |  |  |  |  |  |  |  |  |
| XIAO KOU | **CXK-18-F2** | 0.6960 ± 0.0008 | 3281 ± 4 | 0.870 ± 0.003 | 1.358 ± 0.004 | 1.1232 ± 0.0017 | U loss falling into forbidden zone | | | |
|  | **CXK-18-F3** | 0.8822 ± 0.0019 | 4273 ± 63 | 0.88 ± 0.02 | 1.397 ± 0.021 | 1.1637 ± 0.0011 | U loss falling into forbidden zone | | | |
|  | **CXK-18-F3** | 0.8802 ± 0.0013 | 4252 ± 58 | 0.87 ± 0.02 | 1.383 ± 0.018 | 1.1600 ± 0.0018 | U loss falling into forbidden zone | | | |
|  | **CXK-18-F4** | 3.2870 ± 0.0054 | 845 ± 7 | 6.60 ± 0.08 | 0.559 ± 0.005 | 1.0574 ± 0.0007 | 81 ± 1 | 74 ± 4 |  | 1.076 ± 0.003 |
|  | **CXK-18-F4** | 3.2932 ± 0.0060 | 842 ± 9 | 6.60 ± 0.01 | 0.556 ± 0.006 | 1.0580 ± 0.0007 | 81 ± 1 | 73 ± 4 |  | 1.077 ± 0.003 |
|  |  |  |  |  |  |  | ***81.0*** | ***73.7*** |  |  |
| QUEQUE | **QQ-F1** | 0.1389 ± 0.0001 | 114.65 ± 0.2 | 4.253 ± 0.015 | 1.157 ± 0.004 | 1.136 ± 0.002 | 405 ± 13 | 386 ± 111 | **392 ± 75** | 1.491 ± 0.075 |
|  | **QQ-F2** | 0.1421 ± 0.0001 | 136.68 ± 0.25 | 3.768 ± 0.012 | 1.194 ± 0.003 | 1.156 ± 0.002 | 443 ± 18 | 422 ± 212 | **428 ± 141** | 1.644 ± 0.206 |
|  |  |  |  |  |  |  |  |  |  |  |
| ZHANWANG |  |  |  |  |  |  |  |  |  |  |
|  | **CZW-F1** | 0.192 ± 0.0002 | 298.83 ± 0.69 | 2.392 ± 0.009 | 1.227 ± 0.004 | 1.137 ± 0.0016 | Minor U loss, falling into Forbidden Zone | | |  |
|  | **CZW-F2** | 0.1294 ± 0.0001 | 51.79 ± 0.1 | 7.851 ± 0.035 | 1.036 ± 0.004 | 1.138 ± 0.0023 | 233 ± 4 | 223 ± 8 | **226 ± 6** | 1.284 ± 0.009 |
|  | **CZW-F3** | 0.1349 ± 0.0001 | 8.95 ± 0.02 | 46.79 ± 0.17 | 1.024 ± 0.003 | 1.112 ± 0.002 | 247 ± 3 | 245 ± 3 | **245 ± 3** | 1.227 ± 0.003 |
|  |  |  |  |  |  |  |  |  |  |  |
|  |  |  |  |  |  |  |  |  |  |  |
|  |  |  |  |  |  |  |  |  |  |  |
| UPPER PUBU |  |  |  |  |  |  |  |  |  |  |
|  | **CUPBC-F1** | 0.02730 ± 0.00002 | 116.9 ± 0.3 | 0.531 ± 0.005 | 0.748 ± 0.006 | 1.339 ± 0.003 | 86 ± 1` |  |  |  |
|  | **CUPBC-F2** | 0.02221 ± 0.00001 | 116 ± 0.2 | 0.457 ± 0.003 | 0.787 ± 0.006 | 1.322 ± 0.002 | 94 ± 1 |  |  |  |
|  |  |  |  |  |  |  |  |  |  |  |
|  |  |  |  |  |  |  |  |  |  |  |

**Table S22**: U-series data from flowstones sampled in the caves of Southern China measured by laser ablation MC-ICP-MS at University of Wollongong

| **CAVE** | **Sample** | **U (ppm)** | **Th (ppb)** | **(^230^Th/^232^Th)** | **(^230^Th/^238^U)** | **(^234^U/^238^U)** | **Uncorr. age (ka)** | **Corr. age (ka)** | **Corr. initial (^234^U/^238^U)** |
| --- | --- | --- | --- | --- | --- | --- | --- | --- | --- |
| **YANLIANG** | Bone_1 | 21.0 ± 1.1 | 15.7 ± 2.1 | 5255.8 ± 38.4 | 1.272 ± 0.009 | 1.2706 ± 0.069 |  |  |  |
|  | Bone_2 | 15.6 ± 1.0 | 8.5 ± 0.8 | 6946.5 ± 69.5 | 1.299 ± 0.013 | 1.2923 ± 0.0073 |  |  |  |
|  | Bone_3 | 11.5 ± 0.9 | 7.8 ± 0.9 | 5566.0 ± 42.6 | 1.308 ± 0.010 | 1.3234 ± 0.0092 |  |  |  |
|  | Bone_3 | 11.5 ± 0.9 | 7.8 ± 0.9 | 5566.0 ± 42.6 | 1.308 ± 0.010 | 1.3234 ± 0.0092 |  |  |  |
|  | Bone_4 | 16.3 ± 0.7 | 15.7 ± 2.0 | 3953.7 ± 30.9 | 1.281 ± 0,010 | 1.2759 ± 0.0077 |  |  |  |
|  | Bone_5 | 16.4 ± 1.2 | 12.1 ± 4.8 | 5072 ± 44.0 | 1.268 ± 0.011 | 1.2636 ± 0.0066 |  |  |  |
|  | Bone_6 | 16.7 ± 1.0 | 10.7 ± 1.9 | 5874.4 ± 55.8 | 1.263 ± 0.012 | 1.2795 ± 0.0083 |  |  |  |
|  | Bone_7 | 17.6 ± 0.9 | 8.7 ± 1.9 | 7987.6 ± 86.9 | 1.286 ± 0.014 | 1.3118 ± 0.0067 |  |  |  |
|  | Bone_8 | 14.5 ± 0.6 | 3.6 ± 0.6 | 15737.5 ± 162.5 | 1.259 ± 0.013 | 1.2704 ± 0.0055 |  |  |  |
|  | Bone_9 | 17.4 ± 1.2 | 7.7 ± 1.8 | 9086.3 ± 86.3 | 1.263 ± 0.012 | 1.2797 ± 0.0065 |  |  |  |
|  | Bone_10 | 15.9 ± 1.0 | 4.67 ± 0.7 | 13853.5 ± 148.6 | 1.305 ± 0.014 | 1.3179 ± 0.0073 |  |  |  |
|  | Bone_11 | 17.0 ± 0.9 | 4.02 ± 0.5 | 16265.8 ± 177.2 | 1.285 ± 0.014 | 1.3303 ± 0.0053 |  |  |  |
|  | Bone_12 | 13.1 ± 1.5 | 9.4 ± 2.4 | 5840.9 ± 81.8 | 1.285 ± 0.018 | 1.351 ± 0.0110 |  |  |  |
|  | Bone_13 | 17.3 ± 0.8 | 8.0 ± 2.5 | 8391.0 ± 89.7 | 1.309 ± 0.014 | 1.3609 ± 0.0075 |  |  |  |
|  | Bone_14 | 17.4 ± 0.7 | 4.4 ± 0.8 | 15623.5 ± 152.9 | 1.328 ± 0.013 | 1.3697 ± 0.0086 |  |  |  |
|  | Bone_15 | 17.7 ± 1.3 | 4.4 ± 0.7 | 16412.5 ± 125.0 | 1.313 ± 0.010 | 1.3286 ± 0.0062 |  |  |  |
|  | Bone_16 | 16.7 ± 1.0 | 4.3 ± 1.1 | 16135.8 ± 160.5 | 1.307 ± 0.013 | 1.3162 ± 0.0063 |  |  |  |
|  | Bone_17 | 17.8 ± 1.1 | 2.4 ± 1.4 | 30953.5 ± 232.6 | 1.331 ± 0.010 | 1.3459 ± 0.0065 |  |  |  |
|  | Bone_18 | 16.1 ± 1.4 | 2.4 ± 0.6 | 28193.5 ± 236.6 | 1.311 ± 0.011 | 1.325 ± 0.0080 |  |  |  |
|  | Bone_19 | 16.5 ± 1.2 | 3.2 ± 0.6 | 21285.7 ± 190.5 | 1.341 ± 0.012 | 1.3379 ± 0.0075 |  |  |  |
|  | Bone_20 | 16.8 ± 1.1 | 6.0 ± 0.8 | 11469.6 ± 104.3 | 1.319 ± 0.012 | 1.3117 ± 0.0067 |  |  |  |
|  | Bone_21 | 16.3 ± 1.0 | 2.6 ± 0.3 | 23703.0 ± 178.9 | 1.325 ± 0.010 | 1.3239 ± 0.0080 |  |  |  |
|  | Bone_22 | 19.1 ± 1.9 | 6.0 ± 1.1 | 11408.7 ± 95.7 | 1.312 ± 0.011 | 1.3069 ± 0.0075 |  |  |  |
|  | Bone_23 | 17 ± 1.4 | 7.1 ± 0.7 | 9500 ± 114.3 | 1.330 ± 0.016 | 1.3346 ± 0.0086 |  |  |  |
|  | Bone_24 | 16.4 ± 1.2 | 5.0 ± 1.0 | 13336.7 ± 112.2 | 1.307 ± 0.011 | 1.309 ± 0.0130 |  |  |  |
|  | Bone_25 | 16.7 ± 1.8 | 12.1 ± 2.1 | 4607.4 ± 55.6 | 1.244 ± 0.015 | 1.2468 ± 0.0087 |  |  |  |
|  | **Open-system model age:** | | **680 +217-188** |  |  |  |  |  |  |
|  |  |  |  |  |  |  |  |  |  |
| **MAFENG** | CMF_F3_1 | 0.286 ± 0.015 | 17.6 ± 4.5 | 64.4 ± 4.3 | 1.294 ± 0.086 | 1.397 ± 0.057 | 217.8 ± 48.9 | 216.8 ± 48.5 | 1.7321 ± 0.0942 |
|  | CMF_F3_2 | 0.332 ± 0.011 | 24.5 ± 3.9 | 49.2 ± 3.1 | 1.195 ± 0.075 | 1.384 ± 0.054 | 183.2 ± 32.8 | 181.9 ± 32.4 | 1.6416 ± 0.0739 |
|  | CMF_F3_3 | 0.257 ± 0.009 | 13.4 ± 1.4 | 63.9 ± 3.9 | 1.094 ± 0.066 | 1.378 ± 0.045 | 153.1 ± 21.7 | 152.2 ± 21.5 | 1.5808 ± 0.0591 |
|  | CMF_F3_5 | 0.314 ± 0.009 | 39.2 ± 3.6 | 27.3 ± 2.1 | 1.120 ± 0.086 | 1.388 ± 0.037 | 157.9 ± 26.7 | 155.7 ± 26.3 | 1.6021 ± 0.0586 |
|  | CMF_F3_6 | 0.247 ± 0.019 | 16.7 ± 2.0 | 60.6 ± 4.1 | 1.406 ± 0.096 | 1.425 ± 0.06 | 261.3 ± 75.2 | 260.2 ± 74.5 | 1.8858 ± 0.1517 |
|  | CMF_F3_7 | 0.323 ± 0.008 | 17.5 ± 2.1 | 72.8 ± 4.3 | 1.318 ± 0.077 | 1.458 ± 0.037 | 200.8 ± 32.7 | 199.9 ± 32.5 | 1.8051 ± 0.0735 |
|  | CMF_F3_8 | 0.236 ± 0.011 | 16.5 ± 2.7 | 51.9 ± 5.1 | 1.220 ± 0.120 | 1.437 ± 0.032 | 174.2 ± 38.9 | 173.0 ± 38.5 | 1.7121 ± 0.0821 |
|  | CMF_F3_9 | 0.300 ± 0.017 | 20 ± 4.1 | 54.2 ± 4.2 | 1.237 ± 0.095 | 1.437 ± 0.042 | 179.7 ± 33.9 | 178.5 ± 33.6 | 1.7232 ± 0.0760 |
|  | CMF_F3_10 | 0.320 ± 0.016 | 50.2 ± 9.3 | 25.4 ± 2.3 | 1.320 ± 0.120 | 1.385 ± 0.069 | 237.6 ± 78.3 | 235.0 ± 76.6 | 1.7473 ± 0.1443 |
|  | **Weighted mean (n=9/9)** | |  |  |  |  | 187 ± 9 | **186 ± 9** |  |
|  |  |  |  |  |  |  |  |  |  |
|  | CMF_F4_1 | 0.239 ± 0.029 | 152 ± 45 | 5.2323 ± 0.4899 | 1.036 ± 0.097 | 1.277 ± 0.054 | 163.8 ± 37.1 | 151.0 ± 35.3 | 1.4242 ± 0.0743 |
|  | CMF_F4_2 | 0.203 ± 0.011 | 94 ± 16 | 7.0067 ± 0.4631 | 1.044 ± 0.069 | 1.262 ± 0.053 | 171.7 ± 31.6 | 162.1 ± 30.3 | 1.4139 ± 0.0699 |
|  | CMF_F4_3 | 0.252 ± 0.008 | 171 ± 27 | 4.6018 ± 0.2896 | 1.017 ± 0.064 | 1.255 ± 0.038 | 164.3 ± 25.6 | 149.6 ± 26.4 | 1.3889 ± 0.0514 |
|  | CMF_F4_4 | 0.310 ± 0.023 | 184 ± 37 | 5.328 ± 0.3228 | 1.007 ± 0.061 | 1.223 ± 0.043 | 171.8 ± 28.5 | 158.9 ± 28.1 | 1.3492 ± 0.0578 |
|  | CMF_F4_5 | 0.261 ± 0.023 | 135 ± 16 | 5.5756 ± 0.3721 | 0.959 ± 0.064 | 1.229 ± 0.034 | 152.8 ± 23.2 | 141.0 ± 23.6 | 1.3409 ± 0.0461 |
|  | CMF_F4_6 | 0.232 ± 0.015 | 114 ± 15 | 6.0435 ± 0.3665 | 0.973 ± 0.059 | 1.242 ± 0.043 | 153.5 ± 22.9 | 142.7 ± 23.1 | 1.362 ± 0.0561 |
|  | CMF_F4_7 | 0.232 ± 0.016 | 110 ± 16 | 6.1962 ± 0.462 | 0.979 ± 0.073 | 1.243 ± 0.044 | 155.2 ± 27.3 | 144.6 ± 26.8 | 1.3655 ± 0.0589 |
|  | CMF_F4_9 | 0.229 ± 0.026 | 87 ± 11 | 8.7097 ± 1.0484 | 1.080 ± 0.130 | 1.244 ± 0.065 | 193.6 ± 65.9 | 185.7 ± 61.9 | 1.4121 ± 0.1012 |
|  | CMF_F4_10 | 0.258 ± 0.013 | 141 ± 21 | 5.5028 ± 0.4199 | 0.996 ± 0.076 | 1.249 ± 0.052 | 159.0 ± 30.2 | 146.9 ± 29.3 | 1.3769 ± 0.0686 |
|  | CMF_F4_11 | 0.360 ± 0.023 | 242 ± 47 | 4.1843 ± 0.1935 | 0.908 ± 0.042 | 1.238 ± 0.036 | 135.0 ± 14.4 | 119.9 ± 18.9 | 1.3338 ± 0.0452 |
|  | CMF_F4_12 | 0.467 ± 0.074 | 237 ± 65 | 5.0253 ± 0.3165 | 0.794 ± 0.050 | 1.260 ± 0.034 | 103.5 ± 11.6 | 92.8 ± 14.7 | 1.3378 ± 0.0412 |
|  | **Weighted mean (n=9/11)** | |  |  |  |  | 162 ± 5 | **150 ± 4** |  |
|  |  |  |  |  |  |  |  |  |  |
|  | CMF_F5_1 | 0.130 ± 0.008 | 26.4 ± 2.3 | 14.4309 ± 1.3657 | 0.951 ± 0.090 | 1.376 ± 0.058 | 118.7 ± 21.3 | 114.9 ± 20.9 | 1.520 ± 0.0725 |
|  | CMF_F5_2 | 0.098 ± 0.01 | 17.0 ± 3.6 | 14.5455 ± 2.0000 | 0.80 ± 0.110 | 1.292 ± 0.068 | 100.3 ± 23.5 | 96.8 ± 22.9 | 1.3837 ± 0.0833 |
|  | CMF_F5_3 | 0.098 ± 0.004 | 8.6 ± 1.4 | 34.2657 ± 4.5455 | 0.980 ± 0.130 | 1.357 ± 0.083 | 128.5 ± 34.1 | 126.9 ± 33.6 | 1.5107 ± 0.1063 |
|  | CMF_F5_4 | 0.169 ± 0.009 | 10.8 ± 2.7 | 41.8687 ± 4.0404 | 0.829 ± 0.08 | 1.412 ± 0.062 | 91.3 ± 14.6 | 90.2 ± 14.5 | 1.5314 ± 0.0732 |
|  | CMF_F5_5 | 0.209 ± 0.009 | 9.6 ± 2.2 | 50.1183 ± 4.0237 | 0.847 ± 0.068 | 1.398 ± 0.053 | 95.9 ± 13.2 | 94.9 ± 13.1 | 1.5202 ± 0.0630 |
|  | CMF_F5_6 | 0.154 ± 0.023 | 26.0 ± 15 | 17.3077 ± 2.3077 | 0.900 ± 0.120 | 1.381 ± 0.063 | 107.7 ± 24.7 | 104.6 ± 24.2 | 1.5119 ± 0.0798 |
|  | CMF_F5_7 | 0.175 ± 0.008 | 12.1 ± 4 | 37.6959 ± 4.4239 | 0.818 ± 0.096 | 1.339 ± 0.055 | 97.7 ± 18.9 | 96.4 ± 18.8 | 1.4449 ± 0.0676 |
|  | CMF_F5_8 | 0.151 ± 0.009 | 4.8 ± 1.4 | 68.0000 ± 9.600 | 0.850 ± 0.120 | 1.325 ± 0.061 | 105.7 ± 25.4 | 104.9 ± 25.3 | 1.4369 ± 0.0775 |
|  | CMF_F5_9 | 0.136 ± 0.003 | 9.1 ± 1.9 | 37.7626 ± 3.242 | 0.827 ± 0.071 | 1.365 ± 0.072 | 96.2 ± 15.2 | 94.9 ± 15 | 1.4771 ± 0.0847 |
|  | CMF_F5_10 | 0.141 ± 0.012 | 14.3 ± 2.2 | 27.8287 ± 3.3639 | 0.910 ± 0.110 | 1.322 ± 0.057 | 118.8 ± 26.4 | 116.8 ± 26 | 1.4478 ± 0.0741 |
|  | **Weighted mean (n=10/10)** | |  |  |  |  | 102.9 ± 3.4 | **101 ± 3** |  |
|  |  |  |  |  |  |  |  |  |  |
| **WUYUN** | CWUY_F1_1 | 0.319 ± 0.021 | 765 ± 53 | 1.384 ± 0.187 | 1.110 ± 0.150 | 1.004 ± 0.058 |  |  |  |
|  | CWUY_F1_2 | 0.380 ± 0.027 | 972 ± 97 | 1.091 ± 0.065 | 0.927 ± 0.055 | 0.990 ± 0.035 |  |  |  |
|  | CWUY_F1_3 | 0.510 ± 0.110 | 1050 ± 120 | 1.599 ± 0.461 | 1.180 ± 0.340 | 1.108 ± 0.097 |  |  |  |
|  | CWUY_F1_4 | 0.622 ± 0.059 | 1790 ± 230 | 1.05 ± 0.08 | 1.008 ± 0.077 | 0.979 ± 0.034 |  |  |  |
|  | CWUY_F1_5 | 0.412 ± 0.052 | 852 ± 62 | 1.724 ± 0.862 | 1.260 ± 0.630 | 1.080 ± 0.220 |  |  |  |
|  | CWUY_F1_6 | 0.329 ± 0.021 | 776 ± 57 | 1.195 ± 0.08 | 0.913 ± 0.061 | 1.017 ± 0.045 |  |  |  |
|  | CWUY_F1_7 | 0.450 ± 0.077 | 882 ± 56 | 1.513 ± 0.076 | 1.041 ± 0.052 | 1.031 ± 0.042 |  |  |  |
|  | CWUY_F1_8 | 0.422 ± 0.026 | 1090 ± 120 | 1.091 ± 0.07 | 0.922 ± 0.059 | 0.964 ± 0.035 |  |  |  |
|  | CWUY_F1_9 | 0.339 ± 0.022 | 799 ± 47 | 1.262 ± 0.071 | 0.978 ± 0.055 | 1.019 ± 0.034 |  |  |  |
|  | CWUY_F1_10 | 0.265 ± 0.015 | 650 ± 130 | 1.183 ± 0.111 | 0.946 ± 0.089 | 0.964 ± 0.041 |  |  |  |
|  | CWUY_F1_11 | 0.339 ± 0.022 | 851 ± 88 | 1.547 ± 0.507 | 1.250 ± 0.410 | 1.029 ± 0.072 |  |  |  |
|  | CWUY_F1_12 | 0.378 ± 0.069 | 810 ± 110 | 1.334 ± 0.067 | 0.974 ± 0.049 | 0.963 ± 0.049 |  |  |  |
|  | **Isochron age: 230 ± 103 ka (n=12)** | | |  |  |  |  |  |  |

Results 2

Ages reported in Table S21 are the weighted means of all ages for a given sample with their standard error (SE). For flowstones CMF-F3, F4, F5, uncorrected and corrected ages are within error, suggesting that detrital Th had a minimal role on calculated ages. Corrected ages range from 101 ± 3 to 186 ± 9 ka. For sample CWUY_F1, because of the large amount of detrital Th, a single isochron age was calculated from the twelve analyses using a closed system model^197^. This was performed using the R package IsoPlotR^195^. We obtained an isochron age for this sample of 230 ± 130 ka. This age is of little use considering the large uncertainty.

Discussion

Except for samples CMF-F3, F4, F5, the majority of the flowstones in these caves are composed of poor-quality calcite and have a high detrital component as reflected by the high levels of ^232^Th concentrations and very low ^230^Th/^232^Th activity ratios (especially those <2) that often afforded imprecise and unreliable age results. Such high levels of a detrital component were due to the fact that seepage water near cave entrances is usually muddier, resulting in incorporation of abundant silts into flowstone formations. Correlation for the contribution of ^230^Th from the detrital component toward ^230^Th/U ages will result in a large error magnification due to the uncertainty in the assumed ^230^Th/^232^Th value of the detrital component for calculation. In addition, it must be pointed out that samples that return a calculated age in excess of 500 ka should be treated with caution and considered as >500 ka with its older limit unconstrained. This is because the degree of U-Th disequilibrium in samples >500 ka is at the detection limit of the mass spectrometry. However, for open systems (e.g. bone), valid ^230^Th-U ages can exceed 500 ka. For flowstones CMF-F3, F4, F5, reliable corrected closed-system ages ranging from 101 ± 3 to 186 ± 9 ka were calculated. For the bone with 25 single analytical spot measurements, an age of 680 +217-188 ka was calculated, using the open-system diffusion adsorption model^197^. While the uncertainty is large, it gives an estimate of the minimum burial age of this bone. Age estimates <500 ka provide a useful minimum and maximum age range for overlying and underlying flowstones that bracket the breccia in most of these caves, and provide a vital independent age estimates for the the pIR-IRSL, coupled US-ESR and ESR dating techniques.

**SI section 9: Modelling of caves and EW**

The first step to addressing the issue of extinction is establishing the exact timing of the extinction window, but few sites have been dated radiometrically thus the chronology of the *G. blacki* faunas remains uncertain ^11^. At present *G. blacki* disappearance has been inferred to be late middle Pleistocene ~400-200 ka^2,6.9.107^, but in each of these cases the chronology has been established using dating of just a few teeth (e.g., ^2^) with no sedimentary context, or using cave deposits/and or fossils that may not be in situ (e.g., ^150^). None have dated the entire range of *G. blacki* in the fossil record nor provided chronologies for younger sites that do not contain *G. blacki* evidence. We have addressed these limitations by dating both the sedimentary and fossil context for both *G. blacki* bearing and non-bearing caves across a wide region.

The majority of age estimates from the six dating techniques were entered into an individual model for each cave – with 22 models in total being produced (see Fig. S11 for an example of the code and Fig. S12 for an example of modelling process both for Bapeng Cave). As the uncertainties for age estimates were presented at either 1 or 2 σ (as is customary for each specific dating technique, e.g., all trapped charge techniques; pIR-IRSL, OSL and ESR dating on sediments are presented at 1 σ, while U-series dating of carbonates and teeth are presented at 2 σ, as coupled US-ESR is a mixture of the two it is also presented at 1 σ ) – all uncertainties were converted to 1 σ for use in the model, but to be increasingly conservative in the the age ranges of the caves the model outputs are presented at 2 σ.

A small number of data were excluded from the age models based on methodological issues. For U-series dating of flowstones at Shuangtan, the upper F1 proved problematic due to high Th contamination issues resulting in a very imprecise corrected age. Thus, samples CSHT-F1, and CSHT-F1B-2017 (Table S21) were not used in the model for this reason. In addition, the ESR dating of quartz also proved problematic at some sites (as explained in Supp section 7). Therefore, samples QQ1 and CBAIK10 were excluded from the model due to the low quality of the ESR data with a low goodness-of-fit and D_e_ precision. Furthermore, they represent maximum ages estimates, which are at odds with the other independent age estimates for the site. The coupled US-ESR dating of teeth at Baikong and Yanliang also proved problematic with Tooth 858 (Baikong) proving unreliable and therefore was not included in the model. Tooth 859 (Baikong) and 806 (Yanliang) contained high U content in the enamel (ca. 4 ppm) and could reasonably be considered as underestimating so it was entered in the model as a minimum age estimate. In all, only 6 out of 157 age estimates were excluded from the model.

The models were structured using phases and boundaries in a contiguous pattern, with each stratigraphic unit representing a separate phase. The aim of the modelling was to estimate the age of the boundaries between each stratigraphic unit based on the dating results obtained for that unit. No attempt was made to remove identified outliers. This is because we do not know the underlying ‘true’ age depth model, and we are using several different dating methods, so it is difficult to specify the criterion to identify true outliers.

Rather than this approach, we have explicitly specified minimum and maximum ages where appropriate to do so, in-keeping with the nature of the dating methods and the quality of the results. For example, U-series dating on bone and teeth are presented as minimum age estimates due to fact that the technique dates the migration of uranium into the skeletal tissues during burial, which may not necessarily be the initial burial time. In contrast, pIR-IRSL single-aliquots are presented as maximum age estimates due to the averaging effect of partially bleached grains on each disc. However, as each single-aliquot contains only 1-2 grains that luminescence the averaging effect is present but minimal. Thus, we believe that the inclusion of the single-aliquots results, albeit as maximum ages, is still justifiable. The inclusdion of both U-series dating on teeth and bone and the pIR-IRSL aingle-aliquot age stimates into the model provides supporting data that help to sandwich the true burial age of the breccia.

We believe this is a superior method compared to outlier analysis in this context, as it avoids unnecessary bias (e.g. in the choice of criterion) and represents a more conservative approach.

**Fig S11**: An example of the Oxcal Bayesian code written for the Bapeng Cave chronology -all 22 codes are available in a public data repository (Zenodo DOI: 10.5281/zenodo.10077255)

Options()

{

Resolution=500;

};

Plot()

{

Sequence()

{

Boundary("silts to F3");

Phase("1")

{

Date("U-series 1", N(calBP(500000), 20000));

};

Boundary("F3 to G breccia");

Phase("2")

{

Date("US-ESR 1", N(calBP(324000), 44000));

Date("pIR SG 1", N(calBP(456000), 225000));

Date("U-series 2", N(calBP(309000), 14000));

Before()

{

Date("US 1 min", N(calBP(180000), 8000));

};

};

Boundary("G breccia to F2");

Phase("3")

{

Date("U-series 3", N(calBP(311000), 14000));

};

Boundary("F2 to upper seds");

Phase("4")

{

Date("pIR-SA1", N(calBP(148000), 39000));

};

Boundary("Upper seds to F1");

};

};

.
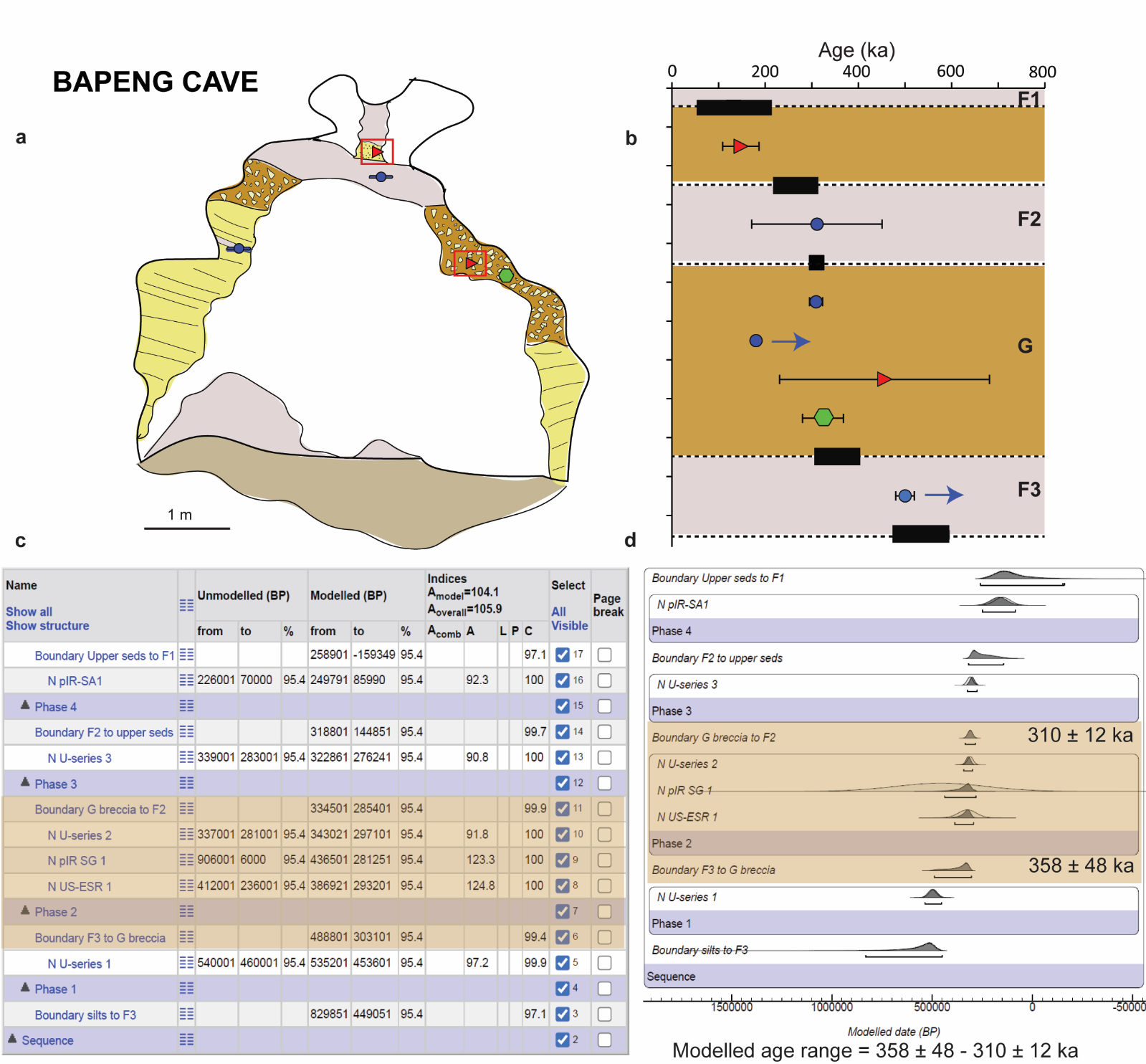


**Fig S12**: An example of the modelling process for Bapeng Cave. The stratigraphy drawing demonstrates the samping locations (a) and the age estimates plotted within their stratigraphic relationships (b). These age estimates were entered into the Bayesian model (n=157) with each data point representing the mean age with s.d. at 2 σ uncertainties (c) to constrain the boundaries above and below the *G. blacki* bearing layer (highlighted in brown on c and d). d is a multiple plot representing the sedimentary phases and boundaries with the modelled age range of 358 ± 48 to 310 ± 12 ka. The data points represent mean ages with s.d. at 2σ uncertainties.

The age estimates are coeval and the uncertainties are relatively small. As such, the identified boundary ages are not sensitive to removal of individual dates, or to changes in, for example, the model calculation resolution. None of the changes we made to the model set-up produced appreciable differences in the age model results. The age of the breccia in each cave was conservatively estimated as the boundary between the overlying flowstone unit and the age estimates of the breccia and fossils from within the breccia, incorporating all of the constraints described above (Supplementary Information sections 4-8) and the resulting age estimates (Supplementary Tables S11, 13-14,19-21). The final modelled ages are presented in Table S22 with the multiple plot presented in ED6. The age estimates from the breccia, derived from U-series and coupled US-ESR dating of teeth and pIR-IRSL and OSL_SG_ dating of breccia matrix are coeval within error margins despite constraining two different events, i.e., the death of the fauna and the deposition of the breccia unit, respectively. This indicates that the timing between death and final burial within most of the caves was very short and that on the whole the breccia was deposited rapidly as one unit.

**Table S23**: Modelled age range for all 22 caves and the EW

| **Area** | **Cave** | **Modelled age range upper** | **Modelled age range lower** |
| --- | --- | --- | --- |
| **CZ** | BAIKONG | 2350 ± 630 | 613 ± 203 |
| **BB** | CHUIFUNG | 2048 ± 266 | 1420 ± 378 |
| **CZ** | QUEQUE | 1151 ± 332 | 704 ± 150 |
| **CZ** | SANHE | 1145 ± 366 | 549 ± 280 |
| **CZ** | ZHANWANG | 973 ± 330 | 469 ± 230 |
| **CZ** | YIXIANTIAN | 765 ± 124 | 324 ± 69 |
| **CZ** | YANLIANG | 716 ± 154 | 310 ± 102 |
| **CZ** | DAXIN | 632 ± 251 | 426 ± 69 |
| **CZ** | BAPANG | 358 ± 46 | 310 ± 12 |
| **CZ** | HEJIANG | 307 ± 14 | 274 ± 24 |
| **CZ** | SHUANGTAN | 307 ± 35 | 273 ± 31 |
| **CZ** | GONGJISHAN | 252 ± 40 | 200 ± 41 |
| **CZ** | QUZAI | 214 ± 21 | 187 ± 21 |
| **CZ** | BAXIAN | 200 ± 32 | 143 ± 14 |
| **BB** | ZHONG SHAN | 182 ± 64 | 71 ± 7 |
| **CZ** | MAFENG | 171 ± 22 | 149 ± 11 |
| **BB** | WUYUN | 163 ± 48 | 76 ± 16 |
| **BB** | UPPER PUBU | 157 ± 55 | 110 ± 17 |
| **CZ** | XIAO KOU | 88 ± 8 | 82 ± 3 |
| **BB** | LOWER PUBU | 59 ± 18 | 33 ± 12 |
| **BB** | GANXIAN | 57 ± 13 | 10 ± 8 |
| **BB** | LUMEI | 24 ± 6 | 49 ± 5 |

**SI section 10: Pollen analysis**

**Methods**

Pollen analysis followed a modified standard methodology described by^49^, where 2.5 ml of sediment was dispersed in Calgon (3%) and placed in a low temperature water-bath for ½ hour followed by placement on an agitator overnight to further loosen sediment. A single Lycopodium tablet was added to calculate both the pollen and charcoal concentration of each sample. Samples were then treated with HCL (Hydrochloric acid 10%) in a warm bath to remove any carbonates and then sieved at > 125 µm and the > 125 µm fraction was kept for later inspection. The <125 µm fraction was then placed in 500 ml beakers with 500 ml of distilled H_2_O, allowed to settle for three hours and then the upper 400 ml poured or siphoned off. This was repeated until the water column was clear. The settling step removes clays and fine silts. HL (Heavy liquid/LST-Lithium heteropolytungstates) at a density of 2.01 SG and centrifuged at 2000 rpm for 1 hour was used to separate the remaining mineral matter from the organic fraction. Acetolysis which removes cellulose and stains the pollen followed. Acetolysis has three steps, CH_3_COOH (glacial acetic acid) wash, H_2_SO_4_/(CH_3_CO)_2_O (sulphuric acid/acetic anhydride) acetolysis solution step (1:9) in a water-bath at 90°C for up to 10 mins, and then a final CH_3_COOH wash. The remaining sample was then mounted on slides with glycerol with the cover slip sealed with nail polish. Glycerol aids identification and provides moveability on the slide. H_2_O (distilled) washes were completed across all steps. Pollen identification was aided by the Australasian Pollen and Spore Atlas (ANU online resource^50^), and the publication, An illustrated handbook of Quaternary pollen and spores in China^51^.

**Macrocharcoal**

Macrocharcoal analysis followed the methodology outlined by^52^, where 2.5 ml of sediment was dispersed in Calgon (3%) and placed in a low temperature water-bath for ½ hour, left for several days, and then placed on an agitator (slow moving) overnight to further loosen sediment. Samples were then treated with a low concentration (5%) of HCL and then placed in a bleach solution (2.6%) overnight, bleaching any plant material with the exception of charcoal. No vortexing/spinning or centrifuging was implemented to reduce the risk of breakage except prior to the macrocharcoal count were low-speed vortexing was necessary. Macrocharcoal samples were sieved at > 125 µm and > 250 µm prior to counting.

**Palynological results**

The final pollen results are presented for each cave in Fig 2, and on a timeline in Fig. 3. Overall the pollen counts were low as seen in other studies (e.g., ^21,103^), but to avoid the addition of anomamlous data points the data set presented represents only the sites that contained a minimum of 10 pollen counts.

**Modern surface samples**

The modern samples had good pollen recovery and a higher diversity of pollen types than the fossilised samples. Although the presence of *Pinus* may suggest a more temperate environment, the presence of *Alchornea*, Sapotaceae, *Trema*, *Mallotus* and Rubiaceae reflect a more sub-tropical or tropical environment. However, there are differences between the four sites with Xiao Kou, Bapeng and Shuangtan having a greater presence of ferns likely present as an understorey while Hejiang had high numbers of Urticaceae/Moraceae and Poaceae. Urticaceae especially has a diverse suite of plant structural types from trees, shrubs and herbs and with the corresponding high Poaceae values could suggest a more herbaceous origin for Urticaceae in the HEJ sample. The highest microcharcoal and macrocharcoal values are found in the HEJ sample which may be influenced by the presence of Poaceae. Dung fungi, *Sordaria*, *Podospora*, *Delitischia* and *Sporormiella*, are present but with low values in all modern samples.

**From 10,000 to 200,000 years ago**

The fossilised cave sediment samples are pollen poor with the majority of pollen grains degraded or squashed therefore limiting identification. However, there are a number of taxa that are consistently present. The gymnosperm *Pinus* which had high numbers in the modern samples are consistently present from 10,000 years ago to 200,000 years ago. Also present across this time-period are the angiosperms Myrtaceae, Betulaceae, Fagaceae, Poaceae, *Aster*, *Artemisia*, Amaranthaceae, Cyperaceae and ferns. Present in selected samples are *Mallotus/Macaranga*, Fabaceae, *Trema*, *Quercus*, Rhamnaceae, Sapindaceae and Brassicaceae. This mix of taxa includes both temperate and more sub-tropical types, with the relatively high grass and spores percentages, is indicative of a mosaic of sub-tropical/deciduous temperate forest cover with open grasslands. Higher charcoal and fern sper counts suggest the environment was subject to fire and disturbance that is typical of a seasonally dry climate that is also reflected in the Tianyang crater pollen record from the Leizhou Peninsula, southern China (Fig. 14, ^224^). During this period, we see large increase in forest disturbance/high turnover taxa such as *Trema, Celtis, Sapindaceae*. These taxa are used as an indication of changes in the forest plant communities and as a proxy for forest disturbance and are present during the transitional phase, EW and post EW.

**Before 200,000 years ago**

Prior to 200,000 years the pollen concentration is much poorer than in more recent sediment settings. *Pinus,* Fagaceae and Betulaceae are the dominant arboreal taxa with Myrtaceae and *Mallotus* present in selected samples. The Fagaceae type was not separated further due to poor pollen condition but the likely candidates for this type are either *Castanopsis* and *Lithocarpus* or *Castanea*. The dominance of *Pinus,* Fagaceae and Betulaceae, both temperate taxa, and the occational increase in subtropical taxa suggests the persistence of continuous forest cover including evergreen/deciduous forest and subtropical forest cover over time. The impact of glacial to interglacial climate change is likely to have influenced the dominace of different forest types over time (Fig. 14), with grasslands being relatively poortly represented prior to 200,000 years ago (Zheng and Lei 1999). Poaceae is in low numbers but is the dominant non-arboreal taxa while Cyperceae and ferns continue to be present. The increase in forest disturbance taxa starts during this period ~630 ka during a transitional phase of increasing environmental variability. Although low pollen numbers preclude a more detailed environmental reconstruction the taxa which are present and relatively low charcoal concentrations suggest a forested and relatively cooler and wetter/less seasonal conditions charcterise the region prior to 200,000 years ago.

Dung fungi is relatively rare with *Podospora*, *Sordaria* and *Sporormiella* present at CDAXC Upper 8 (350,000), *Sordaria* at CDAXC Upper 9 (350,000), *Podospora* at CHEJC G2 3.5 (351,000), *Sporormiella* at HEJ 02 (351,000) and *Sordaria* at BAP 01 (354,000). Although rare it is of interest that all the dung fungi presence is centred within the period 350,000 and 351,000 years ago.

**Discussion**

The wind pollinated nature of *Pinus, Betulaceae, Poaceae, Aster* and *Artemisia* could suggest their pollen grains were blown into the cave, although other modes of transport are also possible while the majority of the other taxa are likely to be influenced by invertebrate (entomophily) and vertebrate transport. *Cyperaceae*, and the ferns (spore) can be wind pollinated but are also influenced by surface hydrophily which depending on the magnitude of the event (cave inundation) may have an overall impact on all taxa although rarer. But what is clear is that the pollen is coming from a wider area than is represented by the faunal analysis and this is thus considered when interpreting both data sets. The location and altitude of the caves in relation to the plains also has a bearing on the pollen interpretation. The towering karst tends to be dominated by arboreal species due to their height so the resulting cave pollen is over represented by these species even when a shift to more non-arboreal species can be observed in most parts of Southeast Asia as early as ~800 ka (e.g., ^22^). The associated shift is observed in our data set as late as ~200 ka, this means that towering karst environments were experiencing slightly different vegetation changes during this period than seen in the lowlands and plains. Thus, the timing of this shift in seasonality can be observed slightly later in the pollen record than seen in the fauna, trace element and DMTA analyses.

Overall, the pollen displays an early dominance of a mosaic environments of forested species with some patches of grasses, which gives way to a mosaic of grasses with some forested areas. The pollen data also suggests some early phases of fern growth at ~1.2 ma and 600 ka and an additional phase indicating some disturbance right before the extinction window at ~350 ka. It is assumed that these pulses of disturbance had a direct effect on *G. blacki* populations. The final phase of disturbance is ~200 ka and is much larger than the previous changes and occurs in the post-extinction period. The data presented on a timeline in Fig. 3 displays a correlation between the pulses of fern expansion and the macrocharcoal indicating that the phases of disturbance were also characterisaed by natural burning. It is ironic that after the extinction window, the arboreal species experience a tempoary increase in numbers, as seen at the Baxian site, before steadily deteriorating over the next 200 kyrs.

In comparison to ^21^ palynological study of cave in this region, our study provides more details on how and when the mosaic environments shifted towards increased seasonality. Our study contains more caves over a wider age with more robust dating, therefore we are able to estimate the timing of the major changes and isolate those changes that directly impacted on *G. blacki* extinction.

**Fig S13a**: Pollen, spore and charcoal diagrams for all caves (excluding Yixiantian, Shuangtan Upper and Zhanwang). The diagram shows amalgamated sample data from distinct time periods at each cave setting, the oldest at the base to the youngest (including modern suface samples) at the top. The summary histogram shows the relative proportions of arboreal (green), non-arboreal (orange) and spores (pink) at each site alongside the NAP:AP ratio. Selected arboreal and non-arboreal pollen taxa % are calculated on the total pollen+spore+indetrminate sum. The total % of indeterminate (often squashed) pollen and spore taxa are also plotted (grey) alongised the total pollen+spore+indetrminate sum for each sample. The concentration of microcharcoal (<125 μm charcoal particles/cm^3^) and macrocharcoal (>125 μm charcoal particles/cm^3^) in each sample site are plotted as black histograms.

**Fig S13B**: A comparison of palaeoenvironmental features pre and post extinction (n=32 samples). **a** the relationship between arboreal, non-arboreal and ferns (same colours used as in S13a) in the pre-extinction period, draw as box plots where the box represents the first to the third quartile and the whiskers/error bars go from each quartile to the minimum or maximum. The median is represented by a vertical line through each box. **b** the same relationship in the post EW period. **c** the NAP:AP relationship pre extinction, **d** the same relationship post extinction. **e** the concentration of microcharcoal and macrocharcoal found in the cave sediments pre-extinction, **g** the concentration of microcharcoal and **h** macrocharcoal found in the cave sediments post-extinction. **i** the concentration of dung fungi found in the sediments pre extinction, and **j** – post extinction.

**
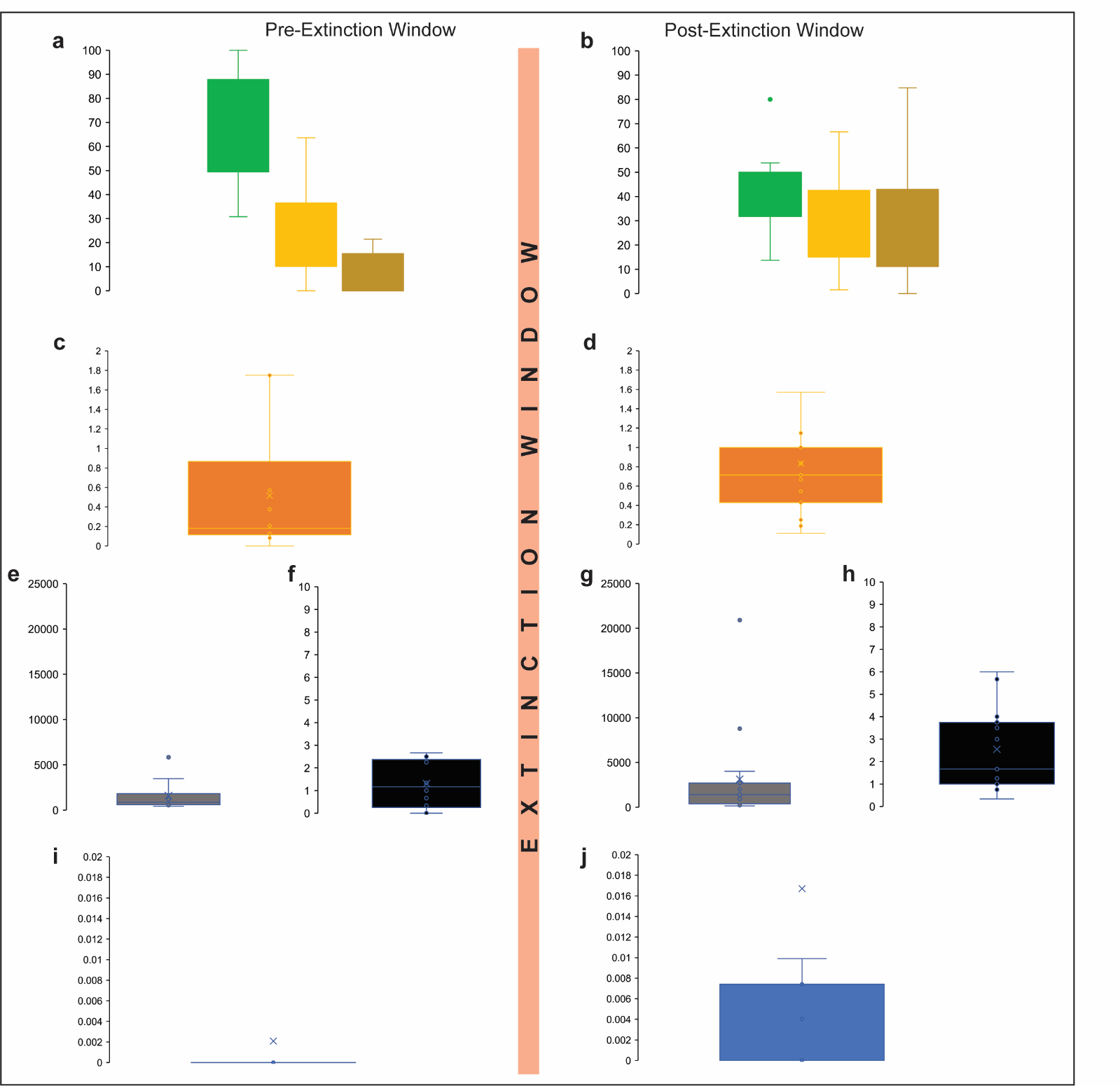
**

**Fig S14**: Comparison between (a) pollen and spore spectra for all caves analysed in this study dating to the last 400,000 years, and (b) the SPECMAP tuned Tianyang crater pollen record from the Leizhou Peninsula, southern China ^224^. The pollen regcords for grass show a consistent increase after ~200,000 years ago as more open and seasonally arid environments expand in the southeast region of China.


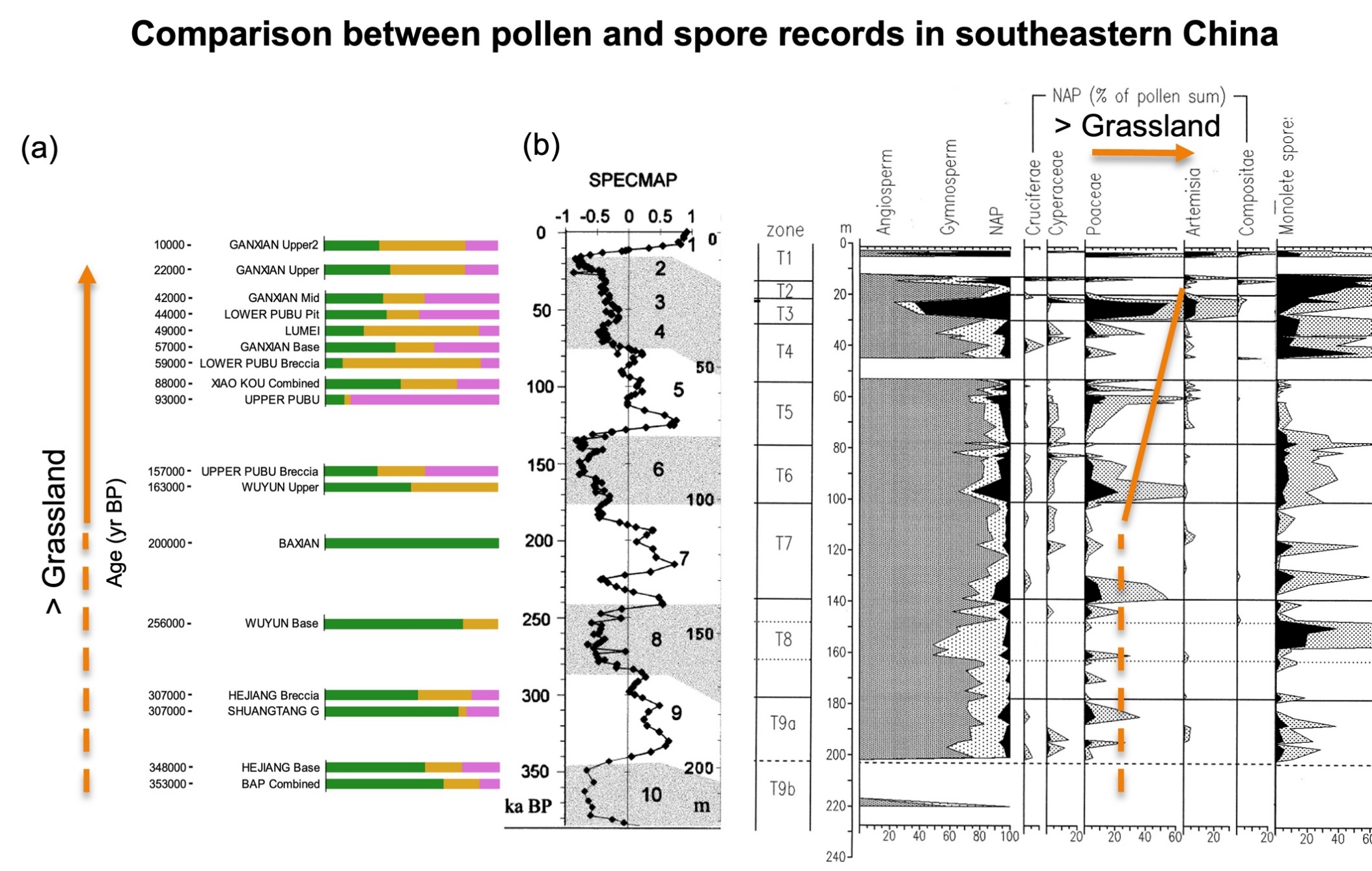


**SI section 11:** **Microstratigraphic analyses**

**Background**

Sediments from Liucheng Cave, in Guangxi, were analysed for palaeoenvironmental reconstructions related to *G. blacki*’s lifestyle by^147^. This represents the only other study in which local karst geomorphology and ‘macro’-sedimentology of the region is considered in association with *G. blacki* evidence. Sediment-based *micro-analytical techniques* have never been applied, but could be useful for studying the potential changes in environmental conditions leading up to the extinction. We present the first ever application of microstratographic techniques to *G. blacki*’s environment. Techniques include: *microstratigraphy*, *particle size analysis* (*PSA*), *alkalinity* tests (*pH*), *X-ray Diffraction* (*XRD*) and *X-ray Fluorescence* (*XRF*). Microstratigraphy (also called ‘micromorphology’), through petrographic microscopy of thin sections of in-situ sampled sediments, is widely being applied to study the diagenetic and depositional history of archaeological and, increasingly, palaeontological sites (*e.g.* ^225-231^). PSA^232,233^, is a standard technique in sedimentological and environmental related research, and alkalinity tests, XRD^234^ and XRF^235^ are essential to characterise and examine the mineralogical and geochemical aspects of (palaeo)environments, especially in poorly studied humid (sub)tropical regions^236^.

**Methods**

To assist in our understanding of the depositional environments associated with the fossil record, and better constrain the context of this material, five intact sediment blocks (~20x10x10 cm) were extracted from five caves, from the youngest to the oldest: Mafeng (CMF); Shuangtan (CSHT); Hejiang (CHEJ); Bapeng (CBAP); and Queque (CQQ) (Fig. S15, Table S24), following standard field protocols for microstratigraphic block sampling^227,230,237-242^. Blocks were taken from distinct fossiliferous breccia units in the south wall of Mafeng Cave, east wall of Shuangtan Cave, north wall of Hejiang Cave, north wall of Bapeng Cave (at the transition of a silty clay and breccia) and east wall of Queque Cave (Fig. S15). All breccia units are known to contain *G. blacki* materials, except that at Mafeng Cave (S24). Prior to sampling, the (macro)stratigraphy, sedimentary textures and structures, of each fossiliferous unit per selected cave wall, were studied with a hand lens (mag. 5×). The sediments were classified (following ^243,244^), their macrofacies were described, and their profiles were photographed and drawn (Fig. S15). Sediment colours were determined with a Munsell Soil Colour Chart^245^.

The anterior side of the blocks was plastered, leaving the posterior side open for additional bulk analyses (Fig. S16). The sediment blocks were carefully extracted intact from trench/wall profiles in order to retain the original relationships between the sediments and any included material. After transport from the field, sediment blocks were air-dried, soaked with a two-part polyester resin, and diluted with styrene monomer at the Flinders University Microarchaeology Laboratory. After the resin cured the blocks were cut into ‘wafers’ (70×50×10 mm), and labelled to retain orientation information. These wafers were sent to Adelaide Petrographics for thin section manufacture, producing two 30 µm sediment slices mounted on glass microscope slides (76 × 50 mm) for each site, with a total of 10 thin sections manufactured. The thin sections were scanned with on an Epson V600 flatbed film scanner to first assess the thin sections at the ‘meso-scale’Thin sections were manufactured from the blocks’ interior, and we analysed bulk disaggregated sediments from their posterior sides.

To enable microstratigraphic examination through petrographic microscopy, two thin sections were manufactured from each block – one from the upper, younger half (thin section ‘A’) and one from the lower, older half (thin section ‘B’). Block sample resination and thin section manufacture followed common lab protocols (*e.g.* ^230,231,241^). Blocks were air dried for 24 hrs at room temperature, soaked with large plastic containers filled with a two-part polyester resin (Polyplex clear ortho casting resin, Allnex) and diluted with styrene monomer (7:3 ratio) (Allnex). After the resin was partly cured, the blocks were removed from the container and placed in a drying oven (Labec) over 48 hrs at 40°C. After complete curing, the blocks were cut into 5 mm thick wafers by using a core saw (Barranca Diamond), with regards to their original top (field) position and lines indicated during the preparation. The wafers were sent to Adelaide Petrographics Pty Ltd. for thin sections manufactures. The thin sections were scanned with a flatbed scanner ^247^ and analysed using a Leica DM2700 P polarising microscope at magnifications 2.5×, 5× and 10×, and under plane and crossed polarised light (PPL and XPL) (Fig. S17a-e). A semi-quantitative microfeatures and –facies trait table was made (Table S24), based on the design in ^242,243^. The terminology used to describe the microfeatures followed the guidelines of ^56^. Microstratigraphic analysis was conducted at the Microarchaeology Laboratory, Flinders University.

PSA was performed on bulk samples from each block (Fig. S16). The samples were placed in an ultrasonic probe (Vibra-Cell, Sonics, USA) to break down sediment aggregates and isolate individual grains. Two grams of sediment from each sample were placed in a plastic 120 ml containers, filled up with deionised water and the suspension was gently agitated. Two runs were performed on each sample: a first run of 2:30 mins with an amplitude of 30% followed by a second run of 30 seconds with an amplitude of 50%. The pulser function was not used. Removal of organic matter followed the sodium hypochlorite method of ^248^, with the exception of HCl that was replaced by a 10% nitric acid solution (HNO_3_) (to adjust the alkalinity from buffered sodium hydrochlorite (NaClO) from ~12.5 to <9.5, measured by pH meter). Sodium hydroxide (NaOH) was not used. Each sample was carefully treated individually in a hot water bath (80°C) (TWB-24D, Thermoline Scientific) and centrifuge (1500 rpm) (GT-20, Spintron), as described in step 1 and 2 of ^248^. After the chemical preparation the five samples were split in four-to-six subsamples during multiple rounds of splitting, with a wet splitter, garden pressure hose (Hozelock 5L) and medium sized beakers. The samples (in suspension) were loaded in a wet dispersion unit (Hydro 2000G) of the Mastersizer 2000 (Malvern). The laser obscuration for loaded material per subsample was around 15%, the stirrer function at 500 rpm and pump function at 1700 rpm. The sample material was set at default and the dispersant was deionised water. Two cleaning cycles were performed between each subsample of each site and three cleaning cycles between the three different sites. Data visualisation and calculation of standard parameters were performed in Gradistat 9.1 ^249^. PSA took place in the Sediment Lab at Macquarie University.

Present day alkalinity (pH) values of bulk sediment per block (Fig. S16) were determined with a soil test indicator, colour developer and alkalinity colour chart with pH ranges 2–10 (Sørensen scale). Testing took place close to a window to facilitate natural light conditions. One lab spoon (Kartell) of flat stroked sediment per site was placed on a clean, white, plastic spot plate and covered with 15 droplets of liquid indicator solution. The substance was stirred to a thick dark paste. A thin layer of white barium sulphate (BaSO_4_) powder was sprinkled on the paste and each mixture was given 3 mins of development time. The experiment was repeated twice on each sample to rule out incorrect colour development that could have been caused by anthropogenic errors.

Alkalinity experiments were followed by XRD and XRF analyses on bulk (Fig. S16). To enable a better identification of the chemical components, the samples first had to be micronised to individual clay particles of micron (μm) scale, in a microniser mill (McCrone). A small amount of fresh untreated bulk from each sample (5 g) was dry grinded with a mortar and pestle and placed in a glass beaker. The spoon, mortar and pestle were cleaned with ethanol and paper towel between each sample. The beakers were filled up with ethanol (10 ml) to produce a ‘slurry’ required for wet grinding. Each slurry was poured in to a sample holder that was filled up with 8 piles of 6 stacked agate stubs (total 48) enabling wet grinding with ethanol in all directions. After milling, the fine-grained slurry was placed back in the original beaker that was cleaned with ethanol in between runs. The beakers were placed in a fume hood for 24 hrs, facilitating ethanol evaporation, followed by another 24 hr in a drying oven to get them completely dry for XRD and XRF. The micronized bulks were re-grind to powder with a pestle and mortar and placed in stubs.

This enabled XRD^234^ by a 23 mm fixed beam mask in an Aeris benchtop X-ray diffractometer (Malvern PANalytical), for 13 mins per sample, under diffraction angles from 3.5° to 90° and standard resolution. XRD data-analysis was performed in HighScore Plus (2020) and took place in the Instrument Lab at Macquarie University.

The same bulk powder samples were used for XRF ^235^, including loss on ignition (LOI) measurements at 1000°C ^250^. From each sample, two grams were placed in a glass vial tube. The vial tubes were placed in a drying oven (Heraeus, Kendro) for 3 hrs (105°C), to ensure all atmospheric water vapour that could have affected the moisture content of the samples between different analyses had left the powder. The tubes were then placed in a vacuum desiccator (Wheaton) filled with purple silica beads for four days, to absorb any remain moisture from the air. The samples were mixed with pre-dried x-ray flux (at 600°C) (XRF Scientific), to lower the melting point of the samples. One gram of sample per vial was added to 10 g of flux in a platinum crucible, and mixed with a spatula to a homogenous powder. Sample and flux weights were recorded and the scale was cleaned between every sample. The crucibles were placed in a rocking furnace, for 30 mins (1050°C) (Fusilux) and additionally one pellet of ammonium iodide (NH_4_I) wetting agent was added to each sample-flux mixture to make the substance less viscous. The liquid was placed back in the furnace (3 mins), after which it was poured into a platinum disc and cured to a coloured glass disc. This procedure was repeated for every individual sample. The five glass discs, each representing one site, were put aside to cool down. Recorded weights of each sample and flux were set in the sample changer software before the discs entered the Axios WD-XRF spectrometer (Malvern PANalytical). XRF-analysis took place at Macquarie University GeoAnalytical (MQGA).

**Fig S15**: Overview of sampled sediment blocks (top) and field (macro)stratigraphy logs (**bottom**) of the five sites selected in this study for microstratigraphic analysis. From left to right, in chronostratigraphic order from young to old: Mafeng Cave (CMF); Shuangtan Cave (CSHT); Hejiang Cave (CHEJ), Bapeng Cave (CBAP) and Queque Cave (CQQ). Corresponding ages are in Ma. Please note, the blocks are displayed from their non-plastered posterior view with dimensions indicated as R (right), L (left) and T (top).


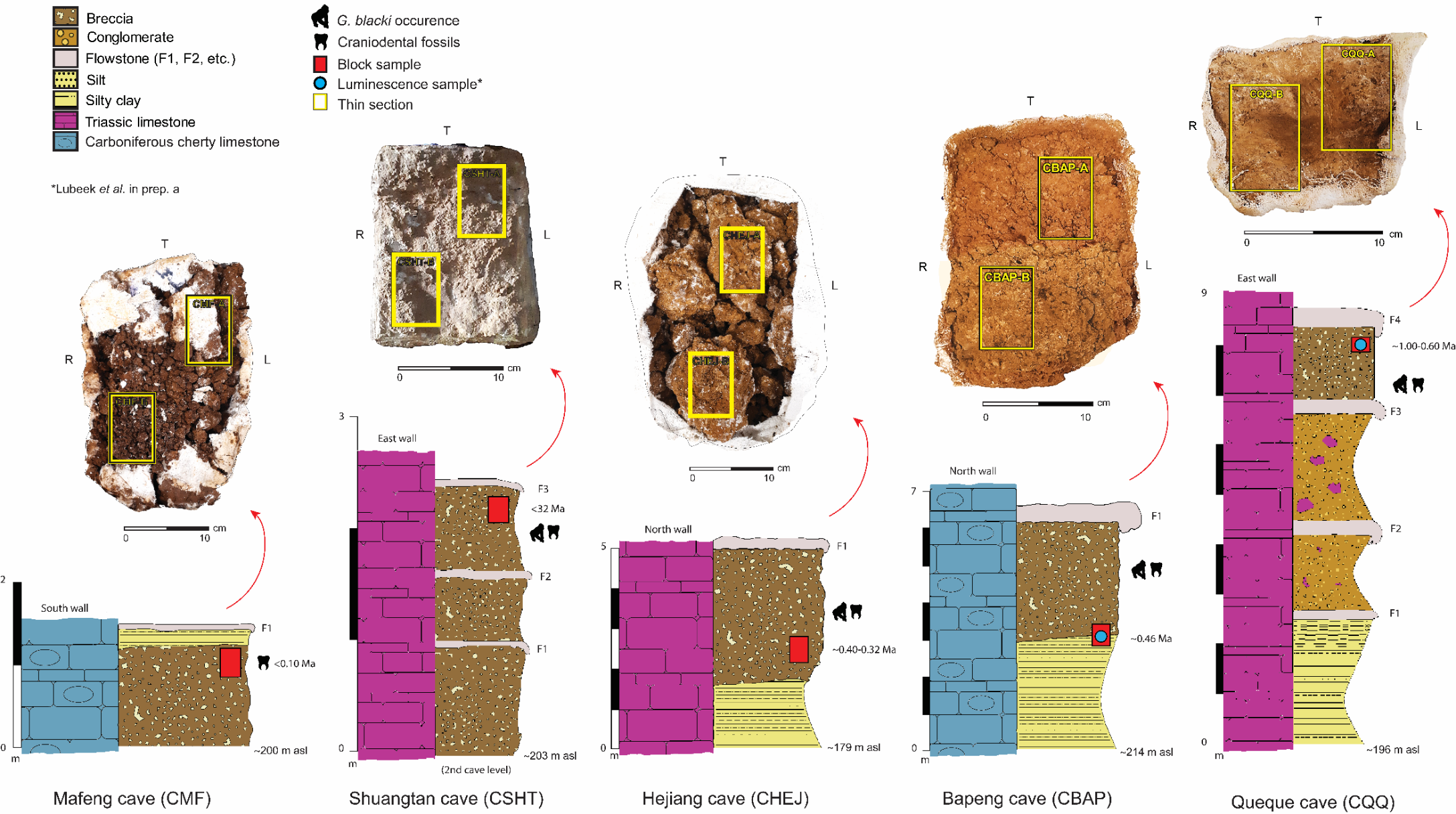


**Fig S16**: **a:** Schematic explanation of block sampling in a cave setting/from a cave wall sequence (from Shuangtan Cave). Step 1: carving out of the block at the best suitable sample location; Step 2: plastering of the anterior (A), left and right sides of the block up to ~3 cm towards its posterior side (P); Step 3: careful removing of the block with a hammer and/or field knife, under an angle of 45° by pulling and lifting. **b**: Overview of the techniques applied to different targeted areas within one block. These include: microstratigraphy (MS) of thin sections from the interior, and bulk-based particle size analysis (PSA), alkalinity (pH), x-ray diffraction (XRD) and x-ray fluorescence (XRF) from the non-plastered posterior side. The techniques are colour-coded per scientific discipline from which they are derived. Block dimensions are in cm.


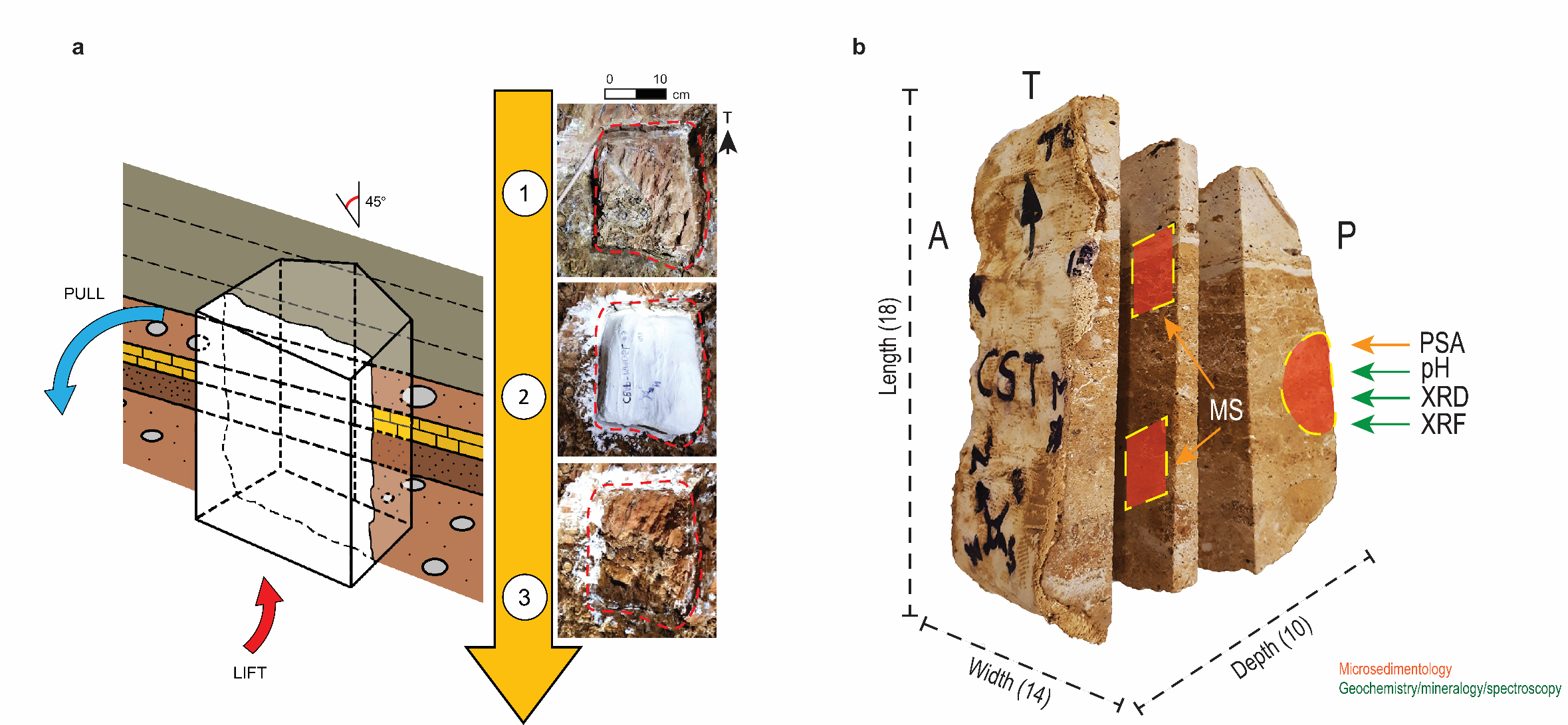


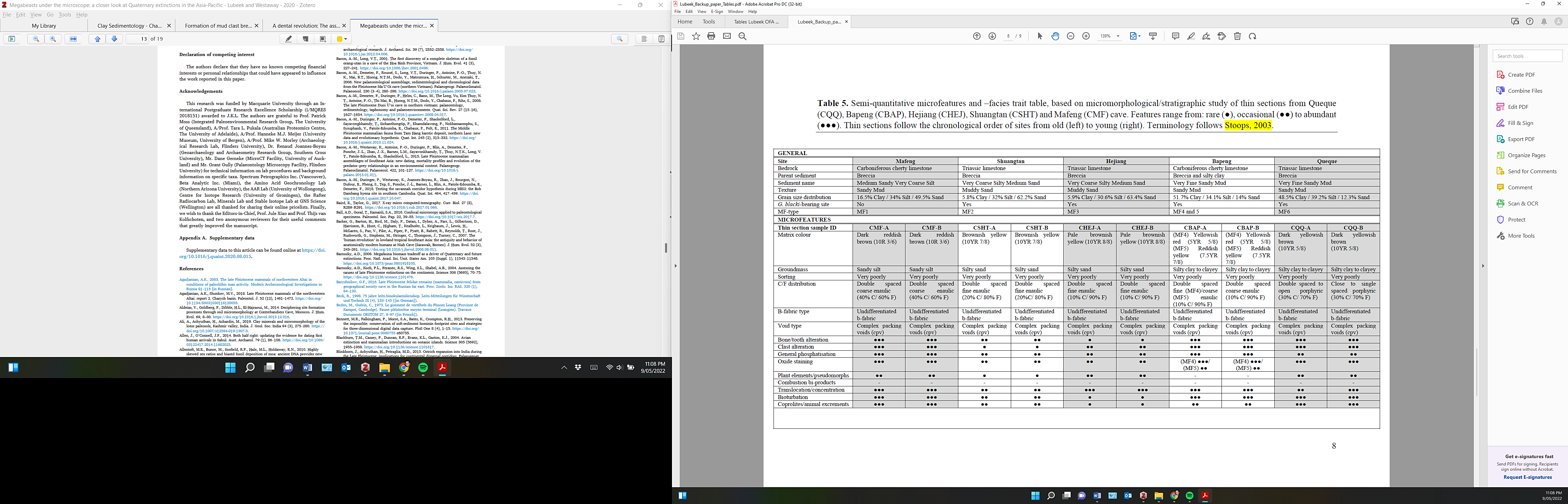
**Table S24:** Semi-quantitative microfeatures and –facies trait table, based on micromorphological/stratigraphic study of thin sections from: Mafeng (CMF), Shuangtan (CSHT), Hejiang (CHEJ), Bapeng (CBAP), and Queque (CQQ) Caves. Features range from: rare (●), occasional (●●) to abundant (●●●). Thin sections follow the chronological order of sites from young (left) to old (right). Terminology follows ^56^.

**Table S24.** Microfacies type (MF-type) descriptions continued.

| **MICROFACIES** | |
| --- | --- |
| **Type** | **Description** |
| MF1 | Dark reddish brown sandy silt groundmass with double spaced coarse enaulic C/F distribution in both CMF thin sections, that is very poorly sorted and comprises heterogenic grains. MF1 shows an undifferentiated b-fabric with complex packing voids. Most microfeatures in MF1 are abundantly observed. Plant elements/pseudomorphs occur occasionally, mostly as organic pigments, while combustion bi-products are absent. Bone/tooth alteration, oxide staining and clast alteration, as well as general phosphatisation are severe. MF1, the youngest facies type, shows most similarities with MF4-5 and MF6, the oldest facies types in this study. |
| MF2 | Brownish yellow silty sand groundmass with double spaced coarse enaulic C/F distribution in both CSHT thin sections. MF2 is very poorly sorted and comprises heterogenic grains in an undifferentiated b-fabric with complex packing voids. MF2 displays significant differences in microfeatures and their occurrence, compared to MF1. Bone/tooth alteration, clast alteration and general phosphatisation are less severe compared to MF1, resulting in a better preservation of bone/tooth and clast materials. Especially flowstone materials seem better preserved compared to other facies, clast alteration is therefore rare. MF2 is the only facies type in which (fresh water) shell fragments are recorded. Plant elements/pseudomorphs are rare and sporadically occur in the form of organic pigments. Combustion bi-products are absent, while translocation/concentration of microfeatures, bioturbation and coprolites/animal excrements occur occasionally. MF2 shows most overlap with MF3. |
| MF3 | Pale brownish yellow silty sand groundmass with double spaced coarse enaulic C/F distribution in both CHEJ thin sections. MF3 is very poorly sorted and comprises heterogenic grains in an undifferentiated b-fabric with complex packing voids. This MF-type holds less bone/tooth materials compared MF2 and therefore has a lower (rare) bone/tooth alteration, while the clast alteration seems more occasional. Most features are similar in occurrence to the CSHT thin sections. MF3 displays clear similarities with MF2, although plant elements/pseudomorphs and translocation/concentration of microfeatures seem more abundant in this facies. |
| MF4 | Yellowish red silty clay to clayey groundmass with double spaced fine enaulic C/F distribution in CBAP-A, and coarse enaulic C/F distribution in CBAP-B. Very poorly sorted with heterogenic grains observed in the coarse fraction. MF4 shows an isotropic/undifferentiated b-fabric with complex packing voids. Matrix colour and c/f distribution are the main micromorphological differences between MF4 and 5. Oxide staining occurs abundantly. Coprolites and other animal excrements occasionally occur, while plant elements/pseudomorphs and combustion bi-products are absent. Alteration of bones/teeth, clasts and general phosphatisation are abundant. |
| MF5 | Reddish yellow silty clay to clayey groundmass with double spaced coarse enaulic C/F distribution in both CBAP thin sections. Very poorly sorted with heterogenic grains observed in the coarse fraction. MF3 shows an isotropic/undifferentiated b-fabric with complex packing voids. Matrix colour and c/f distribution are the main micromorphological differences between MF4 and 5. Oxide staining is occasionally observed. Coprolites and other animal excrements occasionally occur, while plant elements/pseudomorphs and combustion bi-products are absent. Alteration of bones/teeth, clasts and general phosphatisation are abundant. MF4 and 5 differ significantly from MF2 and 3. |
| MF6 | Dark yellowish brown silty clay to clayey groundmass with double spaced to open porphyric C/F distribution in CQQ-A and close to single spaced porphyric in CQQ-B. Very poorly sorted with a more concentrated heterogenic coarse grained structure in CQQ-A and evenly heterogenic in CQQ-B. MF6 shows an isotropic/undifferentiated b-fabric with complex packing voids. Translocation/concentration of bioturbated microfeatures is occasional in CQQ-A and abundant in CQQ-B. Combustion bi-products are absent, while oxide staining and coprolites/animal excrements are abundant. Clast alteration and oxide staining are abundant, while plant elements/pseudomorphs occur occasionally. MF6 shows most similarities with MF4-5 and MF1. |

**Results**

The field stratigraphy is shown in Fig S15. For the purposes of the current paper emphasis is placed upon the microstratigraphic content. Further details and interpretations of field stratigraphy from overlapping (Bapeng and Queque Cave) and associated sites (Mafeng Cave) in the region will be addressed separately in another publication. No differences in pH were measured between the five sites. All block samples contain highly alkaline sediments (pH = 9) as might be expected in a limestone karst environment. However, we emphasise that these pH values reflect the present-day environment, and these were likely to have differed in the past. Microstratigraphy, PSA, XRD and XRF results (the latter three datasets are shown in Fig S18) could resolve this uncertainty.

*Microstratigraphy results*

*Micro Facies 1 (Mafeng Cave, CMF)*

The youngest of the five sites, Mafeng Cave (CMF) sediments form Micro Facies (a package of sediments associated with a specific depositional and/or post-depositional environment; MF) 1 (Table S24, Fig S17a). MF1 comprises a dark reddish brown (10R 3/6)) sandy silt groundmass with a double spaced coarse enaulic coarse/fine (C/F) particle size distribution in both CMF thin sections. The coarse fraction consists of grains of polygenetic origin, *e.g.* calcite, kaolinite, and the sulfides cryptohalite, melanite, hematite and birnessite (Fig S17a CMF). The PSA results (GradiStat 9.1, Blott, 2020; Table S24) show that the sediment comprises predominantly of sand (49.5%), and can be termed a ‘Medium Sandy Very Coarse Silt’. The sediment is very poorly sorted, and classified as a ‘Sandy Mud’ in texture. The particle size histogram (Fig S18, CMF) shows a polymodal distribution with five peaks, of which one clear peak in clay, two modest ‘peaks’ in the silt population, one modest peak at the transition of silt to sand and a clear peak in sand. Both CMF thin sections (Fig. S17a) show an undifferentiated b-fabric in which the compounds are separated by complex packing voids. There are no noticeable differences in features between CMF-A, the younger (top) thin section, and the older (bottom) CMF-B thin section.

Most microfeatures that constritute MF1 are observed in abundance (Table S24). Evidence for heterogenic grains suggestive of polygenetic origin are exemplified by a lighter coloured, coarse-grained and heterogeneous composition, which may be part of the original/older matrix (Fig. S17a A, G). These lenses occur in between a dominant clay altered matrix of variously-coloured, large clay compound aggregate grains with dark striae separated by calcite filled voids and larger clasts (Fig. S17a D). Features consistent with deposition by gravity and traces of bioturbation and coprolites/animal excrements are abundant, including burrows with altered, phosphatised rock and clay infillings (Fig. S17a B), guano, reworked clay and calcite remnants (Fig. S17a C), and flowstone or speleothem grains (Fig. S16a F). Plant elements/pseudomorphs occur occasionally, mostly as organic pigments in sand lenses (Fig. S17b G), while combustion bi-products are absent. Bone and tooth alteration features, oxide staining and clast alteration, as well as general phosphatisation are abundant and severe, suggesting chemical diagenesis. This is illustrated by a bioeroded bone fragment (Fig. S17a E) and diagenetically altered clay features (Fig. S17a H). Mafeng Cave shows relatively high concentrations of silicon dioxide (SiO_2_), aluminium oxide (Al_2_O_3_) and slightly lower concentrations in calcium oxide (CaO), titanium dioxide (TiO_2_) and iron(III) oxide (Fe_2_O_3_). The LOI (at 1000°C) is also high (Fig. S18, CMF). MF1 is consistent with a locally-derived colluvial deposit that has been subject to some minor and locally significant chemical alteration.

**Fig. S17a-e**: Thin section micrographs from: **a** Mafeng Cave (CMF); **b** Shuangtan Cave (CSHT); **c** Hejiang Cave (CHEJ); **d** Bapeng Cave (CBAP); and **e** Queque Cave (CQQ). **a** Flatbed scans (**left**) and selection of micrographs from MF1 in the Mafeng Cave (CMF) thin sections (CMF-A and B) (**right**). To test for reproducibility, we used the point-counting method to assess the relative abundance of a particular feature and tested there was consistency between point-counts. Features are only significant if abundance counts of >10% are recorded across the slide **A**: Lighter coloured more heterogenic grained (possibly original) matrix (2.5×, PPL); **B**: Burrow with altered, phosphatised rock and clay infilling (2.5×, XPL); **C**: Guano-induced (arrow) matrix of reworked clay and calcite remnants (2.5×, PPL ); **D**: Large clay clast in contact with voids in clay altered matrix (2.5×, XPL); **E**: Bioeroded bone fragment (red contour line) (10×, PPL); **F**: Flowstone or speleothem intrusion (2.5×, XPL); **G**: Original sandy-silty matrix with few clay aggregates and possibly organic pigments. An alteration due to manufacture also visible and should not be confused with a microfeature (red contour line) (2.5×, PPL); **H**: Lighter coloured sandy-silty banded remnant feature with (Fe, Ti, and/or Al) oxide stains in clayish matrix (2.5×, PPL).

**a CMF***
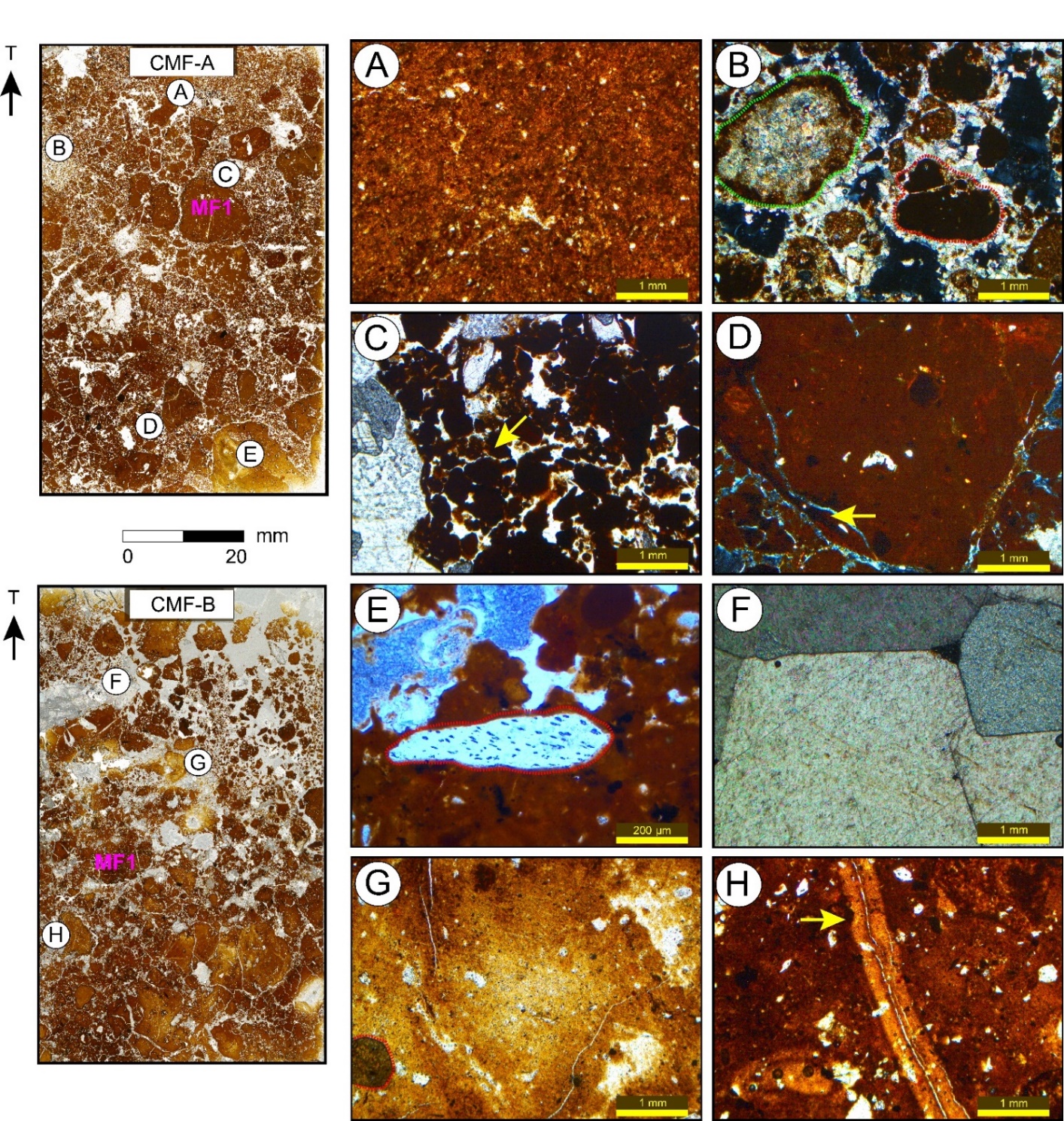
*

**b CSHT -** Flatbed scans (**left**) and selection of micrographs from MF2 in the Shuangtan Cave (CSHT) thin sections (CSHT-A and B) (**right**). **A**: Contact of the matrix with flowstone (at the top) and a clay clast (at the bottom). The arrow indicates a close-up (2.5×, PPL); **B:** Close-up of early coarse crystalline flowstone growth (red contour line) between the finer-coarsed flowstone at the top, and clay clast at the bottom (5×, PPL); **C:** Phosphatised bone fragment (red contour line) with clay coating (arrow) (2.5×, PPL). **D:** Phosphatised bone fragment with calcite inclusion (arrows) (2.5×, PPL and XPL); **E and** **F**: Bioeroded bone fragments (arrow and red contour) (5×, PPL and XPL **G**: Close-up of phosphatised bone fragment with oxide stains at the rims (10×, PPL) **H**: Phosphatised flowstone with calcite cap (green contour line and arrow) (2.5×, PPL); **I**: Possibly a gastropod shell in cross-section (arrow and red contour line) (10×, PPL); **J**: Possibly a shell fragment in longitudinal section (red contour line). Notice the banded colourings (arrow) (10×, PPL and XPL).


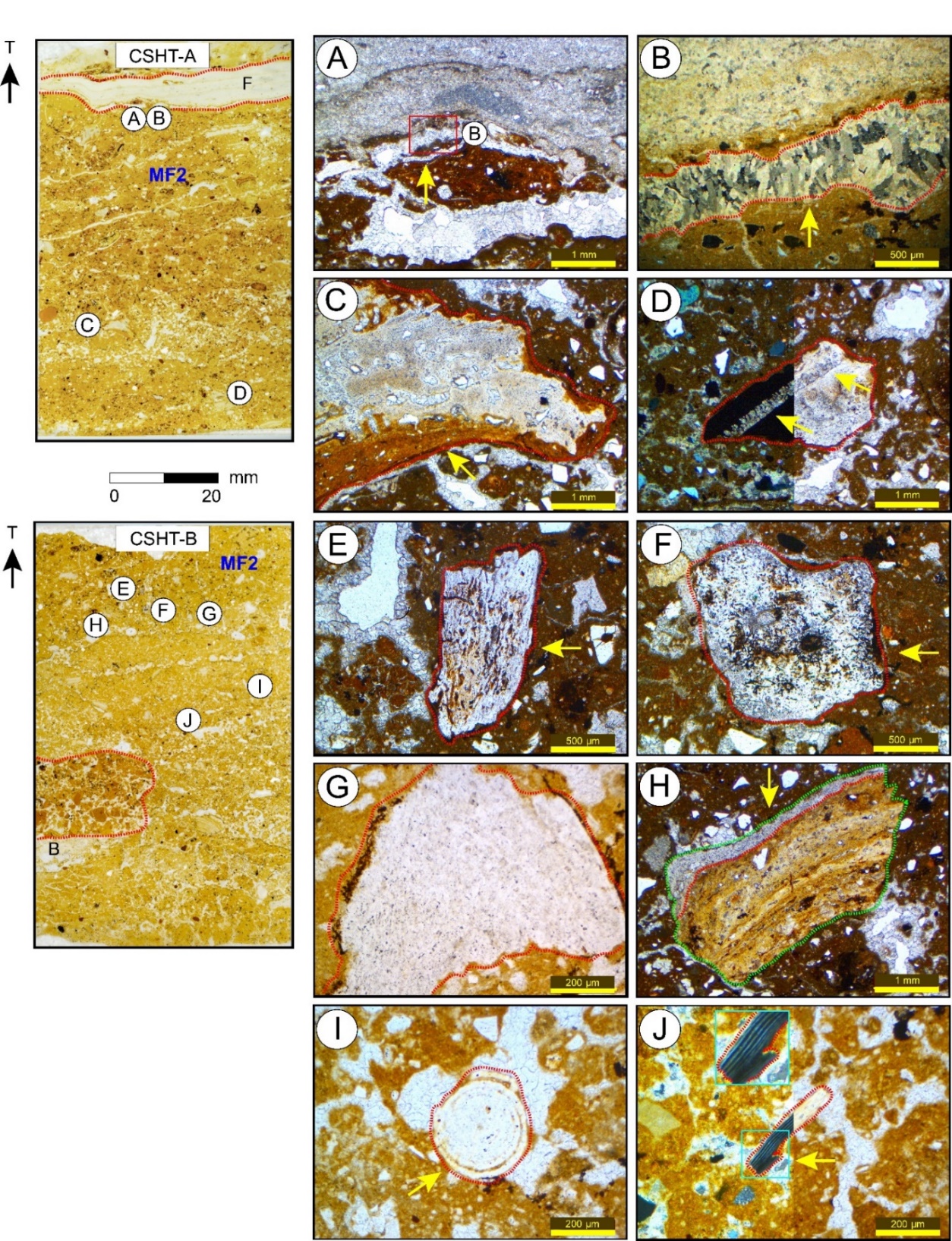


**c CHEJ** Flatbed scans (**left**) and selection of micrographs from MF3 in the Hejiang Cave (CHEJ) thin sections (CHEJ-A and B) (**right**). **A:** Metamorphic rock remnants (red contour lines) in clay dominated matrix (2.5×, PPL); **B**: Phosphatised bone fragment with organic pigments and oxide stains (arrow and red contour line) (2.5×, PPL and XPL). **C**: Fine sandy to silty matrix with organic pigmentation, oxide staining and calcite infilled veins (arrow) (2.5×, PPL). **D**: Mosaic of rock remnants, clay, and calcite infilled veins (2.5×, XPL); **E**: Remnant of original sandy-silty matrix with reworked clay, calcite infilled voids, oxide stains and organic pigments (2.5×, PPL). **F**: Reworked clay with oxide stains and organic pigments at the left and centre. Rock remnant on the right, with calcite infilled voids in between (2.5×, PPL).


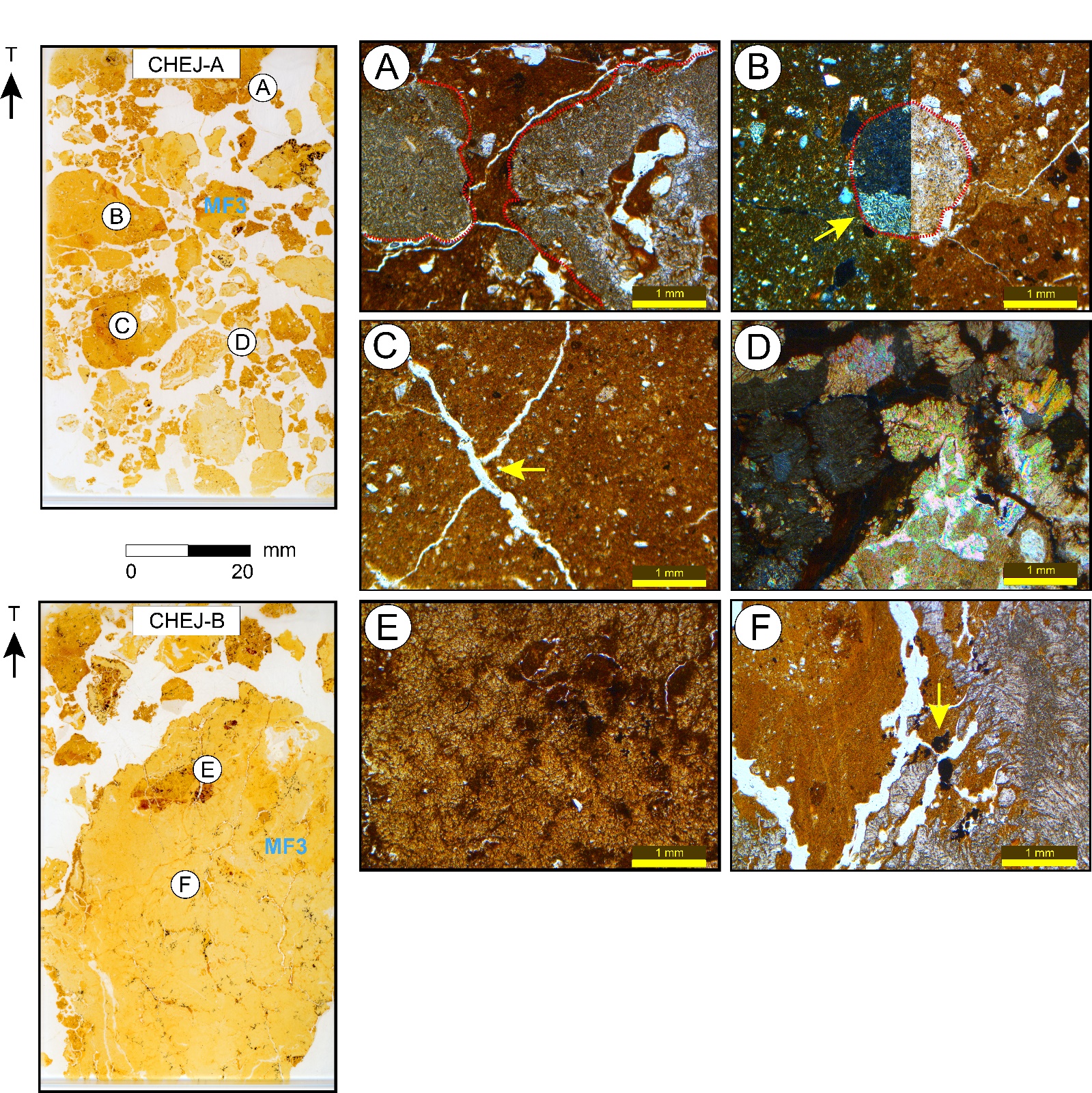


**d CBAP -** Flatbed scans (**left**) and selection of micrographs from MF4 and 5 in the Bapeng Cave (CBAP) thin sections (CBAP-A and B) (**right**). **A**: Relative sharp boundary transition of MF5 (left) and MF4 (right) groundmass. Both facies are heavily interbedded throughout the thin sections (5×, PPL); **B**: Gradual boundary transition (yellow contour line) through a void between MF5 (top) and MF4 (bottom) (2.5×, PPL); **C**: Oxide staining surrounding a void filled with calcite in a clay clast dominated matrix (10×, XPL); **D**: Burrow with altered clay clasts (arrow) and calcite filled voids mixed with guano ( 2.5×, PPL); **E**: Dark striae of different oxides (arrow) in a clay clast dominated matrix (2.5×, XPL); **F**: Typical MF5 groundmass and matrix with calcite and clay aggregates (red contour line), and iron oxide and/or titanium dioxide nodules (green contour line). Possibly some organic (botanic) pigments as well (arrows) (2.5×, PPL); **G**: Typical MF4 groundmass and matrix with lots of oxide staining and voids with calcite infillings (2.5×, PPL); **H**: Gradual transition boundary between MF4 (top) and MF5 (bottom) with channelling voids in between filled up with calcite (2.5×, PPL).


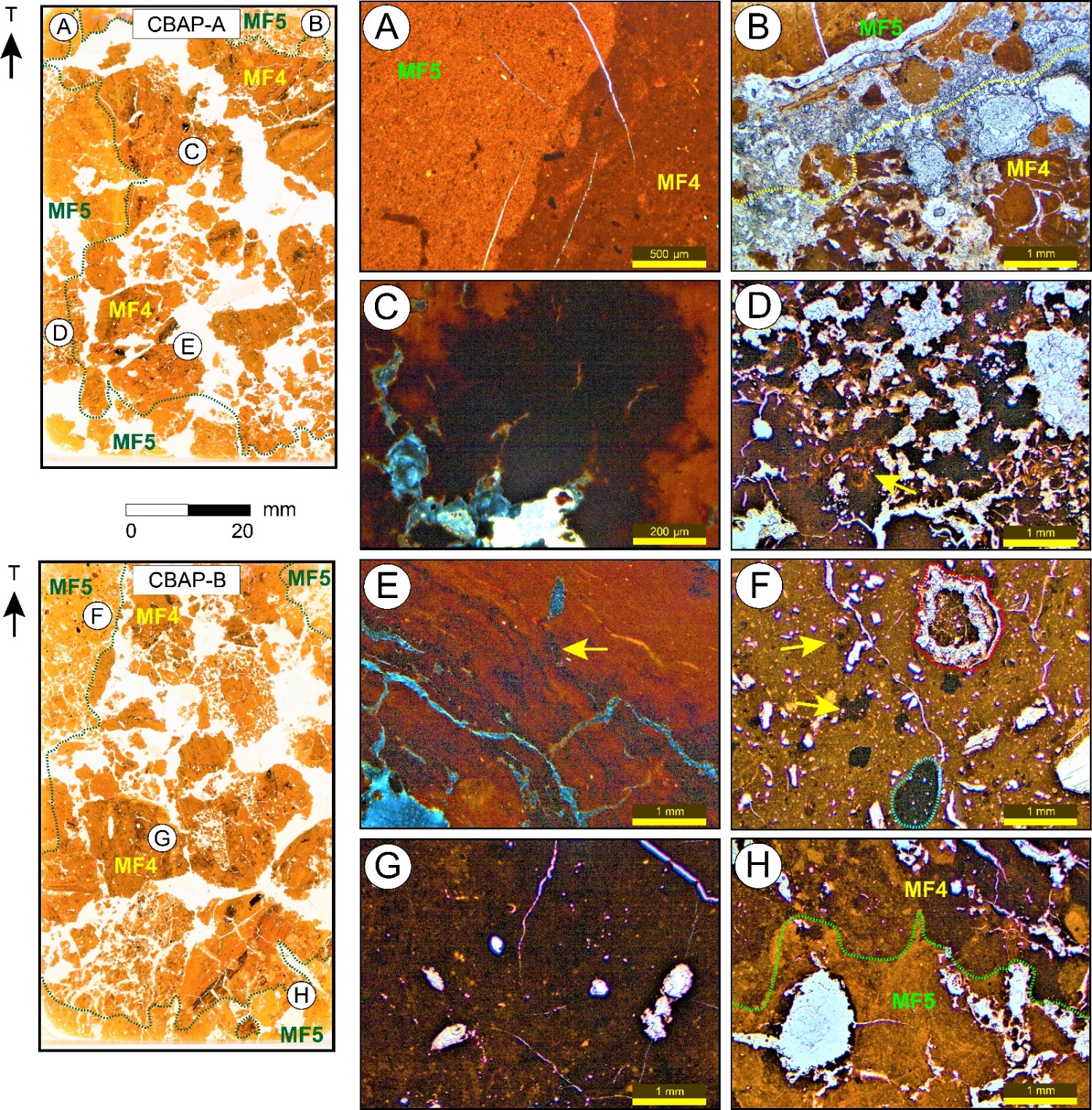


**e CQQ -** Flatbed scans (**left**) and selection of micrographs from the Queque Cave (CQQ) thin sections (CQQ-A and B) (**right**). **A**: Weathered coprolite (2.5×, PPL); **B**: Pelloidal structure of microfaunal excrements (arrow) (2.5×, PPL); **C**: Weathered rock fragment (possibly schist or quartzite with calcite infillings) (2.5×, XPL); **D**: Neoformed calcite (calcite replaced by phosphates) (arrow and red contour line) (2.5×, XPL); **E**: Phosphatised speleothem fragment (2.5×, PPL); **F**: Phosphatised bone fragment (red contour line) (2.5×, PPL); **G**: Close-up of phosphatised bone fragment with a fine layer of clay (arrow) on the transition with the matrix (10×, PPL); **H**: Burrow (red contour line) with accumulation of clay, guano, coprolites, organic (botanical) materials and phosphatised bone (2.5×, PPL); **I**: Phosphatic nodule (red contour line) with banded remnants of clay (arrow), flowstone and rock fragments (2.5×, XPL); **J**: Oxidised iron replaced organic matter (red contour line), likely botanic (5×, PPL).


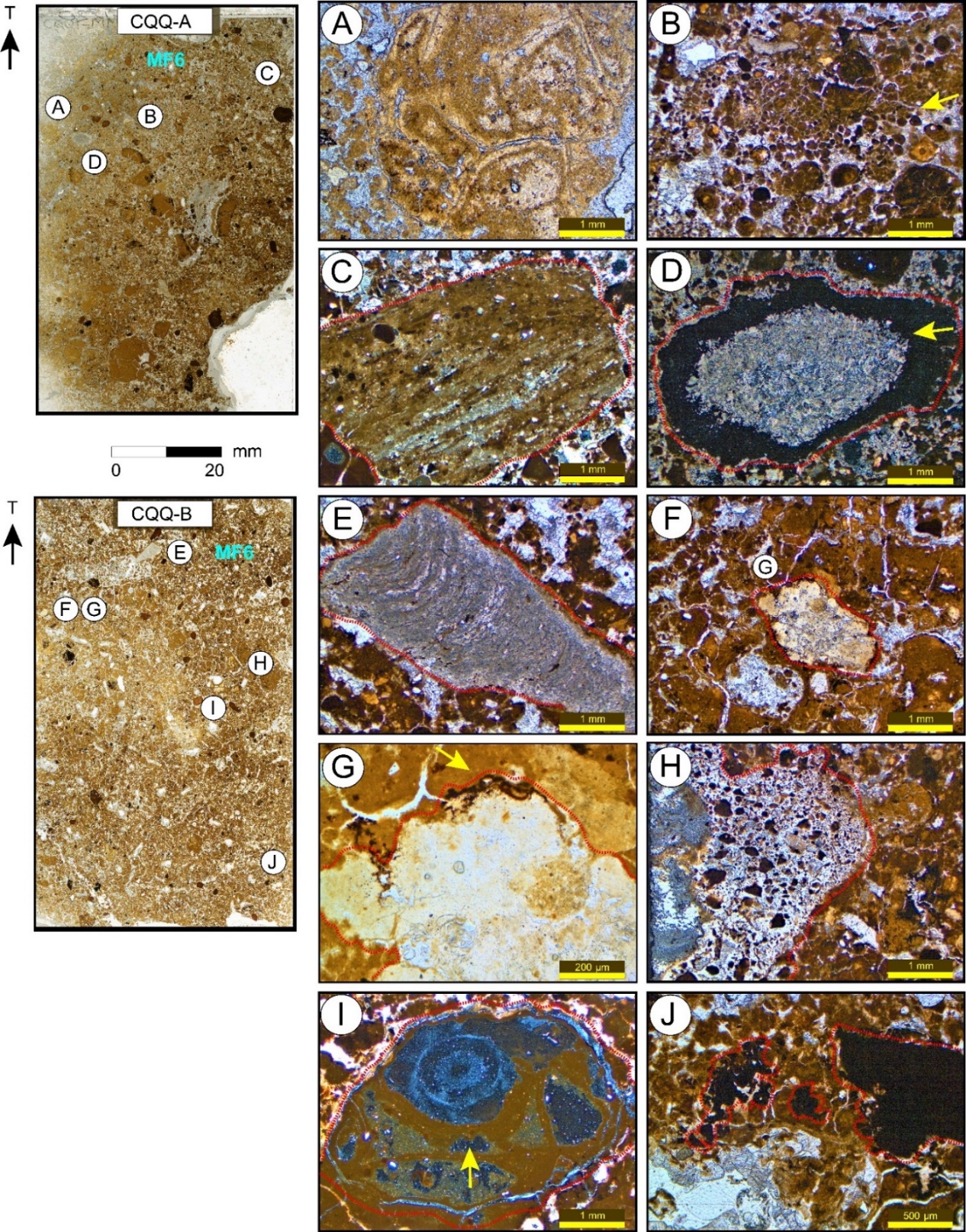


**Fig S18**. Results of PSA, XRD, and x-ray fluorescence XRF (on bulk) (from left to right) per site. From top to bottom (from young to old): Mafeng Cave (CMF), Shuangtan Cave (CSHT), Hejiang Cave (CHEJ), Bapeng Cave (CBAP) and Queque Cave (CQQ). In the PSA histograms, the respective size fractions and corresponding populations are plotted in different shades for clarity (GradiStat 9.1). The grain size boundaries are defined as: clay (0.01–2 μm), silt (2–62.5 μm) and sand (62.5–2000 μm), following the Wentworth scale^249^. XRD data is summarised as relative abundances of minerals per site based on semi-quantitative weights in % (HighScore Plus 2020). Raw data intensity (2-theta) plots can be found in the SI. Major element compounds as detected by XRF (bulk) include: silicon dioxide (SiO_2_), aluminium oxide (Al_2_O_3_), calcium oxide (CaO), iron(III) oxide (Fe_2_O_3_), phosphorus pentoxide (P_2_O_5_), titanium dioxide(TiO_2_) and sulphur trioxide (SO_3_). Minor elements are grouped together and consist of compounds including: vanadium oxide (V_2_O_5_), chromium(III) oxide (Cr_2_O_3_), and Cobalt (II) oxide (CoO). XRF elements are plotted in weight % (Axios WD-XRF Software).


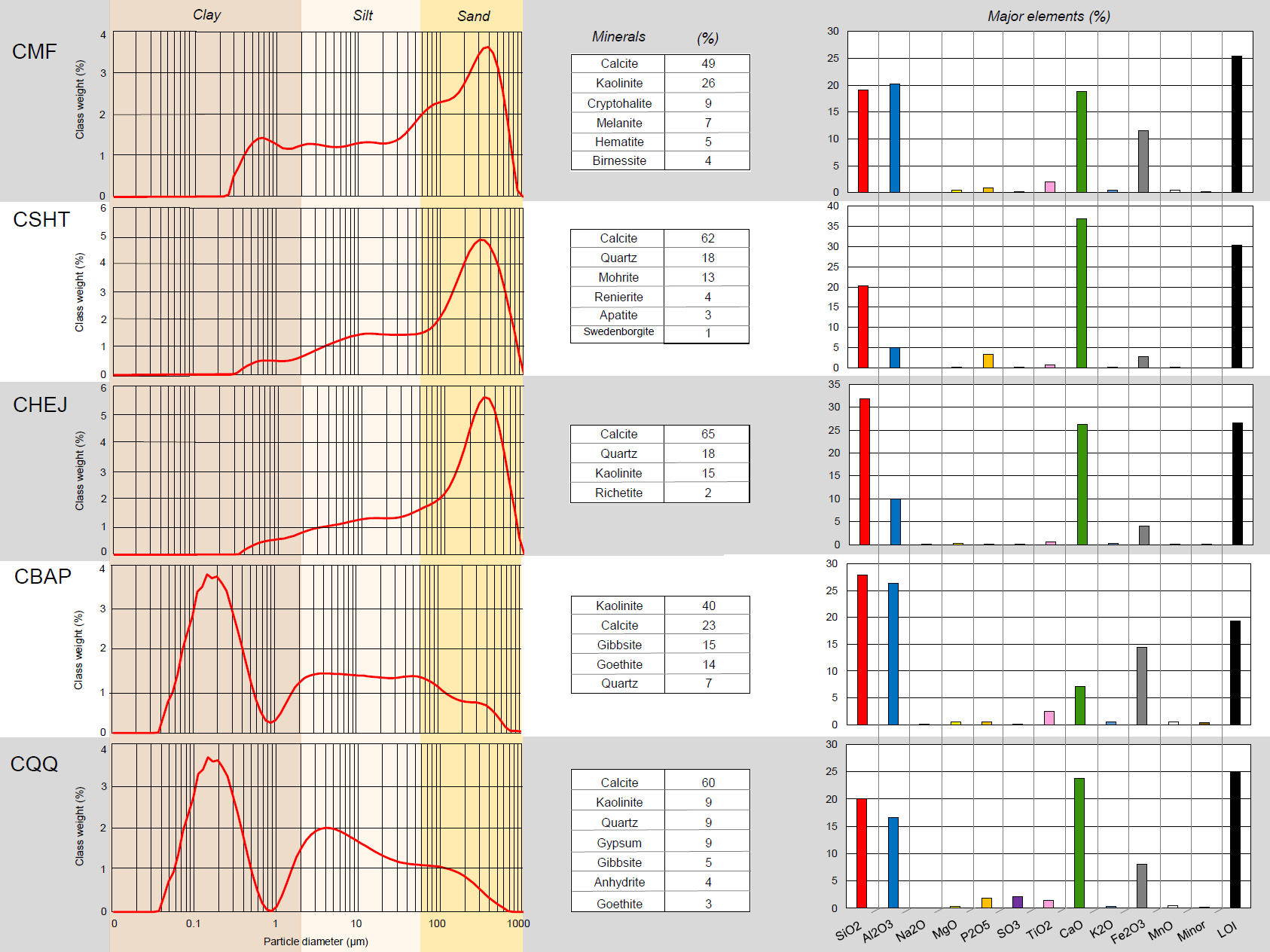


*Micro Facies 2 (Shuangtan Cave, CHST)*

The sediments from Shuangtan Cave (CSHT) can be classified as Micro Facies 2 (MF2). MF2 displays a brownish yellow, silty sand groundmass with double spaced coarse enaulic C/F distribution. The coarse fraction comprises a diverse range of mineral and compound grains of polygenetic origin, including calcite, quartz and apatite, but also some more exotic minerals (*e.g.* renierite and swedenborgite) (Fig. S18, CSHT). The PSA results (GradiStat 9.1, Blott, 2020; Table S24) show that the sediment comprises 62.2% sand and can termed a ‘very coarse silty medium sand’. The sediments are very poorly sorted, and classified as a ‘Muddy Sand’ in texture. The particle size histogram (Fig. S18, CSHT) shows a trimodal distribution, with modest peaks in clay and silt, and a very clear third sand peak. The y-axis for Shuangtan and Hejiang cave is extended to a class weight of 6%, while for the other sites, the maximum class weight value is 4%. Both CSHT thin sections exhibit an undifferentiated b-fabric with complex packing voids. There are no obvious facies differences between the upper, younger (CSHT-A) and lower, older (CSHT) thin section, other than a slightly less porous structure in the upper region (CSHT-A).

Evidence of chemical diagenesis such as bone, tooth and clast alteration and signs of phosphatisation are less severe than recorded in MF1. This means that there is better preservation states of fossils in the sediments from this site. In this respect, the Shuangtan Cave sediments are somewhat similar to the post-cranial-fossil-bearing sediments recorded at Xiaokou Cave, also located in the Chongzuo region. Speleothem fragments are better preserved than in other facies, exemplified by a large flowstone fragment that dissects CSHT-A (Fig. S17b A-B), and a partly-phosphatised flowstone with calcite cap (Fig. S17b H). The contact between the overlying flowstone and the clay clast beneath in Fig. S17b A-B illustrates early coarse, crystalline flowstone growth. Numerous phosphatised bone fragments with clay coatings (Fig. S17b C), neoformed calcite (Fig. S17bD) and oxide staining around the rims (Fig. S17b G) are present, in addition to bioeroded bone fragments (Fig. S17b E-F). Combustion bi-products are absent, while microfeatures diagnostic of bioturbation and coprolites occur only occasionally. Plant elements and plant pseudomorphs are rare, occurring sporadically in the form of organic pigments. MF2 is the only facies-type that contains traces of mollusc, most likely gastropod, and multiple freshwater shells (Fig. S17b I-J). The high frequency of bone is confirmed by a high levels of apatite (Ca5(PO4)3(Cl/F/OH)) shown in the XRD-plots and phosphorus pentoxide (P2O5) in the XRF-plots (Fig. S18, CSHT). LOI (at 1000°C), silicon dioxide (SiO2) and calcium oxide (CaO) are high at this site, while iron(III) oxide (Fe2O3), titanium dioxide (TiO2) are low, and aluminium oxide (Al2O3). In fact, the latter compound is lowest at this site (Fig. S18, CSHT).

*Micro Facies 3 (Hejiang Cave, CHEJ)*

Micro Facies 3 (MF3) is recorded in Hejiang Cave (CHEJ) thin sections CHEJ-A and B (Fig. S17c), comprising a pale brownish yellow, silty sand groundmass with double spaced coarse enaulic C/F distribution. The coarse fraction comprises a limited range of heterogenic grains of polygenetic origin, including calcite, quartz, kaolinite and richetite (Fig S22, CHEJ). The PSA results (GradiStat 9.1, Blott, 2020; Table S23) indicate that the sediment comprises 63.4% sand, and is a ‘very coarse silty medium sand’. The sediments are very poorly sorted, classified as a ‘muddy sand’ in texture. The particle size histogram (Fig S18, CHEJ) shows a bimodal distribution, with a modest peak in silt and a very clear second peak in sand. Similar to CSHT, the y-axis for CHEJ is extended to a class weight of 6%. Both CHEJ thin sections show an undifferentiated b-fabric with complex packing voids. There are no obvious facies differences between the upper (CHEJ-A) and lower (CHEJ-B) thin sections.

Most MF3 microfeatures are similar to those recorded in the CSHT thin sections. However, MF3 contains less bone and tooth fossils compared to MF2. Characteristic of this facies are the metamorphic rock remnants suspended in a clay-dominated matrix (Fig. S17c A) and phosphatised bone fragment with organic pigments and oxide stains (Fig. S17c B). The metamorphic rock fragments frequently occur as a colourful mosaic (in XPL) of rocks, clay, flowstone fragments and calcite infilled veins (Fig. S17c D). The clay frequently exhibits oxide staining and organic pigments, and is mostly separated from the rocks remnant through calcite infilled voids (Fig. S17c F).

Fine sandy lenses can be observed with clay remnants, organic pigments, oxide staining and calcite infilled veins (Fig. S17c, E). Plant elements/pseudomorphs and translocation/concentration of microfeatures seem slightly more abundant in this facies compared to CSHT. XRF-peaks (Fig S18, CHEJ) in iron (III) oxide (Fe2O3), aluminium oxide (Al2O3) and titanium dioxide (TiO2) are lower compared to most other sites. Similar to CSHT, the silicon dioxide (SiO2), calcium oxide (CaO) and LOI (at 1000°C) concentrations at Hejiang Cave are high.

*Micro Facies 4 & 5 (Bapeng Cave, CBAP)*

The sediment block from Bapeng Cave (CBAP) was sampled across the interface of two adjacent layers, capturing the transition between two different microfacies, MF4 and 5 (Table S24, S17d). Field observations showed MF4 primarily at the top of the sample and MF5 at the base. However, at the micro-scale the facies are notably interstratified. MF4 is a yellowish red (5YR 5/8) silty clay with clayey groundmass and double-spaced fine, enaulic C/F distribution (CBAP-A), and a coarse enaulic C/F distribution in CBAP-B. MF5 consists of a reddish yellow (7.5YR 7/8) silty clay to clayey groundmass with double spaced coarse enaulic C/F distribution. Matrix colour and c/f distribution are the main micromorphological features differentiating the two facies. The matrix colour (primarily influenced by presence of oxides) is indicative of the different geochemical compositions of MF4 and 5 (Fig. S17d, CBAP).

The coarse fraction of sediments from CBAP consist of a mix of different grains (*e.g.* quartz Fig. S17d, CBAP). The sediment can be classed a ‘very fine sandy mud’, very poorly sorted, and classified as a ‘sandy mud’ in texture (GradiStat 9.1, Blott, 2020; Table S24). The thin sections are the most clay-dominated of those analysed, with an average clay content of 51.7%. The particle size data (Fig. S17d, CBAP) reveal a polymodal distribution with four peaks, although with modest amplitudes. One clear peak, similar to the data from other sites examined, is observed in clay, two relatively small ‘peaks’ are apparent in the silt population, and a fourth subtle peak in the sand fraction. Both of the thin sections show an isotropic/undifferentiated b-fabric in which particles are separated by complex packing voids. Although the microfacies are heavily interbedded, they can be easily distinguished due to sharp boundaries in the groundmass types (Fig. S17d A) and by the arrangement of voids (Fig. S17d B, H).

Oxide staining occurs in abundance in MF4, while only occasionally observed in MF5. Oxide staining in MF4 is typified by staining surrounding calcite-filled voids (Fig. S17d C) and in the form of dark striae (Fig. S17d E). In both microfacies, translocation/concentration or movement of materials into and out of sediments^56^ often occurs in the form of burrows with altered clay clasts and calcite filled voids mixed with guano (Fig. S17d D). Photomicrographs F and G show details of groundmass and matrix of the two microfacies recorded at this site (Fig. S17d F-G). In photomicrograph G, the groundmass in MF4 shows frequent oxide staining and calcite-filled voids (Fig. S17d G), while micrograph F shows the typical MF5 groundmass with calcite and clay aggregates, organic pigmentation, iron oxide and/or titanium dioxide nodules (Fig. S17d F). Coprolites occur occasionally throughout sediments in both thin sections. Plant remains and combustion bi-products are absent. Alteration features of bones, teeth, and clasts are abundant. Bapeng Cave is the site that is richest in clay minerals such as kaolinite (Al_2_(OH)_4_Si_2_O_5_), gibbsite (Al(OH)_3_) and goethite (α-FeO(OH)) (Fig. S17d, CBAP). This is consistent with the peak in iron(III) oxide (Fe_2_O_3_), and peaks in silicon dioxide (SiO_2_), aluminium oxide (Al_2_O_3_) and titanium dioxide (TiO_2_) (Fig. S17d, CBAP). On the contrary, Bapeng Cave contains sediments that are the least calcareous.

*Micro Facies 6 (Queque Cave, CQQ)*

Analysis of thin sections from Queque Cave reveal sediments that form MF6 (Table S23, Fig. S17e). MF6 comprises a dark yellowish brown (10YR 5/8) clayey groundmass with double spaced to open porphyric C/F distribution (thin section CQQ-A) and close to single spaced porphyric C/F distribution (CQQ-B). Similar to MF4-5 at Bapeng Cave, the CQQ sediment is a ‘very fine sandy mud’, very poorly sorted, and classified as a sandy mud in texture (GradiStat 9.1, Blott, 2020; Table S24). The particle size histogram (Fig. S17e, CQQ) reveal a trimodal distribution with a peak in clay, a second peak in silt, and a third subtle peak in sand. This grain size distribution is very similar to that of Bapeng Cave, but where Bapeng has slightly higher amounts of clay and sand, Queque has a slightly higher silt distribution (Fig. S17e, CQQ, Table S24). Both of the CQQ thin sections show an isotropic/undifferentiated b-fabric in which the components are separated by complex packing voids.

Thin section CQQ-A, at the top of the block, contains a heterogenic coarse-grained structure and is clay-rich towards the base (*e.g.* kaolinite, goethite, gibbsite). CQQ-B, at the base, is uniformly heterogeneous throughout the thin section and there are more void spaces. Bioturbation microfeatures are sparse in CQQ-A and abundant in CQQ-B. in the form of burrows with accumulations of clay, guano, coprolites, plant materials and phosphatised bone (Fig. S17e H). Combustion bi-products are absent and weathered coprolites are abundant in both thin sections (Fig. S17e A). Pelloidal structures with microfaunal excrements are also evident (Fig. S17e B). Clast alteration and oxide staining is abundant, often in the form of rock fragments (possibly schist or quartzite with calcite infillings) (Fig. S17e C), neoformed calcite (calcite replaced by phosphates) (Fig. S17e D), phosphatised speleothem fragments (Fig. S17e E), and phosphatic nodules with banded remnants of clay, flowstone and rock fragments (Fig. S17e I). Chemical diagenesis occurs to a lesser degree at Queque Cave compared to Bapeng Cave, which is reflected by the sporadic but moderately preserved occurrence of phosphatised bone fragments (Fig. S17e F-G). MF4 shows the highest relative occurrence of plant elements/pseudomorphs, which are only occasionally present throughout the CQQ thin-sections, mostly as oxidised iron replacement pseudomorphs of plant matter (Fig. S17e J). Queque Cave exhibits the highest diversity in minerals, including kaolinite (Al_2_(OH)_4_Si_2_O_5_), gibbsite (Al(OH)_3_) and goethite (α- FeO(OH)), carbonates such as calcite (CaCO_3_), as well as the sulfates anhydrite (CaSO_4_) and gypsum (CaSO_4_·2H_2_O) (Fig. S17e, CQQ). As such, the CQQ sediments show the highest concentration in sulfur trioxide (SO_3_) of all sites. Phosphorus pentoxide (P2O5), titanium dioxide (TiO_2_) and LOI (at 1000°C) are also relatively high, while iron (III) oxide (Fe_2_O_3_) is relatively low.

**Discussion: sedimentary signals for changes in depositional environment**

Mafeng Cave contains the youngest facies type, MF1. MF1 shows affinities with MF4 in particular, and MF5 to a lesser extent, both from the (middle) Middle Pleistocene site, Bapeng Cave. It also shares similarities with MF6 from the Early Pleistocene site, Queque Cave. These are the oldest facies types in this study. MF2 and 3, found at the late Middle Pleistocene sites, Shuangtan Cave and Hejiang Caves, are similar to each other but different to the other microfacies.

With respect to the pH conditions at the sites, the chemical environment at Mafeng, Bapeng and Queque Caves may have been more acidic due to the presence of titanium dioxide (TiO_2_), and to a lesser extent sulfur trioxide SO3, the former of which is unfavourable for post-cranial-preservation. Evidence for chemical diagenesis (e.g. phosphatisation) is more common at these sites compared to Shuangtan and Hejiang caves. At Bapeng Cave in particular, phosphate and calcium oxide concentrations decrease, while iron oxides increase, yet the pH of these sites remain the same. This may indicate that past pH values were lowest at Bapeng Cave, with the potential to dissolve bones and calcium carbonate.

The lesser degree of bone and tooth alteration and phosphatisation at Shuangtan and Hejiang Caves may relate to pH, which could have been more alkaline at the time of deposition and thereafter. The pH of water in contact with silicates and carbonates generally increases with the duration of contact with rock, which would support this^251^. This may have had a buffering effect to create a more alkaline environment favourable for the preservation of bone (*e.g.* Fig. S17d C-F), even for those with signs of microbial bioerosion. Microbes, in fact, may also have played a role in the reduced level of phosphatisation^252^ at Shuangtan and Hejiang Caves, as microbes may have been feeding on the soft tissues associated with the post-cranials in a wet cave during sediment deposition. Manganese and iron are being used by micro-organisms in these types of environments, which could explain the low abundances of these compounds at these sites^253-255^. However, the higher amount of oxides at Mafeng, Bapeng and Queque, suggested by abundant oxide staining in the sediment and mineralogical compound peaks in goethite, gibbsite, gypsum and anhydrite, are consistent with a redoximorphic environment with extended wetting-dryng events resulting in weathering and oxide Mn and Fe production.

The four microfacies recorded at Mafeng, Bapeng and Queque Caves are broadly indicative of stable, humid, and low energy depositional conditions. The high occurrence of the clay minerals kaolinite, goethite and gibbsite at the oldest two sites, Bapeng and Queque Cave, may not only indicate drier depositional conditions but also a warmer environment. This aligns with conditions recorded at the Early Pleistocene site of Mohui Cave (Bubing Basin)^159^. Although Mafeng Cave shows a high kaolinite content, it does not have the high diversity of clay minerals as recorded at Queque and Bapeng Caves. Therefore, the depositional conditions at Mafeng Cave may have been slightly cooler and/or wetter compared to Bapeng and Queque Caves.

The presence of guano can be linked to specific environmental conditions and the occurrence of specific mineral assemblages (*e.g.* ^256-258^). When water passes through guano it becomes rich in phosphate, lowering pH and creating a more acidic environment. It is possible that reacting with calcite (or dolomite) the solution can become more alkaline, which is beneficial for stabilisation of carbonates and the preservation of fossils^259^. While coprolites and oxide staining are abundant features at the Mafeng, Bapeng and Queque Caves, these only occur sporadically at Shuangtan and Hejiang cave. Guano-producing animals, such as bats in particular, prefer stable and dry habitats which suggests that drier conditions enabled the bat populations to increase with greater guano deposition to the Mafeng, Bapeng and particular Queque Cave sediments. Guano deposits would have provided new sources of guano acidic leachates that could interact with minerals, resulting a higher mineral diversity^256,259,260^. This scenario could support the diverse range in minerals present at Mafeng and Queque Caves. In addition, these sites both show the highest abundance of sulfate minerals (*e.g.* anhydrite, gypsum, cryptohalite, melanite, hematite and birnessite) which may indicate the most redox-reduced^261^ and therefore most stable depositional conditions in terms of temperature and humidity.

All sediment profiles from which blocks were sampled are capped by flowstones (Fig. S15), but our mineralogical and geochemical data show that the Shuangtan, Hejiang and Queque Cave sediments contain the highest concentrations of calcite (CaCO3), visible in various flowstone remnants under the polarising microscope (Fig. S17d A-B, H; Fig. S18D; Fig. S19 D-E). The growth of flowstones requires laminar surface4 water flow, indicating wet conditions. The indurated matrix of the Shuangtan and Queque blocks (Fig. S15), could be linked to the higher number of flowstones at these sites, from which calcium carbonate-charged groundwater percolated into the microfacies. However, while wetter conditions at Shuangtan Cave may have been caused by greater hydrological input over a shorter period of time (resulting in thinner flowstones), those at Queque Cave may have had an extended and slower time-averaged growth pattern over a longer period of consistently wet conditions, following longer periods of drier conditions. During these drier events, calcite precipitation rates may have been slower, and therefore flowstone formation less prevalent. Queque Cave is significantly older than Shuangtan Cave and contains much thicker flowstones (Fig. S15). Queque Cave may therefore have been drier compared to those shorter, higher energy conditions at Shuangtan (and Hejiang) Cave. The presence of shell fragments (Fig. S17b I-J) at Shuangtan Cave may also indicate an influx of fresh water or continuous groundwater supply.

Our data suggests rapid and relatively high energy fluvial sedimentation and/or local breakdown as the main drivers of deposition of MF2 and 3 at Shuangtan and Hejiang cave respectively. The greater quantities of calcite and apatite, and carbonate and phosphate minerals in general, at Shuangtan and Hejiang Cave, may support a higher energy environment dominated by coarser sandier sediments, as these larger grain sizes require a higher flow velocity to mobilise. Although remnants of most likely older and low-energy clay deposits are recorded in MF2 and 3 (e.g. Fig. S17 C-E). MF1 at Mafeng Cave (despite some sandy lenses), MF4-5 at Bapeng Cave, and MF6 at Queque Cave, probably formed predominantly by slower accumulation of finer silts and clays, rather than sands, over longer time-periods with similar, stable conditions resulting in ephemeral ponding during wetter periods causing the settling out of fine sediments such as clays and fine silts, or erosion during desiccation. Autochthonous sedimentation^262^ of fine percolating sediments from the epikarst above the main cave, and/or accumulation through ephemeral ponding during colluviation^56^ seems more likely at Mafeng, Bapeng and Queque Caves. In fact, when plotting the textural group of each sediment per site in a sediment ternary plot (Fig. S19), we can see this PSA pattern. There seems to exist a ‘textural group shift’: from fine grained sandy clays at Queque and Bapeng Caves, before the extinction period – to coarser grained silty sands at Shuangtan and Hejiang Caves during the extinction period – and back to finer grained (intermediate) sandy silts at Mafeng Cave. The smaller sized grain textures may indicate more stable, less energetic conditions.

**Fig S19**: Grain size data plotted on a ternary diagram for the five sites, with textural groups plotted per site (circles) and age range (colour). Sites include (from young to old): Mafeng Cave (CMF); Shuangtan Cave (CSHT); Hejiang Cave (CHEJ), Bapeng Cave (CBAP) and Queque Cave (CQQ). There is a ‘textural group shift’ through time visible: from fine grained Sandy Muds at Queque and Bapeng Caves, before the extinction period – to coarser grained Silty Sands at Shuangtan and Hejiang Caves during the extinction period – and back to finer grained (intermediate) Sandy Silts at Mafeng Cave. The smaller sized grain textures may indicate more stable, less energetic conditions. Data derived from PSA in GradiStat 9.1.


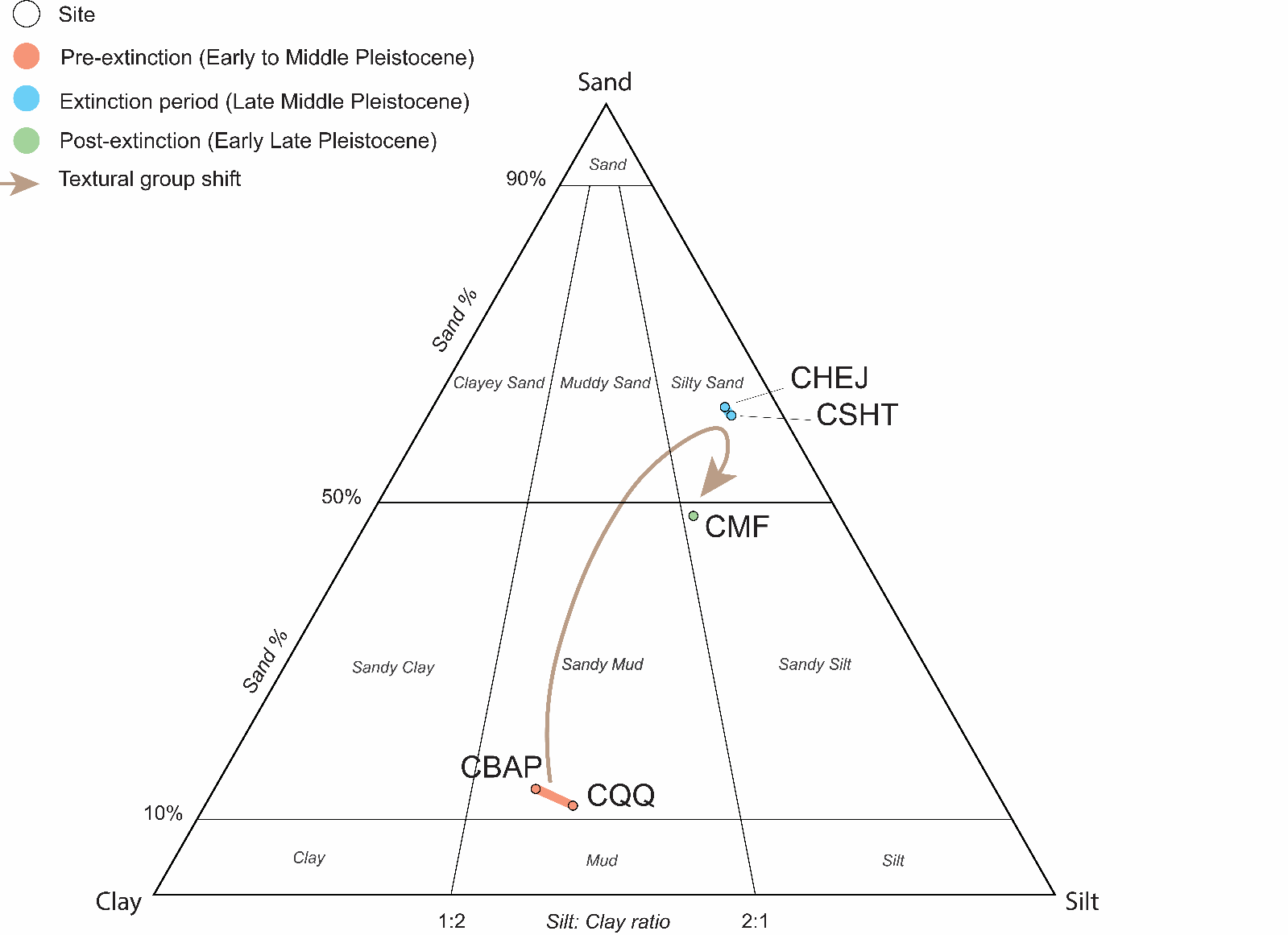


**SI section 12: Stable isotope analysis of teeth**

**Methods**

A total of 27 teeth (15 fossil *G. blacki* and 7 fossil *P. weidenreichi* teeth) were selected from six *G. blacki* bearing caves (Queque, Chuifeng, Bapeng, Hejiang, Shuangtan and Yanliang) alongside 5 modern *Pongo* teeth from Perth Zoo and Utpina. Sampled teeth were cleaned using an air abrasion system to remove any adhering external material. Enamel powder for bulk analysis was obtained using gentle abrasion with a diamond-tipped drill along the full length of the buccal surface to ensure a representative measurement for the entire period of enamel formation. All enamel powder was pretreated to remove organic or secondary carbonate contaminates, following established protocols applied in the region (see ^22,61^). This consisted of a series of washes in 1.5% sodium hypochlorite for 60 min, followed by 3 rinses in purified H_2_O and centrifuging, before 0.1 M acetic acid was added for 10 min, followed by another 3 rinses in purified H_2_O (as per refs. ^263,264^). When comparing the newly acquired data presented here with those from the existing literature, it is worth noting that different pretreatment protocols have been applied in each case—although, for tooth enamel, pretreatment-induced variation is limited (<0.5‰ for δ^13^C and δ^18^O)^265,266^ and these differences have a negligible effect at the scale of the questions examined here^267^.

Following reaction with 100% phosphoric acid, gases evolved from the samples were analysed for their stable carbon and oxygen isotopic measurements using a Thermo Gas Bench 2 connected to a Thermo Delta V Advantage Mass Spectrometer at the Max Planck Institute for Geoanthropology (formerly for the Science of Human History). δ^13^C and δ^18^O values were compared against International Standards (IAEA-603 (δ^13^C = 2.5‰; δ^18^O = −2.4‰); IAEA-CO-8 (δ^13^C = −5.8‰; δ^18^O = −22.7‰); USGS44 (δ^13^C = −42.2‰)) and an in-house standard (MERCK (δ^13^C = −41.3‰; δ^18^O = −14.4‰)). Replicate analysis of MERCK standards suggests that machine measurement error is about ±0.1‰ for δ^13^C values and ±0.2‰ for δ^18^O values. Overall measurement precision was studied through the measurement of repeat extracts from a bovid tooth enamel standard (*n* = 30, ±0.2‰ for both δ^13^C and δ^18^O values).

For the Zoo specimens, we applied a δ^13^C offset of 1.5‰ to account for the Suess effect (see also ^22^).

**Results**

The δ^13^C and δ^18^O results from fossil and modern tooth enamel can be found in ED4, Figure S20 and Table S25. For the pre-EW period the δ^13^C and δ^18^O of *Gigantopithecus* range between –16.2 to –13.8‰ (Median = -15.4‰) and –9.7 to –7.0‰ (Median = -8.2‰), respectively. For the later EW period, this changes to –15.3 to –10.3‰ (Median = -15.0) and –9.3 to –6.3‰ (Median = -7.6) for δ^13^C and δ^18^O, respectively. In the case of *P. weidenreichi*, the pre-EW period δ^13^C and δ^18^O ranges are –14.7 to –13.7‰ (Median = -14.5‰) and –7.1 to 6.3‰ (Median = -6.7‰), changing to –14.7 to –13.3‰ (Median = -14.5‰) and –4.9 and –4.4‰ (Median = -4.8‰) in the ‘Late’ period. Modern Pongo has a δ^13^C and δ^18^O range of –12.5 to –11.0 (Median = -12.9) and –5.5 to –2.7 (Median = -2.9‰), respectively.

**Fig. S20**: Scatterplot of δ^13^C and δ^18^O data for fossil *Gigantopithecus blacki* and fossil and modern *Pongo*.

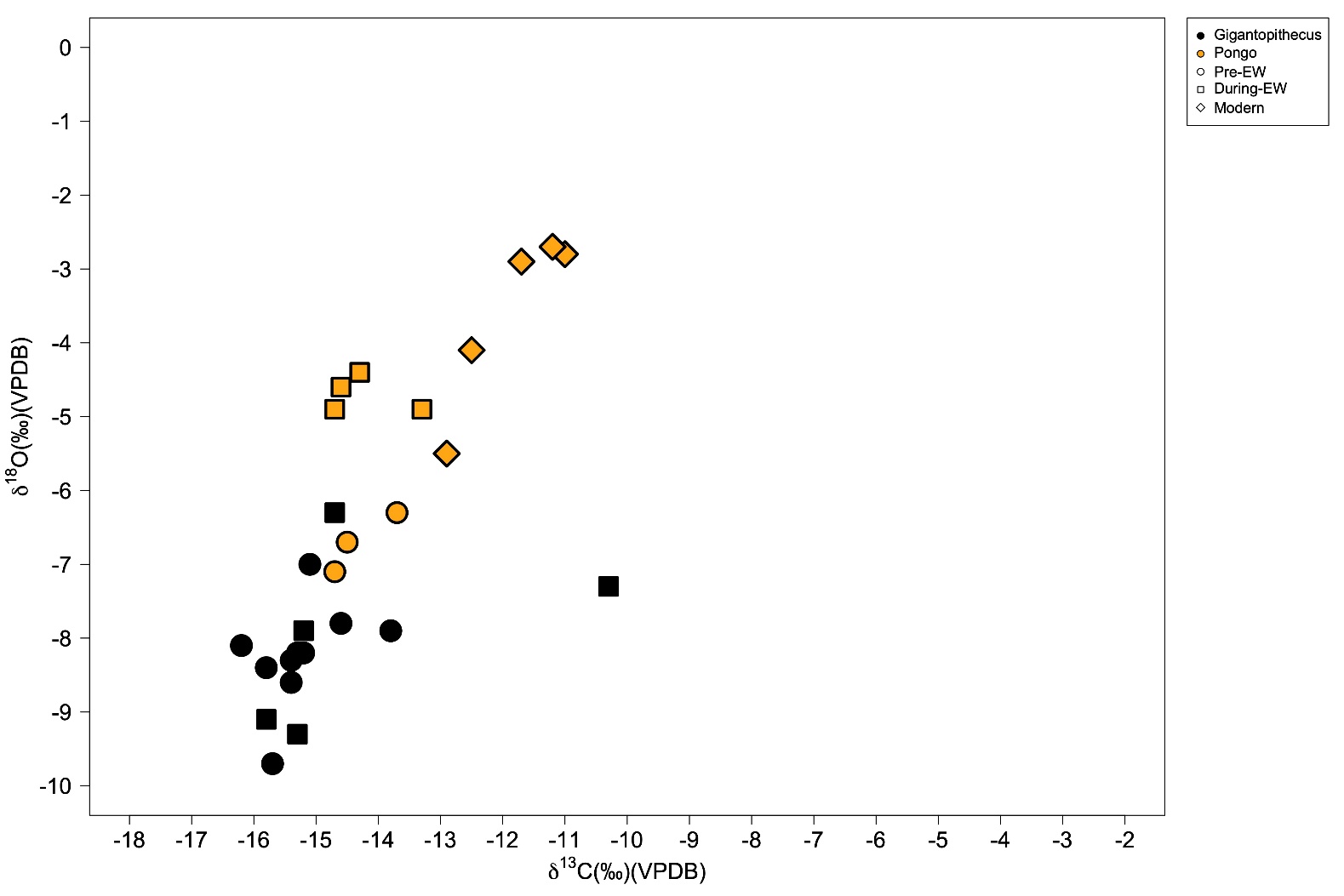


**Discussion**

There is significant isotopic fractionation of ^13^C during photosynthesis, the magnitude of which is shaped by the photosynthetic pathway used by the plant^268,269^. On average, the depletion relative to atmospheric δ^13^CO_2_ (approximately –6.5‰ before AD 1930) is –5‰ for C_4_ plants and –19‰ for C_3_ plants, respectively, leading to distinctive, non-overlapping values. This distinction has been used in tropical settings to explore the relative proportion of tropical grassland (often dominated by C_4_ plants) to tropical woodland or forest (dominated by C_3_ plants) in mammalian diets, and, therefore, indirectly across prevailing landscapes^268-274^. Within C_3_-dominated forest habitats, low light levels and trapped respired CO_2_ lead to further depletion of ^13^C in soils, leaves and fruits—and therefore mammals—in subcanopy environments, something which has become known as the ‘canopy effect’^275,276^ and has been observed among temperate, subtropical and tropical forests^276-282^. For the pre-industrial period, tooth enamel with δ^13^C lower than -14‰ represents reliance on dense or closed canopy forest and *c.* -12‰ and *c.* 0‰ are considered representative of herbivores with 100% C_3_ and C_4_ reliance, respectively^283^.

δ^18^O values in vegetation and animals can be studied to provide further insights into tropical palaeoenvironments. The key site of isotope fractionation in plants is the leaf, with evaporation (namely evapotranspiration) leading to the loss of lighter ^16^O and enrichment in ^18^O in the plant water that remains (ref. [^47^](https://www.nature.com/articles/s41586-020-2810-y#ref-CR47)). The degree of δ^18^O enrichment in leaf water is consequently negatively correlated with relative humidity, with greater humidity resulting in decreased δ^18^O values and vice versa^284-286^. As a result of differences in evaporative potentials across canopy strata and between different plant parts growing at different heights, CO_2_ and vegetation δ^18^O will vary along avertical transect^287-289^. Within tropical environments, the δ^18^O values of vegetation, are tracked especially closely in mammals that obtain most of their water from plants, providing information relating to evaporative potential or the source effect of rainfall as well as the vertical structure of forests^,287-293^. Significantly, folivores or frugivores that feed in the upper canopy will have higher δ^18^O values than animals feeding in the subcanopy^294^.

Our isotope data indicate that for the pre-EW period, both *G. blacki* and *P. weidenreichi* have δ^13^C values indicative of closed canopy forest resource reliance. Both primate groups also broadly overlap in terms of δ^18^O, albeit with Pongo falling towards the higher end of the range, suggesting similar hydrological conditions or canopy niche exploitation. Median values do, however, show Pongo to have a higher δ^13^C, suggestive of either slightly more open habitat feeding or a focus on fruit consumption^295,296^, as is known for orang-utans today. The overall δ^13^C and δ^18^O differences between the primate groups is broadly maintained into the EW period in the medians, although one *Gigantopithecus* individual has a much higher δ^13^C value of –10.3‰ which implies exploitation of more open woodland habitats. There is, however, a shift in Pongo δ^18^O which could imply the use of more seasonal or open areas of the forest (either higher in the canopy or in more open patches). This could perhaps indicate that Pongo populations were more willing to exploit new niches available under more arid, seasonal conditions while, with the exception of one individual, *Gigantopithecus* remained more focused to the same canopy forest niche. This supports the results of existing studies^22,297^ which, while identifying some variability in *Gigantopithecus* diets over time, seem to indicate that it remained more ‘forest-focused’ than its close *P. weidenreichi* relatives.

**Table S25**: δ^13^C and δ^18^O data for fossil *Gigantopithecus blacki* and fossil and modern Pongo. N.B. raw data is presented for the modern samples but the modern δ^13^C values have been corrected by 1.5‰ in Fig S20 and in the summary statistics discussion.

| **Sample** | **Site** | **Taxon** | **Tooth** | **ID** | **Age/period** | δ^13^C (‰) (VPDB) | **SD** | δ^18^O (‰) (VPDB) | **SD** | **Phase** |
| --- | --- | --- | --- | --- | --- | --- | --- | --- | --- | --- |
| GIG1 | Queque | *G. blacki* | Rp3 | CSQSN-41 | 918-615 | -13.8 | 0.1 | -7.9 | 0.1 | Pre- EW |
| GIG2 | Queque | *G. blacki* | Rp3 | CSQSN-31 | 918-615 | -15.4 | 0.1 | -8.6 | 0.1 |
| GIG3 | Queque | *G. blacki* | Lm3 | CSQ0811-5 | 918-615 | -15.8 | 0.1 | -8.4 | 0.1 |
| GIG4 | Queque | *G. blacki* | LM1/2 | CSQ0704-280 | 918-615 | -16.2 | 0.1 | -8.1 | 0.1 |
| GIG5 | Queque | *G. blacki* | RM3 | CSQSN-30 | 918-615 | -15.1 | 0.1 | -7.0 | 0.1 |
| GIG6 | Chuifeng | *G. blacki* | Rm3 | CF1 | 2.0-1.4 | -14.6 | 0.1 | -7.8 | 0.1 |
| GIG7 | Chuifeng | *G. blacki* | Lm2 | CF2 | 2.0-1.5 | -15.4 | 0.1 | -8.3 | 0.1 |
| GIG8 | Chuifeng | *G. blacki* | Lm2 | CF3 | 2.0-1.6 | -15.3 | 0.1 | -8.2 | 0.1 |
| GIG9 | Chuifeng | *G. blacki* | Lm1 | CF4 | 2.0-1.7 | -15.2 | 0.1 | -8.2 | 0.1 |
| GIG10 | Yanliang | *G. blacki* | Lm1 | PA1602.19 | 704-312 | -15.7 | 0.1 | -9.7 | 0.0 |
| GIG11 | Bapang | *G. blacki* | Rm3 | BALM3-1 | 354-310 | -15.8 | 0.1 | -9.1 | 0.0 |
| GIG17 | Queque | *P. weidenreichi* | Rm2 | CSQ0811-4 | 918-615 | -13.7 | 0.1 | -6.3 | 0.1 |  |
| GIG18 | Queque | *P. weidenreichi* | RM2 | CSQSN-35 | 918-615 | -14.7 | 0.1 | -7.1 | 0.1 |  |
| GIG19 | Queque | *P. weidenreichi* | Rm2 | CSQ0704-278 | 918-615 | -14.5 | 0.1 | -6.7 | 0.1 |  |
| GIG12 | Perth Zoo | *P. weidenreichi* | I1 | - | Modern zoo | -12.9 | 0.1 | -5.5 | 0.1 | Modern |
| GIG13 | Utpina | *P. weidenreichi* | m1 | - | Modern | -14.0 | 0.1 | -4.1 | 0.1 |
| GIG14 | Utpina | *P. weidenreichi* | m1 | - | Modern | -12.5 | 0.1 | -2.8 | 0.1 |
| GIG15 | Utpina | *P. weidenreichi* | m2 | - | Modern | -12.7 | 0.1 | -2.7 | 0.1 |
| GIG16 | Utpina | *P. weidenreichi* | m3 | - | Modern | -13.2 | 0.2 | -2.9 | 0.1 |
| 1573.8 Hejiang | Hejiang | *G. blacki* | left M1 | CLMH0904-93 (PA1573.8) | 307-274 | -15.3 | 0.1 | -9.3 | 0.1 | During- EW |
| 1573.9 Hejiang | Hejiang | *G. blacki* | left p3 | CLMH0904-89 (PA1573.9) | 307-274 | -15.2 | 0.1 | -7.9 | 0.1 |
| 50 Shuangtan | Shuangtan | *G. blacki* | upper molar fragment | CMLST0911-50 | 307-273 | -14.7 | 0.1 | -6.3 | 0.1 |
| 115 Shuangtan | Shuangtan | *G. blacki* | p4 | CMLST0911-109 | 307-273 | -10.3 | 0.1 | -7.3 | 0.0 |
| 105 Shuangtan | Shuangtan | *P. weidenreichi* | upper RM1/2 | CMLST0911-105 | 307-273 | -14.3 | 0.2 | -4.4 | 0.4 |
| 036 Shuangtan | Shuangtan | *P. weidenreichi* | lower LM1/2 | CMLST1005-036 | 307-273 | -14.7 | 0.2 | -4.9 | 0.1 |
| 119 Shuangtan | Shuangtan | *P. weidenreichi* | upper RM1/2 | CMLST0911-119 | 307-273 | -14.6 | 0.2 | -4.6 | 0.1 |
| 110 Shuangtan | Shuangtan | *P. weidenreichi* | RM2 | CMLST0911-110 | 307-273 | -13.3 | 0.1 | -4.9 | 0.1 |

**SI section 13: Trace element analysis of teeth**

High-resolution trace-element geochemical analyses of fossil teeth can aid in the reconstruction of extinct hominin dietary behaviour. Teeth are an often-used material in this application. The mineralisation of enamel occurs in an incremental manner, recording the internal and external early-life exposures, thus recording elemental and isotope signals occurring with these exposures^62^. They are also more resistant to post-burial diagenesis than other material such as bone ^298^. This technique has been used previously in the reconstruction of diet, trophic levels^299,300^, breastfeeding and weaning history^301^ and migration patterns of extinct hominins and extant apes^302^. Elemental analysis of eleven *G. blacki*’s and two *P. weidenreichi’s* fossil teeth from four sites in the quaternary period, allow the comparison of potential behavioural changes in response to climatic variations. Especially, dietary and mobility adaptations to seasonal fluctuations are of particular interest, given the pollen data extracted from the sedimentary layers.

**Methods**

Fossil teeth were sectioned with a high-precision diamond saw and polished to >10 µm smoothness. Laser-ablation combined with inductively coupled plasma-mass spectrometry (LA-ICP-MS) was used for trace elemental mapping analyses of the samples according to the published protocol from^62^. The GARG facility at Southern Cross University uses an ESI NW213 coupled to an Agilent 7700 ICP-MS. These systems were used to map the samples using rastered laser beams run along the sample surface in a straight line. A laser spot size of 40 μm, a scan speed of 80 μm.s^-1^, laser intensity of 80%, and a total integration time of 0.50 s were used to produce data points that corresponded to a pixel size of approximately 40×40 μm. NIST610 and NIST612 (certified standard reference materials) were used to assess signal drift.

Elemental maps were constructed using the interactive R Shiny application “shinyImaging” (<http://labs.icahn.mssm.edu/lautenberglab/> to download the application). The app transforms individual laser line csv files for each isotope into a counts per second (cps) matrix (number of ablation lines multiplied by the number of ablation spots per ablation line). For each element, the gas blank collected during the first 10s of each laser lines are subtracted from the rasterstack and elements are normalised to ^43^Ca. The background around the teeth (signal arising from the encasing resin or air) was converted to white colouration (no intensity) to increase clarity of the figures by isolating the dental tissue from its surroundings. Colour scales were applied using the linear blue-red Lookup Table.

**Results**

Of the four sites chosen across the quaternary period, Chuifeng (early Pleistocene) and Queque (early mid-Pleistocene) are older sites, while Hejiang and Shuangtan are much younger (both late mid-Pleistocene), with dates falling right before the proposed extinction window. In general, the elemental maps showed distinct Sr/Ca and Ba/Ca banding in the samples from Chuifeng and Queque, with significantly less visible banding in the younger Shuangtan and Hejiang sites (ED9a). This biogenic banding can be identified in the form of multiple narrow lines across both the enamel and dentine (Fig. S21). This contrasts with the trace elemental mapping of *P. weidenreichi* teeth at Shuangtan that continues to display some form of banding during the same period.

**Discussion**

The Sr/Ca and Ba/Ca tended to be synchronous across all the older individuals, suggesting a common source of exposure or dietary habitats. This is a trend not observed in younger sites, with a more diffuse, some could say chronic distribution of elements across the dental tissues. While the pollen data shows a greater abundance and variety of flora availability during older periods, results also advocate for stronger seasonal variations in late mid-Pleistocene. Yet, Chuifeng and Queque individuals tended to show stronger banding patterns (Fig. 3d) commonly associated with high seasonality. Oppositely, individuals from younger sites such as Shuangtan and Hejiang show much less seasonality across all samples (ED9a *xi-xvi*). The lack of seasonality seen in younger teeth compared to the more distinct banding signal in older sites, might at first seem counterintuitive, although younger sites appeared to have poorer resolution in the elemental mapping (ED9a). Data extraction across the enamel/dentine junction reinforce, the strong variability and repetition in peaks (ED9c) of older sites compare to young ones.

Again, this might seem counterintuitive, with greater flora expected to be associated with greater seasonality, unless the high seasonality is associated with strong variation in vegetation due to high oscillation in hydrology. For example, with introduction to long droughts and heavy monsoon season. The pollen results produced in this study indicate that there was likely better biodiversity at the time of the older samples. Therefore, stronger banding in older sites likely reflect a larger diversity of food sources, including seasonal fruits and flowers. Most likely food sources would have been in greater availability all year long.

Oppositely, individuals from younger sites might have had greatly reduced available food sources, including poorer diversity on a potentially shrinking territory. While, we could expect to see a stronger variability in the dental tissues due to a higher seasonality, it appears plausible that the disappearance of flower and fruits might have forced *G. blacki* to adapt its behaviour to all year-round fall-back food to survive. The lack of food diversity and smaller territory could easily explain the decrease in banding observed in *G. blacki* samples from late mid-Pleistocene sites close to the extinction window. In comparison. *P. weidenreichi* from Shuangtan continues to display some form of banding indicating a more diverse fall-back food and a less stressed population.

**Diagenetic vs Biogenic signal**

Following the original elemental deposition, mineralised tissues can be altered during burial. Enamel, however has a far greater resistance to diagenesis than other tissues, due to its low porosity and low organic content^303^. Many elements are known to slowly diffuse into dental tissue during the deposition processes. Uranium is one of these markers of diagenesis, and was systematically mapped for each tooth to account for potential alteration. The distribution of uranium shows an expected diffuse pattern, distinct to the pattern of tooth growth, with typical enrichment around cracks and fractures (ED9b). It is clear that several zones of dentine and enamel in a lesser extent, show strong diagenetic processes, overshadowing some of the biogenic signal. Yet, the level of post-burial alterations, did not sufficiently alter the banding of Ba/Ca and Sr/Ca which continue to show a typical biogenic pattern of tooth growth, across both amelogenesis and detinogenesis (ED9b).

**SI section 14: Dental Microwear Textural Analysis**

**Background**

The diet of *G. blacki* has been fiercely debated for decades^80,304,305;306^. Since the 1950s, dietary reconstructions of *G. blacki* shifted from a carnivorous hunter of large ungulates^80^ to a C4-dominated grassland feeder^122^ specialised bamboo feeder^5^, forest dwelling frugivore^148^ and ‘all-round’ mixed feeder with a preference for C3 vegetation^6,26,86,307^. *G. blacki* is known for its unusually large molars, tooth root morphology and atypical enamel thickness that all increased in time^13,14^. These features may not only suggest a plausible giant body size^4,5,80,308,309^ but could indicate a diet of shearing tough, fibrous foods^13,14,16,17^. Some researchers suggest it is an adaptation to grinding of hard foods^26^. Only two earlier studies are known in which *G. blacki*’s dental microwear has been examined, through qualitative 2D scanning electron microscopy (SEM)^6,148^. Present study is the first quantitative 3D dental microwear study applied to *G. blacki*.

**Methods**

Dental microwear texture analysis (DMTA) is the study of dental microwear patterns, such as scratches and pits, in enamel on the occlusal surface of teeth. A confocal profilometric microscope is used to reconstruct the diet and feeding ecology of extant and extinct mammals, including great apes, *e.g.*^64,310-316^. DMTA specifically provides dietary indications of the last meal(s) (the ‘last supper effect’) during the final few weeks before death, *e.g.* ^317-319^. Developed from pioneering 2D stereomicroscopy and SEM^320-326^, DMTA has proven to be (semi)automatic, repeatable and more quantitative, which minimizes observer errors and makes data more reliable^327^.In this study, DMTA was performed on facet 9, as close as possible to the (ante mortem) puncture crushing or tip crushing point (Fig. S22) towards the lingual tip where facet 9 is in contact with lingual phase I facets 5 and 6. Facet 9 is a common abrasion-dominated phase II facet in the mammalian- and therefore primate chewing cycle^328-333^.

Left and right lower permanent molars (*n*_total_ = 41) of adult individuals regardless of gender were used, and comprised first (M_1_), second (M_2_) and third molars (M_3_). These include: fossil *G. blacki* (*n* = 16), comparative fossil *Pongo weidenreichi* (*n =* 22), and comparative extant *Pongo pygmaeus* (*n* = 3) (Table S26). Only molars that displayed moderately worn enamel surfaces were selected, in particular those with wear stages 2, 3 and 4^334^. In cases where isolated *G. blacki* first or second molars were difficult to identify, they were classified as ‘M1/2’. The majority of studied molars were excavated by Y. Zhang (Chinese Academy of Sciences, CAS) (*e.g.,* ^9,113^), at 12 of the 13 selected cave sites. Mafeng Cave was excluded from the DMTA. All fossil materials are stored at the Institute of Vertebrate Palaeontology and Palaeoanthropology (IVPP, Beijing). Although we acknowledge earlier interpretations of Early to Middle Pleistocene southern Chinese *Pongo* fossils assigned to *P. weidenreichi* and Late Pleistocene material to *P. devosi* ^89,335^, we will follow the latest interpretation that suggests all of the *Pongo* material during this age range from the Chongzuo area is likely *P. weidenreichi*^27^. Besides *P. weidenreichi*, additional comparative dentition comprises extant orang-utan, *P. pygmaeus*, which is derived from wild shot animals in Indonesia, and stored at the South Australian Museum (SAM, Adelaide). The latter species was only used for comparative dietary purposes at the species level and was not included in any boxplot time series (Fig. 2c; Fig. 3f, g), as these only apply to the fossil pongines of southern China in this paper.

Moulding and casting of the selected molars following standard DMTA procedures (*e.g.,*^64,65-67^). Molars were cleaned with acetone and cotton swabs to remove any lipid residues or remnants of moulding materials. Silicon moulds were taken from the occlusal surface with a dispenser gun (DS74 50 ml Coltène/ Whaledent, Henry Schein) loaded with polyvinylsiloxane (PVS) (AFFINIS regular body 50 ml, Coltène/Whaledent, Henry Schein), on location in the respective collections. In the following step, so-called ‘buckets’ – to pour the epoxy resin in – were made at Macquarie University with putty base and catalyst (AFFINIS base and catalyst 300 ml, Coltène/Whaledent, Henry Schein). Casting was performed with EPOTEK-301 two-component epoxy resin (ONBoard Solutions) at room temperature. The moulds with casting resin were placed in a centrifuge (GT-20, Spintron) at 1500 rpm for 1 min to remove air bubbles. Scanning was performed using blue light confocal microscopy^64,312,313,314^. Each cast was examined at a magnification of 20 × (EPI-N) to find a suitable (flat) surface for scanning towards the tip crush area on facet 9. Scans were taken at 100× (ELWD-N) magnification in rectangular grids of 242 × 181 μm^2^. Scanning was conducted on a PLμ neox confocal profiler (Sensofar 2009) at the Flinders University Palaeontology Microscopy Facility. The resultant scanned digital elevation models (DEMs) were prepared within SensoMap Premium V8.2.9564 (MountainsMap, DigitalSurf) following the ‘soft filter’ procedure^68^ and analysed with the embedded scale-sensitive fractal analysis (SSFA) module.

Quantitative-spatial data obtained from the 3D models enabled statistical analyses. Following^315^ we performed three MANOVAs, six one-way ANOVAs and 12 pairwise comparisons, in Minitab 19.2020.1., for the main two DMTA parameters: complexity (*Asfc*) and anisotropy (*epLsar*) (Fig. S22). Complexity is mostly visible in the form of pits on the enamel surface, from small and shallow to deep and wide, and are most prominent when chewing on hard or brittle foods like fruits, seeds and nuts, or lesser so on those that tend to crush and grind leaves. As such, complexity distinguishes ‘grinding’ taxa that consume brittle foods from those consuming softer or harder foods. Anisotropy is mostly visible in the form of scratches, from narrow and shallow, to deep, wide and elongated, caused by chewing on tough or fibrous foods, like grasses, shoots and bark. Phytoliths may also play a prominent role in scratch formation ^336^, as previously has been demonstrated in *G. blacki* by^5^. However, the role of fine dust (during drier periods) is increasingly being suggested as primary factor of increased scratches (resulting in higher anisotropy)^332^. Despite this conjecture, anisotropy has been a suitable parameter to distinguish taxa that consume fibrous foods from those consuming tender or tougher foods^64;310,312-315^. Anisotropy could even be considered a potential marker for reconstructing the Pleistocene history and distribution of fruit masting events in Southeast Asia^24^.

All analyses were performed on three different categories as factor or group variable. Firstly, one interspecific MANOVA between *species* in general (extinct *G. blacki* and *P. weidenreichi*, extant *P. pygmaeus*) was performed to determine the significant dietary variations between all species, regardless of site and age. Secondly, two intraspecific MANOVAs were performed between individual *G. blacki* bearing- and *P. weidenreichi*-bearing *sites*, to determine their respected dietary variations over time. Afterwards, for a similar purpose, with the same variables and categories, six one-way ANOVAs were performed on each dependent variable (two) per category (three). Finally, two pairwise comparison tests, Tukey’s Honest Significant Difference (HSD) *post-hoc* test and Fisher’s Least Significant Difference (LSD) *a priori* test, were run for each variable per category to balance the risks of Type I and Type II errors^337-339^ (Table S27). Data visualisation was carried out in R Studio 1.4.1717. (2009-2021) (Fig. 2c, ED10f, g).


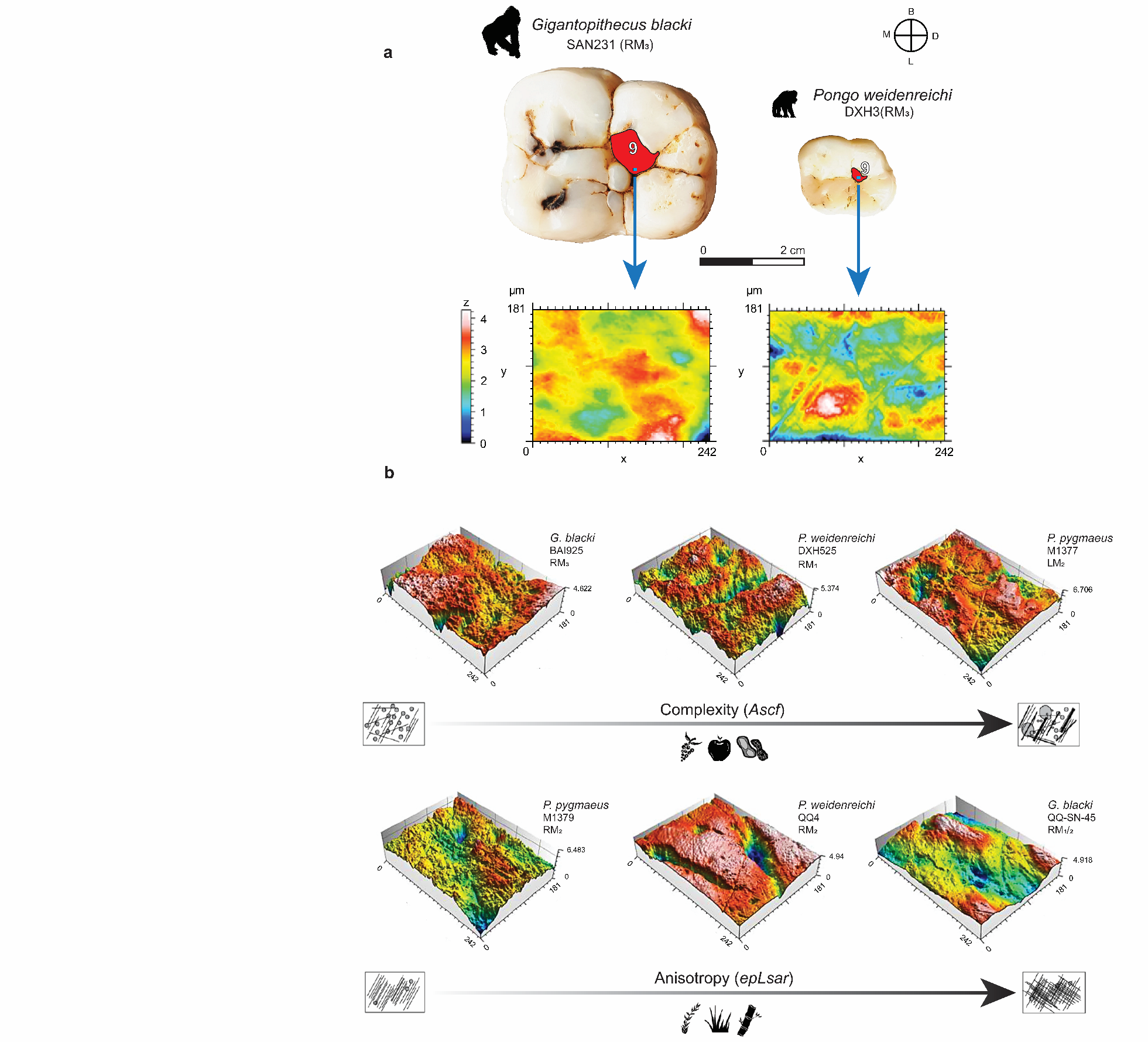


**Fig S22: DMTA methodology. a** The sample location of scanned areas (181 × 242 μm^2^) on facet 9 (red shade), close the tip crushing point. Exemplified here are a lower right M_3_ of *G. blacki* (left, SAN231) and *P. weidenreichi* (right, DXH3). The occlusal compass indicates the spatial directions, clockwise: buccal (B), distal (D), lingual (L) and mesial (M). 2D occlusal maps of the scanned areas are indicated by the blue arrows. **b** 3D surface models (181 × 242 μm^2^) of characteristic microwear for maximal values per parameter and species. **Top:** increasing complexity (*Asfc*) (from left to right), illustrated by small and shallow to deep and wide pits, caused by chewing on hard or brittle foods like fruits, seeds and nuts. **Bottom:** increasing anisotropy (*epLsar*) (from left to right), illustrated by narrow and shallow, to deep, wide and elongated scratches, caused by chewing on tough or fibrous foods, like grasses, shoots and bark. 3D-models are made in SensoMAP Premium 8.2.9564.

**Results**

Results of the statistical analyses are listed in Tables S27-28. The MANOVA performed between species with three different test criteria (Wilk’s Lambda, Lawley-Hotelling and Pillai’s Trace) shows that there is a significant difference between the DMTA signature of the species (*P* ≤ 0.001). Most of our significant results are ‘obviously’ significant (many with *P*-values <0.001). However, between *G. blacki*- and *P. weidenreichi*-bearing sites, the *P*-values indicate *no* significant difference (Table S27). Similarly, the one-way ANOVA results show that the only significant difference can be observed within the complexity between different species (Table S27). Both pairwise comparisons (Tukey’s HSD and Fisher’s LSD) support this by revealing that the only significant difference between species exists between the complexity of extant *P. pygmaeus* versus the other two extinct pongins (Table S28). Interestingly, the only significant differences observed within *G. blacki-*bearing sites are for anisotropy at four different sites: Baikong, Bapeng, Yanliang and Queque. There are no significant differences for complexity in *G. blacki*-bearing sites. Similarly, despite nuanced variations between the highest and lowest mean-values, there is no convincing statistical evidence for significant dietary differences in terms of complexity and anisotropy between *P. weidenreichi-*bearing sites (Table S28).

Based on the various statistical tests, scatterplots have been constructed of individual dental elements for interspecific dietary variation (ED10f, g) and boxplot time-series (Fig. 2c; Fig. 3f, g) for intraspecific dietary change over time. As displayed in ED10f, g, the statistical association of individually plotted data points in space (‘point clouds’) is weakest in *P. weidenreichi*, strongest in *P. pygmaeus* and intermediate in *G. blacki*. Generally, there seems to be a high overlap between the dietary variation of *G. blacki* with *P. weidenreichi.* However, where *G. blacki*, shows a relatively dominant distribution of data parallel aligned to the y-axis (anisotropy), this trend seems less clear in *P. weidenreichi.* It must be noted that one data point, representing one *G. blacki* M_3_ (+) from Baikong Cave, is excluded from the point cloud as this outlier shows much higher complexity (but is still included in the statistics and boxplot time-series). *P. weidenreichi* shows the widest distribution of data points in both anisotropy and especially complexity (x-axis) but this could be due to the higher sample size (*n* = 22) of *P. weidenreichi* compared to that of *G. blacki* (*n* = 16). It is clear, however, that extant *P. pygmaeus* shows the highest values in complexity and its cluster does not overlap with *G. blacki*.

In ED10 f,g individual dental elements are plotted per site (*n* = 14 of which 12 Chinese sites and two Indonesian sites), following the chronology from Early Pleistocene (top) to Holocene (bottom). Only three sites show overlap for the presence of *G. blacki* and *P. weidenreichi* fossils: Baikong, Queque and Bapeng. *G. blacki* shows a slightly higher complexity (excluding the outlier) at Baikong, while the opposite is true at Queque Cave. The anisotropy at both sites is similar between both species. At Bapeng Cave, although only based on one sample per species, *G. blacki* shows a lower anisotropy while their complexities are relatively similar.


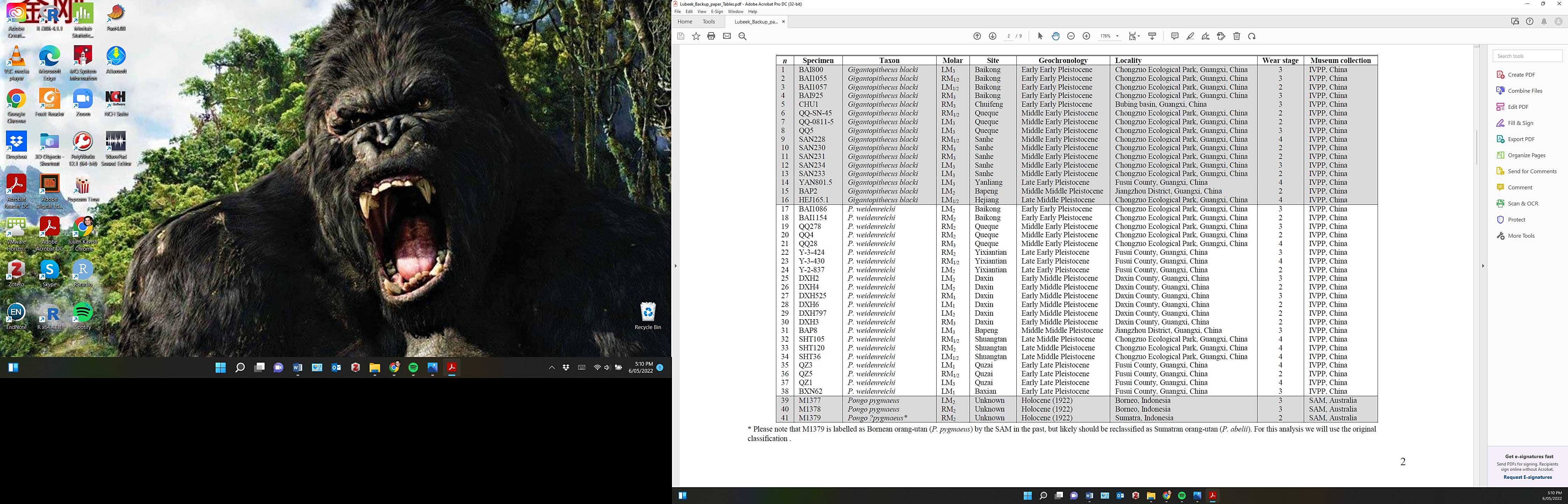
**Table S****26:** Overview of lower hominid molars (M1, M2, M3; n_total_ = 41) used in this study, categorised per species and site. Difficult to identify isolated G. blacki molars have been classified as first or second lower molars (M1/2). G. blacki and comparative fossil Pongo specimens were sampled at the Institute of Vertebrate Palaeontology and Palaeoanthropology (IVPP), comparative extant P. pygmaeus molars were sampled at the South Australian Museum (SAM). Wear stages follow^3^

When considering their mean complexity and anisotropy values over time, as illustrated in the boxplot time series per site (Fig. 2c; Fig. 3f, g), *G. blacki* tends to show slightly higher fluctuations, especially in the anisotropy trend line, while those of *P. weidenreichi* seem more stable. A potentially steeper decline in both complexity and anisotropy of *G. blacki* may be observed over the course of the Middle Pleistocene, from Yanliang Cave (~0.8-0.6 Ma) to Hejiang Cave (0.4-0.32 Ma) which is considered an important site associated with the period of extinction (grey shade in Fig. 2c; Fig. 3f, g) ^4,9,12^. Although *P. weidenreichi* also seems to show a decrease in complexity during the late Middle Pleistocene, this is a somewhat ‘delayed pattern’ followed by a likely steeper increase compared to *G. blacki*. In addition, there seems to be a slightly higher increase in anisotropy of *P. weidenreichi* towards the late Middle Pleistocene, which is the opposite of what is observed in *G. blacki*.

**Table S27.** Descriptive two-way statistics through three MANOVAs and six One-Way ANOVAs of two standard DMTA variables (complexity/Asfc and anisotropy/epLsar) for the three groups (species, *G. blacki*-bearing sites, and *P. weidenreichi*-bearing sites). Significant differences in p are indicated in bold font.

| **MANOVAs**^1^ | | | | | | |
| --- | --- | --- | --- | --- | --- | --- |
| **Group** |  | | | *DF* | |  |
| **Species** | *Criterion* | *Test statistic* | *F* | *Num.* | *Denom.* | *P-value* |
|  | Wilk’s Lambda | 0.549 | 6.459 | 4 | 74 | **<0.001** |
|  | Lawley-Hotelling | 0.818 | 7.361 | 4 | 72 | **<0.001** |
|  | Pillai’s Trace | 0.452 | 5.546 | 4 | 76 | **0.001** |
| *s* = 2; *m* = –0.5; *n* = 17.5 | | | | | | |
| ***G. blacki*-bearing sites** | *Criterion* | *Test statistic* | *F* | *Num.* | *Denom.* | *P-value* |
|  | Wilk’s Lambda | 0.213 | 1.001 | 14 | 12 | 0.505 |
|  | Lawley-Hotelling | 2.777 | 0.992 | 14 | 10 | 0.519 |
|  | Pillai’s Trace | 0.983 | 0.967 | 14 | 14 | 0.525 |
| *s* = 2; *m* = 2; *n* = 2 | | | | | | |
| ***P. weidenreichi*-bearing sites** | *Criterion* | *Test statistic* | *F* | *Num.* | *Denom.* | *P-value* |
|  | Wilk’s Lambda | 0.472 | 0.845 | 14 | 26 | 0.619 |
|  | Lawley-Hotelling | 0.940 | 0.806 | 14 | 24 | 0.656 |
|  | Pillai’s Trace | 0.612 | 0.881 | 14 | 28 | 0.586 |
| *s* = 2; *m* = 2; *n* = 5.5 | | | | | | |

^1^Two-way tests *s*, *m* and *n* are intermediate results used in computing the multivariate test statistics and their associated degrees of freedom

| **ONE-WAY ANOVAs**^2^ | | | | | | | | |
| --- | --- | --- | --- | --- | --- | --- | --- | --- |
|  | **Variables** | | | | | | | |
| **Group** | **Complexity (*Asfc*)** | | | | **Anisotropy (*epLsar*)** | | | |
|  |  | *DF* | |  |  | *DF* | |  |
| **Species** | *F* | *Num.* | *Denom.* | *P-value* | *F* | *Num.* | *Denom.* | *P-value* |
|  | 14.51 | 2 | 38 | **<0.001** | 0.08 | 2 | 38 | 0.922 |
| ***G. blacki*-bearing sites** | *F* | *Num.* | *Denom.* | *P-value* | *F* | *Num.* | *Denom.* | *P-value* |
|  | 0.58 | 6 | 9 | 0.737 | 2.59 | 6 | 9 | 0.096 |
| ***P. weidenreichi*-bearing**  **sites** | *F* | *Num.* | *Denom.* | *P-value* | *F* | *Num.* | *Denom.* | *P-value* |
|  | 0.91 | 7 | 14 | 0.526 | 0.61 | 7 | 14 | 0.736 |

^2^ Two-way tests

Null hypothesis H_0_: All means (x̄) are equal
Alternative hypothesis H_1_: Not all means (x̄) are equal
Significance level α = 0.05
Equal variances were assumed for the analysis
**Table S28.** Twelve two-way Tukey’s HSD and Fisher’s LSD pairwise tests comparisons of two standard DMTA variables (complexity/Asfc and anisotropy/epLsar) for the three groups (species, G. blacki-bearing sites, P. weidenreichi-bearing sites). Significant differences in p are indicated in **bold** font.

| **Species**^1^ |  |  |  |  | |  | |  |  |  | | | |  | |  |  |  |
| --- | --- | --- | --- | --- | --- | --- | --- | --- | --- | --- | --- | --- | --- | --- | --- | --- | --- | --- |
|  | *Species* |  |  |  | |  | | | | | | | **Grouping** | | | | | |
| *Variable* |  | *n* | *Mean* | *SD* | | *Whiskers (min, max)*^2^ | *Q1* | *Median* | | | *Q3* | *IQR* | ***Tukey’s HSD*** | | ***Fisher’s LSD*** | | | |
| Complexity (*Asfc*) | *P. pygmaeus*  *G. blacki*  *P. weidenreichi* | 3  16  22 | 2.710  0.887  0.865 | 0.403  0.615  0.547 | | (2.430, 3.172)  (0.285, 1.662)  (0.241, 1.866) | 2.429  0.415  0.475 | 2.529  0.733  0.695 | | | 3.172  1.268  1.151 | 0.743  0.853  0.676 | A B B | | A  B  B  A  A  A | | | |
| Anisotropy (*epLsar*) | *G. blacki*  *P. weidenreichi*  *P. pygmaeus* | 16  22  3 | 0. 018  0. 018  0. 018 | 0.001  0.001  0.000 | | (0.017, 0.020)  (0.017, 0.019)  (0.018, 0.018) | 0.018  0.018  0.018 | 0.018  0.018  0.018 | | | 0.019  0.019  0.018 | 0.001  0.001  0.001 | A A A | |  |  |  |  |
|  |  | |  |  | |  | |  | | | | | ***P*-values** | | | | | |
| *Variable* | *Difference of levels* | | | | |  | | | | | | | ***Tukey’s HSD*** | | ***Fisher’s LSD*** | | | |
| Complexity (*Asfc*) | ***P. pygmaeus***  *P. weidenreichi*  ***P. weidenreichi*** |  | | | **v. *G. blacki*** v. *G. blacki* **v. *P. pygmaeus*** | | | | | | | | **<0.001**  0.992  **<0.001** | | **<0.001**  0.903  **<0.001** | | | |
| Anisotropy (*epLsar*) | *P. pygmaeus*  *P. weidenreichi*  *P. weidenreichi* |  | | | v. *G. blacki* v. *G. blacki* v. *P. pygmaeus* | | | | | | | | 0.942  0.945  0.985 | | 0.743  0.750  0.868 | | | |

^1^Two-way tests

Null hypothesis H_0_: All means (x̄) are equal
Alternative hypothesis H_1_: Not all means (x̄) are equal

Significance level α = 0.05
Equal variances were assumed for the analysis
Means that do not share a letter are significantly different

Individual confidence level = 98.05% (Tukey’s HSD) and simultaneous confidence level = 87.99% (Fisher’s LSD).

^2^Whiskers excl. outliers

| ***G. blacki*-bearing sites**^1^ | | | | | | | | | | | | | |
| --- | --- | --- | --- | --- | --- | --- | --- | --- | --- | --- | --- | --- | --- |
|  |  |  |  | | | | | | | | **Grouping** | | |
| *Variable* | *Site* | *n* | *Mean* | *SD* | *Whiskers (min, max)* | | | *Q1* | *Median* | *Q3* | *IQR* | ***Tukey’s HSD*** | ***Fisher’s LSD*** |
| Complexity (*Asfc*) | Yanliang  Baikong  Sanhe  Hejiang  Bapeng  Queque  Chuifeng | 1  4  5  1  1  3  1 | 1.374 1.292 0.846 0.820 0.602 0.517 0.454 | * 0.846 0.681 * * 0.203 * | * (0.805, 2.558)  (0.288, 1.662)  *  *  (0.285, 0.661)  * | | | *  0.817  0.325  *  *  0.285  * | *  0.904  0.402  *  *  0.606  * | *  2.156  1.589  *  *  0.661  * | *  1.339  1.264  *  *  0.376  * | A  A  A   A  A  A  A | A  A   A  A  A  A  A |
| Anisotropy (*epLsar*) | Yanliang Queque Sanhe Chuifeng  Hejiang Baikong  Bapeng | 1  3  5 1 1 4 1 | 0.002 0.019 0.018 0.018 0.018 0.018 0.017 | *  0.001  0.000  *  *  0.001 * | *  (0.018, 0.020)  (0.018, 0.019)  *  *  (0.017, 0.018)  * | | | *  0.018  0.018  *  *  0.017  * | *  0.019  0.018  *  *  0.018  * | *  0.020  0.019  *  *  0.018  * | *  0.002  0.001  *  *  0.001  * | A  A  A  A  A  A  A | A  A  A B  A B  A B  B  B |
|  |  | |  | | |  | | | | | ***P*-values** | | |
| *Variable* | *Difference of levels* | | |  | | |  | | | | | ***Tukey’s HSD*** | ***Fisher’s LSD*** |
| Complexity (*Asfc*) | Bapeng  Chuifeng  Hejiang Queque Sanhe Yanliang Chuifeng Hejiang Queque Sanhe Yanliang Hejiang Queque Sanhe Yanliang Queque Sanhe Yanliang Sanhe Yanliang Yanliang | v. Baikong  v. Baikong  v. Baikong v. Baikong v. Baikong  v. Baikong v. Bapeng v. Bapeng v. Bapeng  v. Bapeng v. Bapeng  v. Chuifeng v. Chuifeng v. Chuifeng v. Chuifeng  v. Hejiang v. Hejiang v. Hejiang v. Queque  v. Queque v. Sanhe | |  | | |  | | | | | 0.960  0.909  0.994  0.736  0.945  1.000  1.000  1.000  1.000  1.000  0.978  1.000  1.000  0.997  0.950  1.000  1.000  0.996  0.992  0.913  0.988 | 0.383  0.295  0.546  0.166  0.349  0.916  0.880  0.824  0.916  0.749  0.439  0.710  0.937  0.608  0.360  0.706  0.973  0.576  0.521  0.300  0.493 |
| Anisotropy (*epLsar*) | Bapeng  Chuifeng  Hejiang **Queque** Sanhe **Yanliang** Chuifeng Hejiang **Queque** Sanhe **Yanliang** Hejiang Queque Sanhe Yanliang Queque Sanhe Yanliang Sanhe Yanliang Yanliang | v. Baikong  v. Baikong  v. Baikong **v. Baikong** v. Baikong  **v. Baikong** v. Bapeng v. Bapeng **v. Bapeng**  v. Bapeng **v. Bapeng**  v. Chuifeng v. Chuifeng v. Chuifeng v. Chuifeng  v. Hejiang v. Hejiang v. Hejiang v. Queque  v. Queque v. Sanhe | |  | | |  | | | | | 0.948  1.000  1.000  0.217  0.609  0.252  0.924  0.932  0.203  0.442  0.183  1.000  0.795  0.990  0.628  0.778  0.987  0.613  0.891  0.989  0.731 | 0.355  0.719  0.744  **0.027**  0.115  **0.032**  0.315  0.327  **0.025**  0.069  **0.022**  0.979  0.198  0.508  0.122  0.188  0.487  0.117  0.274  0.501  0.164 |

^1^Two way tests. Individual confidence level = 99.51% (Tukey’s HSD) and simultaneous confidence level = 64.85% (Fisher’s LSD).
*^*^*N/A when *n*=1

| ***P. weidenreichi-*bearing sites**^1^ | | | | | | | | | | | | | |
| --- | --- | --- | --- | --- | --- | --- | --- | --- | --- | --- | --- | --- | --- |
|  | *Site* |  |  | | | | | | | | | **Grouping** | |
| *Variable* |  | *n* | *Mean* | *SD* | *Whiskers (min, max)* | *Q1* | *Median* | *Q3* | *IQR* | ***Tukey’s HSD*** | | | ***Fisher’s LSD*** |
| Complexity (*Asfc*) | Daxin  Queque  Shuangtan  Quzai  Yixiantian  Bapeng  Baxian  Baikong | 6  3  3  3  3  1  1  2 | 1.223  0.948  0.887  0.816  0.798  0.425  0.370  0.267 | 0.812  0.312  0.265  0.432  0.390  *  *  0.037 | (0.617, 2.566)  (0.588, 1.138)  (0.711, 1.191)  (0.427, 1.280)  (0.491, 1.237)  *  *  (0.241, 0.294) | 0.618  0.588  0.711  0.427  0.491  *  *  0.241 | 0.836  1.112  0.759  0.741  0.667  *  *  0.267 | 2.041  1.138  1.191  1.280  1.237  *  *  0.294 | 1.423  0.549  0.481  0.854  0.746  *  *  0.053 | A  A  A   A  A  A  A  A | | | A  A   A  A  A  A  A  A |
| Anisotropy (*epLsar*) | Bapeng  Baxian  Queque  Shuangtan  Daxin  Yixiantian  Baikong  Quzai | 1  1  3  3  6  3  2  3 | 0.019  0.019  0.019  0.018  0.018  0.018  0.018  0.018 | *  *  0.001  0.001  0.001  0.000  0.000  0.001 | *  *  (0.018, 0.020)  (0.017, 0.019)  (0.017, 0.019)  (0.018, 0.018)  (0.018, 0.018)  (0.017, 0.018) | *  *  0.018  0.017  0.018  0.018  0.018  0.017 | *  *  0.018  0.018  0.018  0.018  0.018  0.018 | *  *  0.020  0.019  0.019  0.018  0.018  0.018 | *  *  0.002  0.003  0.001  0.000  0.001  0.001 | A  A  A  A  A  A  A  A | | | A  A  A  A  A  A  A  A |
|  |  | |  | | | | | | | | ***P*-values** | | |
| *Variable* | *Difference of levels* | | | | | | | | | ***Tukey’s HSD*** | | | ***Fisher’s LSD*** |
| Complexity (*Asfc*) | Bapeng  Baxian  Daxin  Queque  Quzai  Shuangtan  Yixiantian  Baxian  Daxin  Queque  Quzai  Shuangtan  Yixiantian  Daxin  Queque  Quzai  Shuangtan  Yixiantian  Queque  Quzai  Shuangtan  Yixiantian  Quzai  Shuangtan  Yixiantian  Shuangtan  Yixiantian  Yixiantian | v. Baikong  v. Baikong  v. Baikong  v. Baikong  v. Baikong  v. Baikong  v. Baikong  v. Bapeng  v. Bapeng  v. Bapeng  v. Bapeng  v. Bapeng  v. Bapeng  v. Baxian  v. Baxian  v. Baxian  v. Baxian  v. Baxian  v. Daxin  v. Daxin  v. Daxin  v. Daxin  v. Queque  v. Queque  v. Queque  v. Quzai  v. Quzai  v. Shuangtan | | | | | | | | 1.000  1.000  0.454  0.867  0.950  0.912  0.958  1.000  0.873  0.989  0.998  0.995  0.999  0.833  0.981  0.996  0.990  0.997  0.996  0.960  0.986  0.951  1.000  1.000  1.000  1.000  1.000  1.000 | | | 0.819  0.881  0.053  0.200  0.297  0.242  0.312  0.945  0.205  0.428  0.552  0.483  0.570  0.177  0.383  0.498  0.434  0.515  0.495  0.317  0.406  0.297  0.774  0.894  0.745  0.878  0.970  0.848 |
| Anisotropy (*epLsar*) | Bapeng  Baxian  Daxin  Queque  Quzai  Shuangtan  Yixiantian  Baxian  Daxin  Queque  Quzai  Shuangtan  Yixiantian  Daxin  Queque  Quzai  Shuangtan  Yixiantian  Queque  Quzai  Shuangtan  Yixiantian  Quzai  Shuangtan  Yixiantian  Shuangtan  Yixiantian  Yixiantian | v. Baikong  v. Baikong  v. Baikong  v. Baikong  v. Baikong  v. Baikong  v. Baikong  v. Bapeng  v. Bapeng  v. Bapeng  v. Bapeng  v. Bapeng  v. Bapeng  v. Baxian  v. Baxian  v. Baxian  v. Baxian  v. Baxian  v. Daxin  v. Daxin  v. Daxin  v. Daxin  v. Queque  v. Queque  v. Queque  v. Quzai  v. Quzai  v. Shuangtan | | | | | | | | 0.979  0.986  1.000  0.954  1.000  1.000  1.000  1.000  0.988  1.000  0.883  0.994  0.983  0.993  1.000  0.909  0.997  0.989  0.956  0.986  1.000  1.000  0.714  0.987  0.955  0.989  0.998  1.000 | | | 0.372  0.408  0.785  0.304  0.687  0.757  0.908  0.953  0.418  0.895  0.214  0.479  0.391  0.462  0.952  0.239  0.523  0.431  0.309  0.408  0.932  0.868  0.121  0.415  0.307  0.430  0.563  0.828 |

^1^Individual confidence level = 99.67% (Tukey’s HSD) and simultaneous confidence level = 56.54% (Fisher’s LSD).
*^*^*N/A when *n*=1

**Discussion: *G. blacki vs. P. weidenreichi*, differences in diet and foraging strategies**

Bearing in mind that DMTA reflects only short-term dietary patterns prior to death, our results may not necessarily indicate long-term trends. In addition, the narrow framework of comparative microwear data used in this study, only comprising of great apes and excluding locally well-studied and contemporary primates such as *Macaca*, *Trachypithecus* and *Rhinopithecus* (*e.g*. ^12^), also warrants careful interpretation of the results. Although all three great ape species in our study were predominantly frugivorous, there exists some significant difference in diet at the species level, as reflected by the MANOVA results (Table S27). This is caused by *P. pygmaeus*, which has significantly higher means in complexity compared to *G. blacki* and *P. weidenreichi*, as demonstrated by the interspecific one-way ANOVAs (Table S27) and pairwise comparisons (Table S27). However, having relatively small sample sizes (per species, molar specimen and site) made it difficult to ensure that the various model assumptions were satisfied (such as normality of residuals and approximate equality of variables). Contrary to *P. pygmaeus*, in which the *n* represents the precise minimum number of individual animals (MNI) from which teeth were sampled, the MNI of *G. blacki* and *P. weidenreichi* is likely lower than their respective *n* due to isolated fossil specimens of the same molar type at some sites. To overcome the small sample size, we only focused on facet 9 and grouped all dental specimens together, either per species or site. Although M_2_ is the commonly preferred molar in (both micro- and macro-) dental wear studies (*e.g.,* ^332^), the reason for comparing microwear in facet 9 of different molar types is simply to increase the sample size. We believe that comparing higher magnified ‘single facet’ *micro*wear between different molars is less problematic compared to lower magnified ‘multiple facet’ *macro*wear patterns on the occlusal surface of different molar types. Results should nevertheless be interpreted with care.

All (extant) great apes are generally known to be dietary opportunists with a preference for high nutritional foods such as fruits that are soft in pulp and occur in large quantities^340^. Compared to gorillas and chimpanzees, orang-utans are known to incorporate the broadest range of fruit-bearing plant species in their diet^24,341-343^ and are also known to consume nuts, seeds, flowers, vines, pith, bark, shoots, leaves, insects and occasionally small mammals as fall-back foods during seasons of fruit scarcity^24,341,344,345^. This is reflected by their dental morphology, masticatory pathways and wear facet patterns that show more heavily pitted, abrasive worn occlusal surfaces^346-350^. Our microwear results support this by higher complexity values for *P. pygmaeus*. For comparison, *Gorilla* diets (and potentially that of *G. blacki*) are known to differ from *Pongo* (and other great apes such as *Pan*) in the degree to which they consumed leaves and other fibrous foods^351^, which could be reflected in higher anisotropy^352^. The mountain gorilla (*G. b. beringei*) in particular is considered to have the most restricted diet consisting of mechanically resistant and low nutritional foods such as leaves, pith, bark and bamboo. Ecological specialists with small geographic ranges, such as *G. b. beringei*, are therefore more prone to extinction^146,351,353-357^.

Given the chronological and temporal differences in climatic and environmental conditions between the species’ habitats, the seasonal variation in fruit abundance may clarify the higher complexity values in *P. pygmaeus*. Modern orang-utan habitats are situated in year-round tropical insular SEA. Although there are small differences in diet and foraging strategies between Holocene Sumatran orang-utan (*P. abelii*) and Bornean orangutan (*P. pygmaeus*), with the former having a more stable access to fruits and fall-back foods ^24^, their ancestral forms, Late Pleistocene *P. palaeosumatrensis*, *P. javensis* and *P. duboisi*^358^, are also known to be part of Late Pleistocene rainforest faunal assemblages^27,305,335,359,360^. In contrast, Early to Middle Pleistocene *P. weidenreichi* and Late Pleistocene *P. devosi*^89^ of mainland SEA, including southern China, are known to have experienced habitat loss by southern shifts in the northern tropical and sub-tropical zones and increased monsoon-driven seasonality during Pleistocene glacial conditions (*e.g.*^361,362^), resulting in savannah environments that replaced rainforests throughout the region (*e.g.* the ‘central savannah corridor’ of Sundaland^19,24,359,363^). The stable rain forest habitats further south, in modern-day Sumatra and Borneo, may have acted as refugia during glacial periods, although recent re-evaluation of orang-utan palaeoecology and distributions suggest that they may have been more resilient to environmental changes than previously thought ^363^. Regardless, the seasonal impacts on fruit availability during the glacial periods in southern China were more severe compared to Indonesia, which may clarify lower complexity values in *G. blacki* and *P. weidenreichi* compared to *P. pygmaeus*.

As pointed out by the pairwise comparisons between the fossil species, *G. blacki* tends to hold a slightly higher mean difference for anisotropy values, and *P. weidenreichi* a slightly higher mean difference for complexity values. These differences are very subtle. Whether *G. blacki* was more of a ‘shearer’ and *P. weidenreichi* more of a ‘grinder’, at least during periods of fruit scarcity, cannot be confirmed based on this data. Although such periods show some similarities with *masting event**s*^364-366^ or periods of overabundant fruit production by closed canopy trees (driven by drier conditions during El Niño Southern Oscillation)^367-371^, the timescale of these periods are tens of thousands of years in contrast to months or years. Our limited data does not necessarily support earlier suggestions of researchers^13,14,16,17^ that *G. blacki*’s unusually large molars, tooth root morphology and atypical enamel thickness indicate a ‘shearing’ dietary adaptation towards tough, fibrous food. However, in line with ^372^ interpretation on the discrepancy between the robust functional morphology of *Paranthropus boisei* and the lack of microwear evidence for primary consumption of hard, brittle and mechanically-challenging foods, the small differences in our results may reveal *G. blacki*’s diet might have been more varied than its ‘specialised’ morphology suggests. The labels ‘folivore’ versus ‘frugivore’ may therefore not necessarily be useful in this case^372^.

*G. blacki* may have been a mixed feeder, like all great apes, but with a slightly more specific preference for, or reliance on, particular components of the (tougher) vegetation as fall-back foods. As addressed earlier, ecological specialists with small geographic ranges, such as the mountain gorilla, are more prone to extinction ^146,351,353-357^. This may have been the case for *G. blacki* as well. Although a plethora of studies on comparative dental morphology (*e.g.*^10,122,129,373-376^), enamel-dentine junction computed tomography (CT)^63^ and proteomics^7^ confirm a close phylogenetic relationship between the *G. blacki* and *P. weidenreichi*, in terms of behaviour it was likely closer to a gorilla. *G. blacki*’s robust mandible and potentially giant body size may have also been more comparable to that of a large terrestrial gorilla^146,377^ than to an arboreal orang-utan.

From an evolutionary perspective, *Sivapithecus*^344,378-381^, the pongine ancestor of *Pongo* and possibly sister taxon of *Gigantopithecus*, may have had terrestrial lifestyle too ^382^. This Miocene pongine is also known it for its thick molar enamel of which daily enamel secretion rates and periodicity are closest to *G. blacki*, less close to *G. g. gorilla* and most distant from *P. pygmaeus* ^383^. As such, a terrestrial and potentially social harem-group structured lifestyle, similar to that of (mountain) gorillas ^146,384,385^ seems plausible for *G. blacki* that was likely too heavy to climb trees.

In contrast, *P. weidenreichi* may have had a semi-solitary, arboreal, lifestyle in smaller groups, with a wider geographic range, as supported more a wider distribution of *P. weidenreichi* fossils among the (DMTA sampled) sites. Modern orang-utans are known as the world’s largest living semi-solitary forest-canopy animals while having body weights exceeding 80 kg (*e.g.* ^342,344,386-388^). A potentially arboreal lifestyle of *P. weidenreichi* may have facilitated a better access to softer fruit pulp and younger, higher nutritional fresh leaves (which are easier to digest) resulting in lower anisotropy and complexity.

In addition, given the wider geographic distribution in potentially smaller groups, may have increased its chances for survival. *P. weidenreichi* may have been more flexible in consumption of fall-back foods and could have adapted more easily to environmental changes, making *P. weidenreichi* less vulnerable and susceptible to extinction. Given *G. blacki*’s plausible (increasing) giant body size ^4,5,80,308,309^, the higher demand for low nutritional food items, during periods of fruit scarcity, could possibly have outweighed the availability in supply of these foods. A more conservative foraging ecology, a limited foraging area and/or narrower dietary flexibility for *G. blacki*, at least during the early Early Pleistocene, is also supported by a recent stable isotope study^307^.

**References**

69. Joannes-Boyau R., Duval, M. & Bodin, T. MCDoseE 2.0. A new Markov Chain Monte Carlo program for ESR dose response curve fitting and dose evaluation. *Quat. Geochron* **44**, 13-22 (2018).

70. Shao, Q., Bahain, J.-J., Dolo, J.-M. & Falguères, C. Monte Carlo approach to calculate US-ESR age and age uncertainty for tooth enamel. *Quat. Geochron.* **22**, 99-106 (2014).

71. von Koenigswald, G. H. R. *Gigantopithecus blacki* a giant fossil hominoid from the Pleistocene of southern China. *Anthropological Papers of the American Museum of Natural History* **43**, 292–325 (1952).

72. Cullen, C., & Lo, V. *Medieval Chinese Medicine: The Dunhuang Medical Manuscripts*. (Routledge, 2004).

73. Davidson, T. On some fossil brachiopods, of the Devonian age, from China. *Quarterly Journal of the Geological Society* **9**, 353–359 (1853).

74. Schlosser, M. Die fossilen Säugetiere Chinas nebst einer Odontographie der rezenten Antilopen. *Abhandlungen der Bayerischen Akademie der Wissenschaften* **2**, 1-221 (1903).

75. Weidenreich, F. Morphology of Solo man. *Anthropological Paper of the American Museum of Natural History* **43**, 205-290 (1951).

76. Tobias, P.V. The life and times of Ralph von Koenigswald: Paleontologist extraordinary, *Journal of Human Evolution* **5**, 403-410 (1976).

77. Pei, W. C. Fossil mammals from the Kwangsi Caves. *Bulletin of the Geological Society of China* **14**, 413–425 (1935).

78. Teilhard de Chardin, P., Young, C. C., Pei, W. C., & Chang, H. C. On the Cenozoic formations of Kwangsi and Kwangtung. *Bulletin of the Geological Society of China* **14**, 179–210 (1935).

79. Weidenreich, F. Giant early man from Java and South China. *Anthropological Papers of the American Museum of Natural History* **40**, 1–134 (1945).

80. Pei, W. C. Discovery of *Gigantopithecus* mandible and other material in Liucheng district of central Kwangsi in South China. *Vertebrata Palasiatica* **1**, 65–72 (1957).

81. Pei, W. C. Excavation of Liucheng *Gigantopithecus* cave and exploration of other caves in Kwangsi. *Memoir of the Institute of Vertebrate Palaeontology and Palaeoanthropology, Academia Sinica* **7**, 1–54 (1965).

82. Pei, W. C., & Woo, J. K. New materials of *Gigantopithecus* teeth from South China. *Acta Palaeontologica Sinica* **4**, 477–490 (1956).

83. Ciochon, R.L. The ape that was. *Natural History* **11**, 54–63 (1991).

84. Chang, Y., Wu, M., & Liu, C. New discovery of *Gigantopithecus* teeth from Wuming, Kwangsi. *Chinese Science Bulletin* **18**, 130–133 (1973).

85. Han, F., *et al*. Preliminary results of combined ESR/U-series dating of fossil teeth from Longgupo cave, China. *Quaternary Geochronology* **10**, 436–442 (2012).

86. Nelson, S. V. The paleoecology of Early Pleistocene *Gigantopithecus blacki* inferred from isotopic analyses. *American Journal of Physical Anthropology* **155**, 571–578 (2014).

87. Ciochon, R. L. Divorcing hominins from the *Stegodon-Ailuropoda* fauna: New views on the antiquity of hominins in Asia. in *Out of Africa I: the ﬁrst hominin colonization of Eurasia* (eds. Fleagle, J. G., Shea, J. J., Grine, F. E., Baden, A. L., & Leakey, R. E.) 111–126 (Springer, 2010).

88. Ciochon, R. L., *et al*. Dated co-occurrence of *Homo erectus* and *Gigantopithecus* from Tham Khuyen Cave, Vietnam. *Proceedings of the National Academy of Sciences of the United States of America* **93**, 3016–3020 (1996).

89. Schwartz, J. H., Long, V. T., Cuong, N. L., Kha, L. T., & Tattersall, I. A review of the Pleistocene hominoid fauna of the Socialist Republic of Vietnam (excluding Hylobatidae). *Anthropological Papers of the American Museum of Natural History* **76**, 1–24 (1995).

90. Marwick, B. Biogeography of Middle Pleistocene hominins in mainland Southeast Asia: A review of current evidence. *Quaternary International* **202**, 51–58 (2009).

100. Bocherens, H., Schrenk, F., Chaimanee, Y., & Kullmer, O. Flexibility of diet and habitat in Pleistocene South Asian mammals: Implications for the fate of the giant ape *Gigantopithecus*. *Quaternary International* **434**, 148–155 (2017).

101. Qu, Y., Jin, C., Zhang, Y., Hu, Y., & Shang, X. Preservation assessments and carbon and oxygen isotopes analysis of tooth enamel of *Gigantopithecus blacki* and contemporary animals from Sanhe Cave, Chongzuo, South China during the Early Pleistocene. *Quaternary International* **354**, 52–58 (2014).

102. Jin, C. *et al*. Micromammals of the *Gigantopithecus* fauna from Sanhe Cave, Chongzuo, Guangxi. *Quaternary Sciences* **28**, 1129–1137 (2008).

103. Li, S. P., *et al*. Palynological analysis of the late Early Pleistocene sediments from Queque Cave in Guangxi, South China. *Quaternary International* **354**, 24–34 (2014).

104. van Weer, D. J., & Zheng, S. Biometric analysis and taxonomic allocation of Pleistocene *Hystrix* specimens (Rodentia, porcupines) from China. *Beaufortia* **48**, 47–69 (1998).

105. Huang, W., *et al*. Early *Homo* and associated artefacts from Asia. *Nature* **378**, 275–278 (1995).

106. Woo, J. K. The mandibles and dentition of *Gigantopithecus*. *Paleontologica Sinica, New Series D* **11**, 1–94 (1962).

107. Wang, W. New discoveries of *Gigantopithecus blacki* teeth from Chuifeng Cave in the Bubing Basin, Guangxi, south China. *Journal of Human Evolution* **57**, 229–240 (2009).

108. Wang, W. *et al.* Early Pleistocene hominid teeth recovered in Mohui cave in Bubing Basin, Guangxi, South China. *Chinese Science Bulletin* **50**, 2777–2782 (2005).

109. Wang, W., Tian, F., & Mo, J. Recovery of *Gigantopithecus blacki* fossils from the Mohui Cave in the Bubing Basin, Guangxi, South China. *Acta Anthropologica Sinica* **26**, 329–343 (2007).

110. Shao, Q. *et al*. Coupled ESR and U-series dating of Early Pleistocene *Gigantopithecus* faunas at Mohui and Sanhe Caves, Guangxi, southern China. *Quaternary Geochronology* **30**, 524–528 (2015).

111. Zhao, L. X., Tong, H. W., Xu, C. H., Yuan, Z. X., & Cai, H. Y. New discovery of *Gigantopithecus blacki* tooth fossil from Bijie, Guizhou and its signiﬁcance. *Quaternary Sciences* **26**, 548–554 (2006).

112. Liu, J., Zhao, L., Chen, J., Wang, X., Cai, H., & Zhang, Z. The age and environment study of *Gigantopithecus* fauna of Ba’eryan, Bijie, Guizhou, based on the carnivore fossils. *Quaternary Sciences* **31**, 654–666 (2011).

113. Zhang, Y., Jin, C., Kono, R. T., Harrison, T., & Wang, W. A fourth mandible and associated dental remains of *Gigantopithecus blacki* from the Early Pleistocene Yanliang Cave, Fusui, Guangxi, South China. *Historical Biology* **28**, 95–104 (2016).

114. Xu, C. H., Han, K. X., & Wang, L. H. Discovery of *Gigantopithecus* teeth and associated fauna in western Hopei. *Vertebrata Palasiatica* **12**, 293–309 (1974).

115. Zhang, Y., Zhang, Z., & Liu, W. Paleoanthropology. in *Jianshi hominid site*. (ed. Zheng, S.) 26–36 (Science Press, 2004).

116. Cheng, J., Zheng, S., Gao, Z., Zhang, Z., Feng, X., & Wang, X. Natural environment during the living period of the early human and gigantic ape in west Hubei. *Dizhi Xuebao* **80**, 473–480 (2006).

117. Chang, Y., Wu, M., & Liu, C. New discovery of *Gigantopithecus* teeth from Wuming, Kwangsi. *Chinese Science Bulletin* **18**, 130–133 (1973).

118. Chang, Y., Wang, L., Dong, X., & Chen, W. Y. Discovery of a *Gigantopithecus* tooth from Bama District in Kwangsi. *Vertebrata Palasiatica* **13**, 148–154 (1975).

119. Shao, Q. *et al*. U-series and ESR/U-series dating of the *Stegodon- Ailuropoda* fauna at Black Cave, Guangxi, southern China with implications for the timing of the extinction of *Gigantopithecus blacki*. *Quaternary International* **434**, 65–74 (2017).

120. Smith, R. J., & Jungers, W. L. Body mass in comparative primatology. *Journal of Human Evolution* **32**, 523–559 (1987).

121. Fleagle, J. G. *Primate adaptation & evolution.* (Academic Press, 2013).

122. Simons, E. L., & Ettel, P. C. *Gigantopithecus*. *Scienti*ﬁ*c American* **222**, 76–85 (1970).

123. Weidenreich, F. The dentition of *Sinanthropus pekinensis*. *Palaeontologia Sinica, New Series D* **101**, 1–180 (1937).

124. Broom, R. The dentition of the Transvaal Pleistocene anthropoids, *Plesianthropus* and *Paranthropus*. *Annals of the Transvaal Museum* ***19***, 303–314 (1939).

125. Broom, R. & Schepers, G. W. H. The South African fossil ape-men, the Australopithecinae. *Transvaal Museum Memoir* **2**, 1–272 (1946).

126. Pilgrim, G. E. New Siwalik primates and their bearing on the question of the evolution of man and the Anthropoidea. *Records of the Geological Survey of India* **45**, 1–74 (1915).

127. Eckhardt, R. B. *Gigantopithecus* as a hominid ancestor. *Anthropologischer Anzeiger* **34**, 1–8 (1973).

128. Eckhardt, R. B. *Gigantopithecus* as a hominid. in *Paleoanthropology, morphology and palaeoecology* (ed. Tuttle, R.L) 105–127. (Mouton, 1975).

129. Pilbeam, D. *Gigantopithecus* and the origins of Hominidae. *Nature* **225**, 516–519 (1970).

130. Simons, E. L., & Chopra, S. R. K. *Gigantopithecus* (Pongidae, Hominoidea) a new species from North India. *Postilla* **138**, 1–18 (1969).

131. Begun, D. R. The Miocene hominoid radiations. in *A companion to paleoanthropology* (ed. Begun, D. R.) 398–416 (Wiley-Blackwell, 2013).

132. Begun, D. R. Fossil record of Miocene hominoids. in *Handbook of paleoanthropology* (eds. Henke, W. & Tattersall, I.) 1261–1332 (Springer, 2015).

133. Harrison, T. Apes among the tangled branches of human origins. *Science* **327**, 532–534 (2010).

134. von Koenigswald, G. H. R. A possible ancestral form of *Gigantopithecus* (Mammalia, Hominoidea) from the Chinji Layers of Pakistan. *Journal of Human Evolution* **10**, 511–515 (1981).

135. von Koenigswald, G. H. R. The signiﬁcance of hitherto undescribed Miocene hominoids from the Siwaliks of Pakistan in the Senckenberg Museum, Frankfurt. in *New interpretations of ape and human ancestry* (eds. Ciochon, R. L. & Corruccini, R. S.) 517-526 (Plenum Press, 1983).

136. Szalay, F. S., & Delson, E. *Evolutionary history of the primates* (Academic Press, 1979).

137. Kelley, J. The hominoid radiation in Asia. in *The primate fossil record* (ed. Hartwig, W. C.) 369–384 (Cambridge University Press, 2002).

138. Cameron, D. W. A functional and phylogenetic interpretation of the Late Miocene Siwalik hominid *Indopithecus* and the Chinese Pleistocene hominid *Gigantopithecus*. *Himalayan Geology* **24**, 19–28 (2003).

139. Miller, S. F., White, J. L., & Ciochon, R. L. Assessing mandibular shape variation within *Gigantopithecus* using a geometric morphometric approach. *American Journal of Physical Anthropology* **137**, 201–212 (2008).

140. Oxnard, C. E. *Fossils, teeth and sex: New perspectives on human evolution*. (University of Washington Press, 1987).

141. Bronikowski, A., *et al*. Female and male life tables for seven wild primate species. *Sci Data* **3**, 160006 (2016). https://doi.org/10.1038/sdata.2016.6

142. Gatti S, Levréro F, Ménard N, & Gautier-Hion A. Population and group structure of western lowland gorillas (*Gorilla gorilla gorilla*) at Lokoué, Republic of Congo. *Am J Primatol*. **63**, 111–123 (2004). doi:10.1002/ajp.20045

143. Plavcan, J. M. Understanding dimorphism as a function of changes in male and female traits. *Evolutionary Anthropology* **20**, 143–155 (2011).

144. Zhang, Y. Variability and evolutionary trends in tooth size of *Gigantopithecus blacki*. *American Journal of Physical Anthropology* **59**, 21–32 (1982).

145. Zhang, Y. Variability in tooth size of *Gigantopithecus blacki* and the dietary hypothesis for australopithecines. *Acta Anthropologica Sinica* **2**, 205–217 (1983).

146. Groves, C. P. *Gigantopithecus* and the mountain gorilla. *Nature* **226**, 973–974 (1970).

147. White, T. D. Geomorphology to paleoecology: *Gigantopithecus* reappraised. *Journal of Human Evolution* **4**, 219–233 (1975).

148. Daegling, D. J., & Grine, F. E. Bamboo feeding, dental microwear, and diet of the Pleistocene ape *Gigantopithecus blacki*. *South African Journal of Science* **90**, 527–532 (1994).

149. Ciochon, R. L. The mystery ape of Pleistocene Asia. *Nature* **459**, 910–911 (2009).

150. Lopatin, A. V., Maschenko, E. N., & Dac, L. X. *Gigantopithecus blacki* (Primates, Ponginae) from the Lang Trang Cave (Northern Vietnam): The Latest *Gigantopithecus* in the Late Pleistocene? *Doklady Biological Sciences* **502**, 6-10 (2022).

151. Noerwidi, S., Siswanto, & Widianto, H. Giant primate of Java: a new *Gigantopithecus* specimen from Semedo. *Berkala Arkeologi* **36**, 141-160 (2016).

152. Hu, L., *et al*. Permo-Triassic detrital records of South China and implications for the Indosinian events in East Asia. *Palaeogeography, Palaeoclimatology, Palaeoecology* **485**, 84–100 (2017).

153. Waltham, T. (2009). The karst lands of southern China. *Geology Today* **25**, 232–238 (2009).

154. Bacon, A.M. *et al*. New palaeontological assemblage, sedimentological and chronological data from the Pleistocene Ma U'Oi cave (northern Vietnam). *Palaeogeography, Palaeoclimatology, Palaeoecology* **230,** 280–298 (2006).

155. Bacon, A.-M. et al. The late Pleistocene Duoi U’Oi cave in northern Vietnam: palaeontology, sedimentology, taphonomy and palaeoenvironments. *Quaternary Science Reviews* **27**, 1627–1654 (2008).

156. Sweeting, M. M. *Karst in China - Its Geomorphology and Environment*. (Hong Kong: Springer, 1995).

157. White, T. D. Geomorphology to paleoecology: *Gigantopithecus* reappraised. *Journal of Human Evolution* **4**, 219-233 (1975).

158. Williams, P. W. Geomorphic inheritance and the development of tower karst. *Earth Surface Processes and Landforms* **12**, 453-465 (1987).

159. Huang, S. *et al*. Paleoenvironmental background of the Early Pleistocene *Gigantopithecus* fauna in Bubing Basin, south China. *Quaternary International* **434**, 163–168 (2017).

160. Wang, W. *et al*. Sequence of mammalian fossils, including hominoid teeth, from the Bubing Basin caves, South China. *Journal of Human Evolution* **52**, 370–379 (2007).

161. Palmer, A. N. Origin and morphology of limestone caves. *Geological Society of America Bulletin* **103**, 1–21 (1991).

162. Li, Y. J., Lu, J., Li, Y. L., H, J. L., Huang, X. S., Huang, Z., & Zhou, M. L. Climate survey of Guangxi in 2020. *Journal of Meterorological Research and Application* **42**, 100-104 (2021).

163. Qin, C., He, J. L., Li, Y. L., Lu, J., Li, Y. J., Huang, X. S., Liao, S. S., & Zhou, M. L. (2022). Overview of Guangxi climate in 2021. *Journal of Meteorological Research and Application* **43**, 84-89 (2022).

164. Wang, Y., *et al*. The Early Pleistocene *Gigantopithecus-Sinomastodon* fauna from Juyuan karst cave in Boyue Mountain, Guangxi, South China. *Quaternary International* **434**, 4-16 (2017).

165. Pan, Y., Zhang, Y., Yang, L., Takai, M., Harrison, T., Westaway, K., & Jin, C. Preliminary description of a late Middle Pleistocene mammalian fauna prior to the extinction of *Gigantopithecus blacki* from the Yixiantian Cave, Guangxi ZAR, South China. *The Anatomical Record* (2023). https://doi.org/10.1002/ar.25200.

166. Kostopoulos D. S., Guy, F., Kynigopoulou, Z., Koufos, G. D., Valentin, X., & Merceron, G. A 2Ma old baboon-like monkey from Northern Greece and new evidence to support the Paradolichopithecus-Procynocephalus synonymy (Primates: Cercopithecidae). *Journal of Human Evolution* **121**, 178-192 (2018).

167. Qiu, Z. X., Deng, T. & Wang, B. Y. Early Pleistocene mammalian fauna from Longdan, Dongxiang, Gansu, China. *Palaeontologia Sinica*, *New Series C* **27**, 1-193 (2004).

168. Liu, J. Y., *et al*. The giant short-faced hyena Pachycrocuta brevirostris (Mammalia, Carnivora, Hyaenidae) from Northeast Asia: a reinterpretation of subspecies differentiation and intercontinental dispersal. *Quaternary International* **577**, 29-51 (2021).

169. Puspaningrum, M. R., van den Bergh, G. D., Chivas, A. R., Setiabudi, E., & Kurniawan, I. Isotopic reconstruction of Proboscidean habitats and diets on Java since the Early Pleistocene: Implications for adaptation and extinction. *Quaternary Science Reviews* **228**, 106007 (2020).

170. Chen, S. K., Li, Q. & Wang, X. M. Chalicothere fossils from the early Late Miocene of the Qaidam Basin, and their paleoenvironmental implications. *Quaternary Sciences* **35**, 528-538 (2015).

171. Sanz-Pérez, D., Fernandez, M. H., Munoz-Garcia, M. B., Perez-Dios, P., Morales, J., & Domingo, L. Palaeoecological and palaeoenvironmental reconstruction of the upper Miocene vertebrate karstic site of Corral de Lobato, central-eastern Spain. *Palaeogeography, Palaeoclimatology, Palaeoecology* **556**, 109877 (2021).

172. Liu, W. H., Dong, W., Liu, J. Y., Fang, Y. S. & Zhang, L. M. New materials of the Early Pleistocene mammalian fauna from Tuozidong, Tangshan, Nanjing and the indications of paleoenvironment. *Quaternary Sciences* **35**, 596-606 (2015).

173. Rizal, Y., *et al*. Last appearance of *Homo erectus* at Ngandong, Java, 117,000–108,000 years ago. *Nature* **577**, 381-385 (2020).

174. Thomsen, K. J., Murray, A. S., Jain, M. & Botter-Jensen, L. Laboratory fading rates of various luminescence signals from feldspar-rich sediment extracts. *Radiat. Meas*. **43**, 1474–1486 (2008).

175. Thiel, C. *et al*. Luminescence dating of the Stratzing loess profile (Austria)—testing the potential of an elevated temperature post-IR IRSL protocol. *Quat. Int.* **234**, 23–31 (2011).

176. Shackelford, L., *et al*. Additional evidence for early modern human morphological diversity in Southeast Asia at Tam Pa Ling, Laos. *Quat. Int.* **466**, 93–106 (2018).

177. Smith, H.E. *et al.* Taxonomy, taphonomy, and age of the Pleistocene faunal assemblage from Ngalau Gupin cave, Sumatra. *Quaternary International* **603**, 40-63 (2021).

178. Duval, M., *et al.* New Chronological Constraints for the Late Pleistocene Fossil Assemblage and Associated Breccia from Ngalau Sampit, Sumatra. *Open Quaternary* **7**, 1–24 (2021).

179. Murray, A. S. & Wintle, A. G. Luminescence dating of quartz using an improved single-aliquot regenerative-dose protocol. *Radiat. Meas*. **32**, 57–73 (2000).

180. Bøtter-Jensen, L., & Mejdahl, V. Assessment of beta dose-rate using a GM multicounter system. International Journal of Radiation Applications and Instrumentation. Part D. *Nuclear Tracks and Radiation Measurements*, **14**, 187-191 (1988).

181. Brennan, B.J. Beta doses to spherical grains. *Radiation Measurements* **37**, 299-303 (2003).

182. Bell, W.T. & Zimmerman, D.W. The effect of HF acid etching on the morphology of quartz inclusions for thermoluminescence dating. *Archaeometry* **20**, 63-65 (1978).

183. Wang, W. & Xia, J. The measurement of annual dose from Th series and U series decay chains by thick source alpha counting. *Nuclear Techniques* **14**, 101-108 (1991).

184. Feathers, J. K. & Migliorini, E. Luminescence dating at Katanda—a reassessment. *Quat. Sci. Rev*. **20**, 961–966 (2001).

185. Huntley, D. J. & Baril, M. R. The K content of the K-feldspars being measured in optical dating or in thermoluminescence dating. *Ancient TL* **15**, 11–13 (1997).

186. Huntley, D. J. & Hancock, R. G. V. The Rb contents of the K-feldspars being measured in optical dating. *Ancient TL* **19**, 43–46 (2001).

187. Rhodes, E.J. Dating sediments using potassium feldspar single-grain IRSL: initial methodological considerations. *Quaternary International* **362**, 14-22 (2015).

188. Buylaert, J. P., Murray, A. S., Thomsen, K. J. & Jain, M. Testing the potential of an elevated temperature IRSL signal from K-feldspar. *Radiat. Meas*. **44**, 560–565 (2009).

189. Murray, A. S., Buylaert, J. P., Thomsen, K. J. & Jain, M. The effect of preheating on the IRSL signal from feldspar. *Radiat. Meas*. **44**, 554–559 (2009).

190. Li, B. & Li, S. A reply to the comments by Thomsen *et al*. on" Luminescence dating of K-feldspar from sediments: a protocol without anomalous fading correction". *Quat. Geochronol* **8**, 49–51 (2012).

191. Jacobs, Z., Duller, G. A. T. & Wintle, A. G. Interpretation of single grain D_e_ distributions and calculation of D_e_. *Radiat. Meas.* **41**, 264–277 (2006).

192. Galbraith, R. F., Roberts, R. G., Laslett, G. M., Yoshida, H., & Olley, J. M. (1999). Optical dating of single and multiple grains of quartz from Jinmium rock shelter, northern Australia: Part I, experimental design and statistical models. *Archaeometry* **41**, 339-364 (1999).

193. Olley, J.M., Murray, A., & Roberts, R.G. The effects of disequilibria in the uranium and thorium decay chains on burial dose rates in fluvial sediments. *Quat Sci Rev* **15**, 751–760 (1996).

194. Woodroffe, C. D., Short, S. A., Stoddart, D. R., Spencer, T. & Harmon R. S., Stratigraphy and chronology of late Pleistocene reefs in the Southern Cook Islands, South Pacific. *Quat. Res.* **35**, 246-263 (1991).

195. Vermeesch, P. IsoplotR: A free and open toolbox for geochronology. *Geoscience Frontiers* **9**, 1479-1493 (2018).

196. Paton, C., Hellstrom, J., Paul, B., Woodhead, J. & Hergt, J. Iolite: Freeware for the visualisation and processing of mass spectrometric data. Journal of Analytical Atomic Spectrometry 26, 2508-2518 (2011).

197. Dosseto, A. & Marwick, B. UThwigl —An R package for closed- and open-system uranium–thorium dating. Quaternary Geochronology 67, 101235 (2022).

198. Grün, R., Aubert, M., Joannes-Boyau, R., & Moncel, M.H. High resolution analysis of uranium and thorium concentration as well as U-series isotope distributions in a Neanderthal tooth from Payre (Ardèche, France) using laser ablation ICP-MS. Quat. Geochron. 72, 5278-5290 (2008).

199. Joannes-Boyau, R., & Grün, R. A comprehensive model for CO2− radicals in fossil tooth enamel: Implications for ESR dating. Quat. Geochron. 6, 82-97 (2011).

200. Joannes-Boyau, R. Detailed protocol for an accurate non-destructive direct dating of tooth enamel fragment using Electron Spin Resonance. Geochronometria 40, 322-333 (2013).

201. Yu, W., Herries, A. I., & Joannes-Boyau, R. Using X-rays as an irradiation source for direct ESR dating of fossil teeth. Quaternary Geochronology 72, 101372 (2022).

202. Joannes-Boyau, R. & Grün, R. Thermal behavior of oriented and non-oriented CO2− radicals in tooth enamel. Rad. Meas. 44, 505-511 (2009).

203. Duval, M. & Grün, R. 2016. Are published ESR dose assessments on fossil tooth enamel reliable? Quaternary Geochronology 31, 19-27 (2016).

204. Grün, R. The DATA program for the calculation of ESR age estimates on tooth enamel. Quaternary Geochronology 4, 231-232 (2009).

205. Cheng, H., Edwards, R.L., Hoff, J., Gallup, C.D., Richards, D.A. & Asmerom, Y. The half-lives of uranium-234 and thorium-230. Chem. Geol. 169, 17–33 (2000).

206. Grün, R. Beta dose attenuation in thin layers. Ancient TL 4, 1-8 (1986).

207. Grün, R. Alpha dose attenuation in thin layers. Ancient TL 5, 6-8 (1987).

208. Grün, R. & Katzenberger-Apel, O. An alpha irradiator for ESR dating. Ancient TL, 12 (1994).

209. Duval, M., & Guilarte Moreno, V. Assessing the influence of the cavity temperature on the ESR signal of Aluminium center in quartz grains extracted from sediment. Ancient TL 30, 51-57 (2012).

210. Guilarte V., & Duval M. ESR dating of optically bleached quartz grains: intra-laboratory comparison of different experimental setups and their impact on dose evaluation. Geochronometria. DOI: 10.2478/geochr-2020-0005 (2020).

211. Toyoda, S. & Falguères, C. The method to represent the ESR signal intensity of the aluminium hole center in quartz for the purpose of dating. Advances in ESR Applications 20, 7-10 (2013).

212. Duval, M. & Guilarte, V. ESR dosimetry of optically bleached quartz grains extracted from Plio-Quaternary sediment: Evaluating some key aspects of the ESR signals associated to the Ti-centers. Radiation Measurements 78, 28-41 (2015).

213. Duval, M. Dose response curve of the ESR signal of Aluminium center in quartz grains extracted from sediment. Ancient TL 30, 41-50 (2012).

214. Duval, M., Grün, R., Falgueres, C., Bahain, J.J. & Dolo, J.M. ESR dating of Lower Pleistocene fossil teeth: limits of the single saturating exponential (SSE) function for the equivalent dose determination. Radiation Measurements 44, 477-482 (2009).

215. Durcan, J.A., King, G.E. & Duller, G.A.T. DRAC: Dose Rate and Age Calculator for trapped charge dating. Quaternary Geochronology 28, 54-61 (2015).

216. Brennan, B.J., Lyons, R.G., & Phillips, S.W. Attenuation of alpha particle track dose for spherical grains. Int. J. Radiat. Appl. Instrum. Part D. Nucl. Tracks Radiat. Meas. 18, 249-253 (1991).

217, Guérin, G., Mercier, N., Nathan, R., Adamiec, C., & Lefrais, Y. On the use of the infinite matrix assumption and associated concepts: a critical review. Radiat. Meas. 47, 778-785 (2012).

218. Duval, M. et al. Quantifying HF etching of quartz and feldspar coarse grains based on weight loss estimates: implication for ESR and Luminescence dating studies. Ancient TL 36, 1-15 (2018).

219. Vandenberghe, D., De Corte, F. Buylaert, J. P. Kučera J. & Van den haute, P. On the internal radioactivity in quartz. Radiation Measurements 43, 771-775 (2008).

220. Duval, M., Sancho, C., Calle, M., Guilarte, V., & Peña-Monné, J.L. On the interest of using the Multiple Center approach in ESR dating of optically bleached quartz grains: some examples from the Early Pleistocene terraces of the Alcanadre River (Ebro basin, Spain). Quaternary Geochronology 29, 58-69 (2015).

221. Duval, M., Arnold, L.J., Guilarte, V., Demuro, M, Santonja, M., & Pérez-González, A. Electron Spin Resonance dating of optically bleached quartz grains from the Middle Palaeolithic site of Cuesta de la Bajada (Spain) using the multiple centres approach. Quaternary Geochronology 37, 82-96 (2017).

222. del Val, M. et al. Luminescence and ESR dating of the multi-level cave system of Alkerdi-Zelaieta (Navarre, N Spain) and implications for provenance study. Quaternary Geochronology 73, 101380 (2022).

223. Bernal, J.P., Eggins, S.M. & McCulloch, M.T. Accurate in situ 238 U–234 U–232 Th–230 Th analysis of silicate glasses and iron oxides by laser-ablation MC-ICP-MS. Journal of Analytical Atomic Spectrometry 20, 1240-1249 (2005).

224. Zheng, Z. & Lei, Z.-Q. A 400,000 year record of vegetational and climatic changes from a volcanic basin, Leizhou Peninsula, southern China. *Palaeogeography, Palaeoclimatology, Palaeoecology* **145**, 339–362 (1999).

225. Aldeias, V., Goldberg, P., Dibble, H. L., & El-Hajraoui, M. Deciphering site formation processes through soil micromorphology at Contrebandiers Cave, Morocco. *Journal of Human Evolution* **69**, 8-30 (2014).

226. Cruz-y-Cruz, T., Sánchez-Miranda, G., Carpenter, J., Terrazas-Mata, A., Sedov, S., Solleiro- Rebolledo, E., & Benavente-Sanvicente, M. E. Pleistocene paleosols associated with megafauna in Northwestern Mexico: Paleoecological inferences. *Spanish Journal of Soil Science* **8**, 130-147 (2018).

227. Goldberg, P., & Berna, F. Micromorphology and context. *Quaternary International* **214**, 56-62. (2010).

228. Karkanas, P., & Goldberg, P. Site formation processes at Pinnacle Point Cave 13B (Mossel Bay, Western Cape Province, South Africa): Resolving stratigraphic and depositional complexities with micromorphology. *Journal of Human Evolution* **59**, 256-273 (2010).

229. Macphail, R. I., Courty, M.-A., & Goldberg, P. Soil micromorphology in archaeology. *Endeavour* **14**, 163-171 (1990).

230. McAdams, C., *et al.* The Pleistocene geoarchaeology and geochronology of Con Moong Cave, North Vietnam: Site formation processes and hominin activity in the humid tropics. *Geoarchaeology* **35**, 72-97. (2020).

231. Morley, M. W., *et al*. Initial micromorphological results from Liang Bua, Flores (Indonesia): Site formation processes and hominin activities at the type locality of *Homo floresiensis*. *Journal of Archaeological Science* **77**, 125-142 (2017).

232. Kalinski, M. E. Soil Mechanics Lab Manual. 2nd Edition 1–208 (Wiley Global Education, 2011).

233. Syvitski, J.P.N. Principles, Methods and Application of Particle Size Analysis. 1–368, (Cambridge University Press, Cambridge, UK, 2007).

234. Moore, D. M. & Reynolds, R. C. X-Ray Diffraction and the identification and analysis of clay minerals 1-378. 2nd Edition. (Oxford University Press, New York, USA, 1997).

235. Henderson, G.S., Neuville, D. R., & Downs, R. T. (Ed.) Spectroscopic methods in mineralogy and materials sciences. Reviews in Mineralogy and Geochemistry 78, Mineralogical Society of America, 1–818 (Washington, USA, 2014).

236. Morley, M.W. & Goldberg, P. Geoarchaeological research in the humid tropics: A global perspective. *Journal of Archaeological Science* **77**, 1–9 (2017).

237. Estévez, J., Villagran, X. S., Balbo, A. L., & Hardy, K. Microtaphonomy in archaeological sites: The use of soil micromorphology to better understand bone taphonomy in archaeological contexts. *Quaternary International* **330**, 3–9 (2014).

238. Goldberg, P. & Macphail, R. I. Practical and theoretical geoarchaeology. Chapter 16. Laboratory techniques, 335-367. (Blackwell Science Ltd., Oxford, UK, 2006)

239. Karkanas, P. & Goldberg, P. Phosphatic features. In: Interpretation of micromorphological features of soils and regoliths, (Eds.) G. Stoops, V. Marcelino, F. Mees, Elsevier B.V., 521–541 (Amsterdam, The Netherlands, 2010).

240. Nicosia, C., & Stoops, G. (Eds.). *Archaeological Soil and Sediment Micromorphology*. 1–476 (John Wiley and Sons Hoboken, NJ, USA, 2017).

241. Morley, M. W., Goldberg, P., Uliyanov, V. A., Kozlikin, M. B., Shunkov, M. V., Derevianko, A. P., Jacobs, Z., & Roberts, R. G. Hominin and animal activities in the microstratigraphic record from Denisova Cave (Altai Mountains, Russia). *Nature Scientific Reports* **9**, 1-12 (2019).

242. Stephens, M., Rose, J., & Gilbertson, D. D. Post-depositional alteration of humid tropical cave sediments: Micromorphological research in the Great Cave of Niah, Sarawak, Borneo. *Journal of Archaeological Science* **77**, 109-124 (2017).

243. Stephens, M., Rose, J., Gilbertson, D. D. & Canti, M. G. Micromorphology of Cave Sediments in the Humid Tropics: Niah Cave, Sarawak. *Asian Perspectives* **44**, 42-55 (2005).

244. Dunham, R.J. Classification of Carbonate Rocks According to Depositional Texture. In: Classification of Carbonate Rocks, Ham, W.E. (Ed.), 108–121 (American Association Petroleum Geologists, Tulsa, USA., 1962).

245. Folk, R.L. Practical Petrographic Classification of Limestones. American Association *Petroleum Geologists Bulletin* **43**, 1–38 (1959).

246. Munsell Color Co., Inc. Munsell Soil Color Charts (revised ed.) 1–22 (Baltimore, USA. 1994).

247. Arpin, T. L., Mallol, C., & Goldberg, P. Short contribution: A new method of analyzing and documenting micromorphological thin sections using flatbed scanners: Applications in geoarchaeological studies. *Geoarchaeology* **17**, 305–313 (2002).

248. Soukup, D. A., Buck, B. J., & Harris, W. Preparing Soils for Mineralogical Analyses. In: Methods of Soil Analysis, Part 5. Mineralogical Methods. Soil Science Society of America, 5, 1-19 (Madison, Wisconsin, USA, 2008).

249. Blott, S. J., & Pye, K. GRADISTAT: A grain size distribution and statistics package for the analysis of unconsolidated sediments. *Earth Surface Processes and Landforms* **26**, 1237–1248 (2001).

250. Heiri, O., Lotter, A. F., & Lemcke, G. Loss on ignition as a method for estimating organic and carbonate content in sediments: reproducibility and comparability of results. *Journal of Paleolimnology* **25**, 101–110 (2001).

251. Miller, W. R. Influence of Rock Composition on the Geochemistry of Stream and Spring Waters from Mountainous Watersheds in the Gunnison, Uncompahgre, and Grand Mesa National Forests, Colorado. *US Geological Survey* **1667**, 1-59 (2002).

252. Roden, E. & Edmonds, J. Phosphate mobilization in iron-rich anaerobic sediments: Microbial Fe (III) oxide reduction versus iron-sulfide formation. *Archiv für Hydrobiologie* **139**, 347–378 (1997).

253. Carter, D. O., Metcalf, J. L., Bibat, A., & Knight, R. Seasonal variation of postmortem microbial communities. *Forensic Science, Medicine, and Pathology* **11**, 202–207 (2015).

254. Jans, M. M. E., Nielsen-Marsh, C. M., Smith, C. I., Collins, M. J., & Kars, H. Characterisation of microbial attack on archaeological bone. *Journal of Archaeological Science* **31**, 87–95 (2004).

255. McAdams C., Morley M.W., & Roberts R.G. The acid test: An experimental microarchaeological study of guano driven diagenesis in tropical cave sediments. *Journal of Archaeological Science: Reports* **37**, 102947 (2021).

256. Audra, P., *et al*. Bat guano minerals and mineralization processes in Chameau Cave, Eastern Morocco. *International Journal of Speleology* **50**, 91–109 (2021).

257. Bird, M.I., Boobyer, E.M., Bryant, C., Lewis, H.A., Paz, V., & Stephens, W.E. A long record of environmental change from bat guano deposits in Makangit Cave, Palawan, Philippines. *Earth and Environmental Science Transactions of the Royal Society of Edinburgh* **98**, 59–69 (2007).

258. Wurster, C.M., Munksgaard, N., Zwart, C., & Bird, M. The biogeochemistry of insectivorous cave guano: A case study from insular Southeast Asia. *Biogeochemistry* **124**, 163–175 (2015).

259. Karkanas, P., Bar-Yosef, O., Goldberg, P., & Weiner, S. Diagenesis in prehistoric caves: The use of minerals that form in-situ to assess the completeness of the archaeological record. *Journal of Archaeological Science* **27**, 915–929 (2000).

260. Shahack-Gross, R., Berna, F., Karkanas, P., & Weiner, S. Bat guano and preservation of archaeological remains in cave sites. *Journal of Archaeological Science* 31, 1259–1272 (2004).

261. Jones, B. D., & Ingle, J. D. Evaluation of redox indicators for determining sulfate-reducing and dechlorinating conditions. *Water Research* **39**, 4343–4354 (2005).

262. Gunal, H., & Ransom, M. D. Clay illuviation and calcium carbonate accumulation along a precipitation gradient in Kansas. *CATENA* **68**, 59–69 (2006).

263. Lee-Thorp, J. *et al*. Isotopic evidence for an early shift to C4 resources by Pliocene hominins in Chad. *Proc. Natl Acad. Sci. USA* **109**, 20369–20372 (2012).

264. Roberts, P. *et al*. Fruits of the forest: Human stable isotope ecology and rainforest adaptations in Late Pleistocene and Holocene (<36 to 3 ka) Sri Lanka. *J. Hum. Evol*. **106**, 102–118 (2017).

265. Snoeck, C. & Pellegrini, M. Comparing bioapatite carbonate pre-treatments for isotopic measurements: part 1 – impact on structure and chemical composition. *Chem. Geol.* **417**, 394–403 (2015).

266. Pellegrini, M. & Snoeck, C. Comparing bioapatite carbonate pre-treatments for isotopic measurements: part 2 – impact on carbon and oxygen isotope compositions. *Chem. Geol.* **420**, 88–96 (2016).

267. Jiang, Q. Y., Zhao, L. X. & Hu, Y. W. Variations of fossil enamel bioapatite caused by different preparation and measurement protocols: a case study of *Gigantopithecus* fauna. *Vertebrata PalAsiatica* **58**, 159–168 (2020).

268. Craig, H. The geochemistry of the stable carbon isotope. *Geochim. Cosmochim. Acta* **3**, 53–92 (1953).

269. Smith, B. N. & Epstein, S. Two categories of ^13^C/^12^C ratios for higher plants. *Plant Physiol.* **47**, 380–384 (1971).

270. Tieszen, L. L. Natural variations in the carbon isotope values of plants: implications for archaeology, ecology, and paleoecology. *J. Archaeol. Sci.* **18**, 227–248 (1991).

271. Sponheimer, M. *et al*. Do “savanna” chimpanzees consume C4 resources? *J. Hum. Evol.* **51**, 128–133 (2006).

272. Sponheimer, M. *et al*. Isotopic evidence of early hominin diets. *Proc. Natl Acad. Sci. USA* **110**, 10513–10518 (2013).

273. Codron, J. *et al*. Stable isotope series from elephant ivory reveal lifetime histories of a true dietary generalist. *Proc. R. Soc. Lond. B* **279**, 2433–2441 (2012).

274. Crowley, B. E. *et al*. Extinction and ecology retreat in a community of primates. *Proc. R. Soc. Lond. B* **279**, 3597–3605 (2012).

275. Farquhar, G. D., Ehleringer, J. R. & Hubick, K. T. Carbon isotope discrimination and photosynthesis. Annu. Rev. *Plant Physiol. Plant Mol. Biol.* **40**, 503–537 (1989).

276. van der Merwe, N. J. & Medina, E. The canopy effect, carbon isotope ratios and foodwebs in Amazonia. *J. Archaeol. Sci.* **18**, 249–259 (1991).

277. Pearcy, R. W. & Pfitsch, W. A. Influence of sunflecks on the δ13C of Adenocaulon bicolor plants occurring in contrasting forest understory microsites. *Oecologia* **86**, 457–462 (1991).

278. Bonafini, M., Pellegrini, M., Ditchfield, P. & Pollard A. M. Investigation of the ‘canopy effect’ in the isotope ecology of temperate woodlands. *J. Archaeol. Sci*. **40**, 3926–3935 (2013).

279. Ehleringer, J. R., Rundel, P. W. & Nagy, K. A. Stable isotopes in physiological ecology and food web research. *Trends Ecol. Evol*. **1**, 42–45 (1986).

280. van der Merwe, N. J. & Medina, E. Photosynthesis and 13C/12C ratios in Amazonian rainforests. Geochim. Cosmochim. *Acta* **53**, 1091–1094 (1989).

281. Ometto, J. P. H. B. *et al*. The stable carbon and nitrogen isotopic composition of vegetation in tropical forests of the Amazon Basin, Brazil. *Biogeochemistry* **79**, 251–274 (2006).

282. Gonfiantini, R., Gratziu, S. & Tongiorgi, E. in Isotopes and Radiation in Soil Plant Nutrition Studies (Technical Report Series No. 206) (ed. Joint FAO/IAEA Division of Atomic Energy in Agriculture) 405–410 (Isotope Atomic Energy Commission, 1965).

283. Lee-Thorp, J. A., Sealy, J. C. & van der Merwe, N. J. Stable carbon isotope ratio differences between bone collagen and bone apatite, and their relationship to diet. *J. Archaeol. Sci.* **16**, 585–599 (1989).

284. Flanagan, L. B., Comstock, J. P. & Ehleringer, J. R. Comparison of modelled and observed environmental influences on the stable oxygen and hydrogen isotope composition of leaf water in Phaseolus vulgaris *L. Plant Physiol*. **96**, 588–596 (1991).

285. Yakir, D., Berry, J. A., Giles, L. & Osmond, C. B. Isotopic heterogeneity of water in transpiring leaves: Identification of the component that controls the δ^18^O of atmospheric O_2_ and CO_2_. *Plant Cell Environ*. **17**, 73–80 (1994).

286. Sheshshayee, M. S. *et al*. Oxygen isotope enrichment (Δ18O) as a measure of time-averaged transpiration rate. *J. Exp. Bot*. **56**, 3033–3039 (2005).

287. Buchmann, N. & Ehleringer, J. R. CO_2_ concentration profiles, and carbon and oxygen isotopes in C3 and C4 crop canopies. *Agric. For. Meteorol*. **89**, 45–58 (1998).

288. Buchmann, N., Guehl, J. M., Barigah, T. S. & Ehleringer, J. R. Interseasonal comparison of CO2 concentrations, isotopic composition, and carbon dynamics in an Amazonian rainforest (French Guiana). *Oecologia* **110**, 120–131 (1997).

289. da Silveira, L., Sternberg, L., Mulkey, S. S. & Joseph Wright, S. Oxygen isotope ratio stratification in a tropical moist forest. *Oecologia* **81**, 51–56 (1989).

290. McCarroll, D. & Loader, N. J. in Isotopes in Palaeonvironmental Research (ed. Leng, M. J.) 67–116 (Springer, 2006).

291. Carter, M. L. & Bradbury, M. W. Oxygen isotope ratios in primate bone carbonate reflect amount of leaves and vertical stratification in the diet. *Am. J. Primatol.* **78**, 1086–1097 (2016).

292. Kohn, M. J., Schoeninger, M. J. & Valley, J. W. Herbivore tooth oxygen isotope compositions: effects of diet and physiology. *Geochim. Cosmochim. Acta* **60**, 3889–3896 (1996).

293. Levin, N. E., Cerling, T. E., Passey, B. H., Harris, J. M. & Ehleringer, J. R. A stable isotope aridity index for terrestrial environments. *Proc. Natl Acad. Sci. USA* **103**, 11201–11205 (2006).

294. Fannin, L. D. & McGraw, W. S. Does oxygen stable isotope composition in primates vary as a function of vertical stratification or folivorous behaviour? *Folia Primatol.* **91**, 219–227 (2020).

295. Oelze, V.M., *et al.* Exploring the contribution and significance of animal protein in the diet of bonobos by stable isotope ratio analysis of hair. *Proceedings of the National Academy of Sciences* **108**, 9792-9797 (2011).

296. Roberts, P., *et al*. Fruits of the forest: human stable isotope ecology and rainforest adaptations in Late Pleistocene and Holocene (∼ 36 to 3 ka) Sri Lanka. *Journal of human evolution* **106**, 102-118 (2017).

297. Bocherens, H., Drucker, D.G. & Madelaine, S. Evidence for a ^15^N positive excursion in terrestrial foodwebs at the Middle to Upper Palaeolithic transition in south-western France: Implications for early modern human palaeodiet and palaeoenvironment. *Journal of Human Evolution* **69**, 31-43 (2014).

298. Willmes, M., *et al*. Improvement of laser ablation in situ micro-analysis to identify diagenetic alteration and measure strontium isotope ratios in fossil human teeth. Journal of Archaeological *Science* **70**, 102–116 (2016).

299. Ungar PS, & Sponheimer M. The diets of early hominins. *Science* **334**, 190-3 (2011).

300. Sponheimer M, & Lee-Thorp J.A. Isotopic evidence for the diet of an early hominid, *Australopithecus africanus*. *Science* **283**, 368-70 (1999).

301. Austin, C., *et al*. Barium distributions in teeth reveal early-life dietary transitions in primates. *Nature* **498**, 216–219 (2013).

302. Smith, T., *et al*. Wintertime stress, nursing and lead exposure in Neanderthal children. *Science Advances* **4**, eaau9483 (2018).

303. Kendall, C., Eriksen, A.M.H., Kontopoulos, I., Collins, M.J., & Turner-Walker, G. Diagenesis of archaeological bone and tooth. *Palaeogeography palaeoclimatology palaeoecology* **491**, 21-37 (2018).

304. Chow, M. Mammalian faunas and correlation of Tertiary and Early Pleistocene of South China. *Journal of the Paleontological Society of India* **3**, 123–130 (1957).

305. Dickson, P. *Gigantopithecus:* A Reappraisal of Dietary Habits. *Journal of Anthropology* **11**, 28–35 (2003).

306. Livingstone, F. B. Controversy regarding *Gigantopithecus*. *American Anthropologist*, **67**, 1283–1284 (1965).

307. Jiang, Q., Zhao, L., Guo, L., & Hu, Y. First direct evidence of conservative foraging ecology of early *Gigantopithecus blacki* (~2 Ma) in Guangxi, southern China. *American Journal of Physical Anthropology* **176**, 93–108 (2021).

308. De Vos, J., & Long, V.T. Systematic discussion of the Lang Trang fauna. (Unpublished report, 1993).

309. Simons, E. L. Primate evolution: an introduction to man's place in nature. 1–322 (MacMillan, New York, USA, 1972).

310. DeSantis, L.R.G., *et al*. Direct comparisons of 2D and 3D Dental Microwear Proxies in Extant Herbivorous and Carnivorous Mammals. *Public Library of Science One* (PLOS ONE), **8**, 1-11 (2013).

311. Merceron, G., Schulz, E., Kordos, L., & Kaiser, T. M. Paleoenvironment of Dryopithecus brancoi at Rudabánya, Hungary: Evidence from dental meso- and micro-wear analyses of large vegetarian mammals. *Journal of Human Evolution* **53**, 331-349 (2007).

312. Scott, R. S., *et al.* Dental microwear texture analysis shows within-species diet variability in fossil hominins. *Nature* **436**, 693-695 (2005).

313. Scott, R. S., *et al*. Dental microwear texture analysis: Technical considerations. *Journal of Human Evolution* **51***,* 339-349 (2006).

314. Ungar, P.S., Brown, C.A., Bergstrom, T., & Walker, A. Quantification of Dental Microwear by Tandem Scanning Confocal Microscopy and Scale-Sensitive Fractal Analyses. *Scanning* **25**, 185-193 (2003).

315. Scott, R. S., Teaford, M. F., & Ungar, P. S. Dental microwear texture and anthropoid diets. *American Journal of Physical Anthropology* **147***,* 551-579 (2012).

316. Ungar, P.S., Scott, R.S., Scott, J.S., & Teaford, M. 17 Dental microwear analysis: historical perspectives and new approaches. *Technique and Application in Dental Anthropology* **53**, 389 (2008).

317. Grine, F. E. Dental evidence for dietary differences in *Australopithecus* and *Paranthropus:* A quantitative analysis of permanent molar microwear. *Journal of Human Evolution* **15**, 783–822 (1986).

318. Teaford, M. F., & Oyen, O. J. In vivo and in vitro turnover in dental microwear. *American Journal of Physical Anthropology* **80**, 447–460 (1989).

319. Winkler, D. E., *et al*. The turnover of dental microwear texture: Testing the” last supper” effect in small mammals in a controlled feeding experiment. *Palaeogeography, Palaeoclimatology, Palaeoecology* **557**, 109930 (2020).

320. Gordon, K. D. A study of microwear on chimpanzee molars: Implications for dental microwear analysis. *American Journal of Physical Anthropology* **59**,195–215 (1982).

321. King, T., Andrews, P., & Boz, B. Effect of taphonomic processes on dental microwear. *American Journal of Physical Anthropology* **108**, 359–373 (1999).

322. Teaford, M. F. Dental microwear and dental function. *Evolutionary Anthropology: Issues, News, and Reviews* **3**, 17–30 (1994).

323. Teaford, M. F., & Glander, K. E. Dental microwear in live, wild‐trapped Alouatta palliata from Costa Rica. *American Journal of Physical Anthropology* **85**, 313–319 (1991)

324. Teaford, M.F., & Glander, K.E. Dental Microwear and Diet in a Wild Population of Mantled Howling Monkeys (Alouatta palliata). In: Adaptive Radiations of Neotropical Primates, Norconk, M.A., Rosenberger, A.L., Garber, P.A. (Eds.). (Springer, Boston, Massachusetts, USA, 1996).

325. Teaford, M. F., & Tylenda, C. A. A New Approach to the Study of Tooth Wear. *Journal of Dental Research* **70**, 204–207 (1991).

326. Ungar, P. S., & Teaford, M. F. Preliminary examination of non‐occlusal dental microwear in anthropoids: implications for the study in fossil primates. *American Journal of Physical Anthropology* **100**, 101–113 (1996).

327. Strani, F., Profico, A., Manzi, G., Pushkina, D., Raia, P., Sardella, R., & DeMiguel, D. MicroWeaR: A new R package for dental microwear analysis. *Ecology and Evolution* **8**, 7022-7030 (2018).

328. Gordon, K. D. The assessment of jaw movement direction from dental microwear. *American Journal of Physical Anthropology* **63**, 77–84 (1984).

329, Kullmer, O., Benazzi, S., Fiorenza, L., Schulz, D., Bacso, S., & Winzen, O. Technical note: Occlusal Fingerprint Analysis: Quantification of tooth wear pattern. *American Journal of Physical Anthropology* **139***,* 600–605 (2009).

330. Kullmer, O. Menz, U., & Fiorenza, L. Occlusal Fingerprint Analysis (OFA) reveals dental occlusal behavior in primate molars. In: *Mammalian Teeth – Form and Function*, (Eds.) Martin, T. and Von Koenigswald. Pfeil, Dr. Friedrich 25–43, (München, Germany, 2020).

331. Kullmer, O., Schulz, D., & Benazzi, S. An Experimental Approach to Evaluate the Correspondence Between Wear Facet Position and Occlusal Movements. *The Anatomical Record: Advances in Integrative Anatomy and Evolutionary Biology* **295***,* 846–852 (2012).

332. Stuhlträger, J., et al. Dental wear patterns reveal dietary ecology and season of death in a historical chimpanzee population. *PLOS ONE*, **16***,* 1–18 (2021).

333. Von Koenigswald, W., Anders, U., Engels, S., Schultz, J. A., & Kullmer, O. Jaw movement in fossil mammals: Analysis, description and visualization. *Paläontologische Zeitschrift* **87***,* 141–159 (2013).

334. Smith, B. H. Patterns of molar wear in hunter-gatherers and agriculturalists. *American Journal of Physical Anthropology* **63**, 39–56 (1984).

335. Harrison, T., Jin, C., Zhang, Y., Wang, Y., & Zhu, M. Fossil *Pongo* from the Early Pleistocene *Gigantopithecus* fauna of Chongzuo, Guangxi, southern China. *Quaternary International* **354**, 59–67 (2014).

336. Merceron, G., Schulz, E., Kordos, L., & Kaiser, T. M. Paleoenvironment of *Dryopithecus brancoi* at Rudabánya, Hungary: Evidence from dental meso- and micro-wear analyses of large vegetarian mammals. *Journal of Human Evolution* **53***,* 331–349 (2007).

337. Keselman, H.J., *et al*. Statistical practices of educational researchers: an analysis of their ANOVA, MANOVA, and ANCOVA analyses. *Review of Educational Research* **68**, 350-386 (1998).

338. Wilcox, R.R. Applying Contemporary Statistical Techniques. (Academic Press, San Diego, 2023).

339. Wilcox, R.R. Introduction to Robust Estimation and Hypothesis Testing (Second Ed.) (Elsevier Academic Press, Burlington, San Diego, London, 2005)

340. Hohmann, G. The Diets of Non-human Primates: Frugivory, Food Processing, and Food Sharing. In: The evolution of hominin diets: Integrating approaches to the study of Palaeolithic subsistence, Hublin, J.-J., and Richards, M. (Eds.), 1-14, (Springer Science, 2009).

341. Russon, A.E., *et al*. Geographic variation in orangutan diets. In: Orangutans. Geographic Variation in Behavioral Ecology and Conservation, Wich, S.A., Setia, T.M., van Schaik, C.P. (Eds.) 135–156 (Oxford University Press, Oxford, UK, 2009).

342. Vogel, E. R., *et al*. Food mechanical properties, feeding ecology, and the mandibular morphology of wild orangutans. *Journal of Human Evolution* **75**, 110–124 (2014).

343. Vogel, E. R., *et al*. Nutritional Differences between Two Orangutan Habitats: Implications for Population Density. *PLOS ONE* **10**, 2–18 (2015).

344. Delgado, R. A., & Van Schaik, C. P. The behavioural ecology and conservation of the orangutan (*Pongo pygmaeus*): A tale of two islands. *Evolutionary Anthropology: Issues, News, and Reviews* **9**, 201–218 (2000).

345. Hardus, M.E., *et al.* Behavioral, ecological, and evolutionary aspects of meat-eating by Sumatran orangutans (*Pongo abelii*). *International Journal of Primatology* **33**, 287–304 (2012).

346. Blanchard, R., Mitchel, D. R., Klinkhamer, A., & Fiorenza, L. Functional molar macrowear analysis in *Pongo pygmaeus* and *Pongo abelii*. Abstract from Annual Meeting of the European Society for the study of Human Evolution (ESHE 2019) (Liege, Belgium, 2019).

347. Knight-Sadler, J., & Fiorenza, L. Tooth Wear Inclination in Great Ape Molars. *Folia Primatologica*, **88**, 223–236 (2017).

348. Lucas, P.W. Dental Functional Morphology: How Teeth Work. 1–372 (New York: Cambridge University Press, New York, USA, 2004).

349. Lucas, P.W. *et al*. Dental enamel as a dietary indicator in mammals. *Bioessays* **30**, 374–358 (2008).

350. Taylor, A. B. Feeding behaviour, diet, and the functional consequences of jaw form in orangutans, with implications for the evolution of Pongo. *Journal of Human Evolution* **50**, 377–393 (2006).

351. Taylor, A. B. Masticatory form and function in the African apes. *American Journal of Physical Anthropology* **117**, 133–156 (2002).

352. Lubeek, J. K. Dental macrowear and mandibular morphology as proxies for mastication of *Gigantopithecus blacki*: how the ‘King Kong’ cousin of early Homo in Palaeolithic Southeast Asia chewed itself to extinction. (Master Thesis, Leiden University. Leiden, The Netherlands, 2017).

353. Doran-Sheehy, D., Mongo, P., Lodwick, J., & Conklin-Brittain, N. L. Male and female western gorilla diet: Preferred foods, use of fall-back resources, and implications for ape versus old world monkey foraging strategies. *American Journal of Physical Anthropology* **140**, 727–738 (2009).

354. Galbany, J., *et al*. Tooth wear and feeding ecology in mountain gorillas from Volcanoes National Park, Rwanda. *American Journal of Physical Anthropology* **159**, 457–465 (2016).

355. Galbany, J., *et al*. Age-Related Tooth Wear Differs between Forest and Savanna Primates. *PLOS ONE* **9**, 1–7 (2014).

356. Ostrofsky, K. R., & Robbins, M. M. Fruit-feeding and activity patterns of mountain gorillas (Gorilla *beringei beringei*) in Bwindi Impenetrable National Park, Uganda. *American Journal of Physical Anthropology* **173**, 3–20 (2020).

357. Rogers, M. E., *et al*. Western gorilla diet: A synthesis from six sites. *American Journal of Primatology* **64**, 173–192 (2004).

358. Drawhorn, G.M. The Systematics and Paleodemography of Fossil Orangutans (Genus Pongo). (University of California Davis. PhD Dissertation, 1995.

359. Louys, J. & Meijaard, E. Palaeoecology of Southeast Asian megafauna-bearing sites from the Pleistocene and a review of environmental changes in the region. *Journal of Biogeography* **37**, 1432–1449 (2010).

360. Westaway, K. E., *et al*. Age and biostratigraphic significance of the Punung Rainforest Fauna, East Java, Indonesia, and implications for *Pongo* and *Homo*. *Journal of Human Evolution* **53**, 709–717 (2017).

361. Li, D., *et al.* The stable isotope record in cervid tooth enamel from Tantang Cave, Guangxi: Implications for the Quaternary East Asian monsoon. *Quaternary International* **434**, 156–162 (2017).

362. Wang, Y. *et al*. Millennial- and orbital-scale changes in the East Asian monsoon over the past 224,000 years. *Nature* **451**, 1090–1093 (2008).

363. Spehar, S. N. *et al*. Orangutans venture out of the rainforest and into the Anthropocene. *Science Advances* **4**, e1701422 (2018).

364. Kanamori, T., Kuze, N., Bernard, H., Malim, T.P., & Kohshima, S. Feeding ecology of Bornean orangutans (*Pongo pygmaeus morio*) in Danum Valley, Sabah, Malaysia: a 3-year record including two mast fruitings. *American Journal of Primatology*, **72**, 820–840 (2010).

365. Knott, C.D. Changes in orangutan caloric intake, energy balance, and ketones in response to fluctuating fruit availability. *International Journal of Primatolology* **19**, 1061–1079 (1998).

366. Louys, J., Curnoe, D., & Tong, H. Characteristics of Pleistocene megafauna extinctions in Southeast Asia. *Palaeogeography, Palaeoclimatology, Palaeoecology* **243**, 152–173 (2007).

367. Ashton, P.S., Givnish, T.J., & Appanah, S. Staggered flowering in the Dipterocarpaceae: new insights into floral induction and the evolution of mast fruiting in the seasonal tropics. *The American Naturalist* **132**, 44 –66 (1988).

368. Newbery, D.M., Chuyong, G.B., & Zimmermann, L. Mast fruiting of large ectomycorrhizal African rain forest trees: importance of dry season intensity, and the resource-limitation hypothesis. *New Phytology* **170**, 561–579 (2006).

369. Wich, S.A., & Van Schaik, C.V. The impact of El Nino on mast fruiting in Sumatra and elsewhere in Malesia. *Journal of Tropical Ecology* **16**, 563–577 (2000).
[truncated: 3,313 more chars]
