# Supplementary material for: The demise of the giant ape Gigantopithecus blacki
Source: Nature. 2024 Jan 10;625(7995):535–9. doi: 10.1038/s41586-023-06900-0 (PMC10794149; doi:10.1038/s41586-023-06900-0)
Supplement: Supplementary file 2 — Reporting Summary [file 41586_2023_6900_MOESM2_ESM.pdf]

nature portfolio

Corresponding author(s): Westaway and Zhang

Last updated by author(s): Oct 26, 2023

Reporting Summary

Nature Portfolio wishes to improve the reproducibility of the work that we publish. This form provides structure for consistency and transparency in reporting. For further information on Nature Portfolio policies, see our Editorial Policies and the Editorial Policy Checklist.

Statistics

For all statistical analyses, confirm that the following items are present in the figure legend, table legend, main text, or Methods section.

n/a	Confirmed	
☐	☒	The exact sample size (*n*) for each experimental group/condition, given as a discrete number and unit of measurement
☐	☒	A statement on whether measurements were taken from distinct samples or whether the same sample was measured repeatedly
☐	☒	The statistical test(s) used AND whether they are one- or two-sided<br>*Only common tests should be described solely by name; describe more complex techniques in the Methods section.*
☒	☐	A description of all covariates tested
☐	☒	A description of any assumptions or corrections, such as tests of normality and adjustment for multiple comparisons
☐	☒	A full description of the statistical parameters including central tendency (e.g. means) or other basic estimates (e.g. regression coefficient) AND variation (e.g. standard deviation) or associated estimates of uncertainty (e.g. confidence intervals)
☐	☒	For null hypothesis testing, the test statistic (e.g. *F*, *t*, *r*) with confidence intervals, effect sizes, degrees of freedom and *P* value noted<br>*Give P values as exact values whenever suitable.*
☐	☒	For Bayesian analysis, information on the choice of priors and Markov chain Monte Carlo settings
☒	☐	For hierarchical and complex designs, identification of the appropriate level for tests and full reporting of outcomes
☐	☒	Estimates of effect sizes (e.g. Cohen's *d*, Pearson's *r*), indicating how they were calculated

Our web collection on statistics for biologists contains articles on many of the points above.

Software and code

Policy information about availability of computer code

Data collection	All software used for data collection is commercially available or described in the published literature (citations included in supplementary section). Software used includes: MCDoseE 2.0 (for ESR dating), Riso Sequence Pro (4.59) and DRAC v.1.2 (for ESR and OSL dating)
Data analysis	All software used for data collection is commercially available or described in the published literature with citations provided in the methods and supplementary information. Software used includes: Isoplot EX 3.75 (U-series), MATHLAB USESR (US-ESR), OxCal (version 4.2) (Bayesian modeling), Riso Analyst (4.57), Australasian Pollen and Spore Atlas (ANU), SensoMAP Premium 8.2.9564, Minitab (19.2020.1) R Studio (1.4.1717), IUCN habitats classification scheme (Ver. 3.1), Metropolis-Hastings algorithm,coupled US-ESR, DATA programs, Microcal OriginPro 8.5 software using a Levenberg-Marquardt algorithm, R package UThwigl, shinyImaging (for elemental mapping).

For manuscripts utilizing custom algorithms or software that are central to the research but not yet described in published literature, software must be made available to editors and reviewers. We strongly encourage code deposition in a community repository (e.g. GitHub). See the Nature Portfolio guidelines for submitting code & software for further information.

Data

Policy information about availability of data

All manuscripts must include a data availability statement. This statement should provide the following information, where applicable:

- Accession codes, unique identifiers, or web links for publicly available datasets
- A description of any restrictions on data availability
- For clinical datasets or third party data, please ensure that the statement adheres to our policy

The data that support the findings of this study are included in the Supplementary Information. Additional data, including data sets and raw data have been placed in two Zenobo data repositories and are publicly available; Dating; 10.5281/zenodo.10080908, Environment and behaviour; 10.5281/zenodo.10080973. In addition, Custom code for Ox-cal program have been deposited in Zenodo DOI: 10.5281/zenodo.10077255. There are no restrictions on data availability

Research involving human participants, their data, or biological material

Policy information about studies with human participants or human data. See also policy information about sex, gender (identity/presentation), and sexual orientation and race, ethnicity and racism.

Reporting on sex and gender	N/A
Reporting on race, ethnicity, or other socially relevant groupings	N/A
Population characteristics	N/A
Recruitment	N/A
Ethics oversight	N/A

Note that full information on the approval of the study protocol must also be provided in the manuscript.

Field-specific reporting

Please select the one below that is the best fit for your research. If you are not sure, read the appropriate sections before making your selection.

☐ Life sciences ☐ Behavioural & social sciences ☒ Ecological, evolutionary & environmental sciences

For a reference copy of the document with all sections, see nature.com/documents/nr-reporting-summary-flat.pdf

Ecological, evolutionary & environmental sciences study design

All studies must disclose on these points even when the disclosure is negative.

Study description	To determine the timing and potential cause/s of extinction of Gigantopithecus blacki we incorporated three interrelated components; chronology, past environments and behaviour using six dating techniques and eight proxy lines of evidence
Research sample	Primarily we sampled and analysed G. Blacki fossil teeth as evidence of their presence for dating, past environment reconstruction and behavioural analysis. In addition, as we were limited by the number of G. blacki fossils we also identified and analysed faunal teeth and bones from all 22 excavated sites for comparative purposes particularly Pongo as a useful extant species. We also used materials for dating including sediments, cave breccias, speleothem, teeth and bone.
Sampling strategy	To establish a statistically significant sample size we selected 22 caves across two known G. blacki regions in southern China; Chongzuo and Bubing Basin. The caves were selected based on the presence of fossil bearing breccia and the relative height on the karst plain. We sampled all 11 known G. blacki caves as this represents the extent of our knowledge of G blacki material in these areas. We sampled widely across these caves and collected material for six different dating techniques resulting in 157 radiometric ages, including the dating and analysis of 22 G. blacki teeth. Sediment sample locations were chosen based on proximity to the fossils. To constrain the fossil assemblage we conducted 52 direct dating age estimates of the bone and teeth from all 22 caves. Samples were chosen according to the weathering state of the enamel/dentine or collagen. Particular emphasis was placed on dense, thick bones without cortical sections. Such bones come closest to conform to the general assumption of the diffusion adsorption model.
Data collection	The 22 caves were excavated by the IVPP (Chongzuo) and NHMG (Bubing Basin) from 2000-2019, who recorded all data pertaining to the excavation and catalogued all fossils obtained. All data pertaining to the dating techniques were collected by the appropriate dating specialists during fieldwork between 2017-19 and during laboratory analysis from 2017-2019.
Timing and spatial scale	Sampling of the caves for dating was conducted after excavation (see above) but occurred in collaboration with the original excavating team to ensure the association between the fossils and datable material.

Data exclusions	Samples were excluded if they failed tests for each dating method. These tests and exclusion criteria are outlined for each dating method in the Methods Summary and Supplementary Information. Fragmentary fossils that could not be identified were excluded from analysis for the excavations. For the US dating of two teeth samples were excluded from the dating as no enamel layer offered a uranium concentration <5 ppm, which has been described as the maximum acceptable concentration within the enamel layer to obtain a reliable equivalent dose. For the luminescence dating single-aliquots were rejected according to a rejection criteria devised by cited reference.
Reproducibility	Multiple samples (157) and dating methods (six) were used to determine a chronology for the G. blacki evidence. For both the sedimentological and fossil context independent ages estimates were employed to guarantee that the results are reliable and reproducible. Age estimates are consistent and stratigraphically correct between samples.
Randomization	Randomization was used in the Monte Carlo simulations to estimate the fit of the OSL data in Analyst
Blinding	Each dating sample was processed independently with no exchange of data or results until final age estimates had been produced to ensure that the results were generated in isolation.

Did the study involve field work? ☒ Yes ☐ No

Field work, collection and transport

Field conditions	Fieldwork was conducted between July-August and occasionally in December in the Guangxi ZAR region of southern China. The southern hemisphere summer months were hot with occasional rain, while the December trips were cooler and drier. Excavations and sampling were conducted on rugged steep karst terrain, often involving climbing and abseiling activities.
Location	The two study areas are located in a radius of ~100-40 km from Nanning – first around Chongzuo city (22°37'N, 107°37'E), southwest of Nanning and the second close to Bubingzhen town in Bubing Basin (23°35'N, 106°59'E) to the north west of Nanning both in Guangxi ZAR in southern China.
Access & import/export	Permission for excavation and sampling were obtained by the Institute of Vertebrate Paleontology and Paleoanthropology, Chinese Academy of Sciences, Beijing, via a permit issued by The National Bureau of Cultural Relics in Beijing #3312044 (2010-present) and via a collaborative agreement in conjunction with the Chongzuo Museum (signed 8/10/2018). Sediment samples were exported to Australia using Bio-security permits #IP0000522151 and IP0002282095. G. blacki teeth were imported to Australian by Zhang with permission from IVPP and the Chinese Academy of Sciences permit #[2018]9335 for the purpose of dating and analysis.
Disturbance	Mostly this study sampled caves that were already excavated by local institutions so the disturbance to the landscape was minimal. For the two caves excavated during the 2017-19 efforts were made to minimise the impact of the local communities and excavated sediments were backfilled. All excavation procedures and techniques followed national and local regulations.

Reporting for specific materials, systems and methods

We require information from authors about some types of materials, experimental systems and methods used in many studies. Here, indicate whether each material, system or method listed is relevant to your study. If you are not sure if a list item applies to your research, read the appropriate section before selecting a response.

Materials & experimental systems

n/a	Involved in the study	
☒	☐	Antibodies
☒	☐	Eukaryotic cell lines
☐	☒	Palaeontology and archaeology
☒	☐	Animals and other organisms
☒	☐	Clinical data
☒	☐	Dual use research of concern
☐	☒	Plants

Methods

n/a	Involved in the study	
☒	☐	ChIP-seq
☒	☐	Flow cytometry
☒	☐	MRI-based neuroimaging

Palaeontology and Archaeology

Specimen provenance	Fossils were recovered from excavations at 22 caves in the Guangxi ZAR region of southern China. More details about the specimen numbers and provenance of these fossils is included in the Supplementary Information. A full list of permits for the excavations can be found in the "Access and import/export" section above.
Specimen deposition	Fossils recovered from the 2000-2019 excavations at Chongzuo are housed at the Institute of Vertebrate Paleontology and Paleoanthropology, Chinese Academy of Sciences, Beijing, while those collected in Bubing Basin (1999-2016) are housed at Natural History Museum of Guangxi, Nanning, China. Access to these fossil can be sought via these institutions.

Dating methods	The Methods Summary and Supplementary Information provides detailed descriptions of each dating method. Uranium-series dating of the speleothem samples was conducted in the Radiogenic Isotope Facility of The University of Queensland and the Wollongong Isotope Geochronology Laboratory, at University of Wollongong, Wollongong using VG Sector 54 thermal ionisation mass spectrometer (TIMS) and a Nu Plasma multi-collector inductively coupled mass spectrometer (MC-ICP-MS). All luminescence analysis, OSL and pIR-IRSL, was conducted at the "Traps" luminescence dating facility at Macquarie University in Sydney, Australia using a TL-DA-20 Luminescence reader. Laser ablation mass spectrometry to measure U-series isotopes along the teeth were conducted at Southern Cross University, Australia. Additional U-series measurements and ESR measurements were undertaken at GARG Southern Cross University. ESR dating of quartz was undertaken at National Research Centre on Human Evolution CENIEH, Burgos, Spain.

☒ Tick this box to confirm that the raw and calibrated dates are available in the paper or in Supplementary Information.

Ethics oversight	No ethical approval was required for this study as the specimens have been fossilized for many thousands of years

Note that full information on the approval of the study protocol must also be provided in the manuscript.

Dual use research of concern

Policy information about dual use research of concern

Hazards

Could the accidental, deliberate or reckless misuse of agents or technologies generated in the work, or the application of information presented in the manuscript, pose a threat to:

No	Yes	
☒	☐	Public health
☒	☐	National security
☒	☐	Crops and/or livestock
☒	☐	Ecosystems
☒	☐	Any other significant area

Experiments of concern

Does the work involve any of these experiments of concern:

No	Yes	
☒	☐	Demonstrate how to render a vaccine ineffective
☒	☐	Confer resistance to therapeutically useful antibiotics or antiviral agents
☒	☐	Enhance the virulence of a pathogen or render a nonpathogen virulent
☒	☐	Increase transmissibility of a pathogen
☒	☐	Alter the host range of a pathogen
☒	☐	Enable evasion of diagnostic/detection modalities
☒	☐	Enable the weaponization of a biological agent or toxin
☒	☐	Any other potentially harmful combination of experiments and agents

Plants

Seed stocks	Fossilized pollen samples were collected from the cave sediments directly at the ANU palaeoecology laboratory
Novel plant genotypes	N/A
Authentication	N/A
